# Targeted Modulation of *d*‐Band Center in MoS_2_ Interlayer With n‐Type Co/Fe Dopants Accelerating Sulfur Reaction Kinetics in Lithium‐Sulfur Batteries

**DOI:** 10.1002/adma.73445

**Published:** 2026-05-21

**Authors:** Junhyuk Ji, Sangyeon Won, Jaehyeong Yu, Nuri Moon, Dongwoo Kim, Junbeom Maeng, Won Bae Kim

**Affiliations:** ^1^ Department of Chemical Engineering Pohang University of Science and Technology (POSTECH) Gyeongsangbuk‐do Republic of Korea; ^2^ Department of Battery Engineering Graduate Institute of Ferrous and Eco Materials Technology (GIFT) Pohang University of Science and Technology (POSTECH) Gyeongsangbuk‐do Republic of Korea

**Keywords:** *d*‐band center, *d‐p* hybridization, interlayer, Li‐S batteries, MoS_2_ catalyst, n‐type dopant

## Abstract

Although chalcogenide‐based catalysts offer significant potential for enhancing lithium‐sulfur (Li‐S) battery performance, the absence of reliable descriptors linking the *d*‐band center to sulfur conversion kinetics hinders the rational design of electrochemical systems. Herein, we address this limitation by engineering a catalytic interlayer through modification of 2H‐MoS_2_ electronic structure, achieved via substitutional doping of n‐type Co/Fe transition metals (TM) at Mo sites. Comprehensive findings elucidate that such doping initiates a distinct S‐mediated *d*‐*p* hybridization involving Mo 4*d—*S 3*p—*TM 3*d* orbitals, thereby modulating electronic density of states near the Fermi level. Specifically, in the CoFe‐MoS_2_@carbon paper (CP) interlayer, synergistic effect of co‐doping with two different TM drives optimized downshift of the Mo 4*d*‐band center to intermediate energy states, fostering moderate catalyst‐reactant interaction. Furthermore, the simultaneously lowered S 3*p*‐band center enhances the degree of *d*‐*p* orbital overlap. These electronic redistributions enhance both electrical and ionic conductivity, thereby facilitating accelerated redox kinetics with reduced activation energy, while mitigating the shuttle effect and promoting uniform Li_2_S deposition. Consequently, the assembled cell delivers outstanding stability with a low decay rate of 0.024% for 2000 cycles even at 10C. This work emphasizes that a balanced *d*‐band center is key to achieving highly active chalcogenide‐based materials for advanced Li‐S batteries.

## Graphical abstract

A targeted modulation of the MoS_2_ electronic structure is achieved via substitutional n‐type Co/Fe co‐doping. This strategy triggers S‐mediated *d*‐*p* hybridization, optimizing the binding affinity with polysulfides to establish a volcano‐shaped relationship between the *d*‐band center and catalytic activity. Consequently, the designed interlayer promotes rapid redox kinetics and uniform Li_2_S deposition, enabling ultra‐stable operation under high‐rate conditions for Li‐S batteries.

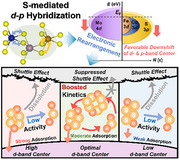

## 1 Introduction

The ever‐growing market quest for high‐energy, cost‐efficient storage systems has placed lithium‐sulfur (Li‐S) batteries at the forefront of research for electric vehicles and clean energy applications due to their potential to achieve practical energy densities exceeding 400 Wh kg^−1^ with their high theoretical specific capacity up to 1675 mAh g^−1^ [1, 2]. However, several persistent technical challenges hinder the practical deployment of Li‐S batteries. These include the inefficient conversion of lithium polysulfides (LiPSs) to Li_2_S due to large energy barriers, detrimental shuttle effect causing anode corrosion, and high internal resistance stemming from the low conductivity of active sulfur and its products, leading to poor discharge capacity and cycling stability [3, 4]. Consequently, despite a decade of dedicated research, the gap between laboratory performance and commercial prerequisites remains substantial [5, 6].

Given the consensus that solid‐liquid‐solid phase transition involved in the Li‐S chemistry necessitates several sequential adsorption and reduction of LiPS intermediates, a primary research strategy dedicates to regulation of an electrochemical cell system with catalytically active materials to expedite each stage of the conversion [7]. The catalyst plays a dual role: first, by accelerating LiPS conversion to facilitate the handling of insulating species (Li_2_S_2_/Li_2_S), which increases capacity, and second, by chemically interacting with soluble LiPS to suppress the loss of active materials, thus improving stability [8, 9]. Hence, in recent years, high‐polarity transition metal (TM) compounds, including oxides, sulfides, nitrides, carbides, and phosphides, have been widely utilized in Li‐S batteries [10, 11, 12, 13, 14]. Especially, distinguished by the presence of soft basic S^2−^/S_2_^2−^ anions rather than hard basic O^2−^ ions, TM sulfides possess a relatively higher density of valence electrons toward effective LiPS binding sites compared to that of TM oxides, where the intrinsic characteristic leads to their extensive study [11, 15]. Among them, two‐dimensional (2D) TM dichalcogenides (TMDCs), with a particular emphasis on 2H‐phase MoS_2_ (2H‐MoS_2_), have garnered attention within the catalysis community. Their prominence is attributed to a combination of advantageous features such as ease of synthesis, superior catalytic efficacy, strong adsorption, and distinct physicochemical properties arising from their intrinsic semiconducting band gap [16, 17, 18]. However, the standalone application of pristine 2H‐MoS_2_ faces fundamental bottlenecks to maximize catalytic interactions: (1) its semiconducting nature resulting in poor electrical conductivity, and (2) the spatial limitation of active sites, as catalysis occurs predominantly on edges while the basal plane is relatively inert [19, 20, 21].

One main approach to resolving such intrinsic problems of the Li‐S batteries involves establishing a relationship model between the *d*‐band center position and electrochemical performance through a systematic analysis of the mechanism how the *d*‐band center influences LiPS adsorption strength and catalytic conversion kinetics [8, 22]. According to the *d*‐band theory, catalytic activity is primarily determined by the electronic structure of the metal *d*‐orbitals, where the adsorption energy of LiPSs serves as a critical determinant of both catalytic activity and overall Li‐S battery performance [23, 24]. Thus, fine‐tuning the *d*‐band electronic structure of a catalyst toward desirable energy levels can offer a pathway to significantly augment its catalytic capability during cell operation [22, 25]. Considering previous studies, heteroatom doping into TMDCs is identified as a key strategy, as it induces significant electronic perturbations that effectively modulate the *d*‐band center. For instance, the influence of P‐doping on Li‐S battery catalysts was investigated using a P‐doped three‐dimensional (3D) MoS_2_ network, revealing that the strong hybridization of P 2*p—*Mo 3*d—*S 2*p* orbitals creates new electronic defect levels at the Fermi energy level (*E*_F_) [26]. This facilitates charge transfer and increases electrical conductivity, thereby inducing an active basal plane for high catalytic capacity. Moreover, p‐type V‐doped and n‐type Mn‐doped MoS_2_ catalysts were designed to shift the *E*_F_ downward and upward, respectively [27]. This strategy was employed to investigate the *d*‐band center as an important descriptor by comparing the adsorption strength and the deposition/decomposition behavior of LiPSs. Meanwhile, an O‐doped engineering approach could be applied to achieve pillar‐free interlayer extension of MoS_2_ for polysulfide conversion, which showed that the strengthened hybridization between Mo *d—*S *p* orbitals induced by O‐doping, thereby reducing the band gap at the *E*_F_ [28]. This, in turn, improves intrinsic conductivity and facilitates ion transport critical for the LiPSs conversion. Extending beyond single‐element doping, simultaneous cationic and anionic modification of MoS_2_ with Ni and Se was shown to shift the *d*‐band center upward and expand the interlayer spacing. This synergistic tuning enables a targeted catalysis effect, lowering the conversion energy barrier for specific polysulfides [29]. Despite numerous attempts using various heteroatom doping strategies to enhance the conductivity and catalytic activity of MoS_2_ catalysts, there is still a significant lack of specific research on establishing the optimal *d*‐band center value in Li‐S batteries. Guided by the Sabatier principle, it is significant to maintain a sustainable and stable chemical reaction which requires for the interaction between intermediate reactants and the catalyst surface to be neither too strong nor too weak for continuous adsorption and desorption [30, 31]. Therefore, the optimization of the electronic structure is essential, ultimately serving as a critical descriptor for efficient catalyst design process.

**FIGURE 1:**
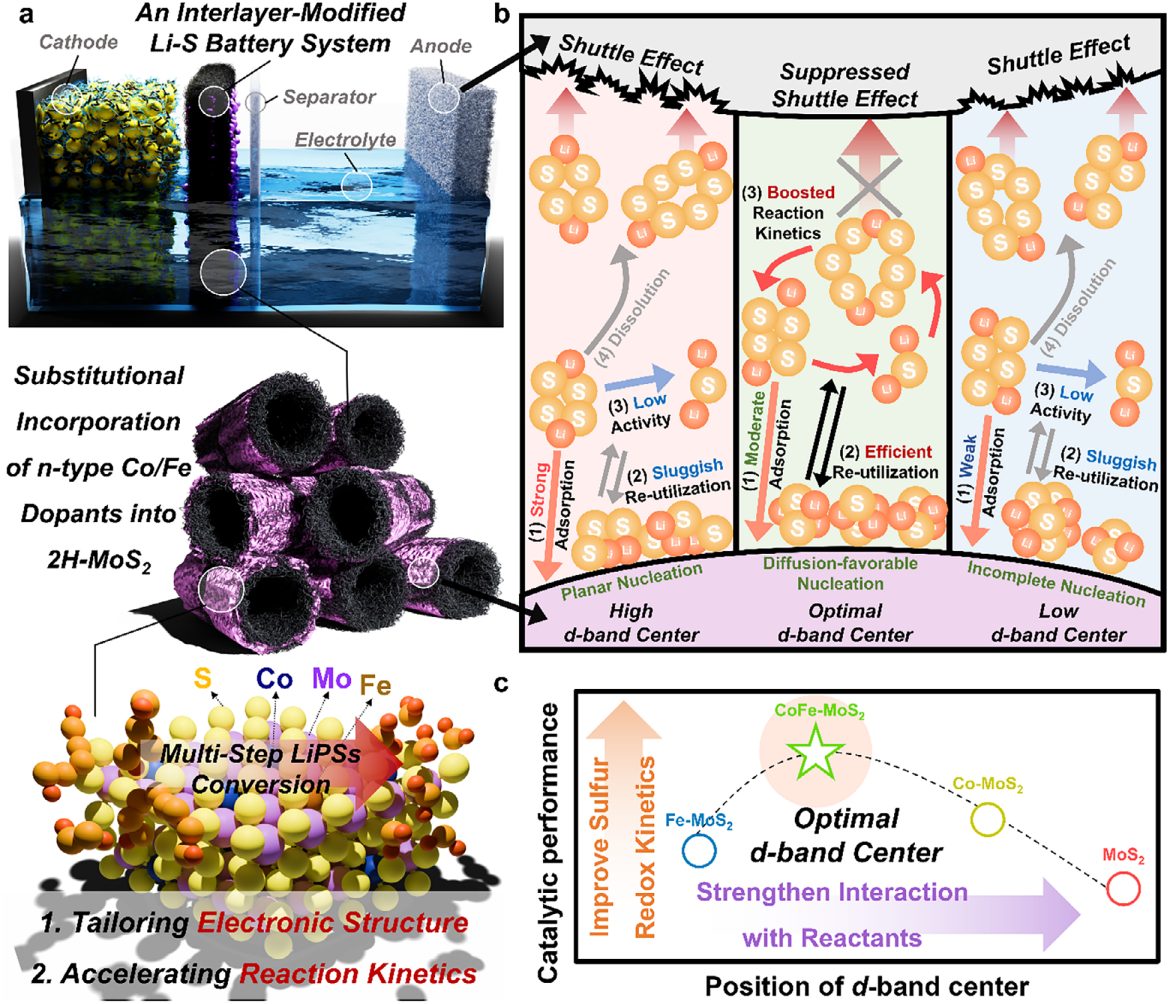
Schematic illustrations for the configuration of the Li‐S battery system, the catalyst interlayer, and the sulfur redox kinetics. (a) Configuration of the interlayer with the CoFe‐MoS_2_ catalyst depicted at both observable and atomic scales. (b) The *d*‐band center effects as a descriptor for sulfur redox reaction efficiency in Li‐S batteries. (c) A qualitative comparison with a volcano‐shaped relationship between the computed *d*‐band center position and the experimentally measured catalytic activity during the Li‐S battery operation.

In this work, we report the development of a highly efficient catalytic interlayer for Li‐S batteries with boosted LiPS conversion kinetics, achieved through the strategic engineering of the 2H‐MoS_2_ electronic structure via Co and Fe co‐doping (Figure 1a). Combined experimental and theoretical analyses reveal that the substitution of n‐type TM dopants at Mo sites triggers a unique S‐mediated *d*‐*p* hybridization among Mo 4*d* ‐S 3*p—*TM 3*d* orbitals, which can effectively modulate the electronic density of states (DOS) [29, 32]. In particular, the synergy arising from the simultaneous incorporation of Co and Fe dopants enables an optimized downward shift of the Mo 4*d d*‐band center, positioning it at an intermediate energy level. The reconfigured electronic structure can overcome the traditional trade‐off between adsorption energy and desorption kinetics by acting to moderate the binding affinity between the catalyst and polysulfides (Figure 1b). Compared to catalysts that induce too strong or too weak interactions, a catalyst with an optimal *d*‐band center can promote diffusion‐favorable Li_2_S nucleation to allow the re‐participation of sulfur species in the reaction while suppressing dissolution into the electrolyte. The resultant volcano‐shaped relationship between the position of the *d*‐band center and catalytic performance of the MoS_2_‐based catalysts underscores the necessity of a fine‐tuned electronic structure for realizing optimal battery performance (Figure 1c). In addition, the electronic redistribution also shifts the S 3*p p*‐band center to lower energy, which increases the electronic overlap ratio between *d*‐PDOS and *p*‐PDOS relative to 2H‐MoS_2_, ultimately facilitating improved electrical and ionic transport. Therefore, the carbon paper (CP) interlayer functionalized with Co and Fe co‐doped MoS_2_ (CoFe‐MoS_2_@CP) enables rapid redox kinetics during the subsequent LiPS conversion process due to minimized activation energy (*E*_a_) while effectively suppressing the shuttle effect and regulating Li_2_S deposition to be uniform. These atomistic benefits manifest as superior electrochemical properties, evidenced by capacity retentions of 43.1% and 47.5% over 2000 cycles at 5C and 10C, corresponding to minimal decay rates of 0.028% and 0.024% per cycle, respectively. Furthermore, when subjected to a high sulfur loading (6.2 mg cm^−2^) and a low electrolyte/sulfur (E/S) ratio (9.7 µL mg^−1^), the CoFe‐MoS_2_@CP cell achieves an outstanding areal capacity of 5.15 mAh cm^−2^ at 0.1C with high stability by retaining 76.4% of its capacity after 200 cycles at a low decay rate of 0.118% per cycle. The proposed approach enables a stable full‐cell operation for practical feasibility under practical conditions (sulfur loading: 6.8 mg cm^−2^, E/S: 8.8 µL mg^−1^, N/P: 2.2), as shown by an initial capacity of 6.22 mAh cm^−2^ and a retention of 4.21 mAh cm^−2^ over 100 cycles. This targeted *d*‐band modulation approach will offer a design guideline for advanced chalcogenide‐based catalysts by bridging the gap between orbital‐level engineering and macroscopic electrochemical performance for the realization of practical Li‐S batteries.

## 2 Results and Discussion

### 2.1 Synthesis and Physicochemical Analysis of Co and Fe Co‐Doped MoS_2_

**FIGURE 2:**
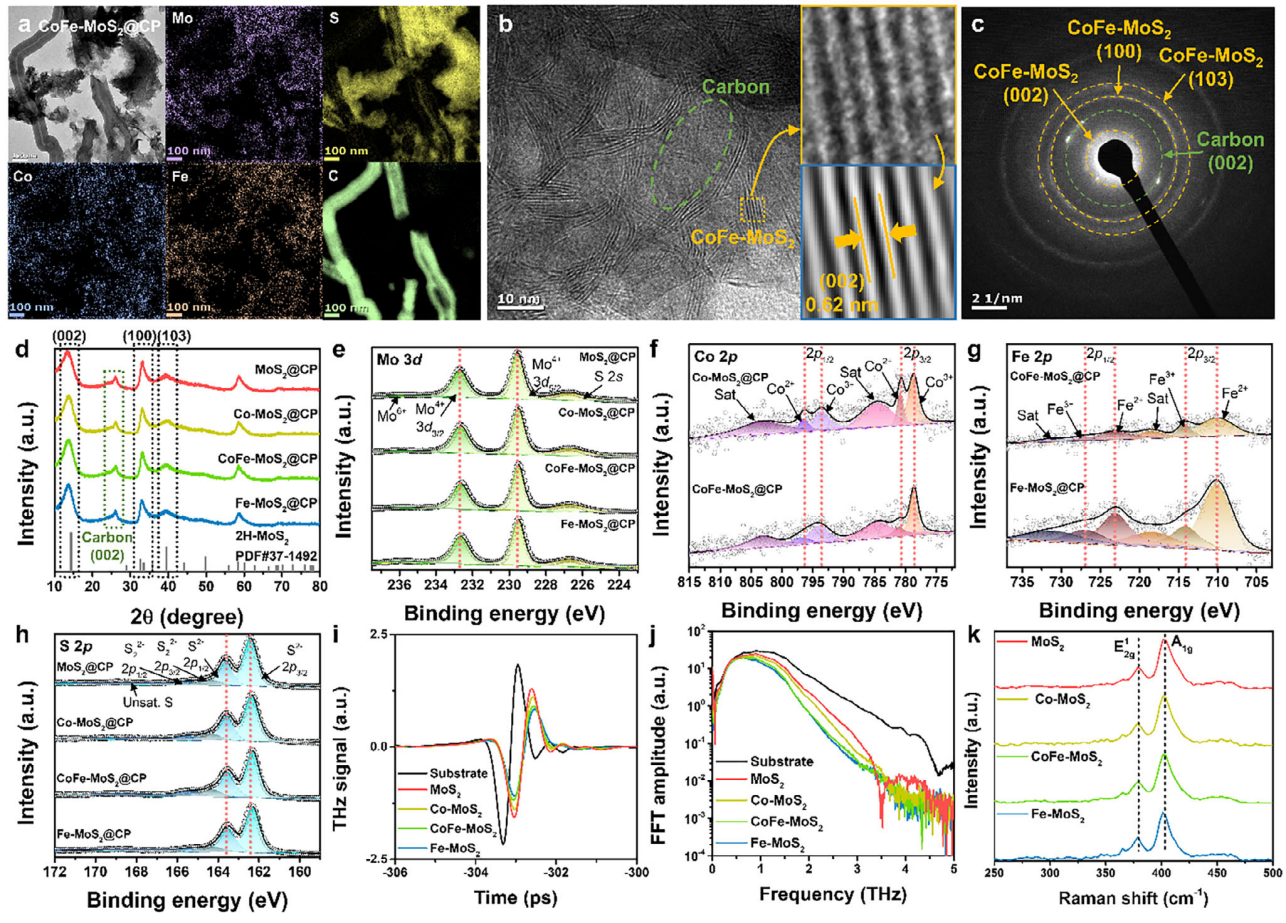
Physicochemical characterization of materials. (a) TEM image with corresponding EELS mappings of the CoFe‐MoS_2_@CP catalyst. (b) HR‐TEM image showing the separated domains within the structure, and (c) SAED pattern identifying the specific crystalline planes of the CoFe‐MoS_2_@CP catalyst. (d) XRD patterns of the MoS_2_@CP, Co‐MoS_2_@CP, CoFe‐MoS_2_@CP, and Fe‐MoS_2_@CP materials. XPS profiles of the catalysts for (e) Mo 3*d*, (f) Co 2*p*, (g) Fe 2*p*, and (h) S 2*p* regions. (i) The time‐domain spectra and (j) frequent‐domain spectra of the catalysts measured by the THz‐TDS method. (k) Raman spectra of the catalysts without carbon substrate.

In this study, the synthesis procedure of the CoFe‐MoS_2_ interlayer involved two‐steps process, beginning with a drop‐casting step of the Mo, Co, Fe, and S precursor solutions onto the CP layer, followed by a thermal annealing step to complete the catalyst interlayer (Figure S1, details in Experimental Section). The inductively coupled plasma‐optical emission spectroscopy (ICP‐OES) analysis revealed that the molar ratio of Mo, Co, and Fe in the CoFe‐MoS_2_ was 26.87:0.81:0.19, with the Co‐MoS_2_ and Fe‐MoS_2_ samples maintaining comparable doping proportions (Figure S2 and Table S1). Transmission electron microscope (TEM) images with electron energy loss spectroscope (EELS) mapping demonstrated that the catalysts were successfully formed and supported on the carbon substrate (Figure 2a and Figure S3). The high‐resolution TEM (HR‐TEM) image of the MoS_2_@CP sample demonstrated clear lattice fringes for both the 2H‐MoS_2_ and carbon phases. It showed a typical lattice interlayer distance of 0.62 nm corresponding to the (002) plane, complemented by selected area electron diffraction (SAED) patterns revealing the 2H‐MoS_2_ structure with distinct (002), (100), and (103) planes (Figure S4a,b). Even after the introduction of TM dopants, HR‐TEM and SAED patterns suggested that the 2H‐MoS_2_ structure was well preserved (Figure 2b,c). The (002) *d*‐spacing remained nearly invariant at 0.62 nm, indicating that the dopants were likely incorporated via cation substitution at Mo sites rather than lattice interlayer intercalation (Figure S5a–c) [17, 33]. The scanning electron microscope (SEM) images and energy dispersive spectroscope (EDS) mappings also demonstrated that the catalyst was well‐fabricated and deposited onto the fibers of CP (Figure S6a–c). The X‐ray diffraction (XRD) pattern showed that TM incorporation occurred without any phase change from the 2H structure (Figure 2d). Furthermore, the hexagonal 2H‐MoS_2_ characteristics were also observed via synchrotron high‐resolution XRD (HRPD) pattern measured on the catalyst powders, suggesting the CP serves as a substrate without inducing any phase deviation in the MoS_2_ (Figure S7) [11]. To elucidate the elemental composition and chemical bonding states of the fabricated catalyst interlayer, X‐ray photoelectron spectroscopy (XPS) measurements were performed. The C 1*s* spectra of all samples showed C─C, C─O─C, and O─C═O bonds, indicating that the catalysts were successfully supported on the carbon substrate without significantly altering its surface chemistry (Figure S8) [6, 34]. Compared to the un‐doped MoS_2_@CP, the Mo 3*d* spectra of Co‐MoS_2_@CP, CoFe‐MoS_2_@CP, and Fe‐MoS_2_@CP samples show minor redshifts, which might reveal a change in the local chemical environment to induce a charge transfer from TM to Mo (Figure 2e) [20, 35]. This transfer makes the TM more positive, imparts an n‐type doping character to the MoS_2_, and thus lowers the electron binding energy [35]. Moreover, in the Co 2*p* and Fe 2*p* spectra of the TM‐doped samples, the clear existence of TM^2+^/TM^3+^ 2*p*_3/2_/2*p*_1/2_ peaks, rather than peaks corresponding to TM^0^, indicates that Co and Fe were doped with specific oxidation states by substituting for Mo sites (Figure 2f,g) [21, 36]. The peak shift observable in the S 2*p* XPS spectra also indicates the change in the chemical environment of 2H‐MoS_2_ due to the successful partial replacement of Mo sites with Co/Fe dopants (Figure 2h) [20, 35]. Furthermore, time‐ and frequency‐domain spectra of femtosecond laser‐induced terahertz time‐domain spectroscopy (THz‐TDS) showed smaller amplitude intensities for all TM‐doped MoS_2_ samples compared to the sapphire substrate and MoS_2_ (Figure 2i,j). Moreover, an analysis of the DC electrical conductivity (*σ*_DC_) derived from THz‐TDS data via the bulk transmission model indicates higher conductivity values for the doped systems (Figure S9a). Similarly, the slopes calculated from 4‐probe powder conductivity measurements for the catalysts in a carbon‐adsorbed interlayer state followed this trend, where the MoS_2_@CP displayed the lowest value (Figure S9b). This demonstrates that the Co and Fe doping strategy can enhance electron transfer efficiency by modifying the electronic structure of semiconducting MoS_2_, which in turn can contribute significantly to an improved catalytic performance [37, 38]. The Raman spectra showed characteristic peaks at ca. 381 and ca. 401 cm^−1^ for in‐plane (E^1^_2g_) and out‐of‐plane (A^1^_g_) phonon vibration modes, respectively, suggesting that all materials show lattice features similar to pristine MoS_2_ by maintaining the 2H‐phase even after doping (Figure 2k) [39, 40]. Furthermore, the slight redshift observed in the E^1^_2g_ and A^1^_g_ modes is attributed to TM dopants in the MoS_2_ lattice, which could soften the Mo─S bonds and reduce their vibrational frequency [41]. The Brunauer‐Emmett‐Teller (BET) analysis revealed diminished surface areas (64.1–70.2 m^2^ g^−1^) and pore volumes (0.101–0.111 cm^3^ g^−1^) for all catalyst‐loaded samples of MoS_2_@CP, Co‐MoS_2_@CP, CoFe‐MoS_2_@CP, and Fe‐MoS_2_@CP relative to that of pristine CP (75.1 m^2^ g^−1^ for surface area and 0.115 cm^3^ g^−1^ for pore volume), which is attributed to the introduction of catalyst particles onto the fiber structure (Figure S10a,b; Table S2). It was also noted that while doping slightly altered the textural properties relative to those of the MoS_2_@CP, the composites demonstrated comparable properties among themselves.

### 2.2 Catalytic Gains of the Fabricated Interlayers for High Electrochemical Performance

**FIGURE 3:**
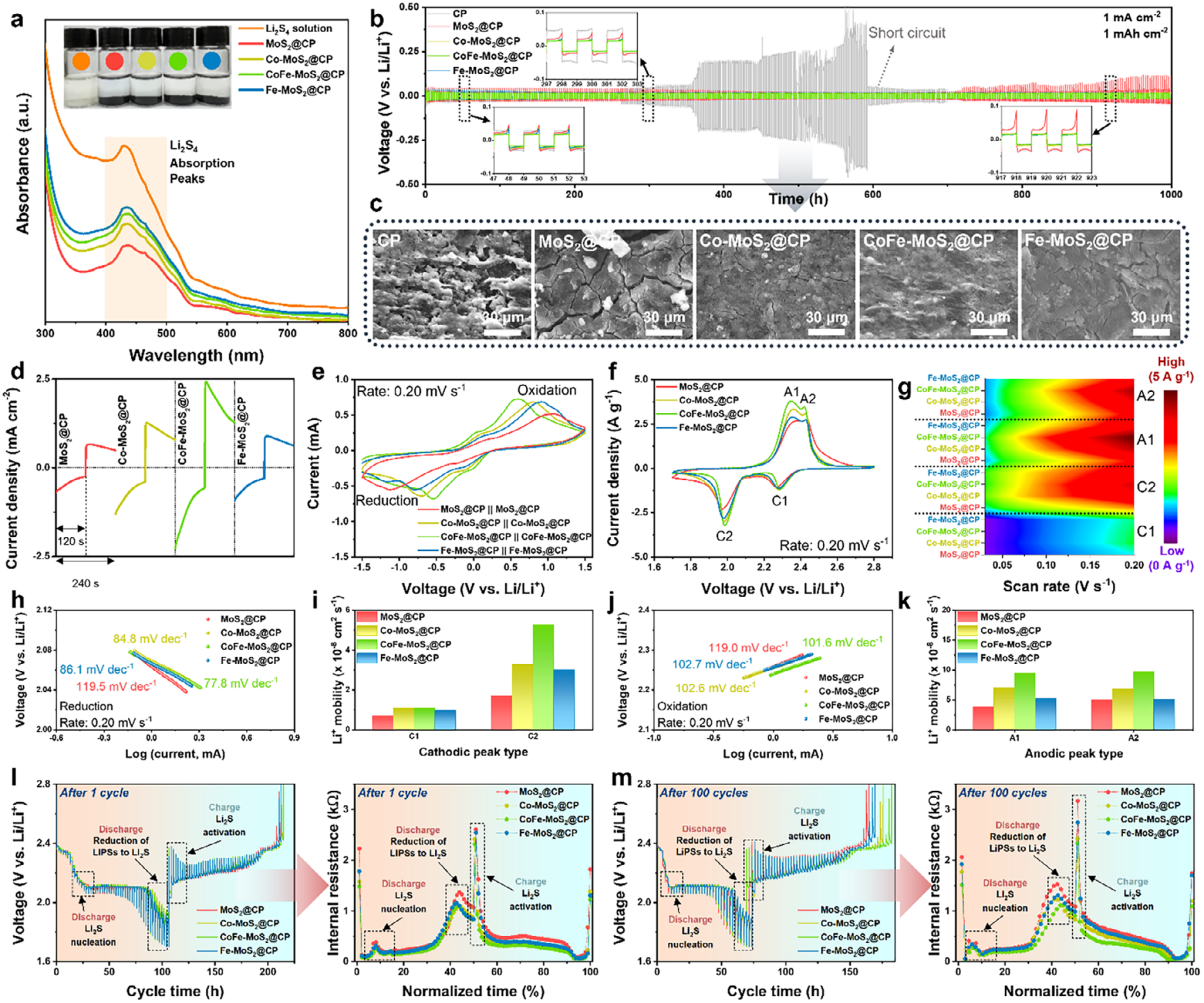
(a) Li_2_S_4_ adsorption test and resultant UV–vis spectra for MoS_2_@CP, Co‐MoS_2_@CP, CoFe‐MoS_2_@CP, and Fe‐MoS_2_@CP catalysts. (b) Cycling performance of Li||Li symmetric cells at 1 mA cm^−2^, 1 mAh cm^−2^ for 1000 h with detailed voltage profiles, and (c) corresponding ex situ SEM images of Li metal for each cell after the cycling process. (d) Chronoamperometric profiles of symmetric cells with Li_2_S_6_ electrolyte, utilizing the identical interlayers for both electrodes. CV profiles for (e) symmetric and (f) asymmetric cells with catalytic interlayers. (g) Comprehensive visualization of the CV profiles, highlighting the peak intensities associated with each redox reaction across a range of scan rates. Tafel plots and calculated Li^+^ mobility of the cells for the (h, i) reduction process and (j,k) oxidation process. GITT profiles of time‐voltage curves and calculated internal resistances as a function of normalized time for the cells at the (l) first cycle and (m) 100th cycle.

As a first step, the Li_2_S_4_ adsorption capability of the MoS_2_@CP, Co‐MoS_2_@CP, CoFe‐MoS_2_@CP, and Fe‐MoS_2_@CP was investigated, as this is considered a key factor for enhancing the overall redox kinetics during cycling in the electrolyte (Figure 3a). In detail, considering that Li_2_S_4_ acts as the bridge intermediate during the solid(S_8_)‐liquid(Li_2_S_x_)‐solid(Li_2_S_2_, Li_2_S) reaction, its efficient adsorption is a prerequisite for facilitating the subsequent 2.1 V‐level conversion step, which accounts for 75% of the discharge capacity [42]. Also, recent statistical analysis reveals that Li_2_S_4_ binding energy has the potential to possess a direct linear correlation with the sulfur reduction reaction (SRR) overpotential [42]. After immersing the samples in the Li_2_S_4_ solution, the doped samples yielded less transparent solutions as resulted by higher UV–vis intensity, signifying weaker affinity. Specifically, Fe‐doping resulted in less adsorption than the Co‐doping case, indicating that intermediate adsorption could be tailored via the proper Co/Fe ratio. Furthermore, by applying the Beer‐Lambert law to the UV–vis intensities, the adsorbed amounts of Li_2_S_4_ over the MoS_2_, Co‐MoS_2_, CoFe‐MoS_2_, and Fe‐MoS_2_ catalysts were calculated to be 43.5, 32.9, 29.8, and 27.6 µmol g^−1^, respectively (Figure S11). Subsequent post‐mortem XPS analysis of the Li_2_S_4_‐adsorbed samples revealed the appearance of a polythionate peak alongside a decrease in overall XPS intensity, indicating the effective adsorption of Li_2_S_4_ (Figure S12a–c). Additionally, a distinct Li_2_S peak was observed for the CoFe‐MoS_2_@Li_2_S_4_ sample, demonstrating effective LiPS conversion capability on the catalyst surface [43]. Based on the Sabatier principle, this tunability is a critical factor to design a catalyst with an optimal binding strength to enhance redox reaction efficiency [30]. Also, a Li dendritic growth issue should be considered to achieve stable Li plating/stripping behaviors via regulation of Li^+^ flux because the utilization of Li metal for the anode material is essential due to lack of Li source within the cathode for the Li‐S battery system [44]. Meanwhile, the enhanced conductivity of a MoS_2_‐based interlayer can reduce the preferential sites for Li dendrite nucleation toward the stabilized anode side [16, 45]. In light of these facts, Li||Li symmetric cells were employed to evaluate the stabilizing effect of the interlayers on the Li metal anode during subsequent plating and stripping. For the pristine CP interlayer cell without a catalyst, an increase in polarization voltage was observed beginning at ca. 262 h, followed by a short circuit at ca. 592 h (Figure 3b). Notably, compared to other cells, the CoFe‐MoS_2_@CP catalyst interlayer enabled the cell to consistently maintain the lowest and most stable voltage hysteresis throughout the long‐term cell operation for 1000 h cycled at 1 mA cm^−2^, corresponding to 1 mAh cm^−2^. Post‐cycling morphological analysis with ex situ SEM images corroborated the electrochemical data (Figure 3c). To be specific, the CoFe‐MoS_2_@CP cell showed a smooth and uniform Li metal surface, validating its minimal polarization during cycling. Conversely, all the control group cells displayed severely damaged surfaces with abundant dendrites and cracks, as a clear sign of poor Li^+^ regulation [46, 47]. To elucidate the intrinsic reasons for the Li dendrite suppression, Li||Li symmetric cells were evaluated (Figure S13). The CoFe‐MoS_2_@CP interlayer exhibited the highest exchange current of 2.52 × 10^−3^ A in Tafel plots (Figure S13a), ensuring accelerated and homogenous Li plating/stripping. Furthermore, electrochemical impedance spectroscopy (EIS) measurements verify that this kinetically favorable interface remained highly stable with minimal resistance after 500 h of cycling (1 mA cm^−2^, 1 mAh cm^−2^), fundamentally inhibiting dendrite growth (Figure S13b). These results suggest that the CoFe‐MoS_2_@CP interlayer can induce a homogenous redistribution of Li^+^ flux. In detail, it shows a synergistic mechanism where the MoS_2_ host provides the basic framework for Li^+^ redistribution, while the Co/Fe dopants deliver the essential kinetic acceleration to stabilize Li metal anode surface with suppressed Li dendrite growth, highlighting its bifunctional merits in Li‐S batteries. The catalytic effect on LiPS conversion was investigated using symmetric cells consisting of a pair of the same interlayers as working and counter electrodes with a Li_2_S_6_ electrolyte. Chronoamperometry revealed a significant current response for all samples, indicative of a Faradaic process of lithiation/delithiation rather than double‐layer capacitance (Figure 3d) [48]. Especially, the CoFe‐MoS_2_@CP symmetric cell demonstrated the most notable current response, indicating the significant effect of promoting the conversion of polysulfides [49]. We applied cyclic voltammetry (CV) techniques over the same Li_2_S_6_ symmetrical cells at various sweep rates from 0.05 to 0.20 mV s^−1^ to further research the catalytic contribution of the CoFe‐MoS_2_@CP (Figure 3e; Figure S14a–c). The distinguished current response also reveals that sulfur redox reactions are the major factor instead of double‐layer capacitance [50]. Moreover, the CV plot of the CoFe‐MoS_2_@CP cell showing the highest current peaks with the smallest shifts of the distinct peak demonstrates its outstanding catalytic ability (Figure S15a–d) [11, 49]. Subsequently, CV analysis for asymmetrical Li‐S cells assembled with a typical cathode (see details in Supplementary Note S1) and the modified interlayers measured with a voltage range of 1.7–2.8 V (vs. Li/Li^+^) at various scan rates from 0.03 to 0.20 mV s^−1^ was also conducted, which shows two cathodic peaks of C1 [S_8_ → Li_2_S_x_ (4 ≤ x ≤ 8)], C2 [Li_2_S_x_ → Li_2_S (2 ≤ x ≤ 4)] and two anodic peaks of A1 [Li_2_S → Li_2_S_x_ (2 ≤ x ≤ 4)], A2 [Li_2_S_x_ (4 ≤ x ≤ 8) → S_8_] (Figure 3f,g; Figures S16a–d and S17a–d). The CoFe‐MoS_2_@CP cell demonstrated the highest peak current and the smallest polarization voltage between stepwise reduction and oxidation peaks, representing remarkable sulfur redox reaction kinetics. To further elucidate the reaction kinetics, two key factors of Tafel slope and Li^+^ diffusion coefficient, could be derived. The CoFe‐MoS_2_@CP cell demonstrated superior kinetics, as it showed the lowest Tafel slope (77.8 mV dec^−1^) and high Li^+^ diffusion coefficients (C1: 1.07 × 10^−8^; C2: 5.29 × 10^−8^ cm^2^ s^−1^) during the discharge (Figure 3h,i; FigureS18a–c). This trend was mirrored during the charge process, where it again showed the lowest Tafel slope (101.6 mV dec^−1^) and high diffusion coefficients (A1: 9.43 × 10^−8^; A2: 9.69 × 10^−8^ cm^2^ s^−1^) (Figure 3j,k; Figure S18d–f). These results demonstrate that the CoFe‐MoS_2_@CP catalyst interlayer significantly enhances Li^+^ mass transfer, leading to more efficient catalytic behavior during the redox reactions [25, 51]. The surface‐contributed currents were calculated to compare different capacity contributions (Equations S1 and S2, see details in Supplementary Note S2). The CoFe‐MoS_2_@CP cell displayed the highest slope values in the log(*v*) vs. log(*i*) plots for all redox peaks, signifying a more dominant surface‐controlled process (Figure S19a–d). Furthermore, the surface‐controlled fractions for the CoFe‐MoS_2_@CP cell are quantitatively larger than those of the other cathodes (Figures S20–S24). These findings imply that one of the reason for the superior catalytic activity of the CoFe‐MoS_2_@CP cell might stem from the appropriate Co/Fe doping ratio, facilitating an optimal level of LiPS adsorption/desorption process and resulting in accelerating the surface chemical reaction rate and enabling more efficient Li^+^ transfer [52, 53]. After that, the galvanostatic intermittent titration technique (GITT) was utilized to measure the internal resistances (Δ*R*) of the cells during the discharge and charge processes, comparing the first cycle and 100th cycle states (Figure 3l,m). Interestingly, the CoFe‐MoS_2_@CP cell maintained its fast reaction kinetics throughout extended cycling as demonstrated by its consistently smaller polarization values compared to other cells during all key redox processes, including Li_2_S nucleation, LiPSs reduction, and Li_2_S activation. Therefore, when the results of the LiPSs affinity and catalytic performance tests are considered collectively, it is evident that the doping state must be properly controlled, which is required to positively influence both catalytic activation and Li^+^ mass transfer by achieving an optimal LiPS adsorption strength, thereby enhancing the subsequent electrochemical performance [25, 54].

### 2.3 Electrochemical Performance of Li‐S Cells Equipped With the Designed Interlayers

**FIGURE 4:**
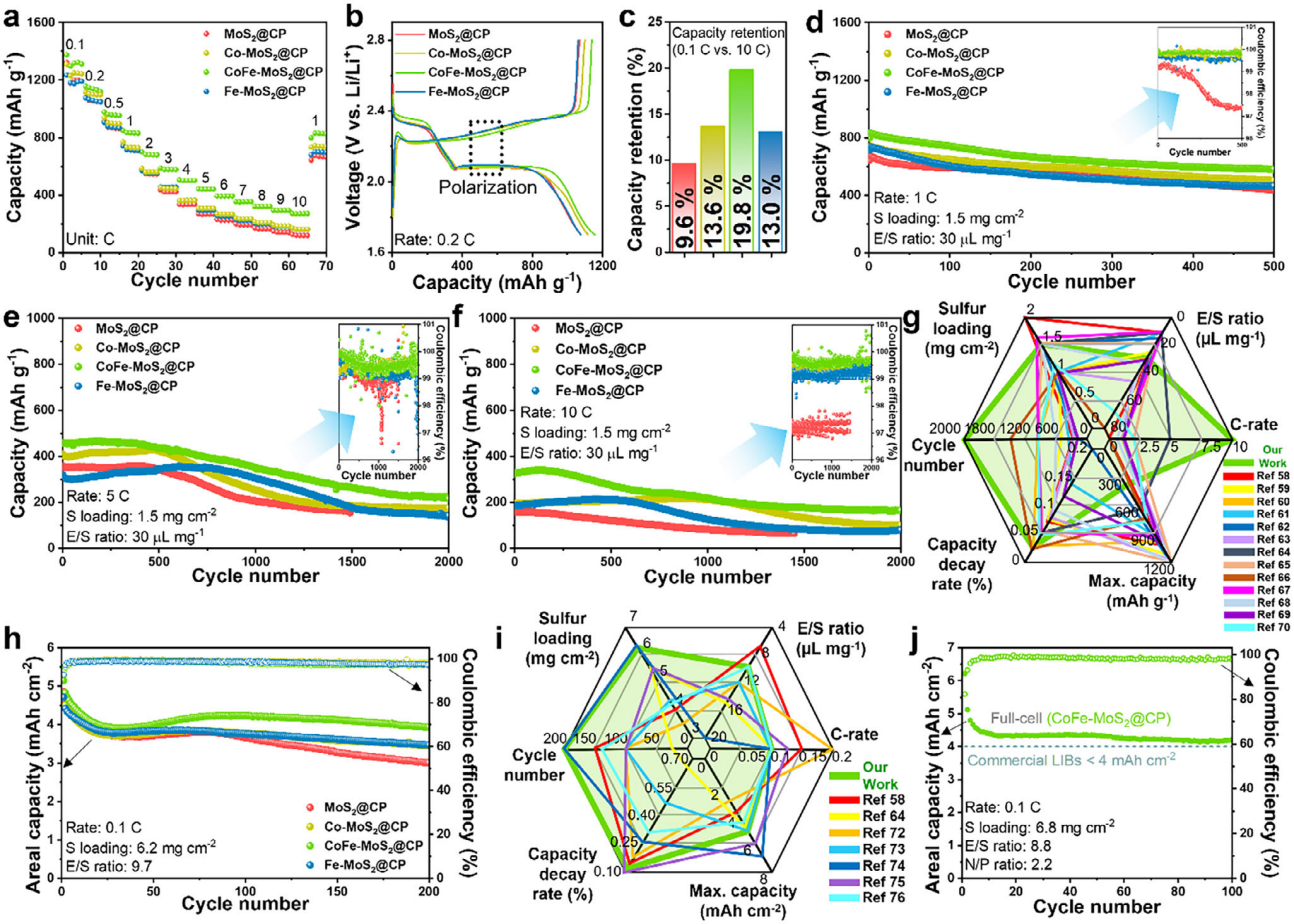
Electrochemical performance of Li‐S cells with various catalytic interlayers of the MoS_2_@CP, Co‐MoS_2_@CP, CoFe‐MoS_2_@CP, and Fe‐MoS_2_@CP. (a) Rate capability tests, (b) corresponding galvanostatic charge‐discharge profiles at 0.2C, and (c) capacity retention values derived from comparing the 0.1C and 10C data. Cycling performances of the cells at (d) 1C and at (e) 5C. (f) Long‐term cycling performance of the CoFe‐MoS_2_@CP cell under a 10C rate. (g) Comparison of electrochemical performance for the CoFe‐MoS_2_@CP cell with low sulfur loading in this work with that of previously reported literature for Li‐S cell. (h) Cycling performance at sulfur loading of 6.2 mg cm^−2^ and E/S ratio of 9.7 µL mg^−1^. (i) Comparison of electrochemical performance for the CoFe‐MoS_2_@CP cell with high sulfur loading in this work with that of previously reported literature for Li‐S cell. (j) Cycling performance of the Li‐S full‐cell incorporating the CoFe‐MoS_2_@CP interlayer at sulfur loading of 6.8 mg cm^−2^, E/S ratio of 8.8 µL mg^−1^, and N/P ratio of 2.2.

To investigate the catalytic influences on the Li‐S batteries driven by the optimized LiPSs‐catalyst interactions, a series of electrochemical measurements were carried out (Figure 4). As a first step, we carried out an optimization process to determine (1) the catalyst loading on the CP, (2) the doping weight percent, and (3) the Co/Fe doping ratio (see the detailed process in Supplementary Note S3). In the rate capability test, the bare CP interlayer cell failed to maintain its discharge capacity starting from 3C current density, and its galvanostatic charge‐discharge (GCD) profile showed very high voltage polarization as early as the 2C rate (Figure S25a,b). However, all of the cells with catalyst‐deposited CP interlayers showed higher charge and discharge capacities than those of bare CP cell. Among them, the CoFe‐MoS_2_@CP delivered outstanding rate capability at all C‐rates (0.1–10C) (Figure 4a and Figure S26). At 0.1, 0.2, 0.5, 1, 2, 3, 4, 5, 6, 7, 8, 9, 10, and second 1C, the cell demonstrated discharge and charge capacities of 1375.4, 1156.2, 977.9, 850.1, 700.2, 586.4, 505.2, 443.1, 393.1, 353.2, 321.1, 294.5, 272.0, and 799.3 mAh g^−1^, respectively. This was paired with the smallest voltage hysteresis at 50% depth of discharge (DoD) as 0.216, 0.184, 0.216, 0.269, 0.360, 0.418, 0.441, 0.435, 0.443, 0.450, 0.454, 0.452, 0.467, and 0.250 V, respectively, indicating minimal energy loss (Figure 4b; Figure S27a–n). The same cell also retained 19.8% of its initial capacity at 0.1C rate even with 100‐fold increase in current density at 10C rate (Figure 4c). In other hands, the cells equipped with the MoS_2_@CP, Co‐MoS_2_@CP, and Fe‐MoS_2_@CP interlayers showed relatively poor performance, yielding capacity retention values of only 9.6%, 13.6%, and 13.0%, respectively, accompanied by relatively higher polarization voltages across all C‐rates (Figure S28a–d). Meanwhile, the serial conversion reactions in the GCD profile are divided into two steps: the first plateau (I), where solid S_8_ converts to liquid Li_2_S_4_, and the second plateau (II), where liquid Li_2_S_4_ converts to solid Li_2_S. The efficiency of this second step is a critical metric reflected in the capacity ratio of region II, which theoretically constitutes 75% of the total discharge capacity [55]. Although the pristine MoS_2_@CP cell lost its distinct second plateau at 4C and the singly doped Co‐MoS_2_@CP and Fe‐MoS_2_@CP lost theirs at 5C, the CoFe‐MoS_2_@CP retained 14.9% ratio in region II even at 10C, highlighting its superior sulfur utilization (Figure S29a–n). Furthermore, even with increasing current density, the CoFe‐MoS_2_@CP cell maintained low overpotentials for these subsequent conversion reactions (Figures S30a–h and S31a–f). Figure S32 visually summarizes the electrochemical achievements from the rate capability tests. The data show the CoFe‐MoS_2_@CP cell consistently outperformed all controls in discharge capacity, polarization, and second plateau ratio. This is attributed to the synergistically facilitated sulfur redox kinetics and finely adjusted LiPSs adsorption capability, driven by the high catalytic activation and lowered internal resistance provided by the optimized TM doping environment. Subsequently, the cells were compared by cycling at 1C rate to assess long‐term stability. The cell using the bare CP interlayer likewise showed rapid discharge capacity decay and a poor Coulombic efficiency trend, once again demonstrating the necessity of using an appropriate catalyst (Figure S33a,b). Similarly, the poor performance of the MoS_2_@CP cell characterized by consistently low discharge capacity and a sharp drop in Coulombic efficiency during cycling, reinforces the need for introducing TM dopants to enhance catalytic activity (Figure 4d). The best performance was demonstrated by the CoFe‐MoS_2_@CP cell, which delivered an initial capacity of 830.8 mAh g^−1^ and maintained 70.0% retention after 500 cycles, with a minimal decay rate of 0.060% per cycle and stable Coulombic efficiency. This outperformed both the singly doped Co‐MoS_2_@CP (67.8% retention, 0.064% decay) and Fe‐MoS_2_@CP (63.1% retention, 0.074% decay) controls. Further evidence of the outstanding stability of the dual‐doped CoFe‐MoS_2_@CP cell could be seen in its GCD profiles, which show the smallest 50% DoD polarization over all cycles (Figures S34a–f and S35a–d). Furthermore, ex situ EIS measurements were performed to investigate the charge transfer resistance (*R*_ct_) at the pristine state and the 500‐cycled state during the long‐term cycling test at a 1C rate (Figure S36a,b). Prior to cycling, the resistances *R*_s_ and *R*_int_, ascribed to the electrolyte and interlayer, respectively, showed comparable values for all four cell types, suggesting that the cells share a virtually identical Li‐S configuration (Table S3). Meanwhile, the CoFe‐MoS_2_@CP cell exhibited the lowest *R*_ct_ value (43.7 Ω), which is attributed to enhanced intrinsic interfacial charge transfer kinetics due to the facilitation of faster electron and ion transport across the electrode‐electrolyte interface [11, 25, 56]. Even after 500 cycles, the *R*_ct_ value for the CoFe‐MoS_2_@CP cell (4.69 Ω) remained notably lower than those of the MoS_2_@CP (11.6 Ω), Co‐MoS_2_@CP (5.15 Ω), and Fe‐MoS_2_@CP (7.59 Ω) owing to efficient sulfur redox reaction toward facile Li_2_S nucleation/dissociation [5, 56]. Post‐mortem analysis of a morphological comparison via SEM images was performed by disassembling the cells cycled at 1 C for 500 cycles to observe the anode surface (Figure S37a–d). The anodes of the Co‐MoS_2_@CP and Fe‐MoS_2_@CP cells were characterized by a bumpy surface, and that of the MoS_2_@CP represented an unstable Li plating morphology with tremendous cracks and dendrites. In contrast, the CoFe‐MoS_2_@CP cell maintained a relatively even and dense Li distribution without significant cracks, suggesting that the enhanced catalytic efficiency suppresses the polysulfide shuttling phenomenon and minimizes anode corrosion [57]. Besides, although the MoS_2_@CP exhibits a high initial II/I plateau ratio due to its strong adsorption capability, the CoFe‐MoS_2_@CP cell consistently maintained a higher II/I plateau ratio and a lower overpotential over 500 cycles, further indicating efficient conversion to Li_2_S (Figure S38a–f and S39a–f). These results can affirm again that an optimal Co and Fe doping strategy is crucial for introducing proper active sites that boost the LiPSs redox function (Figure S40). In addition, although the CoFe‐MoS_2_@CP cell demonstrated the best cycling stability at 5C rate after cell activation, delivering 466.4 mAh g^−1^ and retaining 43.1% even after 2000 cycles with a decay rate of only 0.028% per cycle and the smallest polarization, all the control cells performed poorly in comparison (Figure 4e; Figures S41, S42a–h and S43a–d). Specifically, the MoS_2_@CP cell failed at 1494th cycle, while the Co‐MoS_2_@CP and Fe‐MoS_2_@CP cells showed lower retention (39.8% and 37.6%) and higher decay rates (0.030% and 0.031%), respectively (Figure S44). Even when the long‐term cycling test was conducted at a very fast 10C rate with activation cycle process, the CoFe‐MoS_2_@CP cell showed a high capacity of 340.9 mAh g^−1^ along with 47.5% capacity retention and a 0.024% decay rate per cycle over 2000 cycles (Figure 4f; Figure S45). However, all control cells exhibited comparatively poor performance at 10C rate, suffering from low discharge capacities and high polarization voltages throughout the entire cycling process (Figure 4e; Figures S46a–d and S47). As illustrated in the benchmark plot under low sulfur loading condition, our cell exhibits outstanding performance compared to the previously reported cells when evaluated across metrics such as sulfur loading, E/S ratio, C‐rate, maximum capacity, decay rate, and cycle number (Figure 4g; Table S4) [58, 59, 60, 61, 62, 63, 64, 65, 66, 67, 68, 69, 70, 71]. Additionally, even under harsh conditions of sulfur loading of 6.2 mg cm^−2^ and E/S ratio of 9.7 µL mg^−1^, a high areal capacity of 5.15 mAh cm^−2^ was achieved at 0.1C with 76.4% capacity retention and only 0.118% decay rate per cycle over 200 cycles using the cell equipped with the CoFe‐MoS_2_@CP catalyst interlayer (Figure 4h; Figure S48a–d). When benchmarked against the previously reported cells including high‐loading data, our cell also demonstrates highly competitive performance, as illustrated in the plot (Figure 4i; Table S4) [58, 64, 72, 73, 74, 75, 76]. To evaluate its practical performance, a Li‐S full‐cell was constructed by pairing the CoFe‐MoS_2_@CP interlayer with a Li anode electrodeposited on a Cu substrate (Figure S49). The key parameters were set as 6.8 mg cm^−2^ sulfur loading, 8.8 µL mg^−1^ E/S ratio, and 2.2 N/P ratio. These parameters were set based on physicochemical limits; the 8.8 µL mg^−1^ E/S ratio ensures essential electrolyte wetting for the highly porous 3D CP interlayer, while the restrictive 2.2 N/P ratio addresses the rapid dead‐Li formation of the hostless Cu anode. The full‐cell delivered a remarkable performance, achieving an initial 6.22 mAh cm^−2^ and a final 4.21 mAh cm^−2^ after 100 cycles, corresponding to 914.2^−^ and 618.8 mAh g^−1^, respectively, which exceeds ca. 4 mAh cm^−2^ shown by typical commercial‐level Li‐ion batteries (Figure 4j; Figure S50). This achievement indicates its potential for industrial application with proper cell engineering. Thus, all the aforementioned electrochemical achievements of the CoFe‐MoS_2_@CP cell serve as a clear demonstration of how significantly the catalytic activity of 2H‐MoS_2_ can be enhanced through fine‐tuned TM doping strategy.

### 2.4 Discussion on the Rationale for Enhanced Electrochemical Performance

We have developed and systematically fabricated a highly active and stable CoFe‐MoS_2_@CP interlayer, as described in the prior sections. We successfully applied it to high‐performance Li‐S batteries by modulating the catalyst‐reactant interaction to accelerate LiPSs conversion kinetics. The cell achieved superior cyclic stability, with minimal capacity decay rates of only 0.028% and 0.024% per cycle at 5C and 10C, respectively, over 2000 cycles. Below, we will discuss the critical factors that rationalize this high catalytic performance.

**FIGURE 5:**
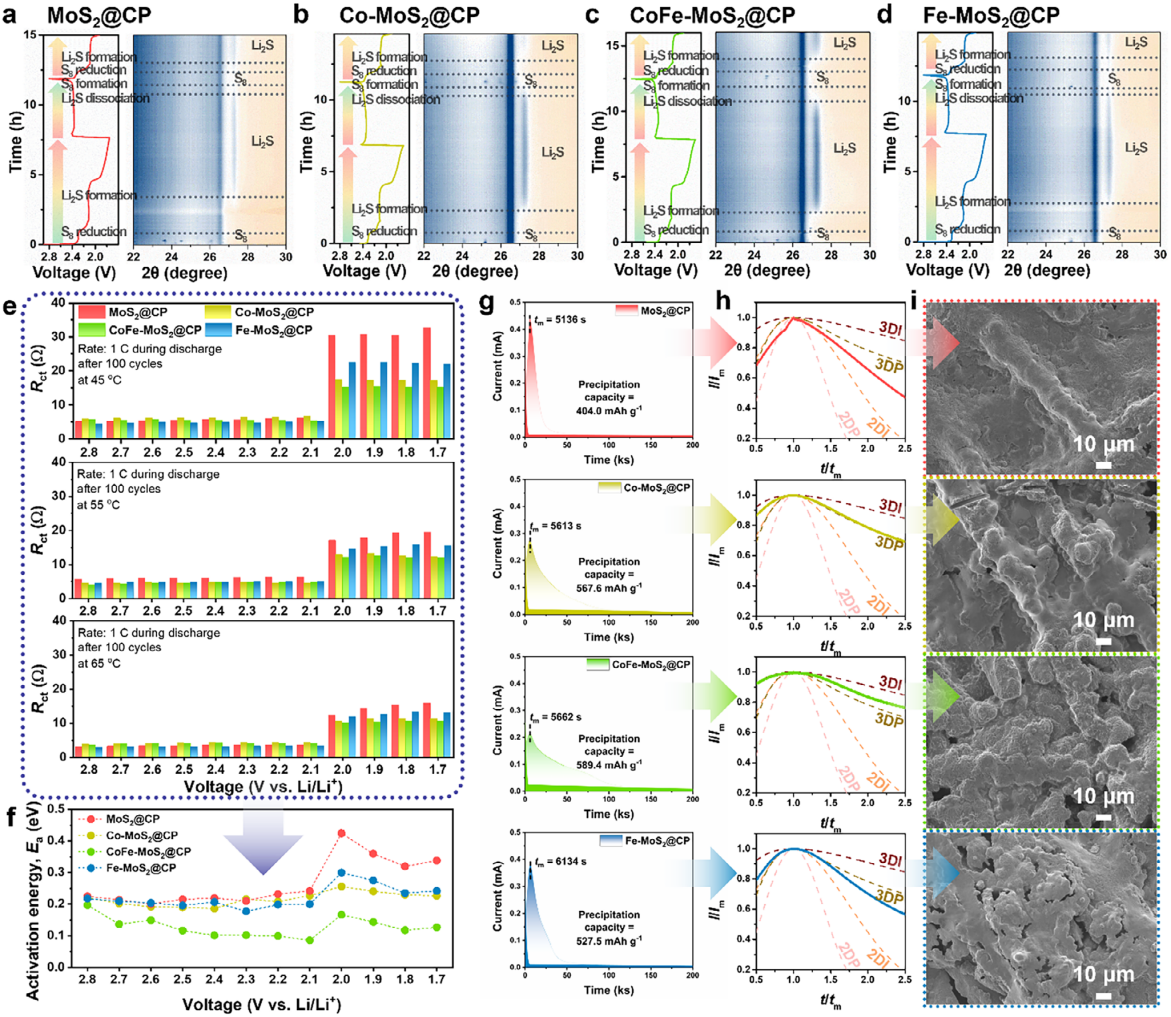
(a) In situ XRD analyses of the (a) MoS_2_@CP, (b) Co‐MoS_2_@CP, (c) CoFe‐MoS_2_@CP, and (d) Fe‐MoS_2_@CP cells at a 0.1C rate. (e) Variation of *R*_ct_ values of the cells during the discharging process at different temperature conditions of 45°C, 55°C, and 65°C, and (f) corresponding activation energy at each discharge step. (g) Potentiostatic discharge profiles of Li_2_S_6_ electrolyte solution at 2.03 V (vs. Li/Li^+^), (h) dimensionless current‐time curves, and (i) corresponding ex situ SEM images visualizing the morphology of Li_2_S deposited on the cathode surfaces.

The first point is the enhancement of kinetics for the phase transition of sulfur species during Li‐S cell operation, supported by the suppression of the polysulfide shuttle. To get a rationale for this, we monitored the polysulfide conversion kinetics using a synchrotron‐based operando XRD technique (Figure 5a–d). The CoFe‐MoS_2_@CP cell demonstrated a significant phase transformation from α‐S_8_ (222) to a distinct, newly nucleated crystalline cubic Li_2_S (111) peak (ca. 27.2°) early in the first discharge plateau. Conversely, solid‐phase Li_2_S nucleation was less distinct for the singly doped Co‐MoS_2_@CP and Fe‐MoS_2_@CP control cells, while for the un‐doped MoS_2_@CP cell, the product phases were not clearly observed overall. Therefore, the real‐time analyses indicate that the strategy of appropriately substituting Co and Fe for Mo in 2H‐MoS_2_ is strongly linked to enhanced Li_2_S conversion kinetics via catalytic activation, which is fully consistent with the previously investigated electrochemical behaviors that demonstrated improved conversion efficiency [77, 78]. To further understand the kinetics of each conversion step, we performed an in situ EIS investigation taken at various DoD states while the cells were discharged at 1C ranging from 45°C to 65°C with 10°C intervals, which allowed us to analyze the *E*_a_ using the Arrhenius equation (Figure S51). This real‐time methodology allows for the deconvolution of the electrochemical reaction kinetics influenced by the interlayers from the interfacial transport phenomena that concurrently take place during the reduction process [79]. The fitted *R*_ct_ results showed that all cells reached their maximum *R*_ct_ at ca. 2.0 V (vs. Li/Li^+^), which is a known phenomenon caused by the volume expansion and deposition of insulating Li_2_S, reducing conductive contact (Figure 5e; Tables S5–S7) [22]. Crucially, this same voltage also corresponds to the highest *E*_a_ value for all cells, signifying that the final conversion to solid Li_2_S can be considered as the rate‐determining step (Table S8). However, the CoFe‐MoS_2_@CP cell maintained a low *R*_ct_ during this phase transition with a relatively lower *E*_a_ (0.166 eV) than that of the MoS_2_@CP (0.424 eV), Co‐MoS_2_@CP (0.256 eV), and Fe‐MoS_2_@CP (0.300 eV) cells, suggesting the TM co‐doping strategy effectively regulated Li_2_S growth/passivation, controlled volume expansion, and ensured efficient charge transfer [79, 80]. Furthermore, the CoFe‐MoS_2_@CP cell showed the lowest *E*_a_ values across the entire DoD, demonstrating that the optimized interlayer accelerates sulfur redox kinetics, particularly in 2.0–2.4 V (vs. Li/Li^+^) range, overcoming the sluggish kinetics associated with phase transitions. To support the observed low resistance during the Li_2_S conversion for the CoFe‐MoS_2_@CP cell, the nucleation mechanism of Li_2_S on the catalysts could be examined, as this is a critical determinant of enhanced catalytic activation [12]. Given that the conversion of Li_2_S_n_ to Li_2_S constitutes a rate‐determining step affecting the subsequent charging process, it is important to evaluate the influence of each interlayer via potentiostatic discharge using a Li_2_S_6_ catholyte. The chronoamperometry curves show a characteristic current transient: an initial ascent to a peak attributed to the growth of Li_2_S nuclei, followed by a decay ascribed to surface passivation caused by the coalescence of these nuclei (Figure 5g) [81]. Given that the adsorption capability for LiPSs on the catalyst surface can influence the growth rate of Li_2_S nuclei, the MoS_2_@CP cell demonstrated the earliest current peak (*t*_m_ = 5136 s), while the Co‐MoS_2_@CP, CoFe‐MoS_2_@CP, and Fe‐MoS_2_@CP cells showed delayed peak times of 5613, 5622, and 6134 s, respectively, consistent with the findings from the adsorption tests (Table S9) [25]. A superior precipitation capacity of 589.4 mAh g^−1^ was obtained for the CoFe‐MoS_2_@CP cell with the optimized doping environment, driven by a well‐maintained current during the Li_2_S_6_‐to‐Li_2_S conversion. This performance compares favorably to the singly doped Co‐MoS_2_@CP (567.6 mAh g^−1^) and Fe‐MoS_2_@CP (527.5 mAh g^−1^) cells and significantly outperforms the un‐doped MoS_2_@CP cell (404.0 mAh g^−1^), which was hindered by sluggish kinetics. Subsequently, a dimensionless analysis of the current transients allowed us to classify Li_2_S nucleation into four growth modes: (1) 2D instantaneous growth (2DI), (2) 2D progressive growth (2DP), (3) 3D instantaneous growth (3DI), and (4) 3D progressive growth (3DP) (Figure 5h) [12, 81]. The 2D modes are indicative of planar deposition arising from the merging of adjacent atoms, whereas the 3D modes represent a volumetric diffusion‐controlled process that boosts the nucleation rate (See Supplementary Note S4). Consistent with the distinct deposition morphology observed in ex situ SEM images, although the CoFe‐MoS_2_@CP cell followed 3DP‐3DI nucleation model, the control cells of the MoS_2_@CP, Co‐MoS_2_@CP, and Fe‐MoS_2_@CP showed 2DI‐3DP, 3DP, and 2DI‐3DP model, respectively (Figure 5i). To physically substantiate these mathematical classifications, the spatial morphology and statistical size distribution of the Li_2_S nuclei were evaluated (Figures S52a–h and S53a–d). The progressive growth modes of the control cells inherently produced a low nucleation density accompanied by a broad variance in grain dimensions. Conversely, the 3DI‐dominated CoFe‐MoS_2_@CP cell generated a massively elevated nucleation density with an exceptionally narrow and uniform size distribution, indicating simultaneous, instantaneous precipitation. Thus, these results clearly demonstrate that the optimized TM active sites facilitate a balance between adsorption capacity and catalytic activation, which acts as the primary driver for significantly lowering the *E*_a_ value of the Li_2_S transformation process, aligned well with the results of the in situ EIS measurement. Moreover, as it is necessary not only to enhance the conversion efficiency of sulfur species but also to suppress the shuttle effect to prevent substantial capacity loss and anode corrosion for maximizing the performance of Li‐S batteries, we carried out shuttle current measurements to validate the migration effects of sulfur species during cycling (Figure S54a–d). The CoFe‐MoS_2_@CP cell maintained a relatively low shuttle current across the full voltage range, especially achieving values of 6.10 × 10^−7^, 7.24 × 10^−7^, and 7.33 × 10^−7^ mA at 2.40, 2.25, and 2.20 V (vs. Li/Li^+^), respectively (Figure S54e). The mitigation of polysulfide shuttling could be quantified by comparing the shuttle currents as the CoFe‐MoS_2_@CP cell showed values that were 3.45‐, 2.80‐, and 2.82‐fold lower than those of MoS_2_@CP; 1.33‐, 1.54‐, and 1.69‐fold lower than those of Co‐MoS_2_@CP; and 1.54‐, 1.48‐, and 1.74‐fold lower than those of Fe‐MoS_2_@CP. These findings imply that well‐targeted dopant substitution is highly effective in mitigating the shuttle effect and suppressing self‐discharge. Additionally, it might show a promise in regulating Li deposition to avoid noteworthy anode corrosion, supported by the SEM observations from both the Li||Li symmetric cell evaluation and the post‐mortem analysis of the long‐term cycled cells [82]. A Li_2_S dissolution experiment was also performed via potentiostatic charging to evaluate the catalytic efficacy during the oxidation process (Figure S55). Attributable to superior catalytic activity, the CoFe‐MoS_2_@CP cell achieved a decomposition capacity of 549.5 mAh g^−1^ and the shortest response time, surpassing that of the MoS_2_@CP (370.4 mAh g^−1^), Co‐MoS_2_@CP (485.4 mAh g^−1^), and Fe‐MoS_2_@CP (446.3 mAh g^−1^) controls. This performance comes from a reduced energy barrier for Li_2_S decomposition, a finding that is in good agreement with the GITT results indicating the lowest internal resistance for Li_2_S activation during the charge process [83]. Comprehensive results reveal that the rational substitution of Mo sites with Co and Fe dopants in the 2H‐MoS_2_ catalyst effectively inhibits unwanted sulfur dissolution into the electrolyte and concurrently enhances the kinetics of the overall sulfur conversion reactions.

**FIGURE 6:**
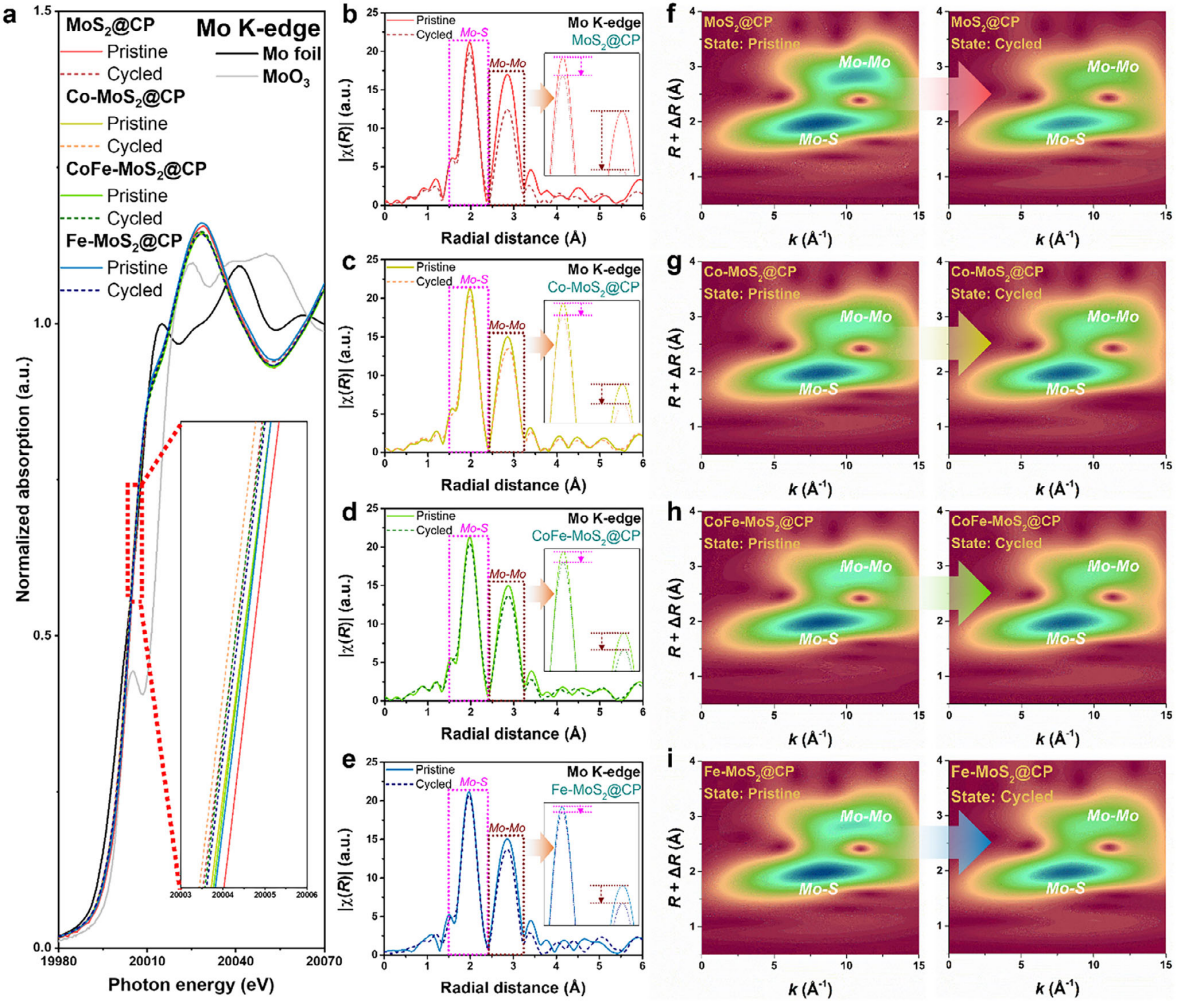
(a) Normalized Mo K‐edge XANES spectra of the MoS_2_@CP, Co‐MoS_2_@CP, CoFe‐MoS_2_@CP, and Fe‐MoS_2_@CP interlayers at both the pristine and cycled states. Corresponding *k* ^3^‐weighted FT‐ and WT‐EXAFS spectra of the (b,f) MoS_2_@CP, (c,g) Co‐MoS_2_@CP, (d,h) CoFe‐MoS_2_@CP, and (e,i) Fe‐MoS_2_@CP interlayers at the pristine and cycled states.

Another factor could be the suppressed local structural disorder, where the catalytic feature can control the chemical interaction with polysulfide reactants, facilitating sustainable redox conversion [11]. For this reason, we conducted ex situ synchrotron‐based X‐ray absorption near‐edge structure (XANES) and extended X‐ray absorption fine structure (EXAFS) analyses of the interlayers under pristine and 100‐cycled at 1C rate condition, which was done to further understand the fundamental effects of dopant states on the electrochemical properties (Figure 6). First, normalized Mo K‐edge XANES spectra of MoS_2_@CP, Co‐MoS_2_@CP, CoFe‐MoS_2_@CP, Fe‐MoS_2_@CP, and Mo reference samples (Mo foil and MoO_3_) are shown (Figure 6a). As shown by the edge absorption positions, which were placed between the Mo foil and MoO_3_ references, the Mo in the prepared catalysts maintains an oxidation state between +0 and +6. Simultaneously, the shift to lower energy after dopant substitution indicates a slight decrease in the Mo oxidation state, which is consistent with the previous XPS data. Notably, after cycling, the edge position of the MoS_2_@CP showed the most drastic energy shift, followed by the Co‐MoS_2_@CP and CoFe‐MoS_2_@CP, while the Fe‐MoS_2_@CP demonstrated a relatively weak shift trend. These results can be explained by the general phenomenon in catalysts where cationic metal centers interact with LiPSs. The adsorption of anion‐rich reactants causes partial electron transfer, which ultimately induces an energy shift with a changed oxidation state [12, 84]. Hence, the most pronounced shift of Mo K‐edge spectra observed in the MoS_2_@CP is attributed to its strongest affinity for the reactants with too much activated metal ions, consistent with the results from the previous adsorption test [85]. It might appear that the relatively strong chemical affinity restricts their efficient conversion, thereby hampering the sulfur species redox reaction during cell cycling, as evidenced via the electrochemical tests above [86]. Also, the corresponding *k* ^3^‐weighted Fourier‐transformed (FT) EXAFS spectra at Mo K‐edge clearly showed characteristic peaks at ca. 1.96 and 2.85 Å for Mo─S and Mo─Mo bonds, respectively (Figure 6b–e). At the pristine state, the substitutional nature of the Co and Fe doping was elucidated by the decreased intensity of Mo‐Mo coordination for the Co‐MoS_2_@CP, CoFe‐MoS_2_@CP, and Fe‐MoS_2_@CP samples compared to that of the MoS_2_@CP, indicating that the TM atoms are doped into the MoS_2_ structure, rather than adsorbed on the surface [87], a finding that is in good agreement with the XPS and Raman characterization. Moreover, the Mo‐S and Mo‐Mo peaks for the MoS_2_@CP interlayer demonstrated significant intensity degradation after the subsequent cycling process, indicative of weakened atomic interactions within the Mo─S and Mo─Mo bond networks (Figure 6b). This suggests a relatively strong interaction with polysulfides on the catalyst surface, leading to a decrease in the long‐range order of the 2H‐MoS_2_ catalyst and inducing a deviation in its electronic structure [11, 88, 89, 90]. The observed structural degradation was effectively mitigated in the Co‐MoS_2_@CP, CoFe‐MoS_2_@CP, and Fe‐MoS_2_@CP interlayers, avoiding the severe shrinkage of Mo‐S and Mo‐Mo peak intensities (Figure 6c–e). While the Fe‐MoS_2_@CP cell exhibited the most pronounced mitigation of peak degradation, the CoFe‐MoS_2_@CP cell displayed a comparable capability in suppressing structural distortion. The curve fitting of the EXAFS data and subsequent parameter tracking revealed that catalysts with relatively stronger adsorption experienced more drastic post‐cycle changes in bond length and significant increases in the Debye–Waller factor, reflecting a major shift in the chemical environment (Figure S56a–d; Table S10). Additionally, the wavelet‐transformed (WT) EXAFS spectra provided further evidence. The TM‐doped samples showed distinctive Mo‐S/Mo‐Mo contour peaks in both *k*‐ and *R*‐spaces even in the cycled state, which contrasts with the spectra obtained for the MoS_2_@CP interlayer (Figure 6f–i). When the Co and Fe doping ratios are differently regulated, a slight edge shift along with more inhibited chemical bond degradation is observed in the sample with a relatively higher Fe content, corroborating the previously identified trends (See Supplementary Note S5). These results further suggest that finely regulated interaction strength with LiPSs can preserve the catalyst structure and guarantee long‐term electrochemical stability.

**FIGURE 7:**
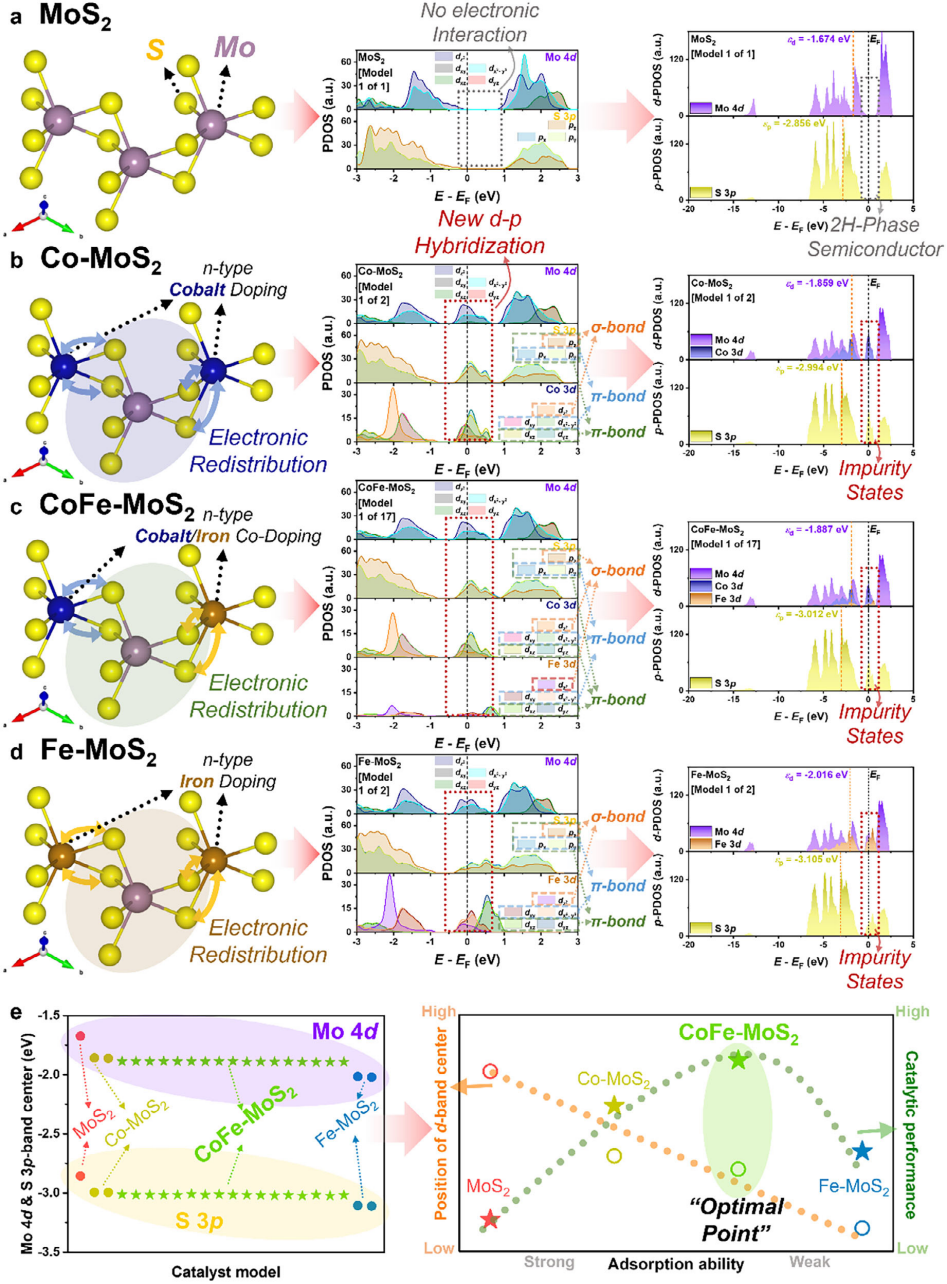
Schematic configuration of S‐mediated bonding states and resultant PDOS spectra, including Mo 4*d*, Co 3*d*, Fe 3*d*, and S 3*p* contributions, for the representative first models of (a) MoS_2_, (b) Co‐MoS_2_, (c) CoFe‐MoS_2_, and (d) Fe‐MoS_2_. (e) Mo 4*d d*‐band and S 3*p p*‐band centers of the various catalyst models, highlighting the optimal balance between adsorption ability and catalytic performance derived from these electronic descriptors.

Significantly, the new *d*‐*p* hybridization accompanied by the *d*‐band shift, could be a fundamental factor for the overall enhanced catalytic activity of the Li‐S cell (Figure 7) [29, 32, 91]. Guided by *d*‐band theory, the Sabatier principle provides the criteria for the optimum catalyst as observed in our Li_2_S_4_ adsorption test. It establishes that catalytic efficiency is maximized at an intermediate binding strength, balancing the kinetics of reactant activation against product desorption, where the mechanism represents the volcano‐type dependence of activity on adsorption energy with the optimal catalytic region [23, 31]. To begin with, if we consider the pristine 2H‐MoS_2_, it is a semiconductor as widely recognized that can be visualized as a central Mo atomic plane sandwiched between two S atomic planes to forming S‐Mo‐S structure, where the layers are characterized by strong intralayer covalent bonding and weak interlayer van der Waals interactions [92]. In this regard, additional theoretical calculations were conducted to analyze the electronic structure of the 2H‐phase catalysts. By altering the specific sites of the Co and Fe dopants, we established a number of computational crystal structure models for DFT calculations, e.g., 1 model for MoS_2_, 2 for Co‐MoS_2_, 17 for CoFe‐MoS_2_, and 2 for Fe‐MoS_2_, as illustrated in Figures S57–S61, respectively. Representatively, the calculated results of the partial density of states (PDOS) corresponding to the first model for each respective catalyst are displayed in Figure 7. The PDOS analysis reveals that while the conduction band (CB) and valence bands (VB) of pristine 2H‐MoS_2_ are derived from an interaction between Mo 4*d* and S 3*p* orbitals, there is no significant orbital interaction near the *E*_F_, exhibiting its semiconducting status with a band gap of approximately 1.18 eV. Also, we can observe that Mo 4*d d*‐band and S 3*p p*‐band centers are located at −1.674 and −2.856 eV, respectively (Figure 7a). In contrast, the introduction of Co and Fe dopants initiates a new S‐mediated *d*‐*p* hybridization process involving TM 3*d* and S 3*p* orbitals, which leads to electronic redistribution, thereby endowing the MoS_2_ catalyst with distinct electronic and physicochemical properties [93, 94]. To be specific, considering the geometric arrangement of the orbitals, the TM 3*d*_z2_ orbital can form a *σ*‐bond with the S 3*p*_z_ orbital through head‐on interaction, and *π*‐bonds could be formed via the side‐on interaction of S 3*p*_x_/*p*_y_ orbitals with the TM 3*d*_xy_/*d*_x2‐y2_ orbitals, as well as of S 3*p*_x_/*p*_y_/*p*_z_ orbitals with the TM 3*d*_xz_/*d*_yz_ orbitals (Figure S62). This orbital hybridization introduces new localized electronic states within the Mo 4*d—*S 3*p—*TM 3*d* bonds to induce the impurity energy bands near the *E*_F_ (Figure 7b–d; Figures S63–S66). A minor factor might be the wave function resonance between TM dopant *p*‐orbitals and Mo *d*‐orbitals, which also contributes to the gap states proximate to the CB (Figures S67–S72) [95]. Since the transport processes are governed by electrons near the *E*_F_, a higher density of electronic states with more valence electrons at *E*_F_ boosts electron transfer, contributing to enhanced electronic conductivity [96]. This suggests that proper TM doping can influence directly on accelerated charge transfer kinetics by changing the intrinsic electronic conductivity of 2H‐MoS_2_ catalyst, which is corroborated by the THz‐TDS analysis [96, 97]. Furthermore, it is well known that such *d*‐*p* hybridization can enhance the charge carrier mobility of the inactive basal plane of 2H‐MoS_2_, thereby increasing the number of active sites on the catalyst [26, 98]. Therefore, the electronic redistribution can facilitate effectively the conversion kinetics, ultimately leading to high Li‐S cell performance [87, 96].

Meanwhile, taking into account the electronic configurations (Mo: 4*d* ^5^5*s* ^1^, Co: 3*d* ^7^4*s* ^2^, Fe: 3*d* ^6^4*s* ^2^), the valence electron‐rich Co^2+/3+^ and Fe^2+/3+^ are widely known to inject extra electrons, which enhances lattice filling and broadens the *d*‐orbital electron clouds [8]. The generation of extra electron energy levels near *E*_F_ by these n‐type dopants shifts the electronic profile closer to the CB [95]. Consequently, the *d*‐band energy levels undergo a relative shift, resulting in a lowered *d*‐band center, well consistent with the obtained PDOS in this study. Consequently, the *d*‐band center shows a decreasing trend in its energy: MoS_2_ (−1.674 eV) > Co‐MoS_2_ (−1.859 eV) > CoFe‐MoS_2_ (−1.887 eV) > Fe‐MoS_2_ (−2.016 eV). Moreover, the *d*‐band center values derived from the other structural models with varying dopant configurations consistently display a similar trend (Figures S73–S75). The reason why Fe doping makes the *d*‐band center relatively more negative than Co doping can be attributed to the difference in orbital interaction between the TM 3*d*‐orbital and the S 3*p*‐orbital. Specifically, substantial overlap between the *d*‐orbitals of metal atoms and the *p*‐orbitals of adjacent non‐metal atoms induces strong orbital interaction, which significantly modulates the electronic structure, leading to a pronounced downward shift of the *d*‐band center [99, 100]. Since *σ*‐bonds contribute more considerably to structural stability than *π*‐bonds among the various orbital interactions, we can quantitatively compare the TM 3*d* and S 3*p* orbital overlap obtained from Co‐MoS_2_ and Fe‐MoS_2_ (Figure S76a–c). The relative surface area values of the TM 3*d*_z2_‐S 3*p*_z_ overlap for Co‐MoS_2_ (Model 1: 19.65 / Model 2: 19.65) were lower than those for Fe‐MoS_2_ (Model 1: 19.83 / Model 2: 19.82). Moreover, the PDOS spectrum of Fe‐MoS_2_ was observed to be distributed toward negative energy values, and a related reason to this phenomenon might be a relatively large magnetic exchange splitting of Fe, whose deeper majority‐spin 3*d* states more strongly stabilize the bridging S 3*p* orbitals and transmit this stabilization to Mo through the *d*‐*p*‐*d* coupling, thereby may induce an additional downward shift of the *d*‐band center [101, 102]. As for the minor contributions, although the Co 3*d*_xz/_ *d*_yz_‐S 3*p*_x/_ *p*_y/_ *p*_z_ overlap (Model 1: 15.78, 15.78 / Model 2:19.65, 19.66) was slightly larger than that of Fe‐MoS_2_ (Model 1:17.76, 17.76 / Model 2: 16.05, 16.05), this was offset by the Fe 3*d*_xy/_ *d*_x2‐y2_‐S 3*p*_x/_ *p*_y_ overlap (Model 1:17.72, 17.72 / Model 2: 19.36, 19.23), which exceeded that of Co‐MoS_2_ (Model 1:16.84, 16.84 / Model 2: 18.96, 19.03). Based on these trends, it can be predicted that Fe‐MoS_2_ shows a relatively more effective TM 3*d*‐S 3*p* overlap than that of Co‐MoS_2_, leading to the downshift of the *d*‐band center. Similarly, the *p*‐band center values also decreased across the series: MoS_2_ (−2.856 eV) > Co‐MoS_2_ (−2.994 eV) > CoFe‐MoS_2_ (−3.012 eV) > Fe‐MoS_2_ (−3.105 eV). Regardless of the specific dopant position, the calculated *p*‐band center values from the remaining models also exhibit a consistent trend, paralleling the behavior of the *d*‐band center. As a result, the electronic distribution overlap between *d*‐PDOS and *p*‐PDOS has increased when considering the difference between *d*‐ and *p*‐band center values, which signifies a conductivity increase in the catalyst due to doping, thereby improving the ion exchange properties of the resulting catalyst [24, 103]. Although the increase in conductivity driven by TM dopants and electronic overlap in 2H‐MoS_2_ facilitates catalytic performance, the data suggest that a unidirectional pursuit of high conductivity is insufficient. More importantly, a strategy that considers reactant interactions to find an optimal doping point is required to get dual advantages by simultaneously enhancing both conductivity and catalytic activity. Therefore, our findings reveal a volcano‐type correlation, which means that maximizing cell performance requires adjusting the *d*‐band center to an intermediate value, thereby avoiding the detrimental effects of extreme high or low energy levels (Figure 7e). Furthermore, these results remain consistent even when adjusting the Co/Fe dopant ratio. Overall, upon conducting a three‐way comparison defining the x‐axis as the 1C final capacity (retention), the y‐axis as the *d*‐band center (adsorption capability), and the z‐axis as the 0.1C–10C discharge capacity ratio (fast charge‐discharge ability), it is clear that the CoFe‐MoS_2_@CP (Co:Fe = 4:1) sample, maintaining a moderate *d*‐band center, exhibits the most superior electrochemical performance, marking the optimal point (See Supplementary Note S6).

**FIGURE 8:**
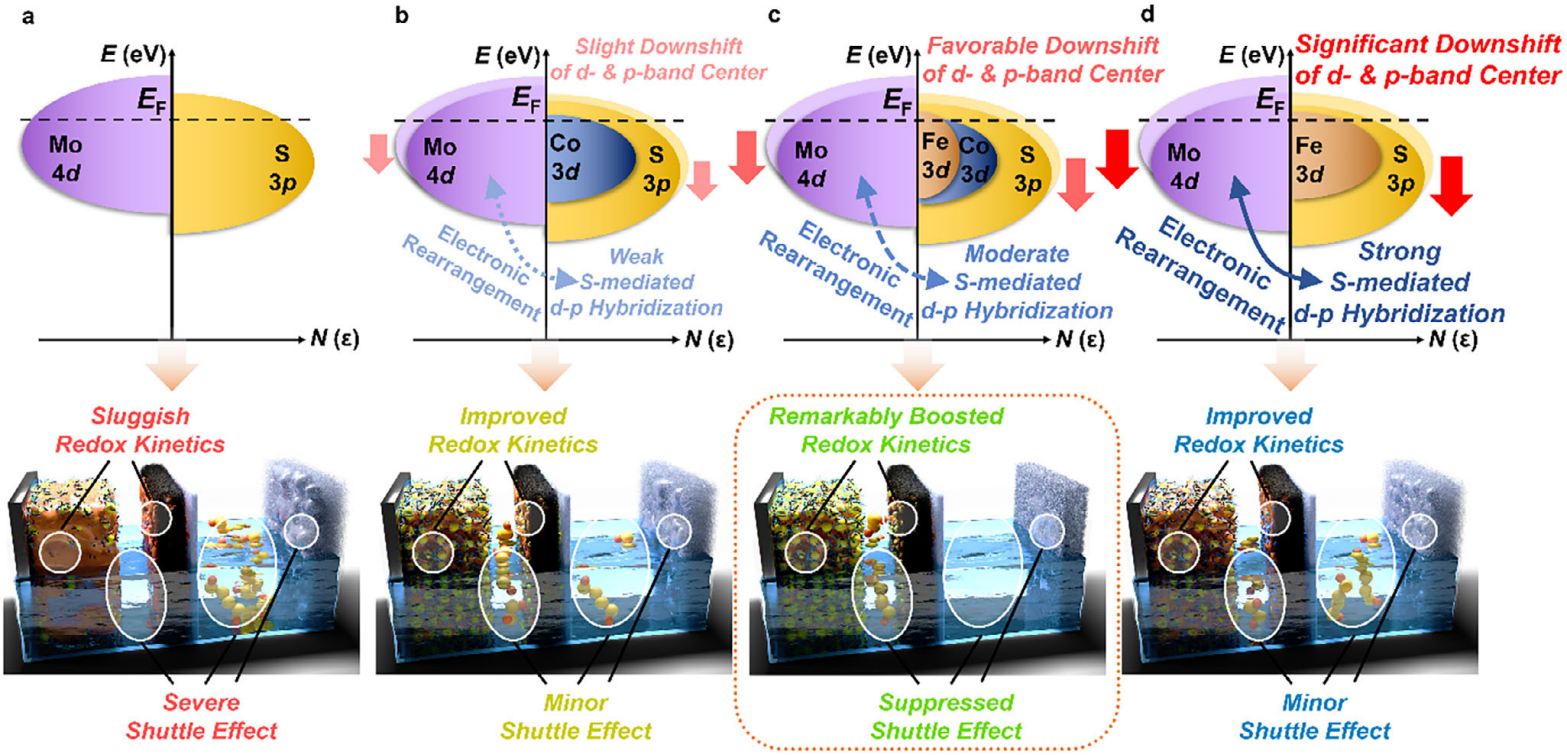
Visual study summary with a conceptual DOS diagram, illustrating S‐mediated *d*‐*p* hybridization via electronic rearrangement upon TM dopant introduction, and band center shifts leading to distinct sulfur redox kinetics and shuttle effects during the operation of (a) MoS_2_, (b) Co‐MoS_2_, (c) CoFe‐MoS_2_, and (d) Fe‐MoS_2_ cells.

Drawing upon the experimental results and theoretical calculations across all catalyst models, this study provides an elucidation of how electronic structures and adsorption abilities of metals influence the catalytic performance of the Li‐S cell (Figure 8; Figure S77). Pristine 2H‐MoS_2_, which possesses only Mo 4*d—*S 3*p* orbital interactions, fails to induce a markedly low *E*_a_ barrier for the conversion of LiPSs to Li_2_S. This leads to sluggish sulfur redox kinetics and causes a severe shuttle effect, thereby deteriorating the long‐term stability of the Li‐S cell (Figure 8a). However, substitution with n‐type TM dopants like Co or Fe induces new S‐mediated *d*‐*p* hybridization, affecting the Mo 4*d*‐orbitals and triggering a rearrangement of the electronic structure of the catalysts (Figure 8b,d). Nevertheless, the single TM doped Co‐MoS_2_ or Fe‐MoS_2_ induces relatively weak or strong hybridization, respectively, both failing to tune the Mo 4*d d*‐band center to the optimal point. Consequently, although sulfur redox kinetics are improved, the problem of ineffectively suppressing unwanted sulfur migration is not able to be resolved. Therefore, by employing the co‐doping strategy, effective *d*‐*p* hybridization can be modulated (Figure 8c). This optimizes the interaction between the catalyst surface and reactants, inducing superior reaction kinetics while effectively suppressing the shuttle effect, thus enabling to remarkably enhance the Li‐S cell performance.

## 3 Conclusion

In summary, we have successfully developed a highly efficient catalytic interlayer for high‐performance Li‐S batteries by engineering the electronic structure of 2H‐MoS_2_ via a co‐doping strategy with Co and Fe dopants. Our experimental and theoretical investigations thoroughly elucidate that the substitution of Mo sites with n‐type TM dopants induces a distinct S‐mediated *d*‐*p* hybridization, which effectively modulates the electronic profiles. Specifically, the synergistic effect of Co and Fe co‐doping optimizes the downward shift of the Mo 4*d d*‐band center to an intermediate and appropriate energy level. This electronic reconfiguration balances the binding affinity between the catalyst surface and polysulfide species, thereby overcoming the trade‐off between adsorption strength and desorption kinetics. The catalyst interlayer can avoid unnecessarily high interactions by establishing an optimal *d*‐band center, which facilitates diffusion‐favorable Li_2_S nucleation, increasing re‐participation of sulfur species and minimizing their dissolution. The resultant relationship between the energy position of *d*‐band center and their catalytic performance in the Li‐S cells follows a definite volcano trend, emphasizing that neither too high nor too low *d*‐band center of the catalyst is desirable. Furthermore, the electronic redistribution induced by TM doping causes a downshift of the *p*‐band center, resulting in an increased overlap ratio between *d*‐PDOS and *p*‐PDOS compared to the pristine 2H‐MoS_2_, thereby enhancing both electronic and ionic conductivity. Consequently, the CoFe‐MoS_2_@CP catalyst interlayer not only facilitates rapid redox reaction kinetics with minimized activation energy but also effectively suppresses the shuttle effect and ensures the uniform deposition of Li_2_S during cell operation. These atomistic advantages can explain the exceptional electrochemical performance, including remarkable capacity retention of 43.1% and 47.5% with decay rates of only 0.028% and 0.024% over more than 2000 cycles at high rates of 5C and even at 10C, respectively. Moreover, under a high sulfur loading (6.2 mg cm^−2^) and a low E/S ratio (9.7 µL mg^−1^), the CoFe‐MoS_2_@CP cell delivers a high areal capacity of 5.15 mAh cm^−2^ at 0.1C, maintaining 76.4% retention even after 200 cycles with a decay rate of only 0.118% per cycle. Furthermore, the practical viability of this approach is validated through a stable Li‐S full‐cell operation (sulfur loading: 6.8 mg cm^−2^, E/S: 8.8 µL mg^−1^, N/P: 2.2), yielding an initial areal capacity of 6.22 mAh cm^−2^ and a final capacity of 4.21 mAh cm^−2^ after 100 cycles. We believe this targeted *d*‐band regulation approach could provide a comprehensive mechanistic understanding of how orbital engineering drives macroscopic performance, which establishes a pioneering design guideline for advanced chalcogenide catalysts, essential for achieving practical high‐performance Li‐S batteries.

## 4 Experimental Section

Detailed experimental procedures can be found in the Supporting Information.

## Conflicts of Interest

The authors declare no conflicts of interest.

## Supporting information




**Supporting File**: adma73445‐sup‐0001‐SuppMat.pdf.

## Data Availability

The data that support the findings of this study are available from the corresponding author upon reasonable request.
