# Supplementary material for: Targeted Modulation of d‐Band Center in MoS2 Interlayer With n‐Type Co/Fe Dopants Accelerating Sulfur Reaction Kinetics in Lithium‐Sulfur Batteries
Source: Adv Mater. 2026 May 21;38(36):e73445. doi: 10.1002/adma.73445 (PMC13310115; doi:10.1002/adma.73445)
Supplement: Supplementary file 1 — Supporting File: adma73445‐sup‐0001‐SuppMat.pdf. [file ADMA-38-e73445-s001.pdf]

Supporting Information

Targeted Modulation of *d*-band Center in MoS_2 Interlayer with n-type Co/Fe Dopants Accelerating Sulfur Reaction Kinetics in Lithium-Sulfur Batteries

Junhyuk Ji, Sangyeon Won, Jaehyeong Yu, Nuri Moon, Dongwoo Kim, Junbeom Maeng, Won Bae Kim*

J. Ji, J. Maeng, Prof. W. B. Kim
Department of Chemical Engineering, Pohang University of Science and Technology (POSTECH), 77 Cheongam-ro, Nam-gu, Pohang-si, Gyeongsangbuk-do 37673, Republic of Korea

S. Won, J. Yu, N. Moon, D. Kim, Prof. W. B. Kim
Department of Battery Engineering, Graduate Institute of Ferrous and Eco Materials Technology (GIFT), Pohang University of Science and Technology (POSTECH), 77 Cheongam-ro, Nam-gu, Pohang-si, Gyeongsangbuk-do 37673, Republic of Korea

E-mail: kimwb@postech.ac.kr (W. B. Kim)

Keywords: Li-S batteries, MoS_2 catalyst, *d*-band center, n-type dopant, *d*-*p* hybridization, Interlayer

Contents

Experimental section ---------- 5

Chemicals ---------- 5

Synthesis of Co, Fe co-doped MoS_2 grown directly on a carbon paper substrate ---------- 5

Material characterization ---------- 5

Electrochemical measurements of cathode catalyst materials ---------- 6

Evaluation of Li_2S_4 Adsorption Capability ---------- 7

Theoretical calculations ---------- 7

Supplementary Note 1 ---------- 9

Preparation and characterization of S/MWCNT composite cathode ---------- 9

Supplementary Note 2 ---------- 10

Scan rate dependent electrochemical behavior and kinetic analysis ---------- 10

Supplementary Note 3 ---------- 11

Design and optimization workflow for the catalyst ---------- 11

Supplementary Note 4 ---------- 31

Analysis of Li_2S nucleation and growth mechanisms ---------- 31

Supplementary Figure ---------- 33

Synthesis and physicochemical analysis of Co and Fe co-doped MoS_2 ---------- 33

Figure S1 (Photos of Prepared Interlayers) ---------- 33

Figure S2 (ICP-OES Calibration Linear Fit) ---------- 33

Figure S3 (TEM and EELS Elemental Mapping) ---------- 34

Figure S4 (SAED Pattern and HR-TEM) ---------- 34

Figure S5 (SAED Patterns of Catalysts) ---------- 35

Figure S6 (SEM Images and EDS Mapping) ---------- 35

Figure S7 (HRPD Patterns of Catalyst Powders) ---------- 36

Figure S8 (C 1*s* Region XPS Spectra) ---------- 36

Figure S9 (Electrical Conductivity Measurements) ---------- 37

Figure S10 (BET Isotherms and Pore Distributions) ---------- 37

Catalytic gains of the fabricated interlayers for high electrochemical performance ---------- 38

Figure S11 (Li_2S_4 Adsorption UV-vis Quantification) ---------- 38

Figure S12 (Post-mortem XPS Spectra Analysis) ---------- 38

Figure S13 (Symmetric Cell Tafel and EIS) ---------- 39

Figure S14 (Symmetric Cell CV Curves) ---------- 39

Figure S15 (Symmetric Cell CV Curves) ---------- 39

Figure S16 (Asymmetric Cell CV Curves) ---------- 40

Figure S17 (Asymmetric Cell CV Curves) ---------- 40

Figure S18 (Peak Current Linear Fit) ---------- 41

Figure S19 (Log-Log Slope Analysis Plot) ---------- 41
Figure S20 (Kinetics Analysis of MoS_2 Cell) ---------- 42
Figure S21 (Kinetics Analysis of Co-MoS_2 Cell) ---------- 42
Figure S22 (Kinetics Analysis of CoFe-MoS_2 Cell) ---------- 43
Figure S23 (Kinetics Analysis of Fe-MoS_2 Cell) ---------- 43
Figure S24 (Surface Contribution Ratio Comparison) ---------- 44
Electrochemical performance of Li-S cells equipped with the designed interlayers ---------- 44
Figure S25 (Rate Capability of Bare CP) ---------- 44
Figure S26 (Charging Rate Capability Performance) ---------- 44
Figure S27 (GCD Profiles at Various Rates) ---------- 45
Figure S28 (GCD Profiles During Rate Test) ---------- 45
Figure S29 (Discharge Plateau Capacity Ratios) ---------- 46
Figure S30 (Enlarged GCD Profile Views 1) ---------- 47
Figure S31 (Enlarged GCD Profile Views 2) ---------- 48
Figure S32 (Overall Rate Performance Comparison) ---------- 48
Figure S33 (Cycling Performance of Bare CP) ---------- 49
Figure S34 (Individual Cycle GCD Profiles) ---------- 49
Figure S35 (Comparative Cycle GCD Profiles) ---------- 50
Figure S36 (EIS Before and After Cycling) ---------- 50
Figure S37 (Post-mortem Li Anode SEM) ---------- 51
Figure S38 (Cycling Plateau Capacity Ratios) ---------- 52
Figure S39 (Enlarged Cycling GCD Profiles) ---------- 52
Figure S40 (1 C Cycling Performance Comparison) ---------- 53
Figure S41 (5 C Activation Cycle Process) ---------- 53
Figure S42 (5 C Cycling GCD Profiles) ---------- 54
Figure S43 (5 C Comparative GCD Profiles) ---------- 54
Figure S44 (5 C Cycling Performance Comparison) ---------- 55
Figure S45 (10 C Activation Cycle Process) ---------- 55
Figure S46 (10 C Cycling GCD Profiles) ---------- 56
Figure S47 (10 C Cycling Performance Comparison) ---------- 56
Figure S48 (High Loading Cycling Performance) ---------- 57
Figure S49 (Li Metal Deposition Profile) ---------- 57
Figure S50 (Li-S Full-Cell Cycling Test) ---------- 58
Discussion on underlying mechanisms for enhanced electrochemical performance ---------- 58
Figure S51 (In-situ EIS at Various Temps) ---------- 58
Figure S52 (Ex-situ SEM of Li_2S Nuclei) ---------- 59
Figure S53 (Statistical Diameters of Li_2S Nuclei) ---------- 60
Figure S54 (Polysulfide Shuttle Current Measurement) ---------- 61
Figure S55 (Li_2S Dissolution Potential Curves) ---------- 61
Figure S56 (EXAFS fitting results) ---------- 62

Supplementary Note 5 ---------- 63

Supplementary XAFS analysis of all Co/Fe co-doped MoS_2 cathode catalysts ---------- 63

Figure S57 (MoS_2 Crystal Structure Model) ---------- 64
Figure S58 (Co-MoS_2 Crystal Structure Model) ---------- 64
Figure S59 (CoFe-MoS_2 Crystal Structure Model) ---------- 65
Figure S60 (CoFe-MoS_2 Crystal Structure Model) ---------- 66
Figure S61 (Fe-MoS_2 Crystal Structure Model) ---------- 67
Figure S62 (Orbital Bonding Geometry Schematics) ---------- 67
Figure S63 (PDOS of Co-MoS_2 Model) ---------- 68
Figure S64 (PDOS of CoFe-MoS_2 Model 1) ---------- 69
Figure S65 (PDOS of CoFe-MoS_2 Model 2) ---------- 70
Figure S66 (PDOS of Fe-MoS_2 Model) ---------- 71
Figure S67 (PDOS of Pristine MoS_2) ---------- 72
Figure S68 (Co 4*p* Orbital PDOS Spectra) ---------- 72
Figure S69 (TM 4*p* Orbital PDOS Spectra) ---------- 73
Figure S70 (TM 4*p* Orbital PDOS Spectra) ---------- 74
Figure S71 (TM 4*p* Orbital PDOS Spectra) ---------- 75
Figure S72 (Fe 4*p* Orbital PDOS Spectra) ---------- 75
Figure S73 (*d*-Orbital PDOS of Co-MoS_2) ---------- 76
Figure S74 (*d*-Orbital PDOS of CoFe-MoS_2) ---------- 76
Figure S75 (*d*-Orbital PDOS of Fe-MoS_2) ---------- 77

Supplementary Note 6 ---------- 78

Design and optimization workflow for the Co2.5 Fe2.5-/Co1 Fe4-MoS_2 catalyst models ---------- 78

Figure S76 (Orbital Overlap Analysis Spectra) ---------- 94
Figure S77 (Calculated *d*-band Center Values) ---------- 94

Supplementary Table ---------- 95

Table S1 (ICP-OES Elemental Composition Results) ---------- 95
Table S2 (Composite Structural Properties (BET/Pore) ---------- 96
Table S3 (EIS Parameters Before/After Cycling) ---------- 97
Table S4 (Performance Comparison with References) ---------- 98
Table S5 (In-situ EIS Parameters (45 ºC) ---------- 100
Table S6 (In-situ EIS Parameters (55 ºC) ---------- 101
Table S7 (In-situ EIS Parameters (65 ºC) ---------- 102
Table S8 (Calculated Activation Energy Values) ---------- 103
Table S9 (Li_2S Nucleation Growth Parameters) ---------- 104
Table S10 (Mo K-edge EXAFS Fitting) ---------- 105

Supporting Information References ---------- 106

Experimental section

Chemicals

Molybdenum(V) chloride ($MoCl_5$, 99.6%), and thiourea (CH_4N_2S, 99%) were purchased from Alfa Aesar^{TM}. Cobalt(II) chloride hexahydrate ($CoCl_2 \cdot 6H_2O$, ReagentPlus, 98%), iron(II) chloride tetrahydrate ($FeCl_2 \cdot 4H_2O$, ReagentPlus, 98%), and absolute ethanol (C_2H_6O, ACS reagent, ≥ 99.5%) were purchased from Sigma-Aldrich. Carbon paper (AvCarb P50T) was purchased from AvCarb Material Solutions.

Synthesis of Co, Fe co-doped MoS_2 grown directly on a carbon paper substrate

For the synthesis of the Co, Fe co-doped MoS_2 interlayer, 0.9 mmol of molybdenum chloride, 3.0 mmol of thiourea, 3.8×10^{-2} mmol of cobalt chloride hexahydrate, and 9.5×10^{-3} mmol of iron chloride tetrahydrate were first added to 3 mL of absolute ethanol. The resulting solution was then magnetically stirred for 2 h at room temperature. Subsequently, a total of 200 μL of the as-prepared metal precursor solution was drop-casted onto a carbon paper (CP) disk (19 mm in diameter) placed on a hot plate at 100 °C. Finally, the sample was annealed in a tube furnace at 400 °C for 1 h under Ar atmosphere to fabricate the interlayer. To synthesize MoS_2 materials with different doping percentages, the molar ratio of cobalt chloride hexahydrate to iron chloride tetrahydrate was adjusted while maintaining their total molar amount constant. Hereinafter, the CP composites loaded with MoS_2, Co-doped MoS_2, Co and Fe co-doped MoS_2, and Fe-doped MoS_2 are referred to as MoS_2@CP, Co-MoS_2@CP, CoFe-MoS_2@CP, and Fe-MoS_2@CP, respectively.

Material characterization

Microstructure of the material was characterized by scanning electron microscopy (SEM; JSM-7800F Prime, JEOL) at 5.0 kV with EDS, and by transmission electron microscopy (TEM; JEM-2200FS, JEOL) at 200 kV with EELS. Metal atomic ratios were determined by inductively coupled plasma-optical emission spectroscopy (ICP-OES, iCAP 7400; Thermo Fisher Scientific). Crystal structure analysis was performed using X-ray diffraction (XRD; Ultima IV, Rigaku) with a Ni-filtered Cu-Kα source (λ = 1.5418 Å, 40 kV, 30 mA), and supplemented by synchrotron high-resolution XRD at the Pohang Light Source (PLS-II) 9B HRPD beamline (λ = 1.54610 Å). X-ray photoelectron spectroscopy (XPS; K-Alpha+, Thermo Fisher Scientific) with a monochromatic Al-Kα source (1486.6 eV) was employed to probe the surface electronic states. X-ray absorption fine structure (XAFS) measurements were conducted at the 7D XAFS and 10C Wide XAFS beamlines of PLS-II. Thermogravimetric analysis (TGA; SDT Q600, TA

instruments) was run under Ar atmosphere. N_2 adsorption–desorption analysis (ASAP 2020; Micromeritics) was utilized to determine the specific surface area and pore diameters via the Barrett-Emmett-Teller (BET) method. Fourier-transform infrared (FT-IR) absorbance spectra were acquired with a Spectrum Two (PerkinElmer) instrument, and Raman spectra were collected using an NRS-5100 spectrometer (JASCO) with a 532 nm laser. Electrical conductivity was measured using a powder resistance meter (HPRM-FA2; ZH Instrument).

Electrochemical measurements of cathode catalyst materials

The sulfur cathode was prepared through a multi-step process. The active material was first synthesized via a melt-infusion technique, wherein a mixture of elemental sulfur (S_8, 80 wt.%) and multiwalled carbon nanotubes (MWCNTs, 20 wt.%) was heat-treated at 155 °C for 12 h. A homogeneous cathode slurry was then formulated by dispersing the resultant S_8/MWCNT composite (80 wt.%), carbon black as a conductive additive (10 wt.%), and poly(vinylidene fluoride-co-hexafluoropropylene) (PVDF-co-HFP) as a polymeric binder (10 wt.%) in N-methyl-2-pyrrolidone (NMP) using a Thinky mixer. The final electrode was fabricated by doctor-blading the slurry onto a carbon-coated Al foil current collector, followed by an overnight drying step in a vacuum oven at 60 °C. Electrochemical performance was evaluated using CR2032-type coin cells assembled in an argon-filled glove box. Cathodes with an areal sulfur loading of approximately 1.0 mg cm^{-2} or 6.2 mg cm^{-2} were used for half-cell tests, whereas a fixed loading of 6.8 mg cm^{-2} was used for full-cell operations. All electrodes had a geometric diameter of 12 mm. The electrolyte consisted of 1.0 M LiTFSI in a 1:1 (v/v) mixture of DOL and DME, with 2 wt.% $LiNO_3$ as an additive. The electrolyte-to-sulfur (E/S) ratio was maintained at 30 μL mg^{-1} for most tests but was adjusted to 9.7 and 8.8 μL mg^{-1} for high-loading and full-cell tests, respectively. All tests were conducted at a strictly controlled temperature of 25 °C using a Won-A Tech WBCS 3000 battery testing system. Galvanostatic charge-discharge (GCD) cycling was performed within a voltage window of 1.7–2.8 V (vs. Li/Li^+). To activate the Li-S cells prior to the long-term cycling tests at 5 C, they were pre-cycled at 0.1, 0.2, 0.5, 1, 2, 3, and 4 C for 1, 1, 2, 3, 4, 5, and 5 cycles, respectively. Similarly, for activation before the 10 C long-term cycling test, the cells were pre-cycled at 0.1, 0.2, 0.5, 1, 2, 3, 4, 5, 6, 7, 8, and 9 C for 1, 1, 2, 3, 4, 5, 5, 5, 5, 5, 5, and 5 cycles, respectively. Cyclic voltammetry (CV) was conducted in the same voltage range at scan rates varying from 0.03 to 0.20 mV s^{-1}. The polysulfide redox kinetics were probed via cyclic voltammetry on symmetric cells constructed with identical electrodes (MoS_2@CP, Co-MoS_2@CP, CoFe-MoS_2@CP, and Fe-MoS_2@CP) in a 0.5 M Li_2S_6 catholyte over a -1.5 to 1.5 V (vs. Li/Li^+) window. Furthermore, the Li metal

anode stabilization effect of the interlayers was assessed through long-term galvanostatic cycling of Li//Li symmetric cells at 1 mA cm^{-2} (1 mAh cm^{-2} per half-cycle) for 1,000 h. The galvanostatic intermittent titration technique (GITT) was employed by applying a 0.1 C current pulse for 10 min followed by a 120 min rest period. Polarization was calculated from GITT data using the formula: $i\Delta R$ (Ω) = $|\Delta V_{QOCV\text{-}CCV}|$, where i is the applied current, ΔR is the internal resistance, and ΔV is the voltage difference between the points of quasi-OCV and closed-circuit voltage. For Li_2S nucleation analysis, chronoamperometry was performed at 2.05 V (vs. Li/Li$^+$) for 200 ks. For Li_2S dissolution analysis, cells were discharged to 1.7 V (vs. Li/Li$^+$) at 0.05 C, followed by chronoamperometry at 2.35 V (vs. Li/Li$^+$) for 100 ks. Ex-situ electrochemical impedance spectroscopy (EIS) was measured with a ZIVELAB potentiostat over a frequency range of 100 kHz to 10 mHz with a 5 mV amplitude. In-situ EIS was conducted under the same conditions as the ex-situ measurements, with the exception that tests were performed at three distinct temperatures: 45, 55, and 65 °C. Concurrently, the cell was galvanostatically discharged from 2.8 V to 1.7 V (vs. Li/Li$^+$) at a rate of 0.1 C, and an EIS spectrum was collected at 0.1 V intervals. Shuttle currents were measured as following: the cells after formation step were first galvanostatically charged to 2.90 V (vs. Li/Li$^+$) at 0.05 C, and discharged sequentially to 2.80, 2.60, 2.45, 2.43, 2.40, 2.39, 2.38, 2.37, 2.36, 2.35, 2.34, 2.25, and 2.20 V (vs. Li/Li$^+$) at 0.05 C with applying 10 h potentiostatic holds at each voltage level. Synchrotron-based operando XRD was performed at the Pohang Light Source (PLS-II) to monitor in-situ cathode variations; data were collected in transmission mode at the 1D XRS KIST-PAL and 6D UNIST-PAL beamlines using X-ray energies of 12.3984 and 18.986 keV, respectively.

Evaluation of Li_2S_4 Adsorption Capability

To investigate the chemical affinity for lithium polysulfides, a static adsorption experiment was designed. First, an analyte solution of 1 mM Li_2S_4 was formulated from lithium sulfide and sulfur (8:3 molar ratio) in DOL/DME (1:1, v/v) at 90 °C. Next, to induce adsorption, 50 mg of each prepared catalyst (MoS_2@CP, Co-MoS_2@CP, CoFe-MoS_2@CP, and Fe-MoS_2@CP) was individually submerged in the Li_2S_4 solution for several days. To quantify the uptake, the remaining concentration of Li_2S_4 in the solution was determined by transferring the supernatant to a UV-cuvette and measuring its UV-visible absorption spectrum on an Agilent Cary 8454 spectrometer.

Theoretical calculations

First-principles calculations were performed using the Quantum Espresso software package. The interaction between ionic cores and valence electrons was described by the projector augmented wave (PAW) method. The exchange-correlation energy was modeled using the Perdew-Burke-Ernzerhof (PBE) functional within the generalized gradient approximation (GGA). Van der Waals (vdW) interactions were included via Grimme's DFT-D3 correction. A plane-wave energy cutoff of 40 Ry was set for all calculations. The geometry was optimized by relaxing the forces on all atoms to a threshold of 0.002 Ry $bohr^{-1}$. The Brillouin zone was sampled using a 3 × 3 × 3 Monkhorst-Pack k-point mesh. To explore various possible dopant configurations, we constructed a library of doped MoS_2 models, which were based on the substitution of five Mo atoms per layer with Co and/or Fe atoms to account for different relative positions, yielding 2 models for singly-doped Co-MoS_2, 17 for co-doped CoFe-MoS_2, and 2 for singly-doped Fe-MoS_2, all of which were subjected to calculation. The *d*-band center (ε_d) is calculated as the weighted average energy of the *d*-electron states relative to the Fermi level (E_F) using the following formula:

$$\varepsilon_d = \frac{\int \varepsilon \cdot n_d(\varepsilon)\, d\varepsilon}{\int n_d(\varepsilon)\, d\varepsilon}$$

where ε is the electronic energy of states, and $n_d(\varepsilon)$ is the electronic energy of states. The integration was conducted over all energy ranges, and the convergence condition for the energy is less than 8 × 10^{-6} Ry per atom.

Supplementary Note 1

Preparation and characterization of S/MWCNT composite cathode

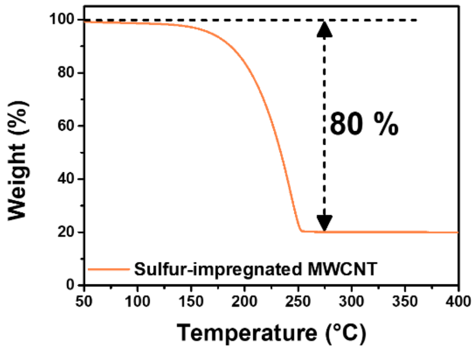

Supplementary Note Figure N1. TGA curve of the MWCNT cathode composite containing melt-diffused sulfur, obtained under an argon atmosphere.

In the typical asymmetrical Li-S cell assembled with Li metal, cathode, separator, and interlayer, a cathode fabricated by blending sulfur-impregnated MWCNTs with a binder and conductive agent was used. The thermal gravimetric analysis (TGA) result confirmed that the melt-diffused sulfur content in the cathode was approximately 80 wt.% as explained in Experimental section (Supplementary Note Figure N1).

Supplementary Note 2

Scan rate dependent electrochemical behavior and kinetic analysis

The contributions from surface and diffusion-controlled processes can be qualitatively analyzed using the following relationships:

$$\log i = \mathrm{a} + \mathrm{b} \log v \tag{S1}$$

$$i = k_1 v + k_2 v^{1/2} \tag{S2}$$

, where i denotes the current at a particular scan rate v, a and b are constants obtained from the intercept and the slope of the linear fit plot of log(v) vs. log(i). Generally, when the b value is close to 0.5, it indicates the electrochemical system is diffusion-controlled, while when approaches to 1, it reveals the process is totally surface-controlled. In this regard, the contributions of surface-controlled process ($k_1 v$) and diffusion-controlled electrochemical reaction ($k_2 v^{1/2}$) could be quantitatively determined.

The Li^+ diffusion coefficient (D_{Li^+}) for ion mobility could be calculated by using the following Randles-Sevick equation:

$$i_p = 2.69 \times 10^5 n^{1.5} A D_{Li+}^{0.5} C_{Li+} v^{0.5} \tag{S3}$$

, where i_p represents the peak current, n is the number of electrons transferred, A is the electrode area (cm^2), D_{Li^+} is the Li^+ diffusion coefficient ($cm^2\ s^{-1}$), C_{Li^+} is the Li^+ concentration in the electrolyte (mM), and v is the scan rate ($V\ s^{-1}$).

Supplementary Note 3

Design and optimization workflow for the catalyst

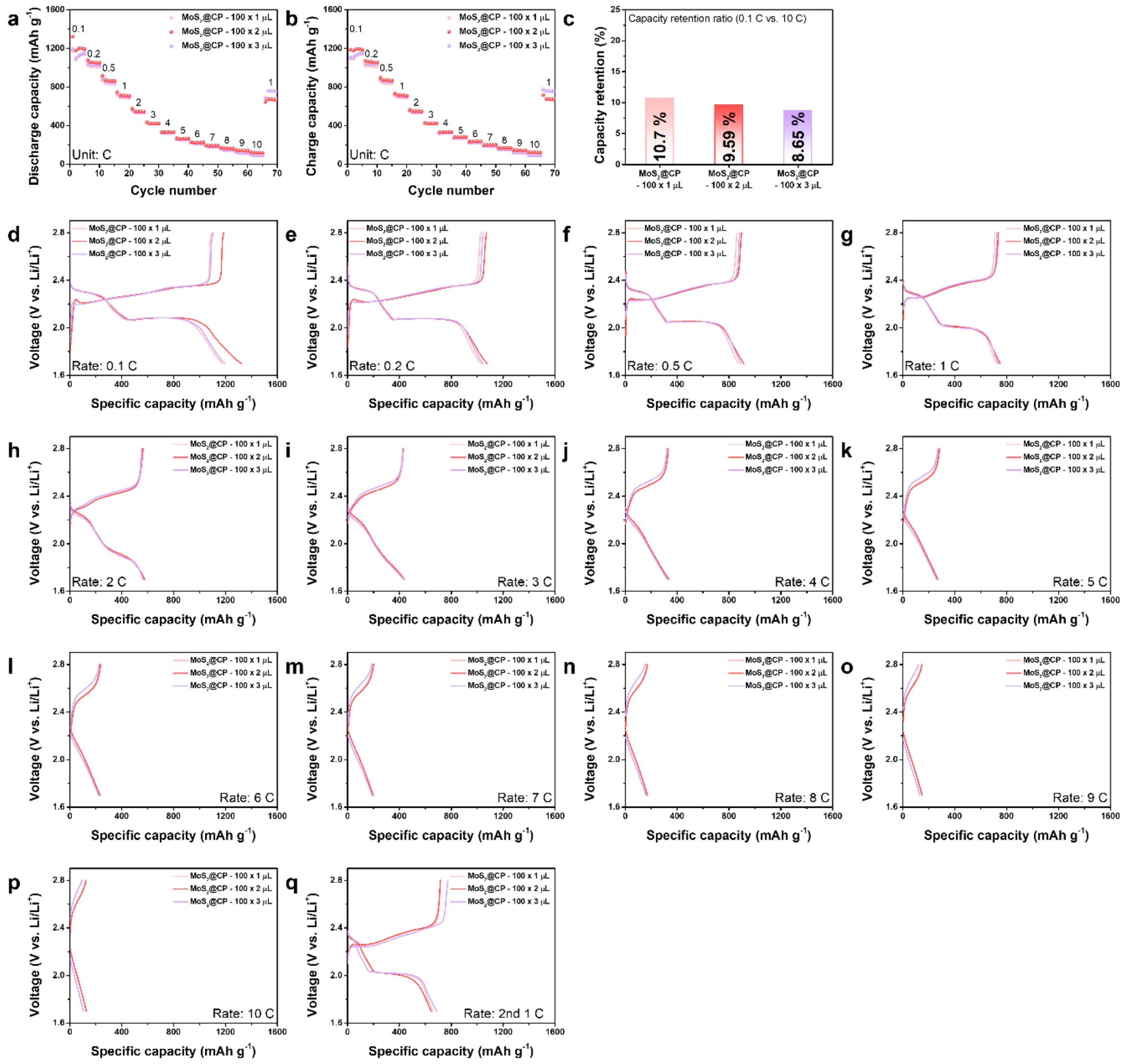

Supplementary Note Figure N2. Comparison of electrochemical performances including (a) rate capability in discharge capacity, (b) rate capability in charge capacity, (c) capacity retention at 10 C compared to 0.1 C, and (d–q) corresponding galvanostatic charge/discharge profiles of MoS_2@CP - 100 × 1 μL, MoS_2@CP - 100 × 2 μL, and MoS_2@CP - 100 × 3 μL cells.

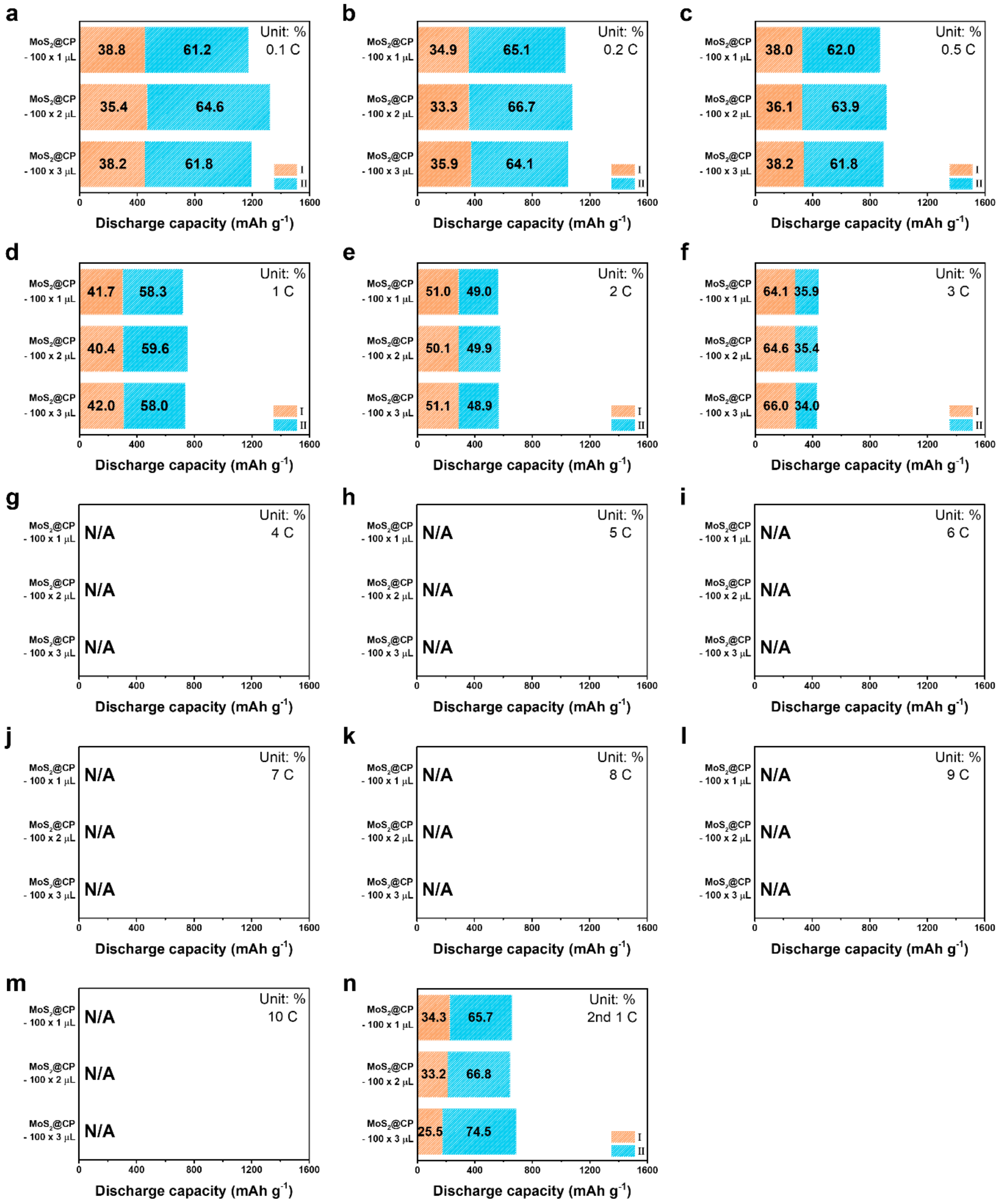

Supplementary Note Figure N3. Discharge capacity ratios calculated from extents of the first plateau (denoted as I) and second plateau (denoted as II) at different current rates of (a) 0.1 C, (b) 0.2 C, (c) 0.5 C, (d) 1 C, (e) 2 C, (f) 3 C, (g) 4 C, (h) 5 C, (i) 6 C, (j) 7 C, (k) 8 C, (l) 9 C, (m) 10 C, and (n) 2nd 0.1 C for the MoS_2@CP - 100 × 1 μL, MoS_2@CP - 100 × 2 μL, and MoS_2@CP - 100 × 3 μL cells.

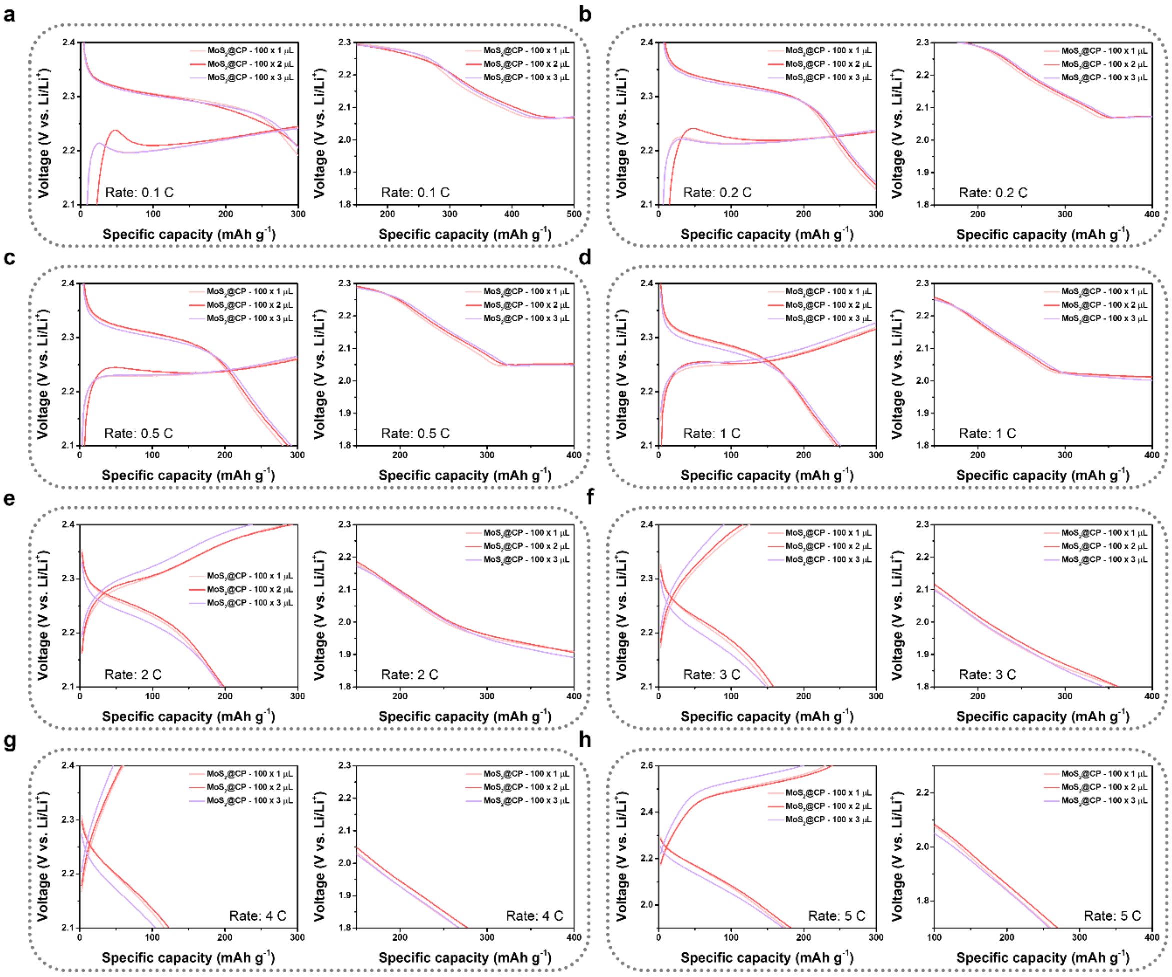

Supplementary Note Figure N4. Galvanostatic charge-discharge profiles with enlarged areas showing the 1st discharge plateau and beginning of the charge process, and the 2nd discharge plateau at different current rates of (a) 0.1 C, (b) 0.2 C, (c) 0.5 C, (d) 1 C, (e) 2 C, (f) 3 C, (g) 4 C, and (h) 5 C for the MoS_2@CP - 100 × 1 µL, MoS_2@CP - 100 × 2 µL, and MoS_2@CP - 100 × 3 µL cells.

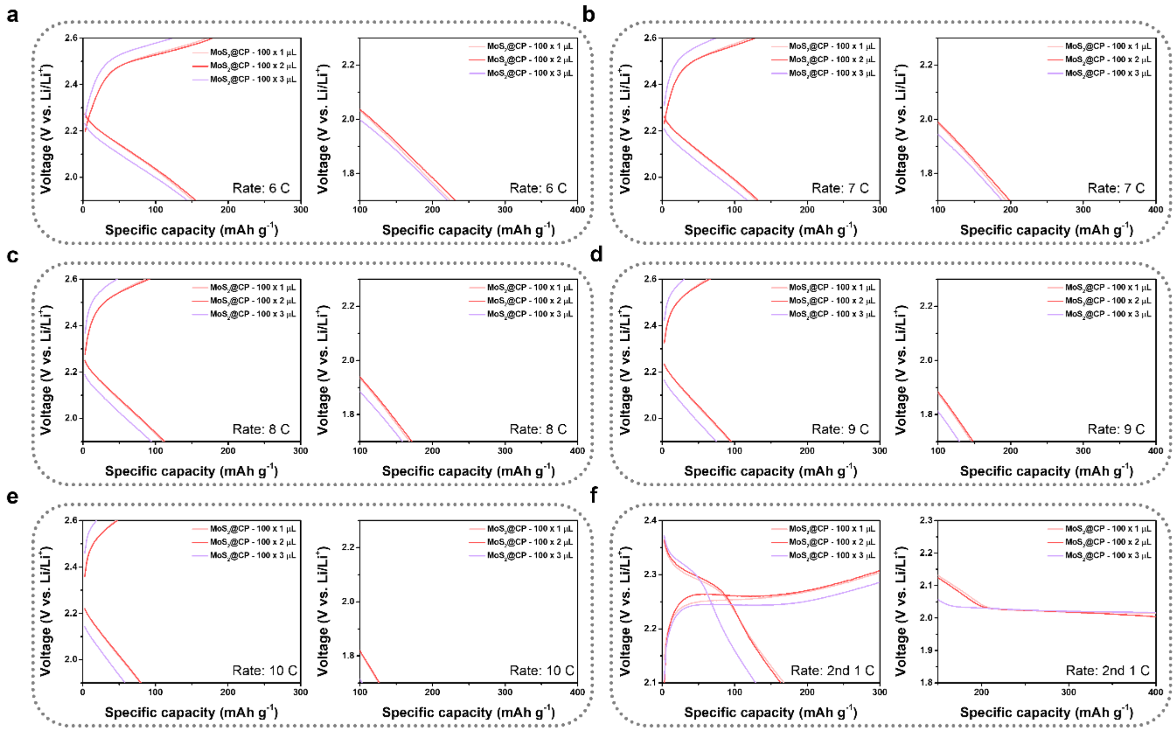

Supplementary Note Figure N5. Galvanostatic charge-discharge profiles with enlarged areas showing the 1st discharge plateau and beginning of the charge process, and the 2nd discharge plateau at different current rates of (a) 6 C, (b) 7 C, (c) 8 C, (d) 9 C, (e) 10 C, and (f) 2nd 0.1 C for the MoS_2@CP - 100 × 1 μL, MoS_2@CP - 100 × 2 μL, and MoS_2@CP - 100 × 3 μL cells.

To determine the optimal chemical composition of the catalyst adsorbed onto the CP, we first adjusted the amount of MoS_2. As detailed in the Experimental section, the MoS_2@CP - 100 × 1 μL, MoS_2@CP - 100 × 2 μL, and MoS_2@CP - 100 × 3 μL interlayers were fabricated by drop-casting 100, 200, and 300 μL of the as-prepared metal precursor solution, respectively, onto CP disks. In the initial experiment, a rate capability test was conducted to evaluate the charge and discharge capacities, from which the capacity retention ratio between 0.1 C and 10 C rates was calculated (Supplementary Note Figure N2a–c). Consequently, the MoS_2@CP - 200 × 1 μL cell maintained the highest capacity even as the current rate increased. Although the MoS_2@CP - 100 × 1 μL cell showed the highest capacity retention ratio of 10.7%, its absolute capacity values at different current densities were relatively low. The MoS_2@CP - 300 × 1 μL cell exhibited the highest capacity when the current rate returned from 10 C to 1 C, but similarly demonstrated a poor ability to maintain capacity at high current rates. Furthermore, the polarization voltage at 50% DoD was consistently the lowest for the MoS_2@CP - 200 × 1 μL cell (Supplementary Note Figure N2d–q). In addition, the MoS_2@CP - 200 × 1 μL cell exhibited a consistently superior II/I plateau ratio and reduced overpotential relative to the control cells

throughout the rate capability assessment (Supplementary Note Figures N3a–n, N4a–h, and N5a–f). This serves as further evidence of the efficient conversion to Li_2S. Based on these results, we designated the MoS_2@CP - 200 × 1 μL cell as the pristine condition, MoS_2@CP.

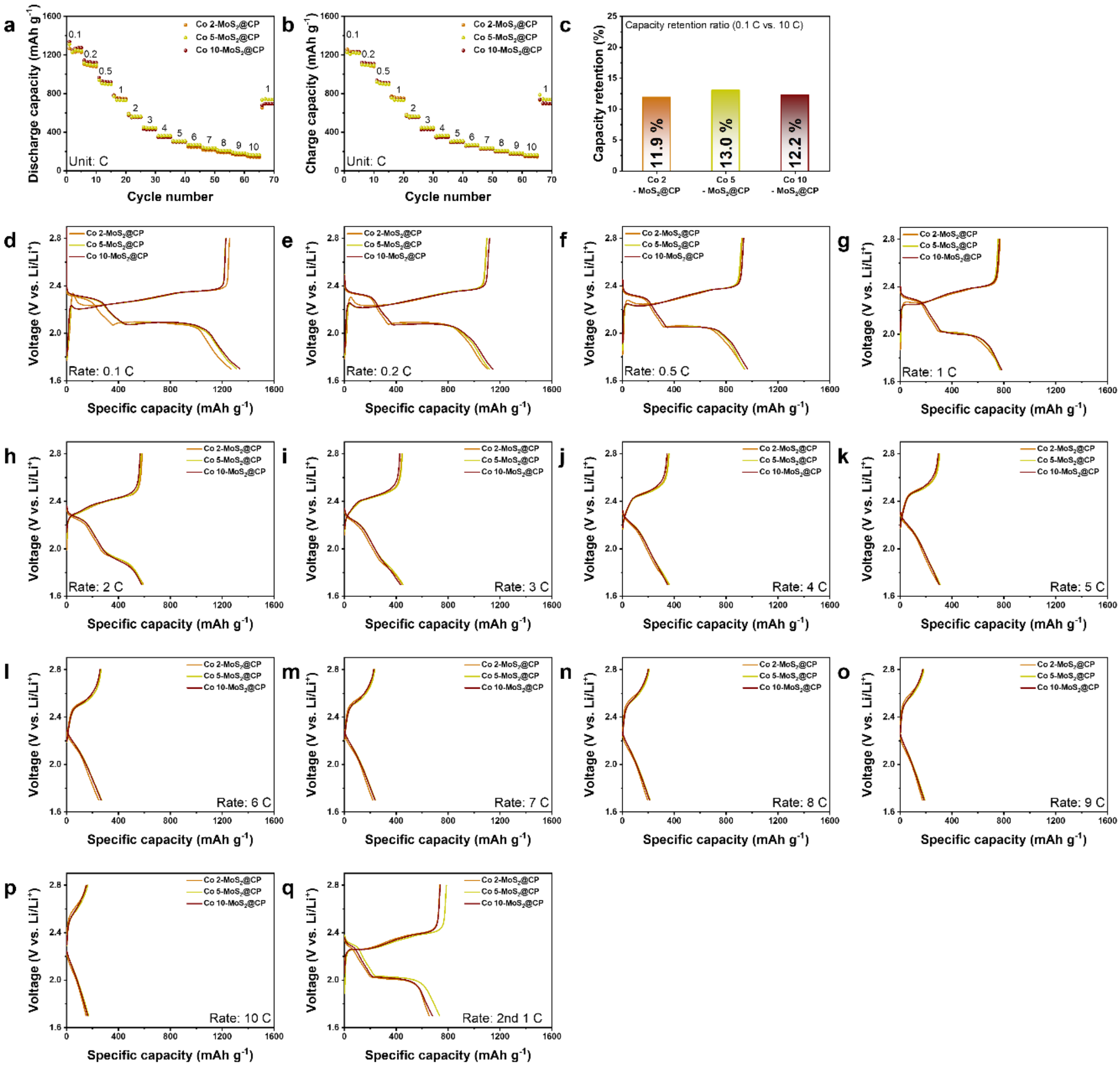

Supplementary Note Figure N6. Comparison of electrochemical performances including (a) rate capability in discharge capacity, (b) rate capability in charge capacity, (c) capacity retention at 10 C compared to 0.1 C, and (d–q) corresponding galvanostatic charge/discharge profiles of Co 2-MoS_2@CP, Co 5-MoS_2@CP, and Co 10-MoS_2@CP cells.

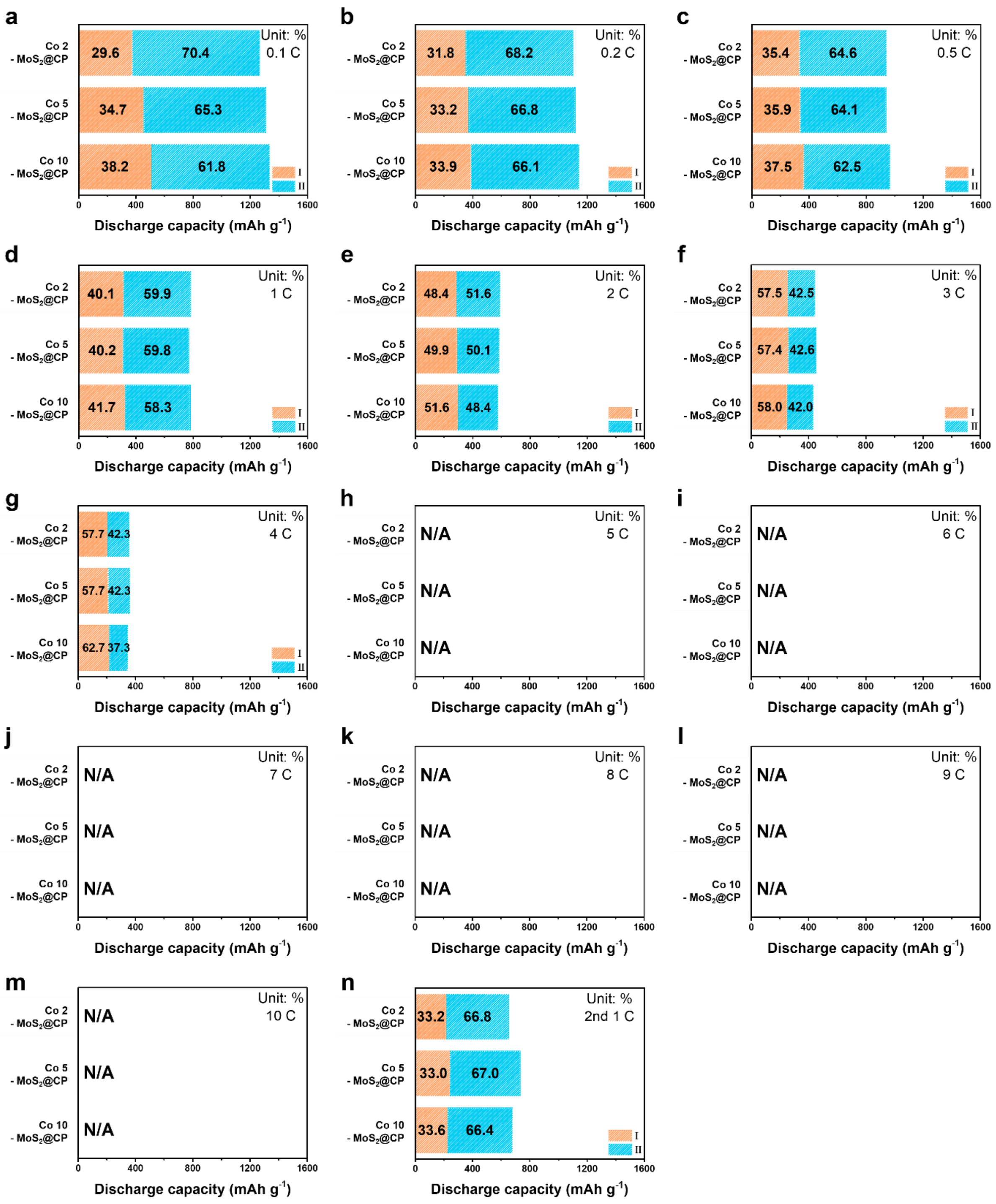

Supplementary Note Figure N7. Discharge capacity ratios calculated from extents of the first plateau (denoted as I) and second plateau (denoted as II) at different current rates of (a) 0.1 C, (b) 0.2 C, (c) 0.5 C, (d) 1 C, (e) 2 C, (f) 3 C, (g) 4 C, (h) 5 C, (i) 6 C, (j) 7 C, (k) 8 C, (l) 9 C, (m) 10 C, and (n) 2nd 0.1 C for the Co 2-MoS_2@CP, Co 5-MoS_2@CP, and Co 10-MoS_2@CP cells.

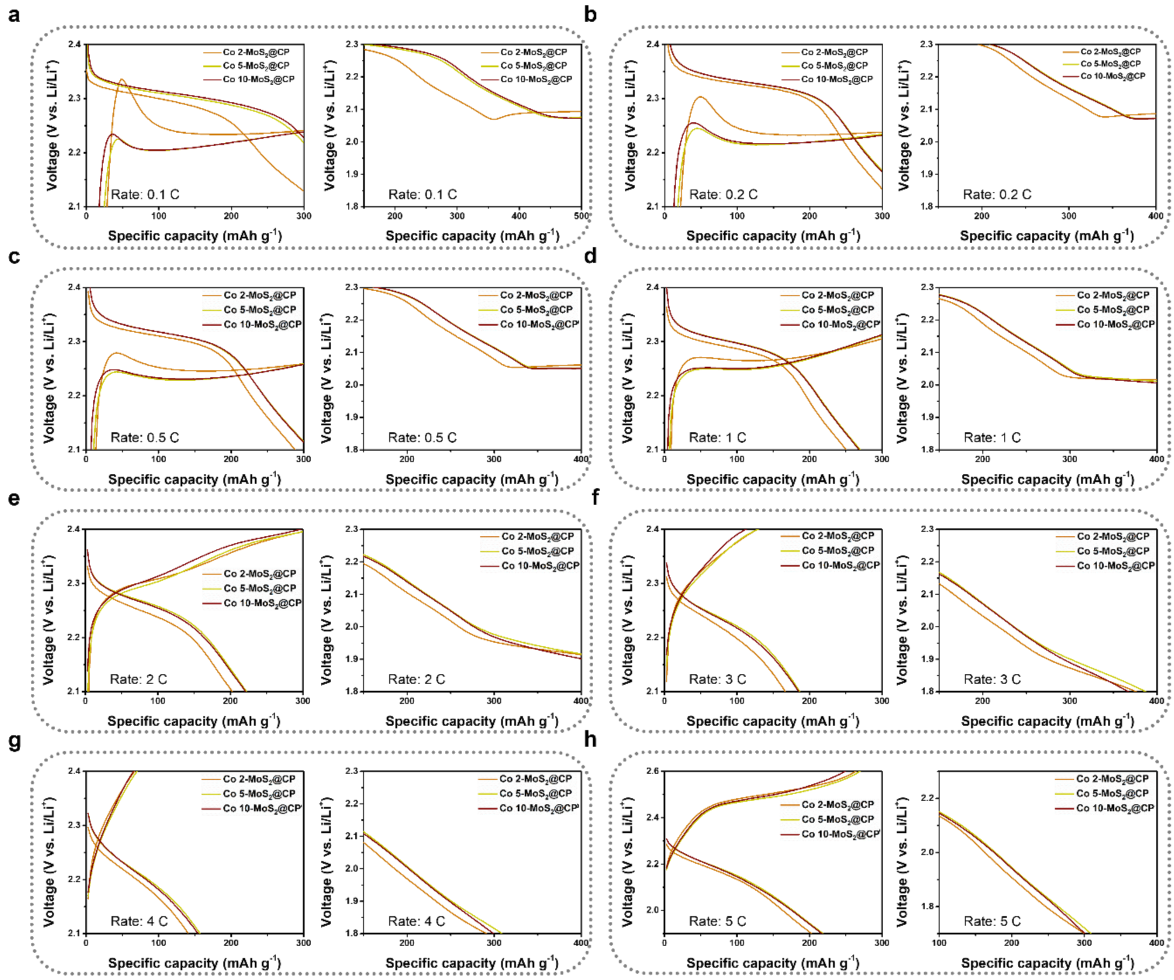

Supplementary Note Figure N8. Galvanostatic charge-discharge profiles with enlarged areas showing the 1st discharge plateau and beginning of the charge process, and the 2nd discharge plateau at different current rates of (a) 0.1 C, (b) 0.2 C, (c) 0.5 C, (d) 1 C, (e) 2 C, (f) 3 C, (g) 4 C, and (h) 5 C for the Co 2-MoS_2@CP, Co 5-MoS_2@CP, and Co 10-MoS_2@CP cells.

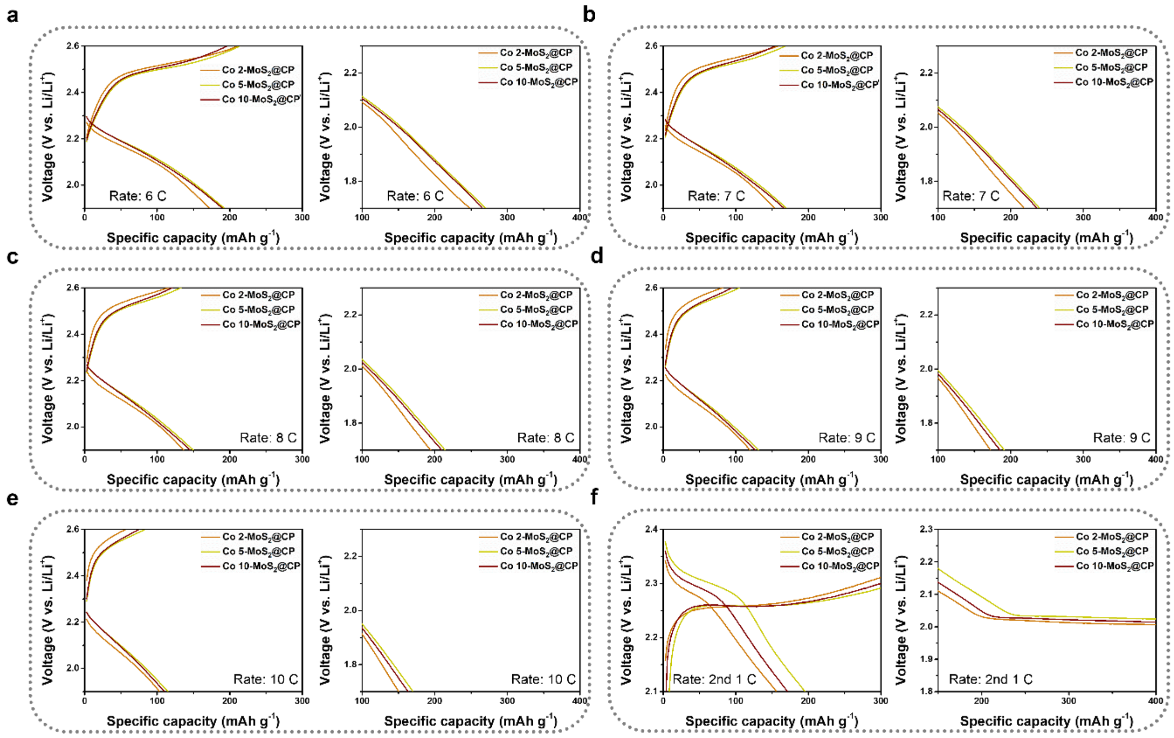

Supplementary Note Figure N9. Galvanostatic charge-discharge profiles with enlarged areas showing the 1st discharge plateau and beginning of the charge process, and the 2nd discharge plateau at different current rates of (a) 6 C, (b) 7 C, (c) 8 C, (d) 9 C, (e) 10 C, and (f) 2nd 0.1 C for the Co 2-MoS_2@CP, Co 5-MoS_2@CP, and Co 10-MoS_2@CP cells.

Subsequent to the optimization of the MoS_2 loading, the TM doping concentration was modulated. Interlayers were fabricated by synthesizing MoS_2 with 2%, 5%, and 10% cobalt dopant precursor, which were then subjected to identical rate capability testing (Supplementary Note Figure N6a–c). As a result, the Co 5-MoS_2@CP cell sustained the highest capacity amidst escalating current rates. In contrast, the Co 2-MoS_2@CP cell displayed the lowest relative capacity across all rates, accompanied by a modest retention ratio of 11.9%. While the Co 10-MoS_2@CP cell demonstrated high capacity at low rates, its performance deteriorated significantly at higher currents. Moreover, the Co 5-MoS_2@CP cell consistently maintained the lowest polarization voltage at 50% DoD (Supplementary Note Figure N6d–q) and demonstrated a superior II/I plateau ratio with reduced overpotential (Supplementary Note Figures N7a–n, N8a–h, and N9a–f), confirming efficient Li_2S conversion. Accordingly, the Co 5-MoS_2@CP formulation was established as the standard doping condition, which is labelled as Co-MoS_2@CP.

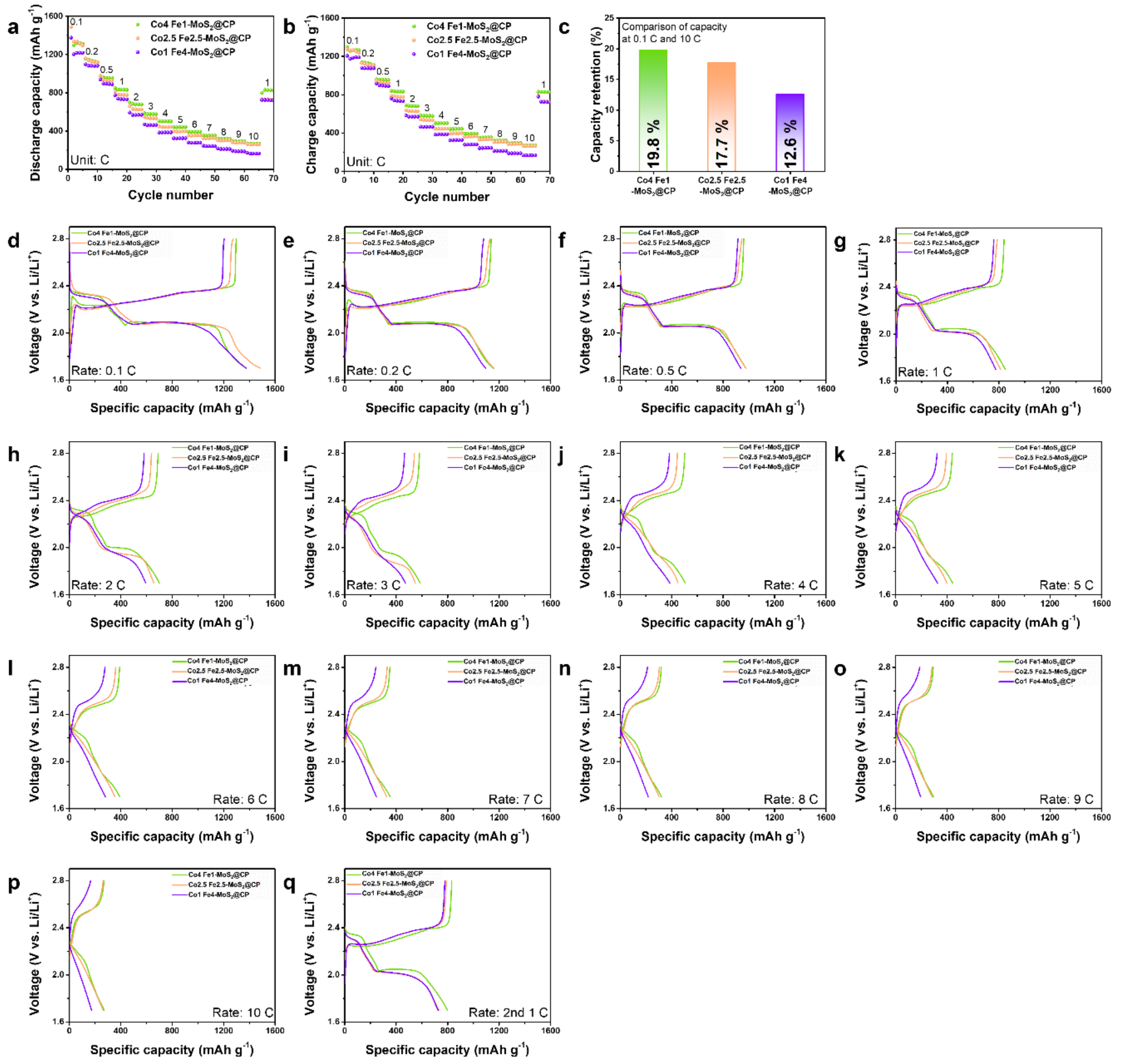

Supplementary Note Figure N10. Comparison of electrochemical performances including (a) rate capability in discharge capacity, (b) rate capability in charge capacity, (c) capacity retention at 10 C compared to 0.1 C, and (d–q) corresponding galvanostatic charge/discharge profiles of Co4 Fe1-MoS_2@CP, Co2.5 Fe2.5-MoS_2@CP, and Co1 Fe4-MoS_2@CP cells.

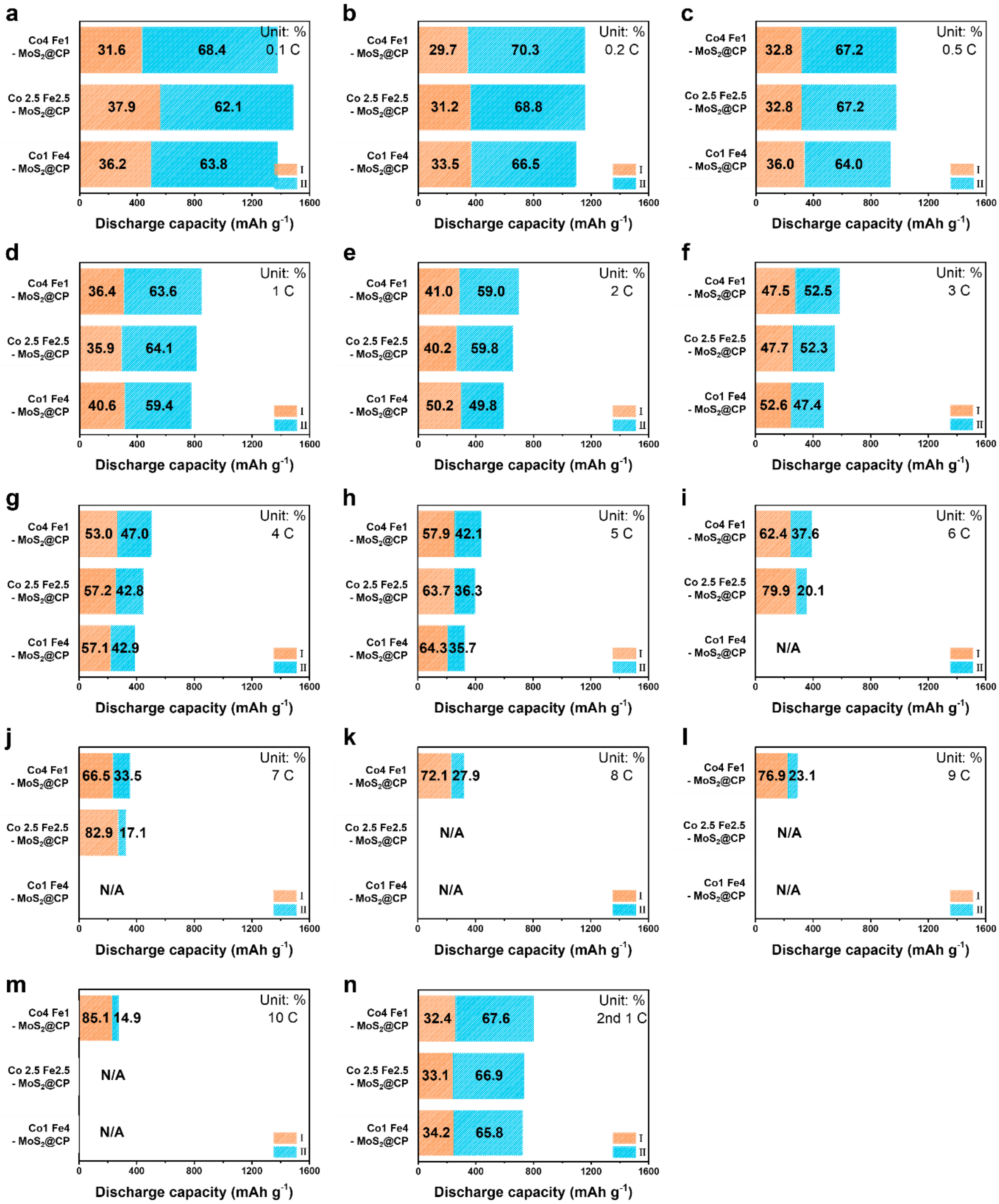

Supplementary Note Figure N11. Discharge capacity ratios calculated from extents of the first plateau (denoted as I) and second plateau (denoted as II) at different current rates of (a) 0.1 C, (b) 0.2 C, (c) 0.5 C, (d) 1 C, (e) 2 C, (f) 3 C, (g) 4 C, (h) 5 C, (i) 6 C, (j) 7 C, (k) 8 C, (l) 9 C, (m) 10 C, and (n) 2nd 0.1 C for the Co4 Fe1-MoS_2@CP, Co2.5 Fe2.5-MoS_2@CP, and Co1 Fe4-MoS_2@CP cells.

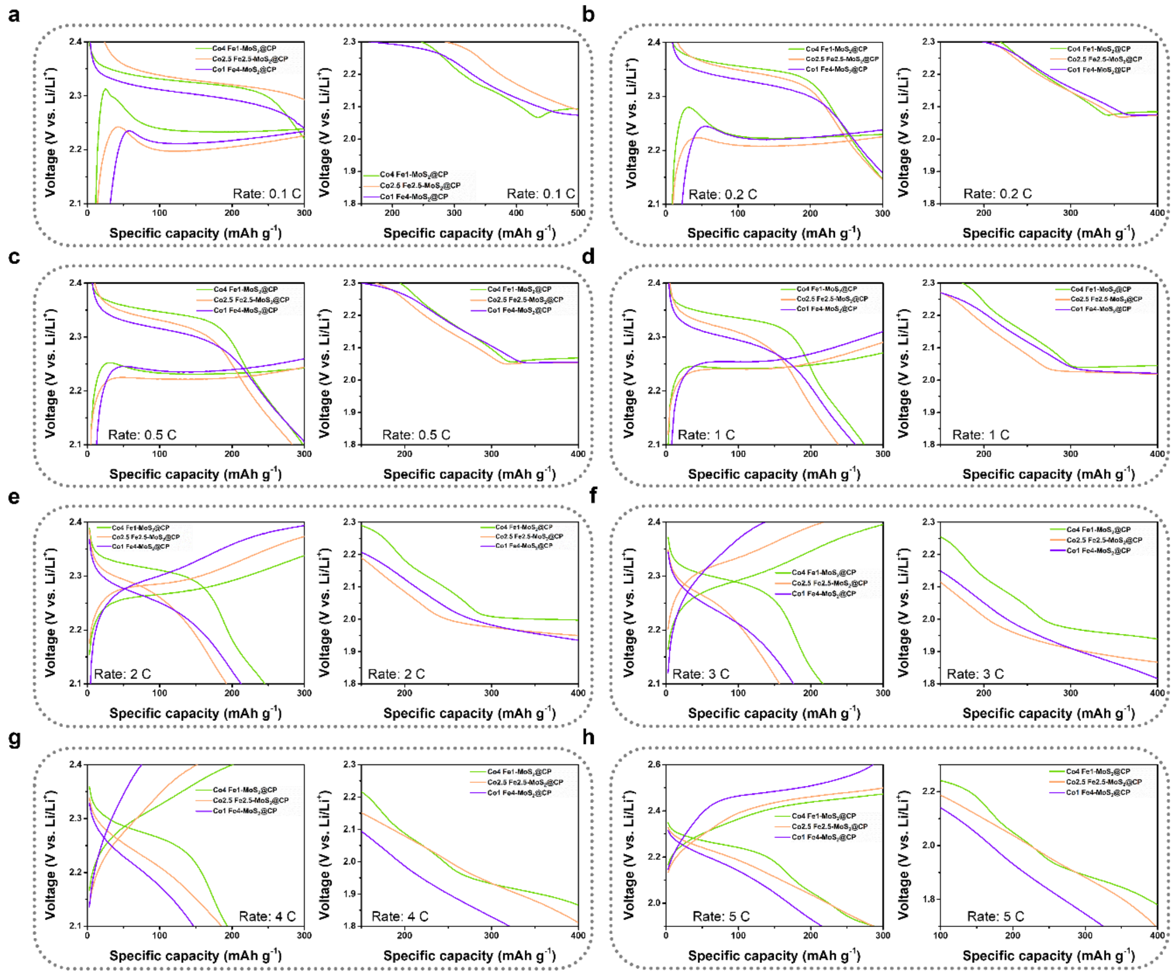

Supplementary Note Figure N12. Galvanostatic charge-discharge profiles with enlarged areas showing the 1st discharge plateau and beginning of the charge process, and the 2nd discharge plateau at different current rates of (a) 0.1 C, (b) 0.2 C, (c) 0.5 C, (d) 1 C, (e) 2 C, (f) 3 C, (g) 4 C, and (h) 5 C for the Co4 Fe1-MoS_2@CP, Co2.5 Fe2.5-MoS_2@CP, and Co1 Fe4-MoS_2@CP cells.

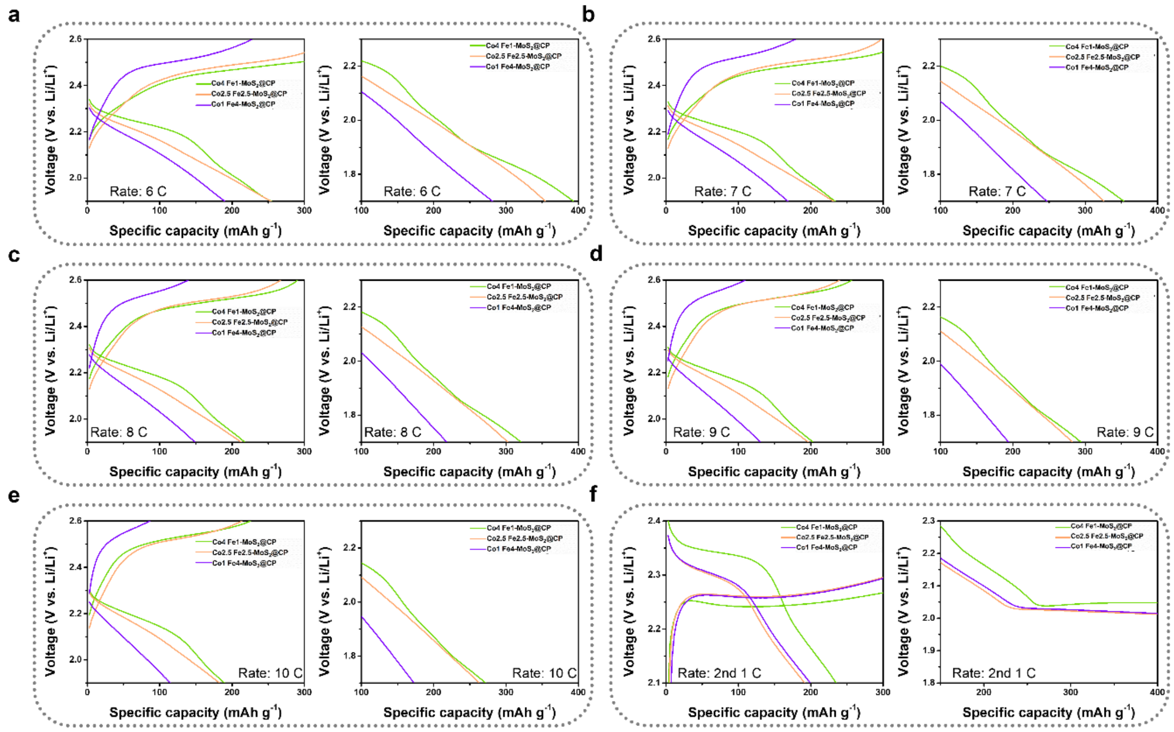

Supplementary Note Figure N13. Galvanostatic charge-discharge profiles with enlarged areas showing the 1st discharge plateau and beginning of the charge process, and the 2nd discharge plateau at different current rates of (a) 6 C, (b) 7 C, (c) 8 C, (d) 9 C, (e) 10 C, and (f) 2nd 0.1 C for the Co4 Fe1-MoS_2@CP, Co2.5 Fe2.5-MoS_2@CP, and Co1 Fe4-MoS_2@CP cells.

Since the core strategy of this study is the co-doping of Co and Fe, the final optimization step involved adjusting the Co-to-Fe doping ratio while maintaining a total added dopant precursor concentration of 5%. Specifically, interlayers with Co/Fe ratios of 4:1, 2.5:2.5, and 1:4, denoted as Co4 Fe1-MoS_2@CP, Co2.5 Fe2.5-MoS_2@CP, and Co1 Fe4-MoS_2@CP, respectively, were fabricated to determine the optimal dopant stoichiometry. The rate capability results for cells fabricated with these interlayers show that the Co4 Fe1-MoS_2@CP cell exhibited the most superior discharge capacity, even under fast current rate conditions, while simultaneously demonstrating a high capacity retention ratio of 19.8% (Supplementary Note Figure N10a–c). Conversely, an increase in the relative Fe doping ratio led to a decline in overall cell performance. As expected, the Co4 Fe1-MoS_2@CP cell maintained the lowest polarization voltage across all current rate conditions, indicating efficient sulfur conversion reactions (Supplementary Note Figure N10d–q). Interestingly, only the Co4 Fe1-MoS_2@CP cell allowed for the determination of the II/I plateau ratio at all current rates, consistently maintaining the highest ratio values. This proves its catalytic superiority in sulfur species redox reactions (Supplementary Note Figure N11a–n). Similarly, the Co4 Fe1-MoS_2@CP cell maintained low

overpotentials at the onset of discharge and charge for all processes, confirming that the most efficient Li-S cell operation is achieved with this interlayer (Supplementary Note Figures N12a–n and N13a–f). Based on this outstanding performance, the Co4 Fe1-MoS_2@CP cell was designated as CoFe-MoS_2@CP for further study.

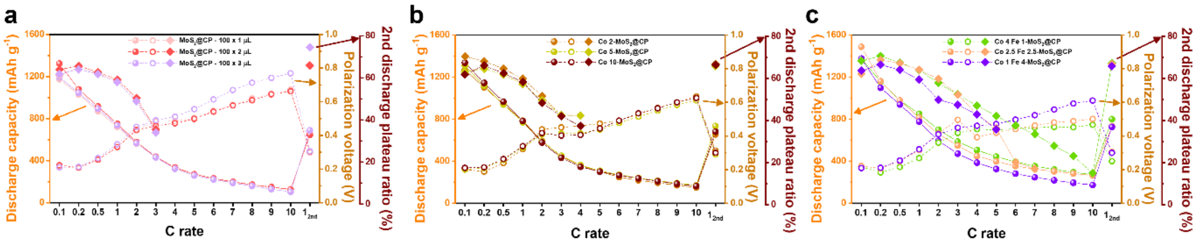

Supplementary Note Figure N14. Overall electrochemical rate capability performance comparison showing discharge capacity, polarization voltage at DoD 50%, and 2nd discharge plateau ratio for the (a) MoS_2@CP - 100 × 1 μL, MoS_2@CP - 100 × 2 μL, MoS_2@CP - 100 × 3 μL cells, (b) Co 2-MoS_2@CP, Co 5-MoS_2@CP, Co 10-MoS_2@CP cells, and (c) Co4 Fe1-MoS_2@CP, Co2.5 Fe2.5-MoS_2@CP, and Co1 Fe4-MoS_2@CP cells.

All the aforementioned results are comprehensively summarized in Supplementary Note Figure N14a–c. Consequently, the interlayers utilizing MoS_2@CP - 100 × 2 μL, Co 5-MoS_2@CP, and Co4 Fe1-MoS_2@CP in each respective optimization process consistently showed high discharge capacity, low polarization voltage at 50% DoD, and a high 2nd discharge plateau ratio. Accordingly, these specific formulations were identified as the optimized conditions.

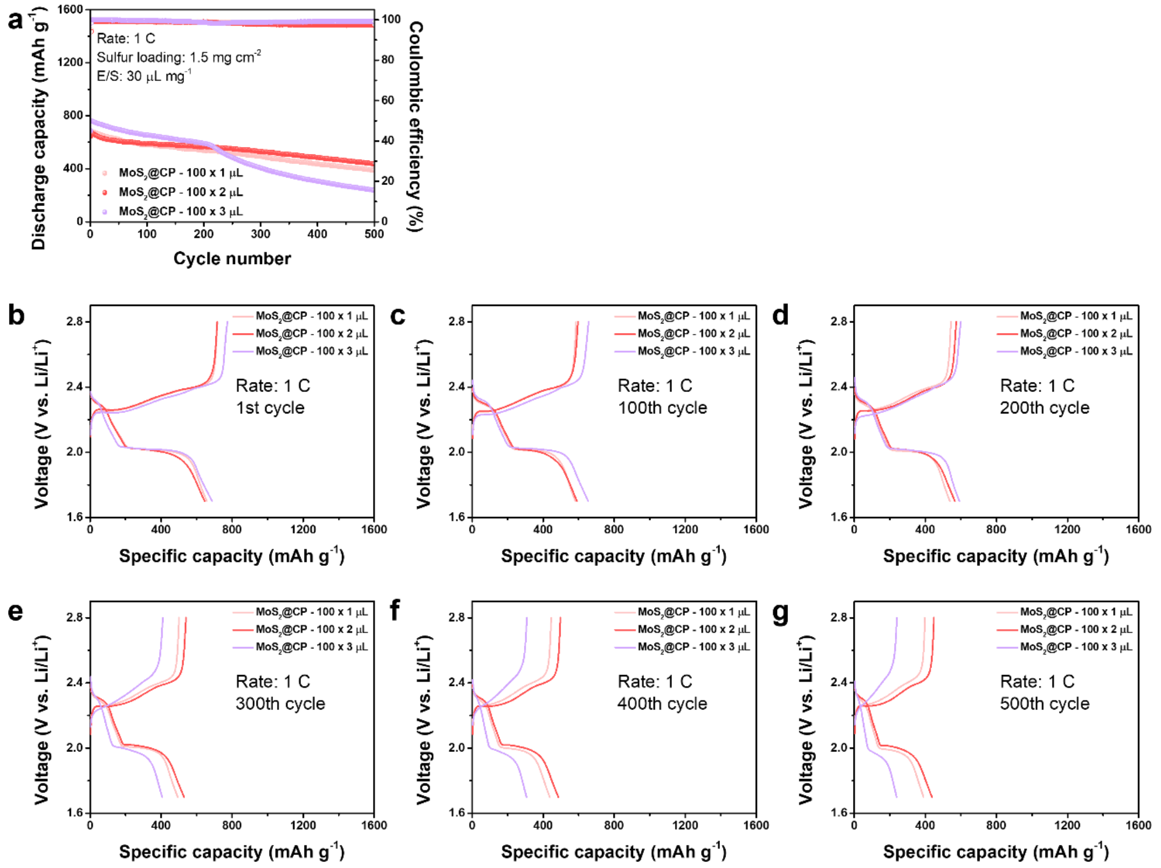

Supplementary Note Figure N15. Long-term cycling performances (a) and specific capacity at 1 C with corresponding galvanostatic charge-discharge profiles at (b) 1st, (c) 100th, (d) 200th, (e) 300th, (f) 400th, and (g) 500th cycle for the MoS_2@CP - 100 × 1 μL, MoS_2@CP - 100 × 2 μL, and MoS_2@CP - 100 × 3 μL cells.

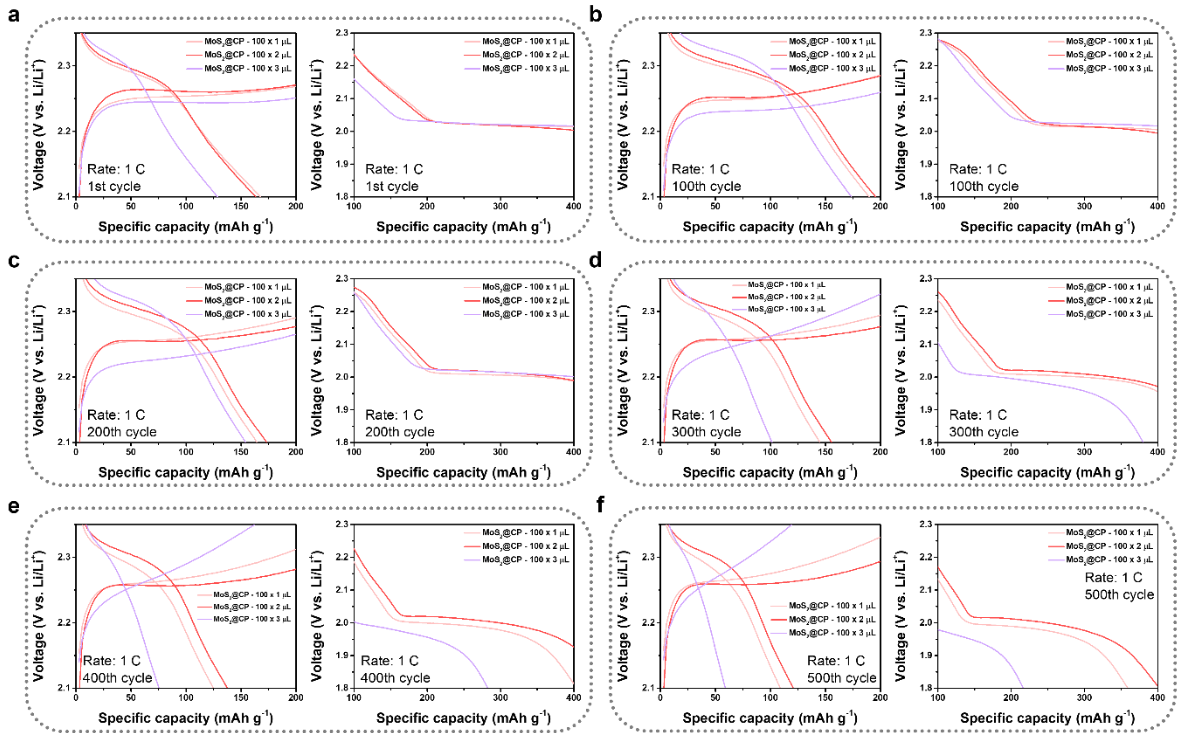

Supplementary Note Figure N16. Galvanostatic charge-discharge profiles during long-term cycling test at 1 C with enlarged areas showing the 1st discharge plateau and beginning of the charge process, and the 2nd discharge plateau at (b) 1st, (c) 100th, (d) 200th, (e) 300th, (f) 400th, and (g) 500th cycle for the MoS_2@CP - 100 × 1 μL, MoS_2@CP - 100 × 2 μL, and MoS_2@CP - 100 × 3 μL cells.

To further validate the performance of each interlayer in the Li-S battery system, long-term cycling performance was evaluated at a 1 C rate. First, the MoS_2@CP - 100 × 2 μL cell stably maintained the highest discharge capacity over 500 cycles while simultaneously demonstrating low polarization voltage (Supplementary Note Figure N15a–g). Additionally, the overpotential at the onset of discharge and charge remained low for this cell throughout the 500 cycles (Supplementary Note Figure N16a–f). These results further justify the optimization process regarding the catalyst loading amount.

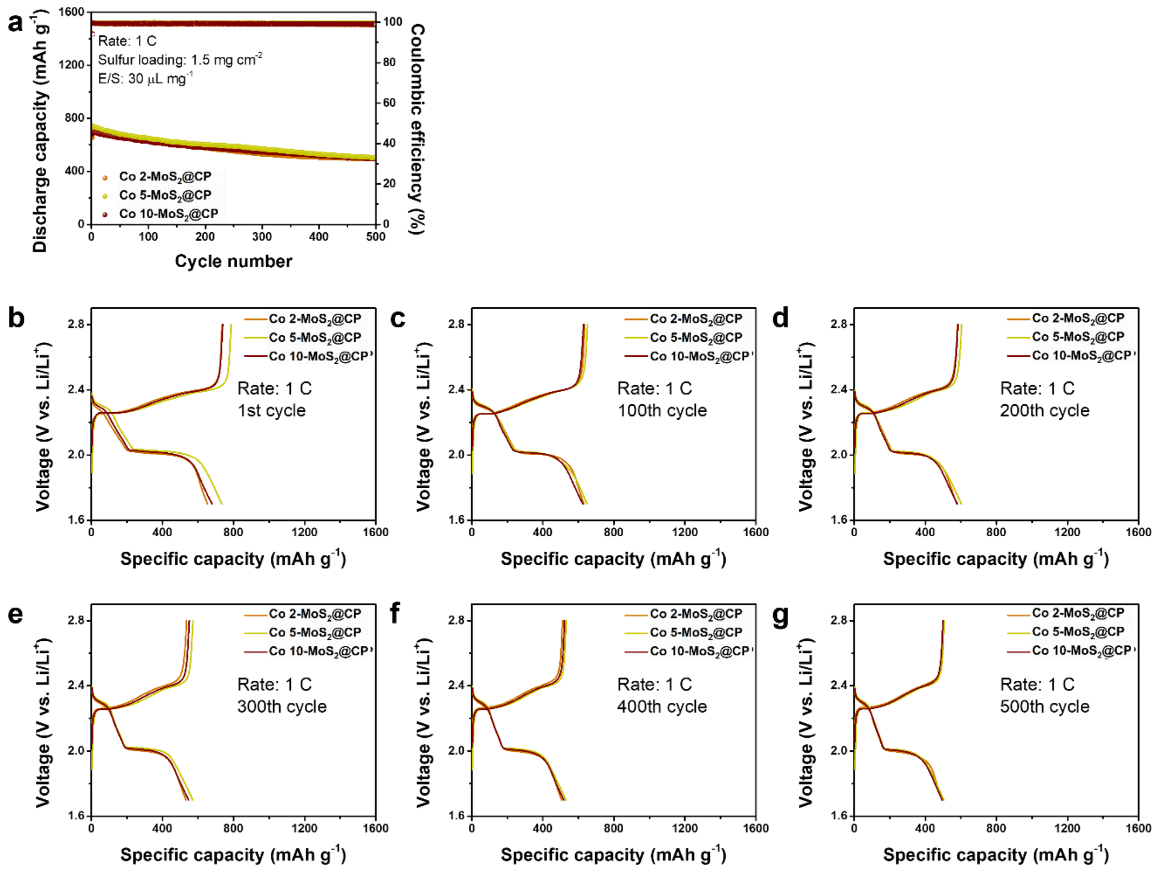

Supplementary Note Figure N17. Long-term cycling performances (a) and specific capacity at 1 C with corresponding galvanostatic charge-discharge profiles at (b) 1st, (c) 100th, (d) 200th, (e) 300th, (f) 400th, and (g) 500th cycle for the Co 2-MoS_2@CP, Co 5-MoS_2@CP, and Co 10-MoS_2@CP cells.

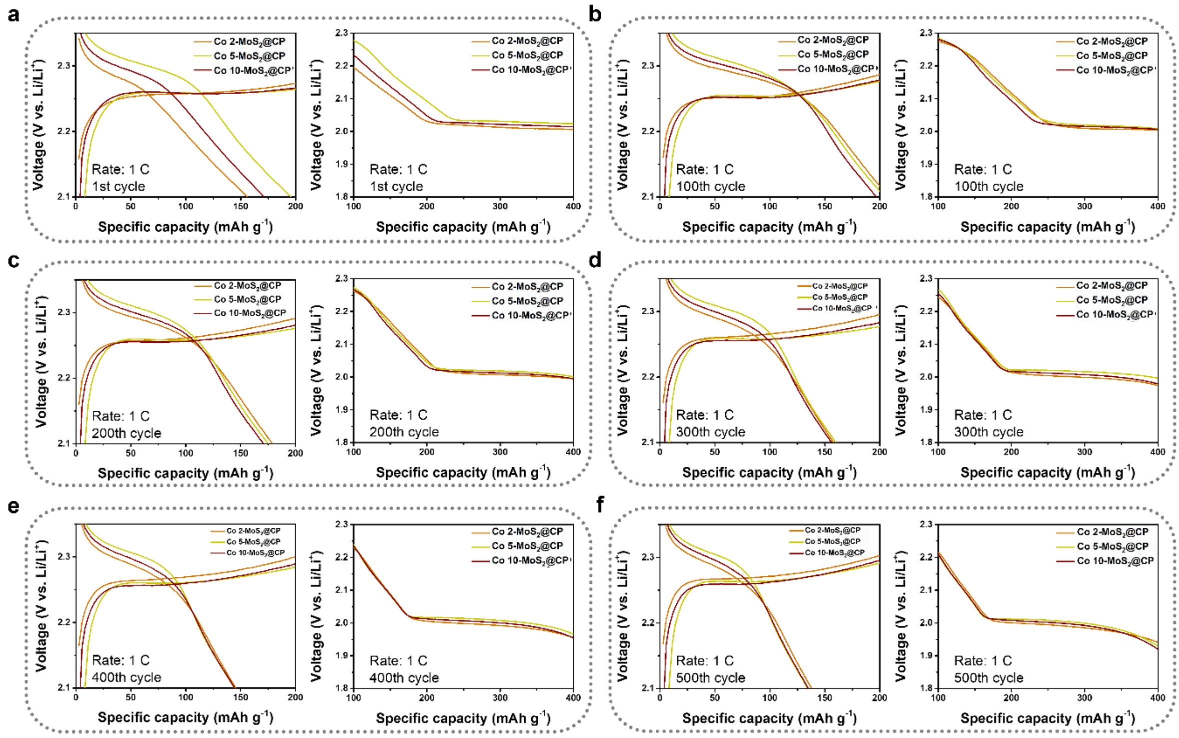

Supplementary Note Figure N18. Galvanostatic charge-discharge profiles during long-term cycling test at 1 C with enlarged areas showing the 1st discharge plateau and beginning of the charge process, and the 2nd discharge plateau at (b) 1st, (c) 100th, (d) 200th, (e) 300th, (f) 400th, and (g) 500th cycle for the Co 2-MoS_2@CP, Co 5-MoS_2@CP, and Co 10-MoS_2@CP cells.

In the experiments concerning the TM dopant amount, similar to the rate capability test, the Co 5-MoS_2@CP cell showed the highest discharge capacity while simultaneously maintaining a low polarization voltage at 50% DoD over 500 cycles (Supplementary Note Figure N17a–g). Likewise, the Co 5-MoS_2@CP cell consistently maintained the lowest overpotential at the initial stage of the discharge and charge processes throughout the long-term cycling test (Supplementary Note Figure N18a–f).

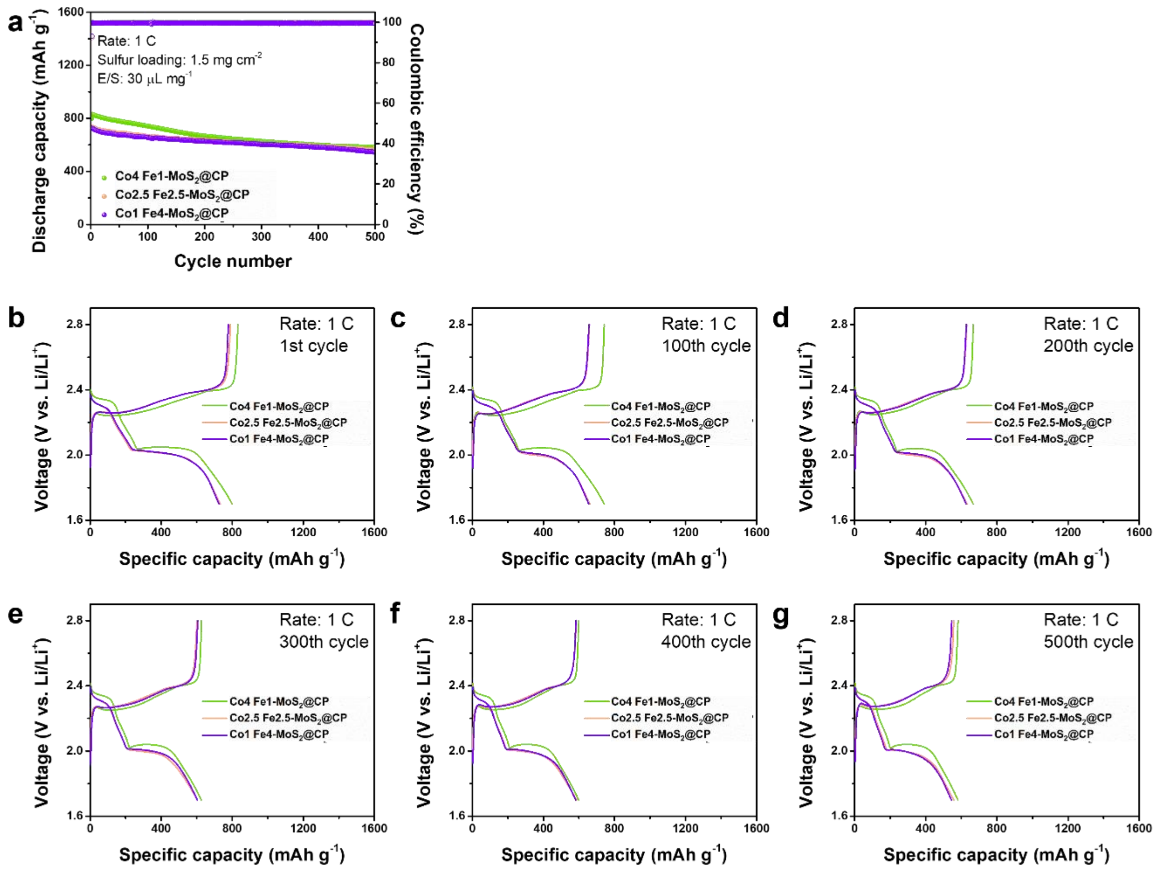

Supplementary Note Figure N19. Long-term cycling performances (a) and specific capacity at 1 C with corresponding galvanostatic charge-discharge profiles at (b) 1st, (c) 100th, (d) 200th, (e) 300th, (f) 400th, and (g) 500th cycle for the Co4 Fe1-MoS_2@CP, Co2.5 Fe2.5-MoS_2@CP, and Co1 Fe4-MoS_2@CP cells.

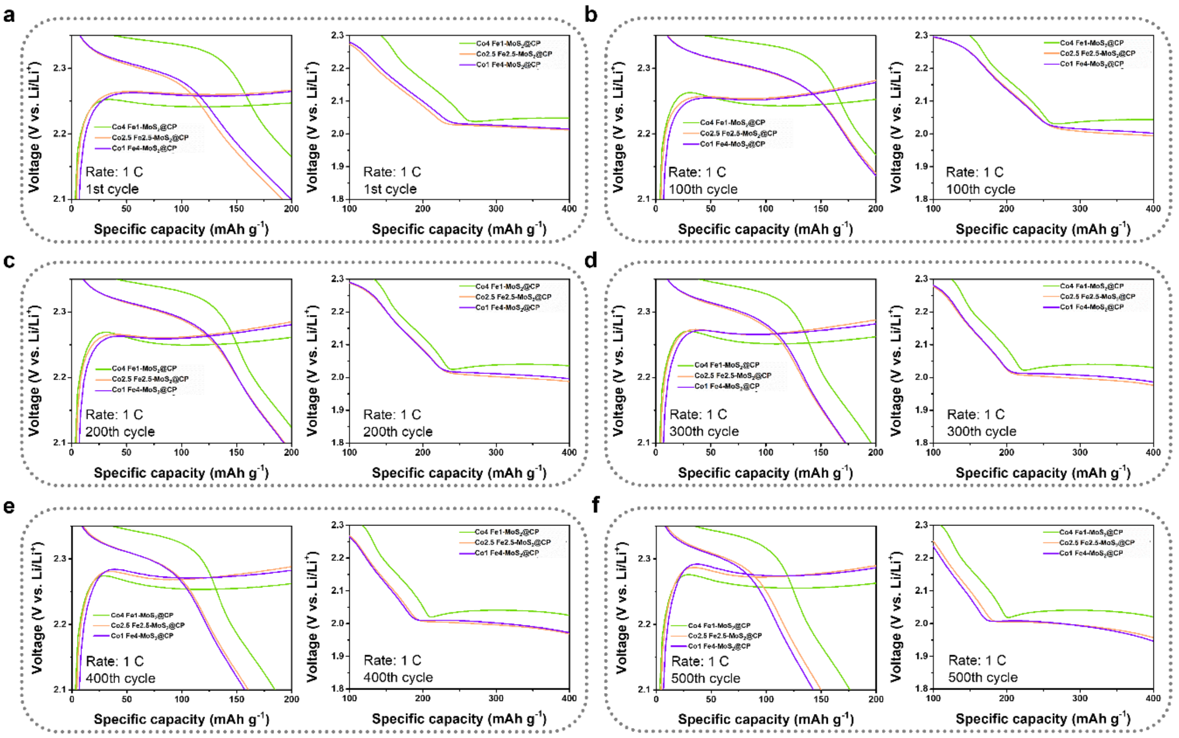

Supplementary Note Figure N20. Galvanostatic charge-discharge profiles during long-term cycling test at 1 C with enlarged areas showing the 1st discharge plateau and beginning of the charge process, and the 2nd discharge plateau at (b) 1st, (c) 100th, (d) 200th, (e) 300th, (f) 400th, and (g) 500th cycle for the Co4 Fe1-MoS_2@CP, Co2.5 Fe2.5-MoS_2@CP, and Co1 Fe4-MoS_2@CP cells.

Lastly, the Co4 Fe1-MoS_2@CP cell maintained a consistently superior discharge capacity throughout the cycling process relative to cells with alternative doping ratios. This performance is attributed to efficient sulfur redox conversion, which is corroborated by the minimal polarization voltage observed during operation (Supplementary Note Figure N19a–g). Additionally, the remarkable efficiency of the sulfur redox reaction in the Co4 Fe1-MoS_2@CP cell is underscored by the significantly reduced overpotentials recorded at the initiation of both discharge and charge phases (Supplementary Note Figure N20a–f).

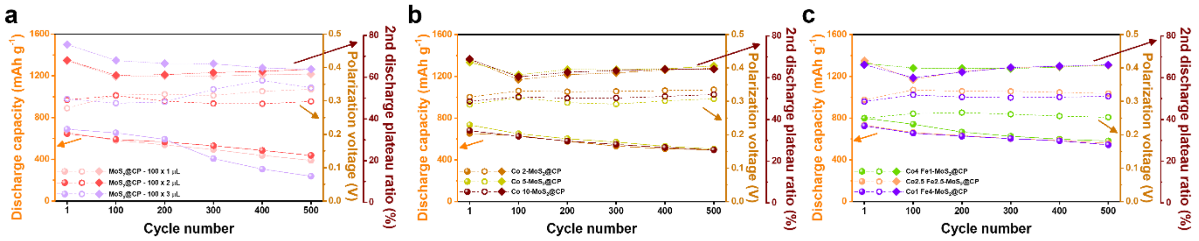

Supplementary Note Figure N21. Overall electrochemical long-term cycling performance comparison at 1 C rate showing discharge capacity, polarization voltage at DoD 50%, and 2nd discharge plateau ratio for the (a) MoS_2@CP - 100 × 1 μL, MoS_2@CP - 100 × 2 μL, MoS_2@CP - 100 × 3 μL cells, (b) Co 2-MoS_2@CP, Co 5-MoS_2@CP, Co 10-MoS_2@CP cells, and (c) Co4 Fe1-MoS_2@CP, Co2.5 Fe2.5-MoS_2@CP, and Co1 Fe4-MoS_2@CP cells.

The results for long-term cycling performance at a 1 C rate are summarized in Supplementary Note Figure N21a–c. Consistent with the previous findings, the cells utilizing the MoS_2@CP - 100 × 2 μL, Co 5-MoS_2@CP, and Co4 Fe1-MoS_2@CP interlayers in each optimization process demonstrated relatively superior cell performance. This was evidenced by their overall high discharge capacity, low polarization voltage, and high 2nd discharge plateau ratio.

Supplementary Note 4

Analysis of Li_2S nucleation and growth mechanisms

To quantitatively fit the peak profiles, dimensionless current-time transients were constructed by normalizing the current maximum (I_m) and the corresponding time (t_m). The nucleation mechanisms of Li_2S were evaluated using both the Bewick-Fleischmann-Thirsk (BFT) model and the Scharifker-Hills (SH) model, which describe two-dimensional (2D) and three-dimensional (3D) nucleation processes, respectively. These models were applied to interpret the chronoamperometric current-time transients through four specific electrochemical deposition mechanisms: (1) instantaneous nucleation and two-dimensional growth (2DI), (2) progressive nucleation and two-dimensional growth (2DP), (3) instantaneous nucleation and three-dimensional growth (3DI), and (4) progressive nucleation and three-dimensional growth (3DP). The 2D models describe atomic incorporation at the interface, leading to a planar layer of deposited material (Equations S4-S8), while the 3D models involve volumetric growth controlled by diffusion across three dimensions (Equations S9-S14).

1. 2D nucleation: A. Bewick, M. Fleischman, and H. R. Thirsk (BFT) model

Equations	Instantaneous nucleation (2DI)	Progressive nucleation (2DP)
(S4)	$I_{2DI}(t) = \frac{2\pi zFMhN_0k_g^2}{\rho} exp\left(-\frac{\pi M^2N_0k_g^2}{\rho^2}t^2\right)$	$I_{2DP}(t) = \frac{\pi zFMhAN_0k_g^2}{\rho} exp\left(-\frac{\pi M^2AN_0k_g^2}{3\rho^2}t^3\right)$
(S5)	$\frac{I}{I_m} = \frac{t}{t_m} exp\left(\frac{t^2 - t_m^2}{2t_m^2}\right)$	$\frac{I}{I_m} = \left(\frac{t}{t_m}\right)^2 exp\left(\frac{-2(t^3 - t_m^3)}{3t_m^3}\right)$
(S6)	$t_m = \left(\frac{\rho^2}{2\pi M^2N_0k_g^2}\right)^{1/2}$	$t_m = \left(\frac{2\rho^2}{\pi M^2AN_0k_g^2}\right)^{1/3}$
(S7)	$I_m = \frac{(2\pi)^{1/2}zFhN_0^{1/2}k_g}{\rho} exp(-1/2)$	$I_m = zFh\left(\frac{4\pi AN_0k_g^2\rho}{M}\right)^{1/3} exp(-2/3)$
(S8)	$I_mt_m = \frac{zF\rho h}{M} exp(-1/2)$	$I_mt_m = \frac{2zF\rho h}{M} exp(-2/3)$

2. 3D nucleation: Scharifker-Hills (SH) model

Equations	Instantaneous nucleation (3DI)	Progressive nucleation (3DP)
(S9)	$I_{3DI}(t) = \frac{zFD_0^{1/2}c}{\pi^{1/2}t^{1/2}}\{1 - \exp(-N_0\pi kD_0t)\}$	$I_{3DP}(t) = \frac{zFD_0^{1/2}c}{\pi^{1/2}t^{1/2}}\left\{1 - \exp\left(-\frac{AN_\infty\pi k'D_0t^2}{2}\right)\right\}$
(S10)	$\mathrm{k} = \left(\frac{8\pi cM}{\rho}\right)^{1/2}$	$k' = \frac{4}{3}\left(\frac{8\pi cM}{\rho}\right)^{1/2}$
(S11)	$\frac{I}{I_m} = \left(\frac{1.9542}{t/t_m}\right)^{1/2}\left\{1 - exp\left(1.2564\frac{t}{t_m}\right)\right\}$	$\frac{I}{I_m} = \left(\frac{1.2254}{t/t_m}\right)^{1/2}\left\{1 - exp\left(2.3367\left(\frac{t}{t_m}\right)^2\right)\right\}$
(S12)	$t_m = \frac{1.2564}{N_0\pi k}$	$t_m = \left(\frac{4.6733}{AN_\infty\pi k'D_0}\right)^{1/2}$
(S13)	$I_m = 0.6382zFD_0c(kN_0)^{1/2}$	$I_m = 0.4615zFD_0^{3/4}\mathrm{c}(k'AN_\infty)^{1/4}$
(S14)	$I_m^2t_m = 0.1629(zFc)^2D_0$	$I_m^2t_m = 0.2598(zFc)^2D_0$

zF: the molar charge transferred during the electrodeposition and dissolution process, where F = 96,485 C mol^{-1}

h: the layer thickness [cm]

k_g: the nucleus lateral growth-rate constant [mol cm^{-2} s^{-1}]

c: the molar concentration [mol cm^{-3}]

ρ, M: the density and molecular weight of Li_2S, where $M = 46$ g mol^{-1} and $\rho = 1.66$ g cm^{-3}

I_m, t_m: corresponding to the point of the maximum current density

A: the nucleation rate constant [s^{-1}]

N_∞: the number density of active sites

N_0, AN_∞: the density number of isolated centers for 3DI and 3DP, respectively [cm^{-2} and cm^{-2} s^{-1}]

D_0: the effective diffusion coefficient [cm^{-2} s^{-1}]

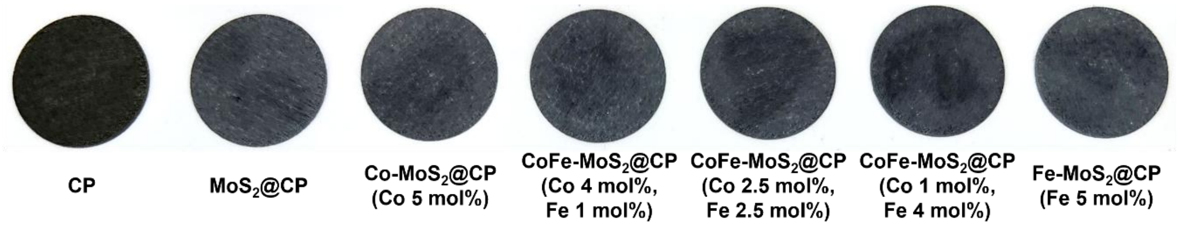

Figure S1. Visual appearance of the interlayers prepared with various catalytic compositions including CP, MoS_2@CP, Co-MoS_2@CP (Co 5 mol%), CoFe-MoS_2@CP (Co 4 mol%, Fe 1 mol%), CoFe-MoS_2@CP (Co 2.5 mol%, Fe 2.5 mol%), CoFe-MoS_2@CP (Co 1 mol%, Fe 4 mol%), and Fe-MoS_2@CP (Fe 5 mol%) catalysts.

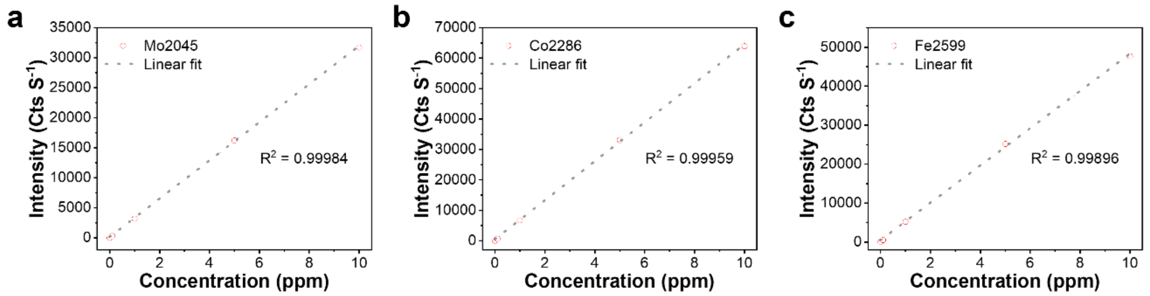

Figure S2. Linear fitting results of the calibration data for ICP-OES measurements of Mo, Co, and Fe.

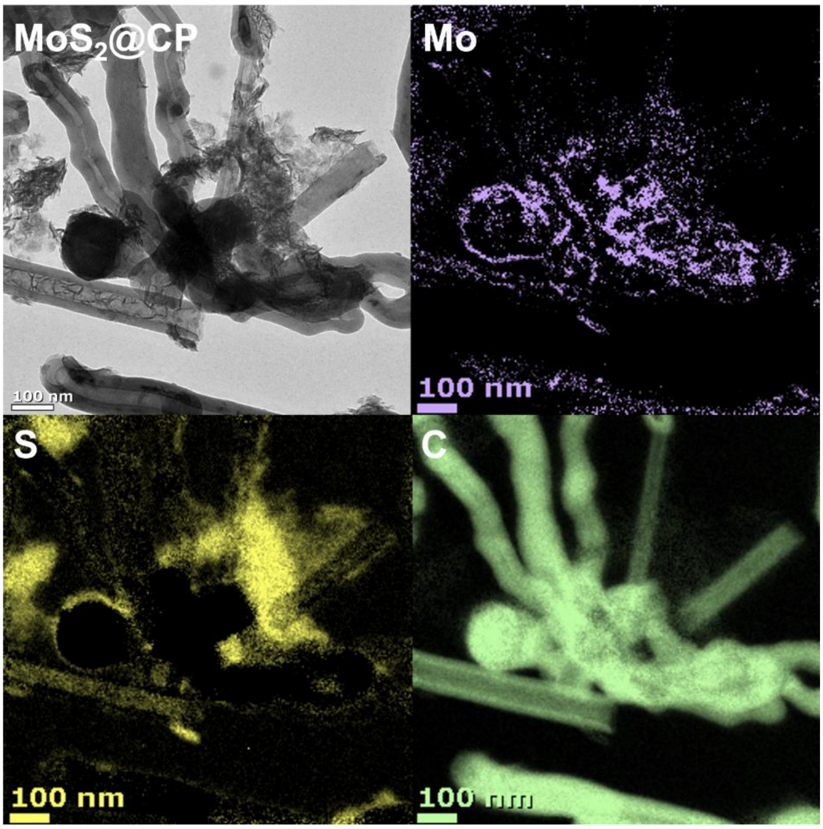

Figure S3. TEM image with corresponding EELS mappings of the MoS_2@CP consisting of Mo, S, and C elements.

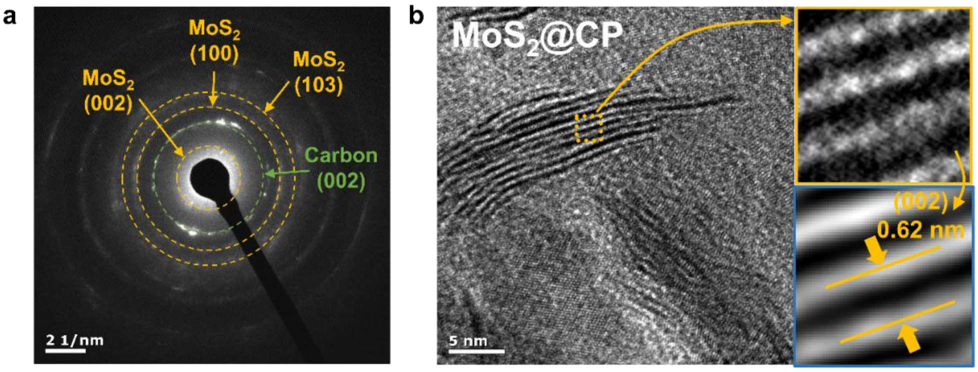

Figure S4. SAED pattern of the MoS_2@CP with corresponding HR-TEM image highlighting the (002) plane.

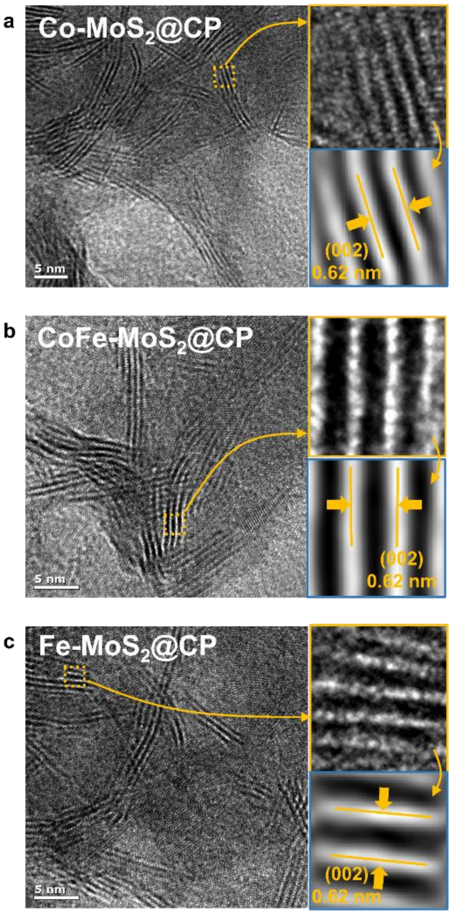

Figure S5. SAED patterns with corresponding HR-TEM images highlighting the (002) plane of the (a) Co-MoS_2@CP, (b) CoFe-MoS_2@CP, and (c) Fe-MoS_2@CP.

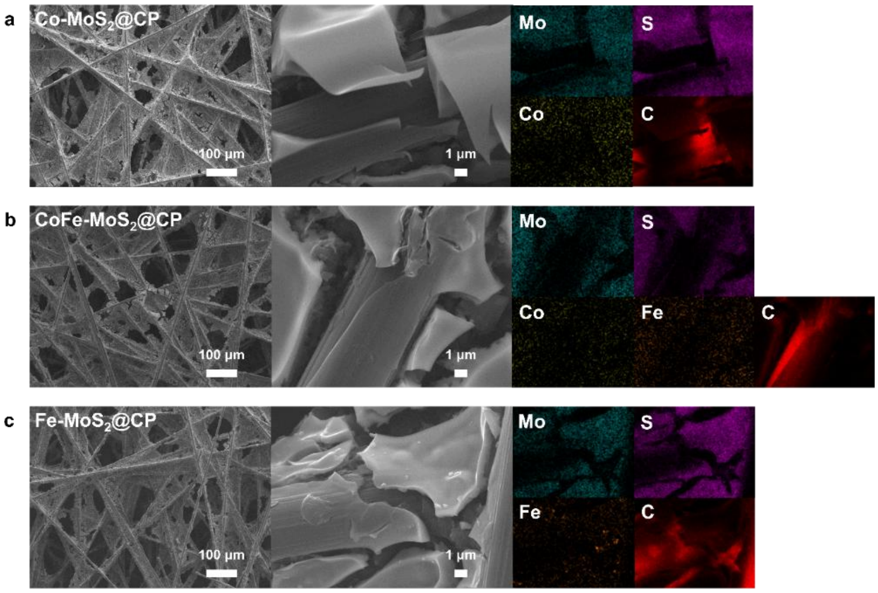

Figure S6. SEM images with corresponding EDS mappings of the (a) Co-MoS_2@CP, (b) CoFe-MoS_2@CP, and (c) Fe-MoS_2@CP consisting of Mo, S, Co, Fe, and C elements.

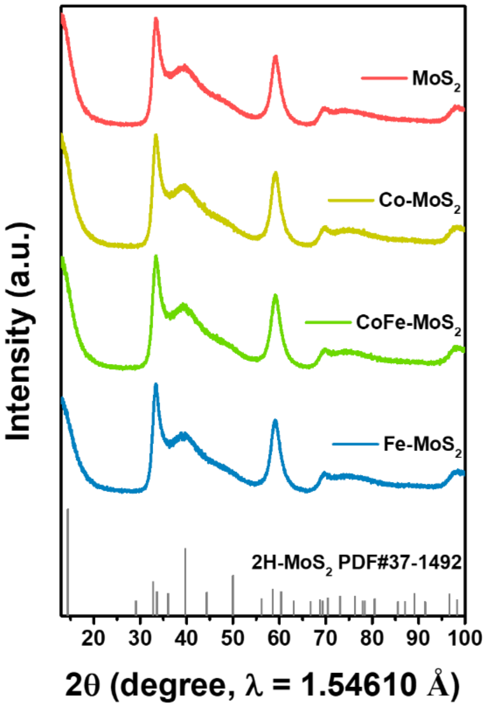

Figure S7. HRPD patterns of the MoS_2, Co-MoS_2, CoFe-MoS_2, and Fe-MoS_2 without the CP substrate.

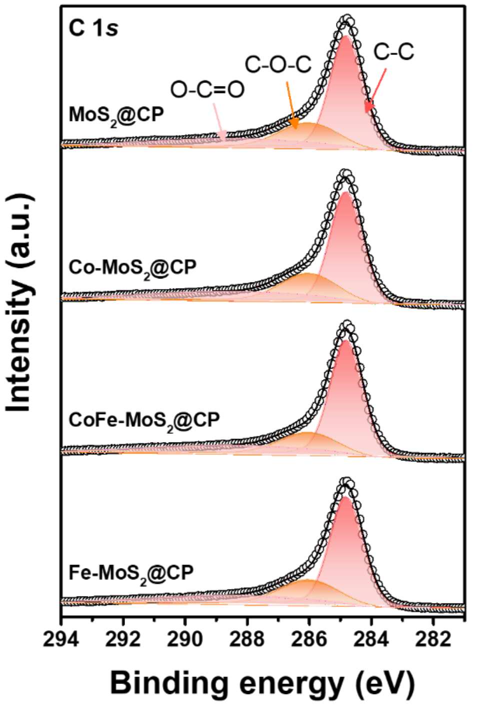

Figure S8. XPS profiles of the materials for C 1*s* region.

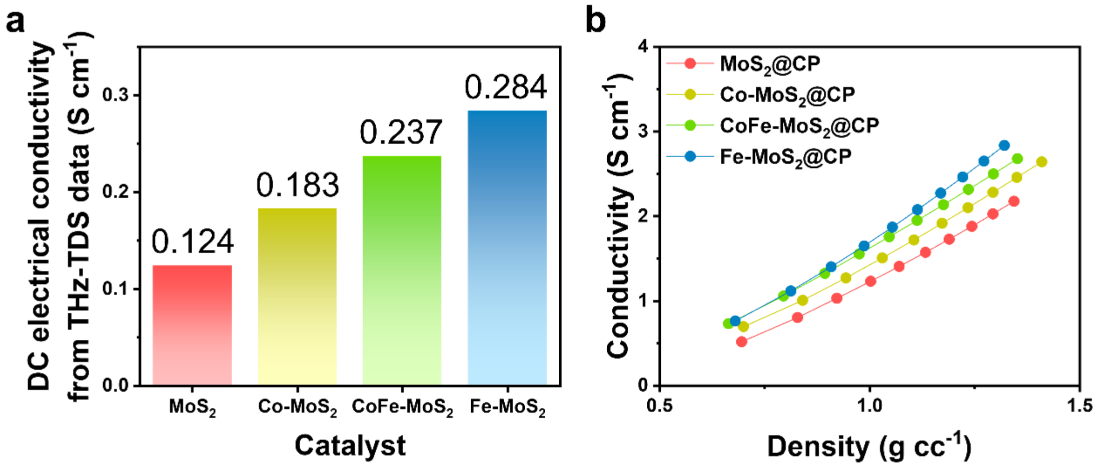

Figure S9. (a) Absolute DC electrical conductivity (σ_{DC}) of the MoS_2, Co-MoS_2, CoFe-MoS_2, and Fe-MoS_2 catalysts. The σ_{DC} values were quantitatively extracted from the THz-TDS time-domain spectra using a bulk transmission model. The frequency-dependent real conductivity was derived by evaluating the complex transmission coefficient and applying Fresnel reflection corrections for the controlled 154 μm catalyst layers on the sapphire substrate. The representative σ_{DC} values were determined by averaging the real conductivity within the stable low-frequency plateau regime (0.5–1.0 THz). (b) Powder conductivity measurements for the MoS_2@CP, Co-MoS_2@CP, CoFe-MoS_2@CP, and Fe-MoS_2@CP composites.

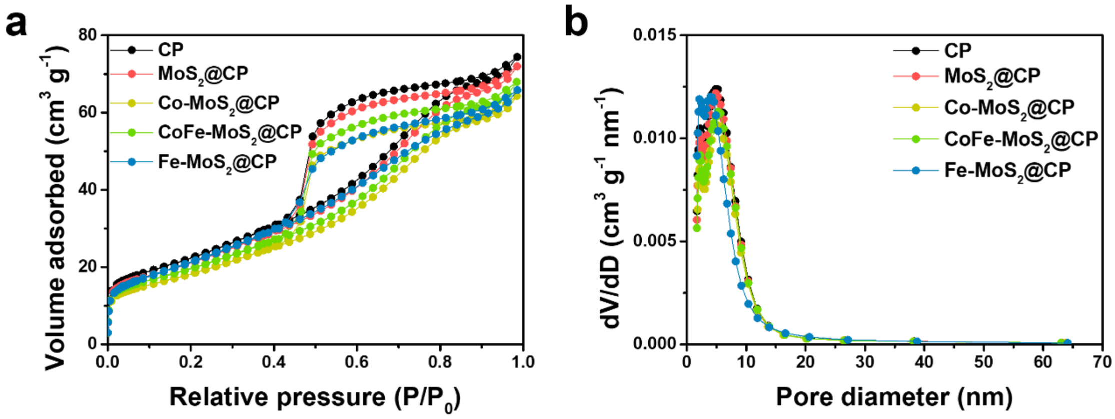

Figure S10. (a) N_2 adsorption/desorption curves and (b) corresponding pore size distributions of the CP, MoS_2@CP, Co-MoS_2@CP, CoFe-MoS_2@CP, and Fe-MoS_2@CP.

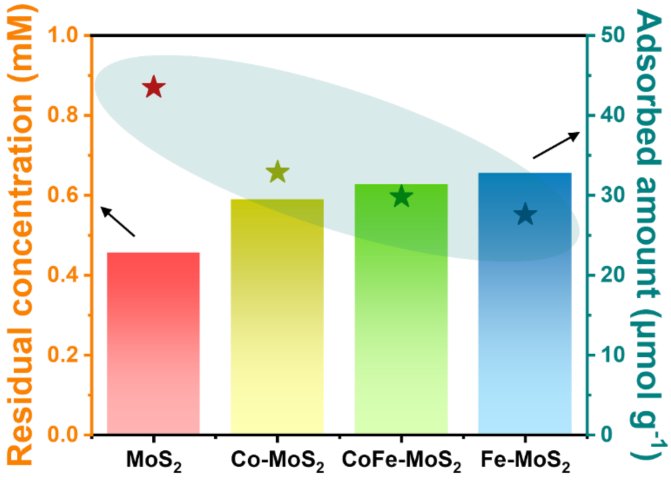

Figure S11. Quantification of Li_2S_4 adsorption based on 430 nm wavelength. Residual Li_2S_4 concentrations and the corresponding adsorbed amounts for each solution, calculated based on the Beer-Lambert law following the adsorption test.

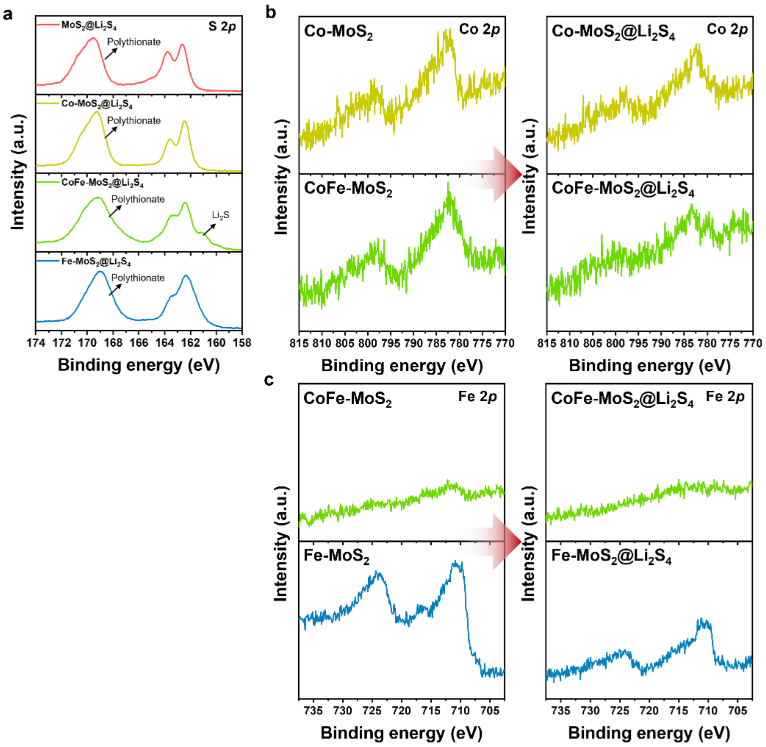

Figure S12. (a) Post-mortem XPS spectra of the MoS_2, Co-MoS_2, CoFe-MoS_2, and Fe-MoS_2 catalysts in the S 2*p* region after Li_2S_4 adsorption. Corresponding post-mortem XPS spectra for the (b) Co 2*p* and (c) Fe 2*p* regions.

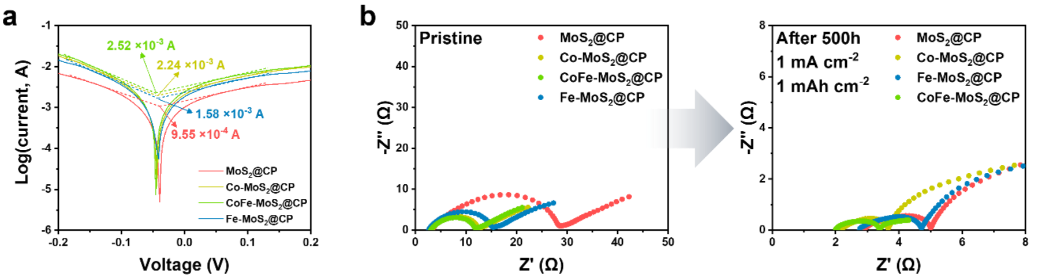

Figure S13. (a) Tafel plots evaluating the Li plating/stripping kinetics in Li||Li symmetric cells with MoS_2@CP, Co-MoS_2@CP, CoFe-MoS_2@CP, and Fe-MoS_2@CP interlayers. (b) EIS Nyquist plots of the symmetric cells at the pristine state and after 500 hours of cycling.

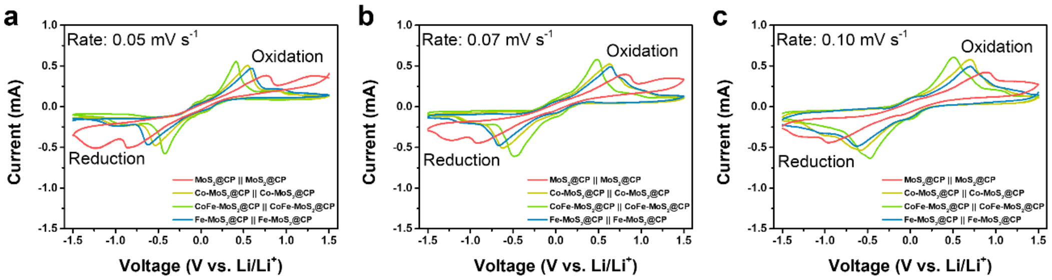

Figure S14. Collected CV curves of symmetric cells containing the MoS_2@CP, Co-MoS_2@CP, CoFe-MoS_2@CP, and Fe-MoS_2@CP interlayers at scan rates of (a) 0.05 mV s^{-1}, (b) 0.07 mV s^{-1}, and (c) 0.10 mV s^{-1}.

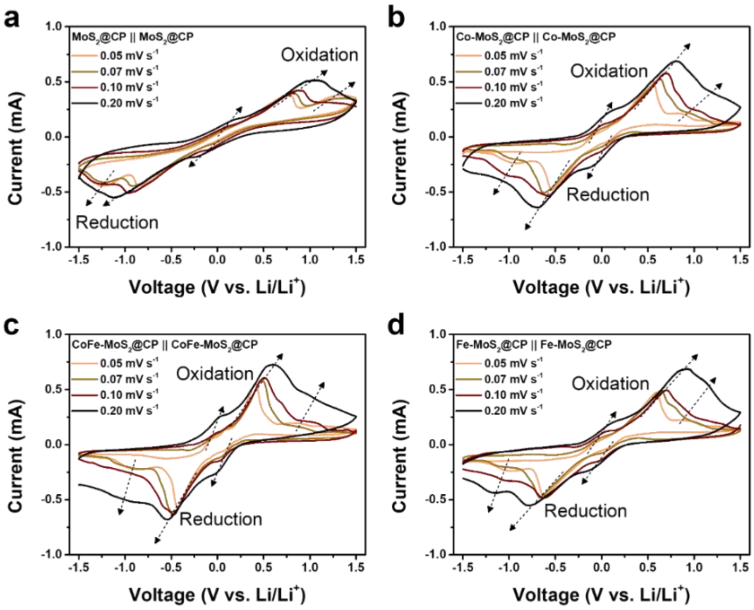

Figure S15. Entire CV profiles at scan rates of 0.05, 0.07, 0.10, and 0.20 mV s^{-1} for symmetric cells containing the (a) MoS_2@CP, (b) Co-MoS_2@CP, (c) CoFe-MoS_2@CP, and (d) Fe-MoS_2@CP interlayers.

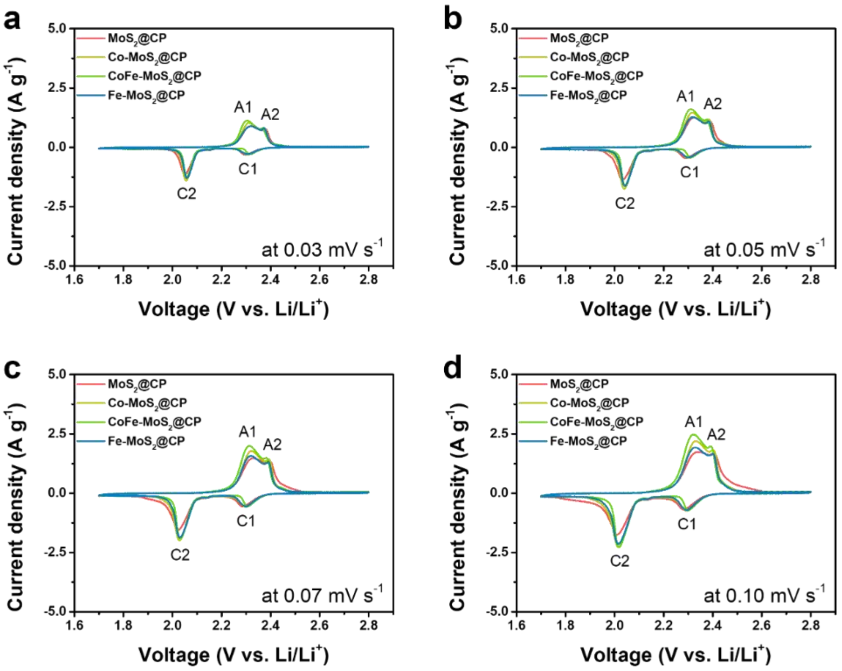

Figure S16. CV curves of asymmetric cells accompanied with the MoS_2@CP, Co-MoS_2@CP, CoFe-MoS_2@CP, and Fe-MoS_2@CP interlayers at various sweep rates of (a) 0.03 mV s^{-1}, (b) 0.05 mV s^{-1}, (c) 0.07 mV s^{-1}, and (d) 0.10 mV s^{-1}.

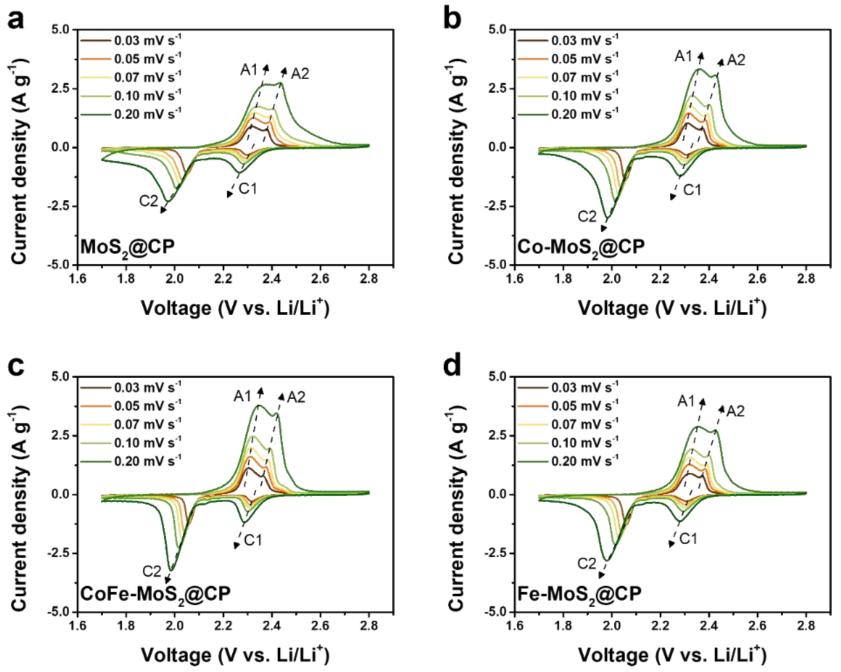

Figure S17. Entire CV profiles at scan rates of 0.03, 0.05, 0.07, 0.10, and 0.20 mV s^{-1} of the (a) MoS_2@CP, (b) Co-MoS_2@CP, (c) CoFe-MoS_2@CP, and (d) Fe-MoS_2@CP asymmetric cells.

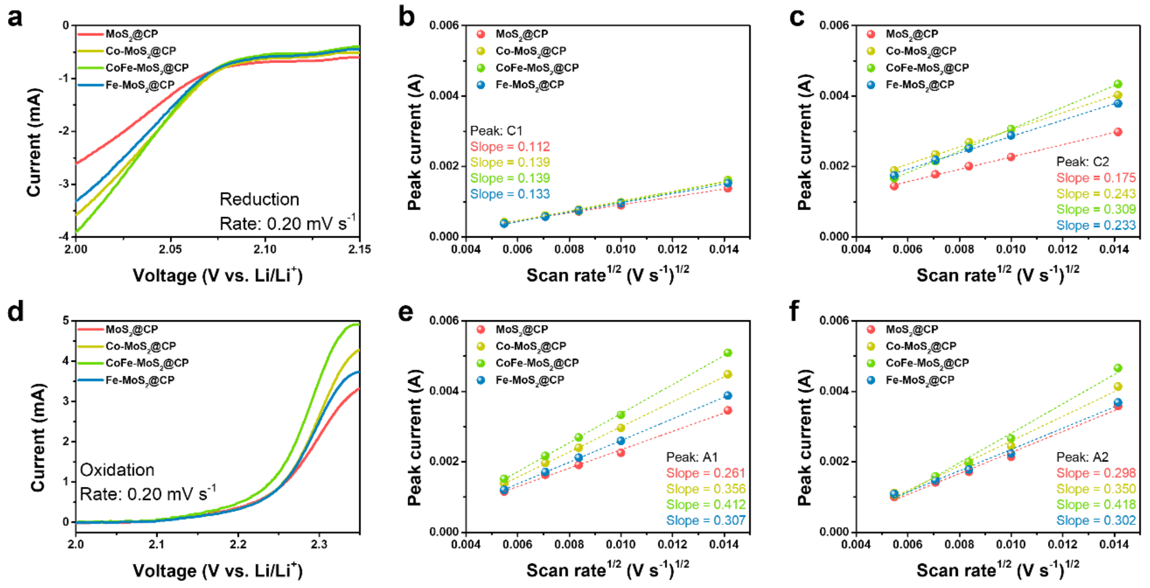

Figure S18. Reduction and oxidation processes of the Li-S cells showing (a,d) magnified view of CV spectra recorded at a scan rate of 0.20 mV s^{-1} and the fitted lines demonstrating the linear relationship for reaction peak currents versus the square root of the scan rate at (b) C1 and (c) C2, (e) A1, and (f) A2.

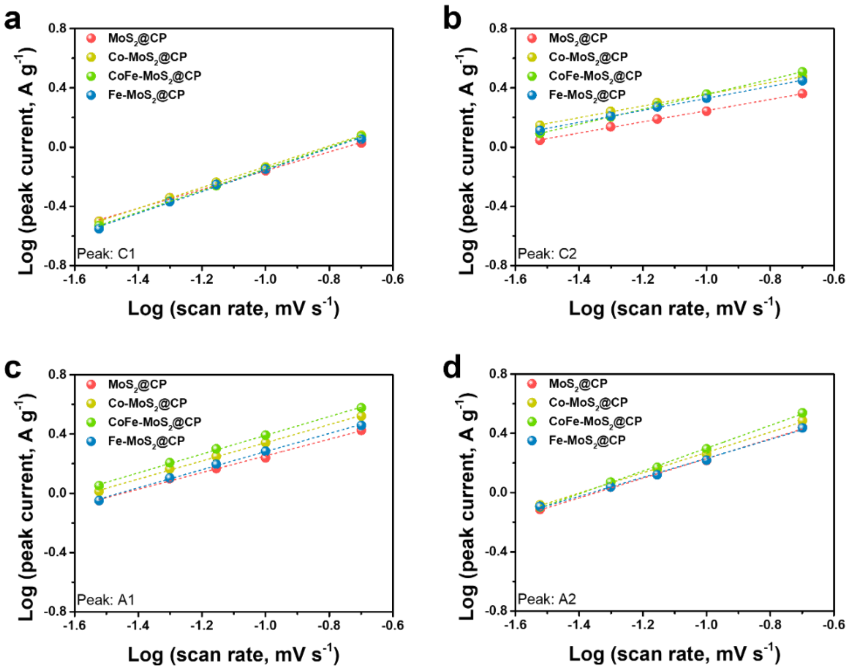

Figure S19. The fitted lines showing linear relationship between peak currents versus scan rate in log-log scale at the oxidation and reduction reactions for the cells positioned at (a) C1, (b) C2, (c) A1, and (d) A2 peaks.

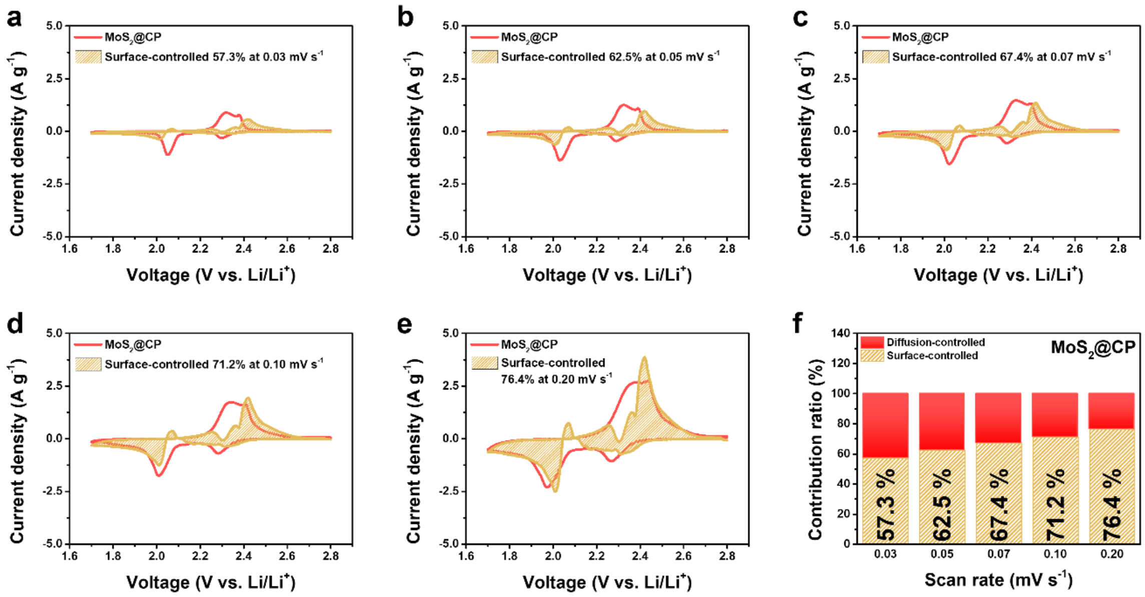

Figure S20. (a–e) CV profiles of the MoS_2@CP cell shown with surface-controlled contribution at various scan rates, and (f) bar graphs showing the corresponding contribution ratios between diffusion- and surface-controlled processes.

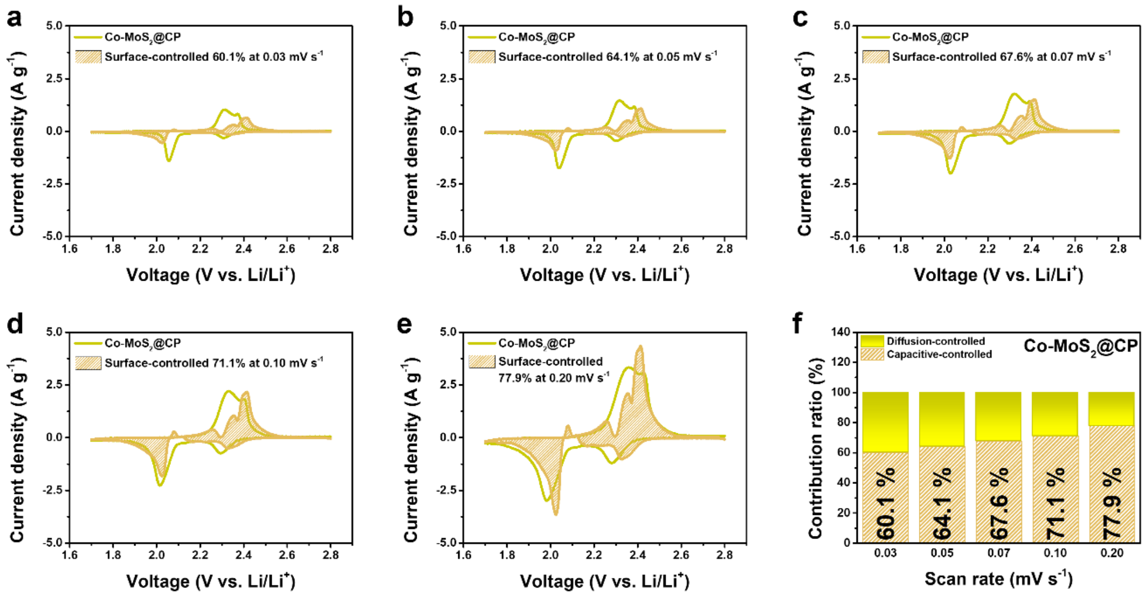

Figure S21. (a–e) CV profiles of the Co-MoS_2@CP cell shown with surface-controlled contribution at various scan rates, and (f) bar graphs showing the corresponding contribution ratios between diffusion- and surface-controlled processes.

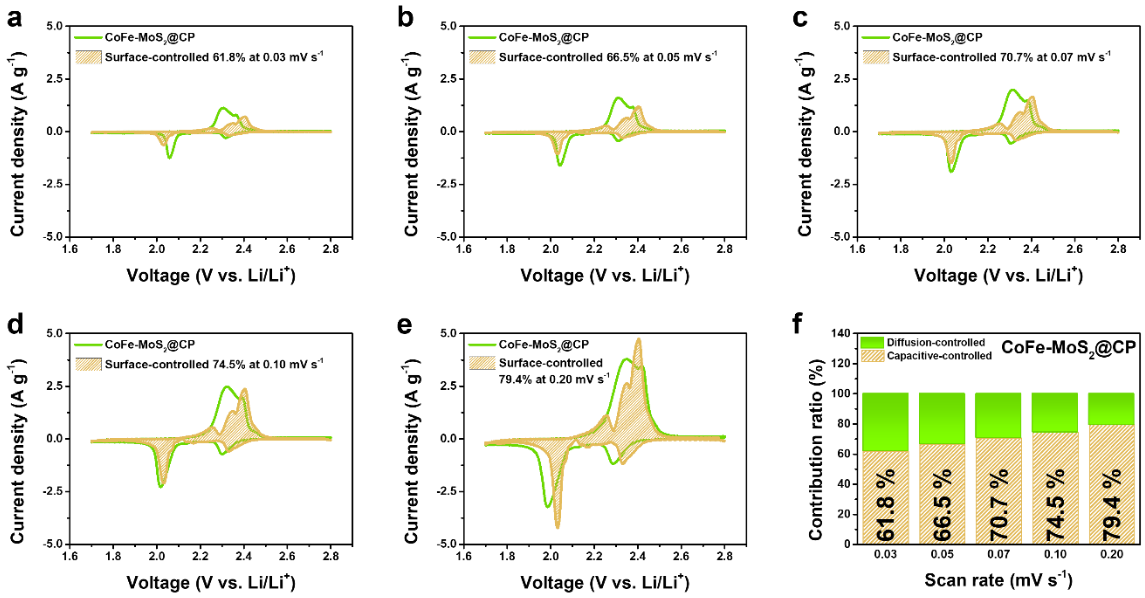

Figure S22. (a–e) CV profiles of the CoFe-MoS2@CP cell shown with surface-controlled contribution at various scan rates, and (f) bar graphs showing the corresponding contribution ratios between diffusion- and surface-controlled processes.

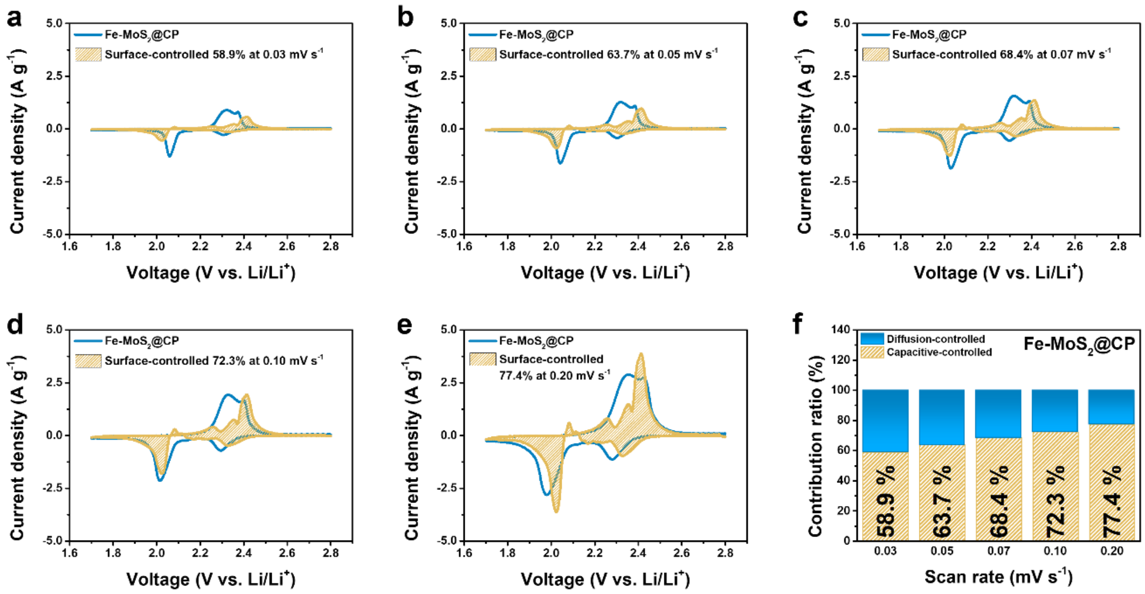

Figure S23. (a–e) CV profiles of the Fe-MoS2@CP cell shown with surface-controlled contribution at various scan rates, and (f) bar graphs showing the corresponding contribution ratios between diffusion- and surface-controlled processes.

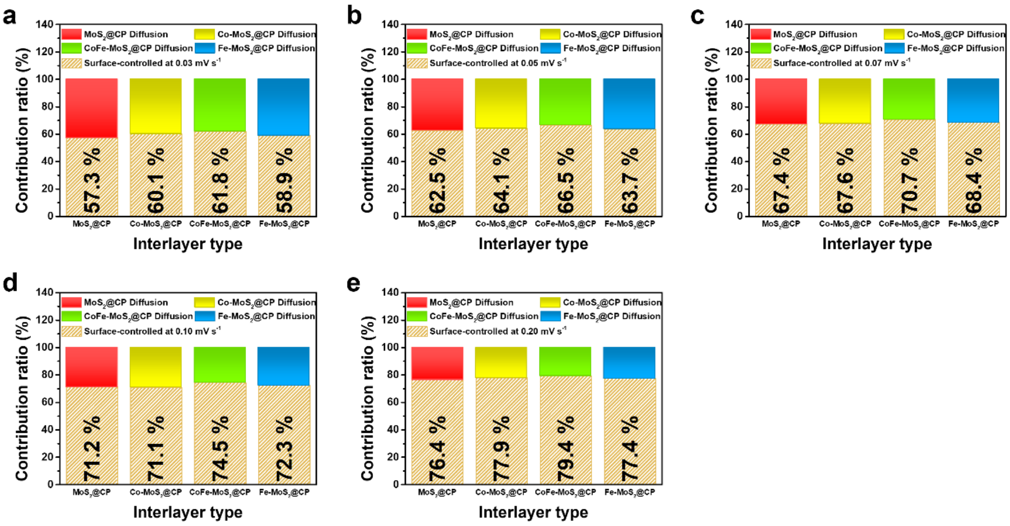

Figure S24. Bar graphs showing surface-controlled contribution ratios at various scan rates for the cells at the scan rates of (a) 0.03 mV s^{-1}, (b) 0.05 mV s^{-1}, (c) 0.07 mV s^{-1}, (d) 0.10 mV s^{-1}, and (e) 0.20 mV s^{-1}.

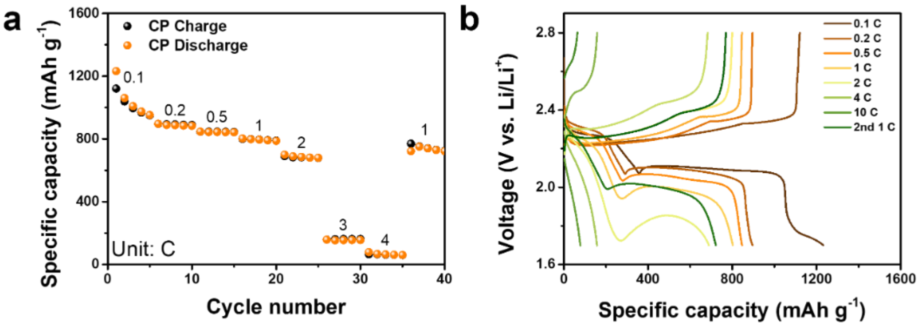

Figure S25. (a) Rate capability performance during discharging process of the bare CP cell with (b) corresponding galvanostatic charge-discharge profiles.

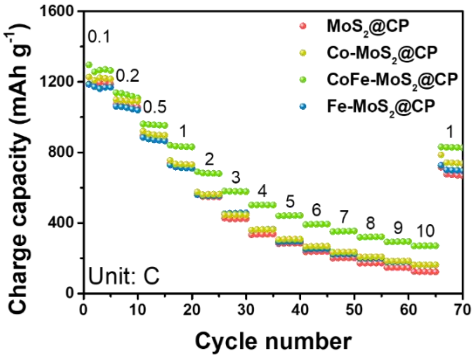

Figure S26. (a) Rate capability performance during charging process of the MoS2@CP, Co-MoS2@CP, CoFe-MoS2@CP, and Fe-MoS2@CP cells.

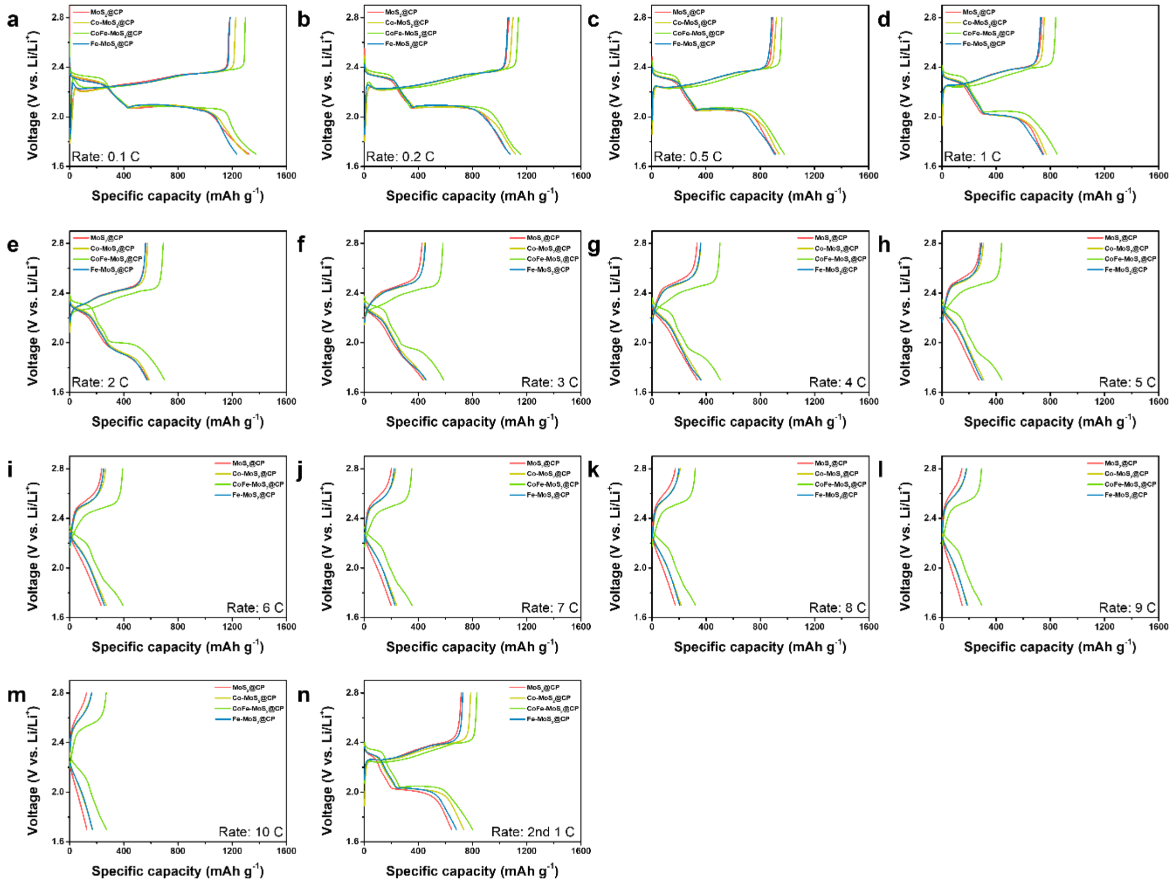

Figure S27. Galvanostatic charge-discharge profiles at different current rates of (a) 0.1 C, (b) 0.2 C, (c) 0.5 C, (d) 1 C, (e) 2 C, (f) 3 C, (g) 4 C, (h) 5 C, (i) 6 C, (j) 7 C, (k) 8 C, (l) 9 C, (m) 10 C, and (n) 2nd 1 C for the MoS_2@CP, Co-MoS_2@CP, CoFe-MoS_2@CP, and Fe-MoS_2@CP cells.

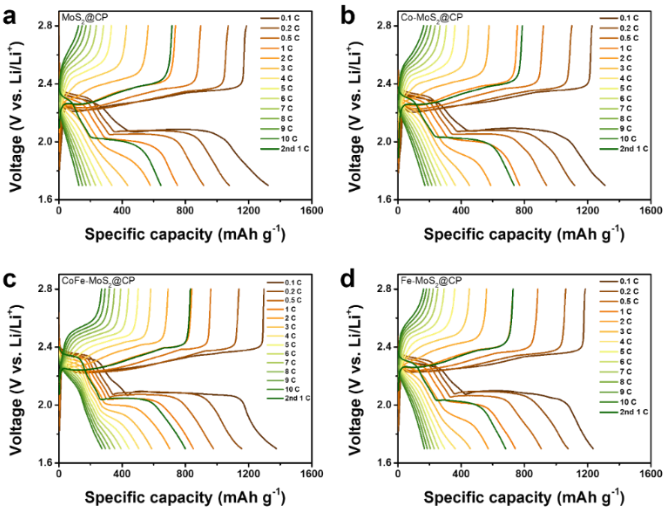

Figure S28. Galvanostatic charge-discharge profiles of the (a) MoS_2@CP, (b) Co-MoS_2@CP, (c) CoFe-MoS_2@CP, and (d) Fe-MoS_2@CP cells during the rate capability test.

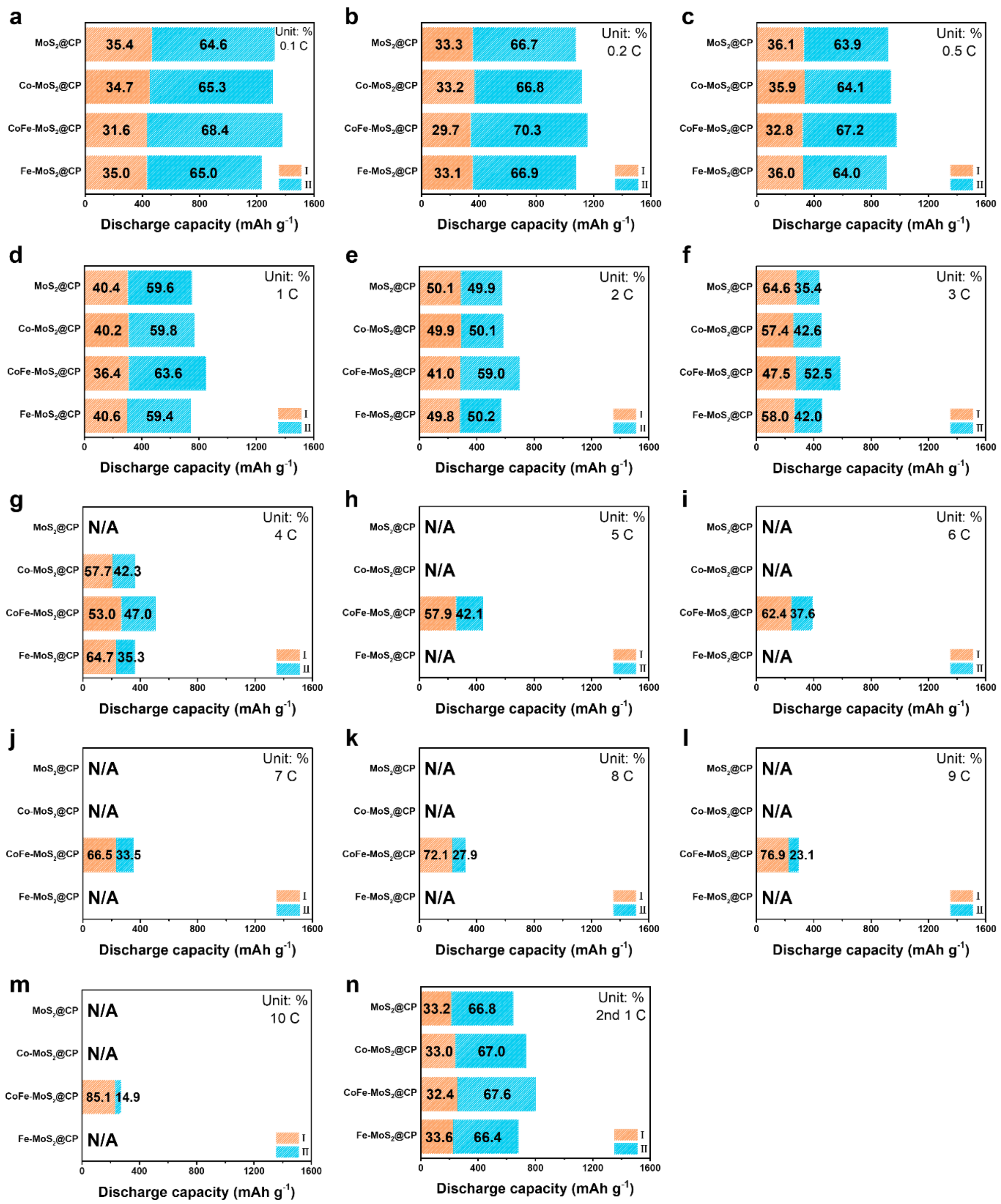

Figure S29. Discharge capacity ratios calculated from extents of the first plateau (denoted as I) and second plateau (denoted as II) at different current rates of (a) 0.1 C, (b) 0.2 C, (c) 0.5 C, (d) 1 C, (e) 2 C, (f) 3 C, (g) 4 C, (h) 5 C, (i) 6 C, (j) 7 C, (k) 8 C, (l) 9 C, (m) 10 C, and (n) 2nd 1 C for the cells.

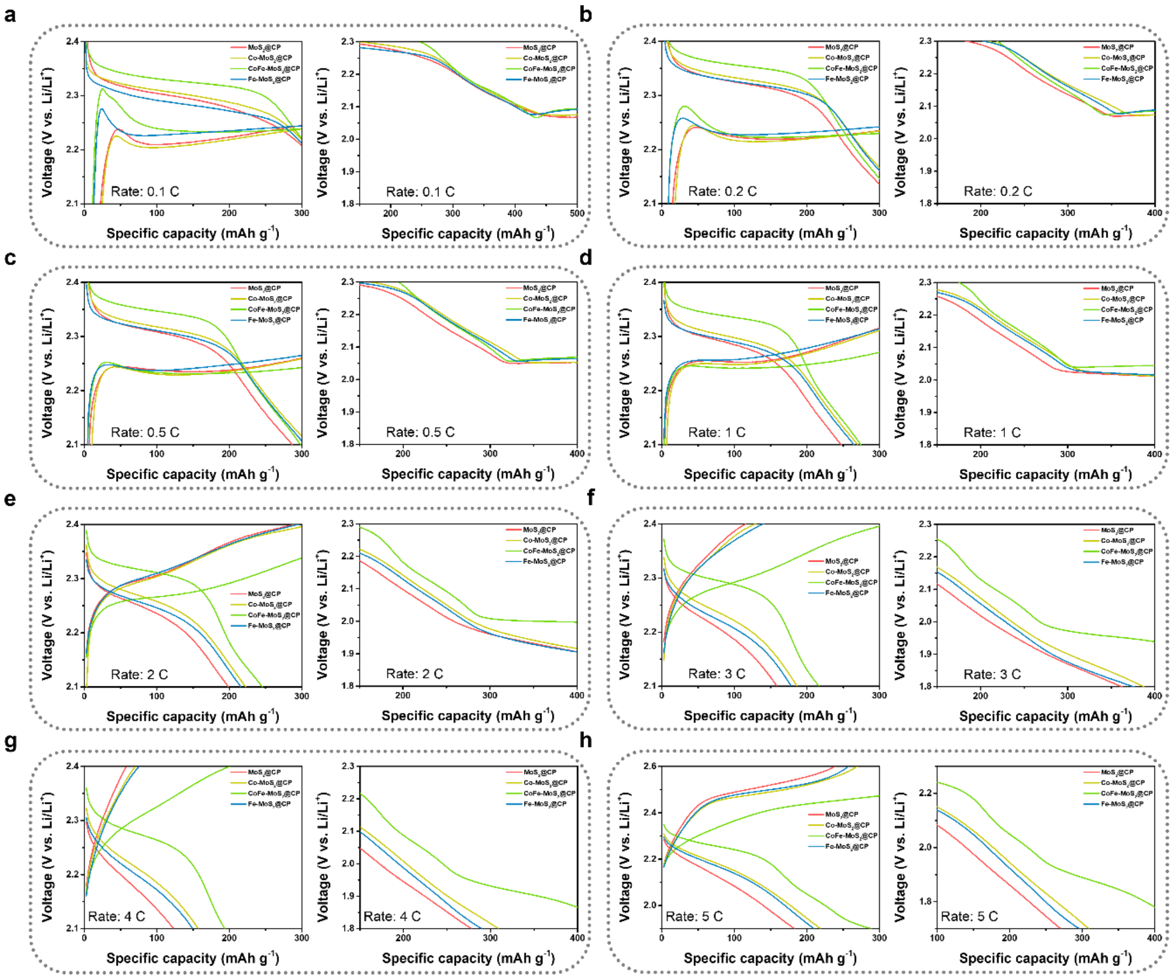

Figure S30. Galvanostatic charge-discharge profiles with enlarged areas of the first and second discharge plateaus, and beginning part of charge of the cells at the scan rates of (a) 0.1 C, (b) 0.2 C, (c) 0.5 C, (d) 1 C, (e) 2 C, (f) 3 C, (g) 4 C, and (h) 5 C.

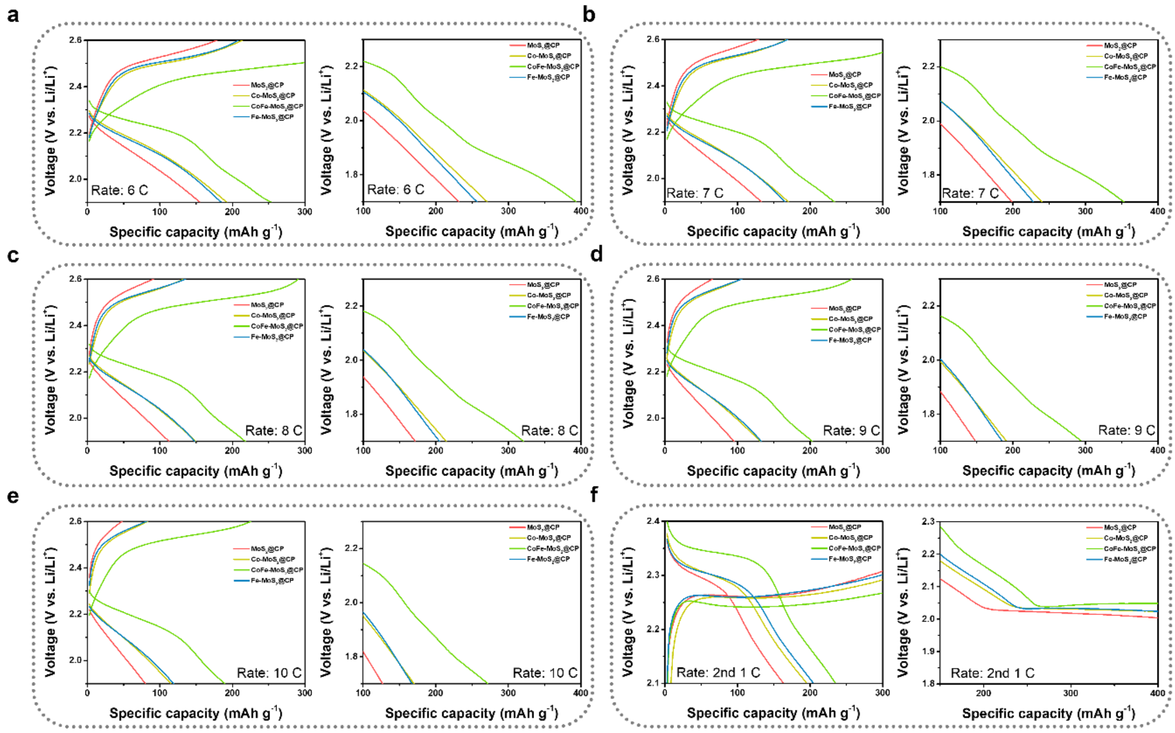

Figure S31. Galvanostatic charge-discharge profiles with enlarged areas of the first and second discharge plateaus, and beginning part of charge of the cells at the scan rates of (a) 6 C, (b) 7 C, (c) 8 C, (d) 9 C, (e) 10 C, and (f) 2nd 1 C.

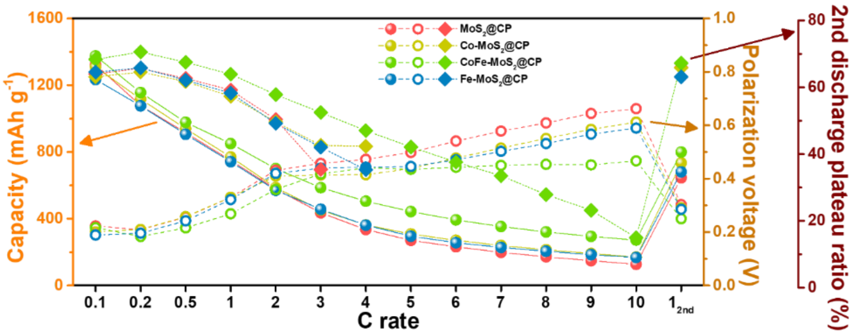

Figure S32. Overall comparison of rate capability performance showing discharge capacity, polarization voltage at DoD 50%, and 2nd discharge plateau ratio for the MoS_2@CP, Co-MoS_2@CP, CoFe-MoS_2@CP, and Fe-MoS_2@CP cells.

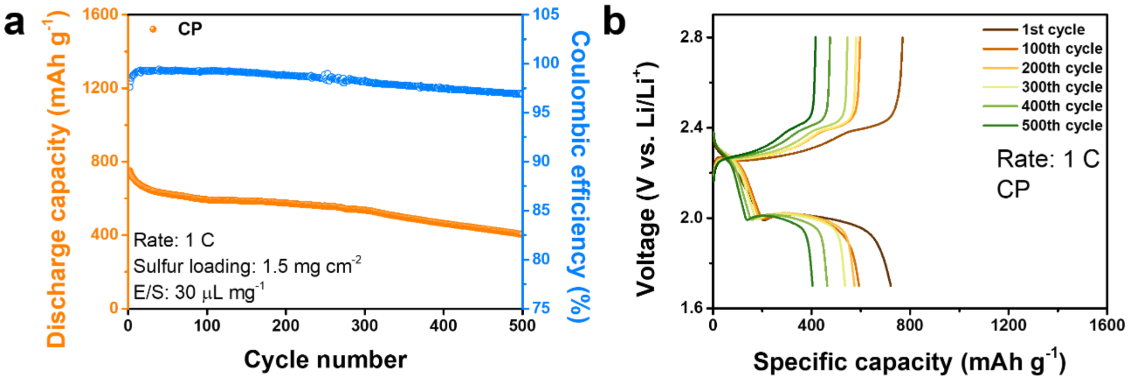

Figure S33. (a) Cycling performance of the bare CP cell up to 500 cycles under conditions as rate of 1 C, sulfur loading of 1.5 mg cm^{-2}, and E/S ratio of 30 μL mg^{-1} and (b) corresponding galvanostatic charge-discharge profiles at 1st, 100th, 200th, 300th, 400th, and 500th cycle.

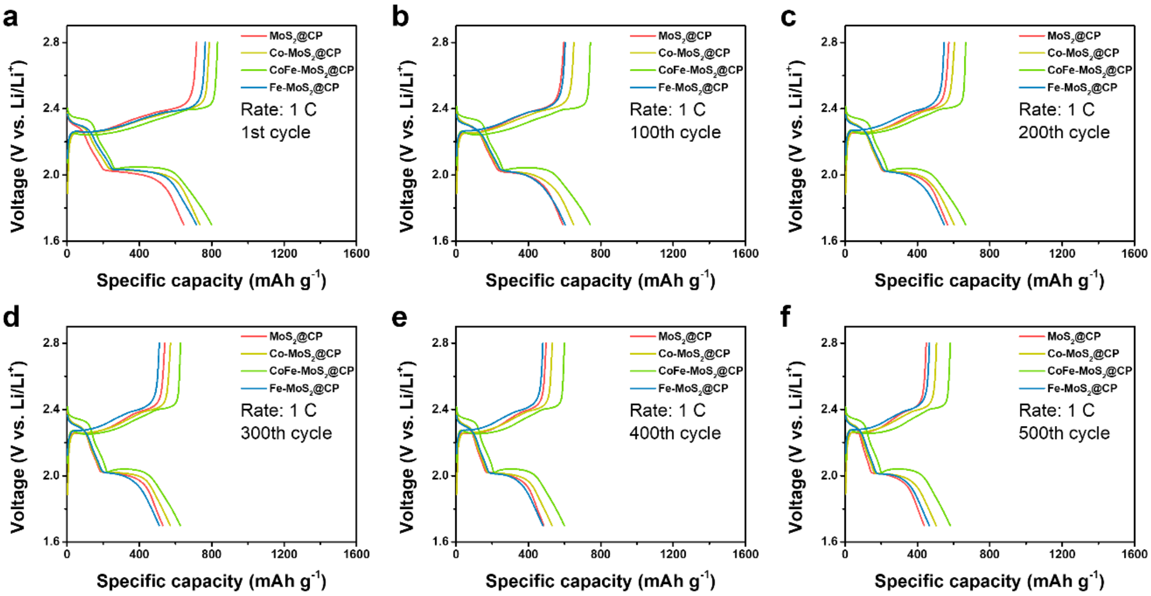

Figure S34. Galvanostatic charge-discharge profiles of the MoS_2@CP, Co-MoS_2@CP, CoFe-MoS_2@CP, and Fe-MoS_2@CP cells during cycling process under 1 C rate condition at (a) 1st, (b) 100th, (c) 200th, (d) 300th, (e) 400th, and (f) 500th cycle.

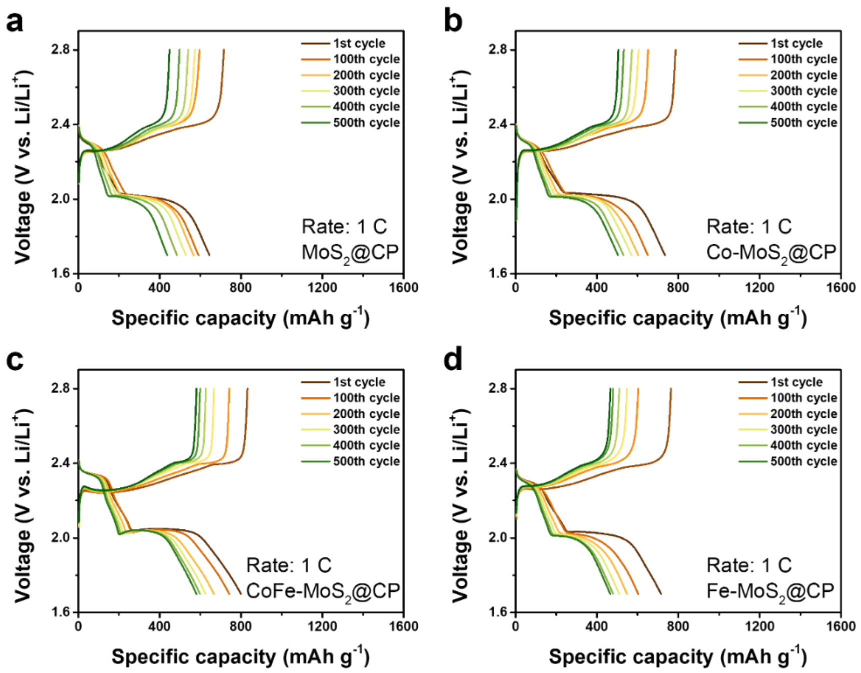

Figure S35. Galvanostatic charge-discharge profiles of the (a) MoS_2@CP, (b) Co-MoS_2@CP, (c) CoFe-MoS_2@CP, and (d) Fe-MoS_2@CP cells during cycling process under 1 C rate condition at 1st, 100th, 200th, 300th, 400th, and 500th cycle.

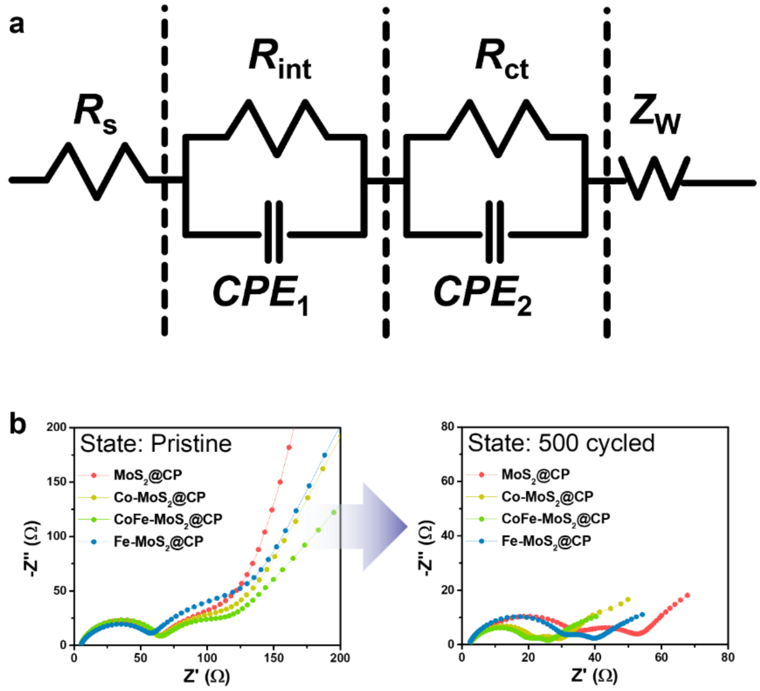

Figure S36. (a) Equivalent circuit diagrams for EIS analysis of the cells. (b) Ex-situ EIS analysis of the cells carried out at pristine state and 500-cycled state, with cycling condition at 1 C rate.

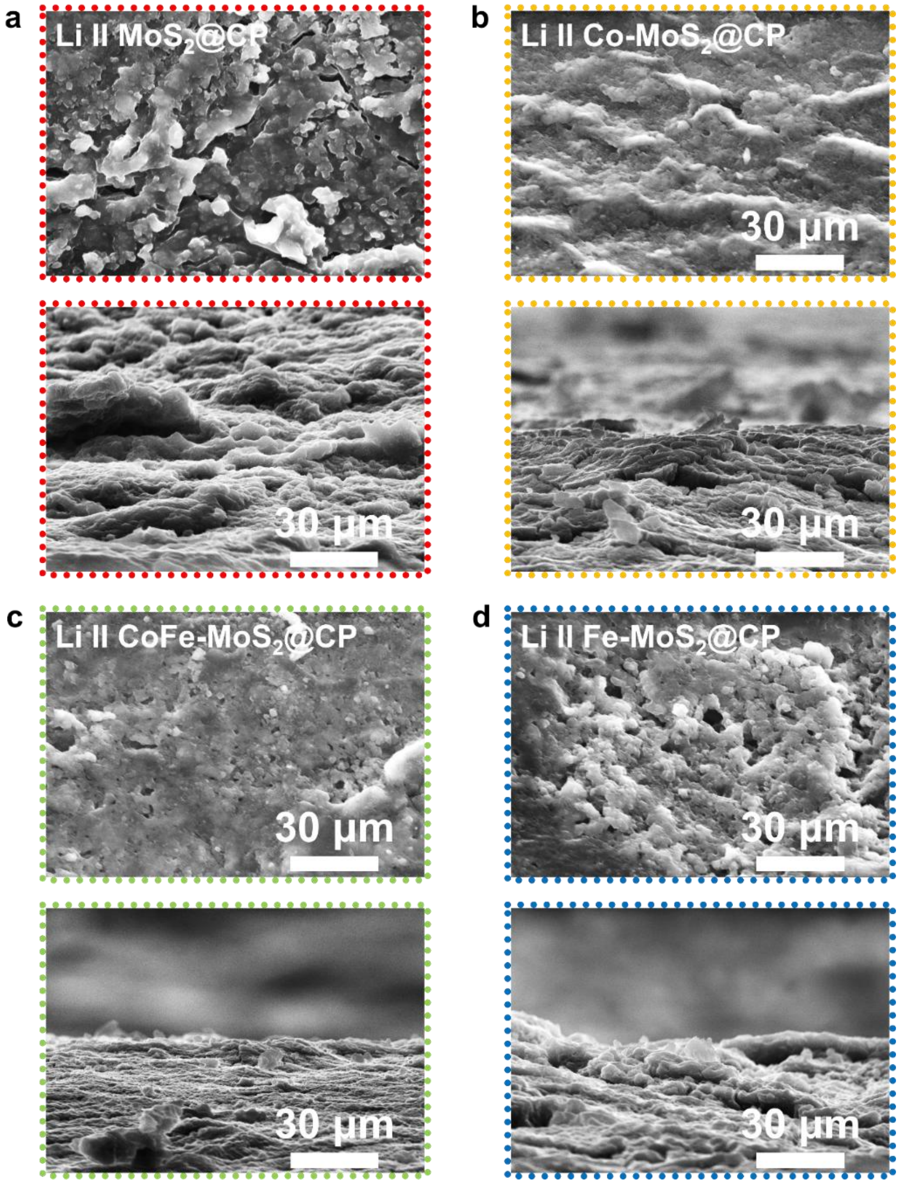

Figure S37. Post-mortem analysis of the Li anodes via observation of ex-situ SEM images as top-view and cross-sectional view in the (a) MoS_2@CP, (b) Co-MoS_2@CP, (c) CoFe-MoS_2@CP, and (d) Fe-MoS_2@CP cells after 500 cycles at 1 C rate.

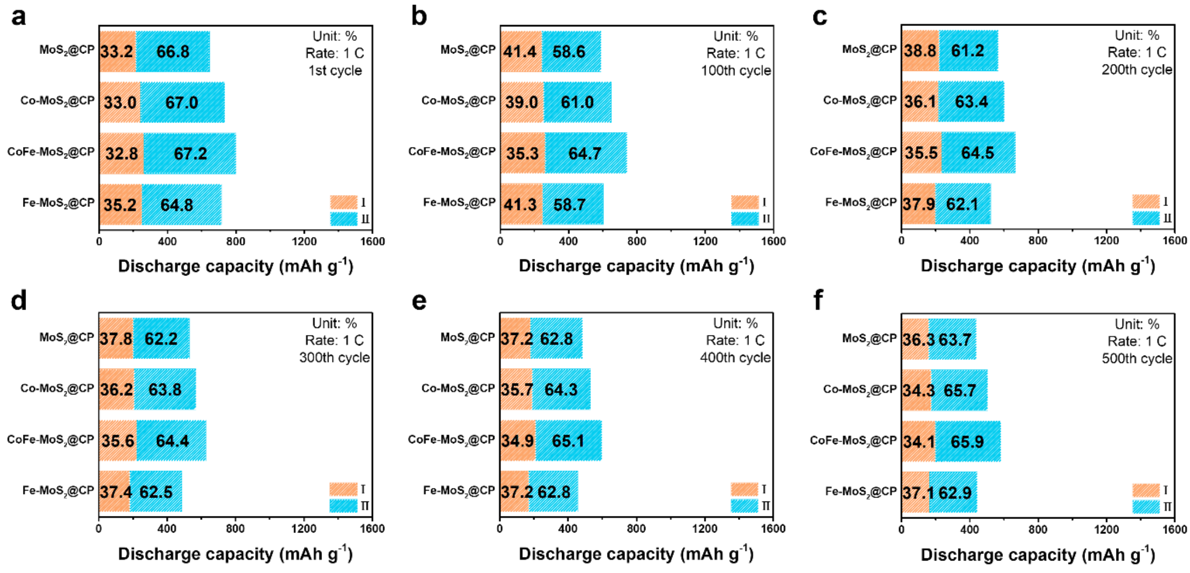

Figure S38. Discharge capacity ratios calculated from extents of the first plateau (denoted as I) and second plateau (denoted as II) during cycling process under 1 C rate condition for the cells at (a) 1st, (b) 100th, (c) 200th, (d) 300th, (e) 400th, and (f) 500th cycle.

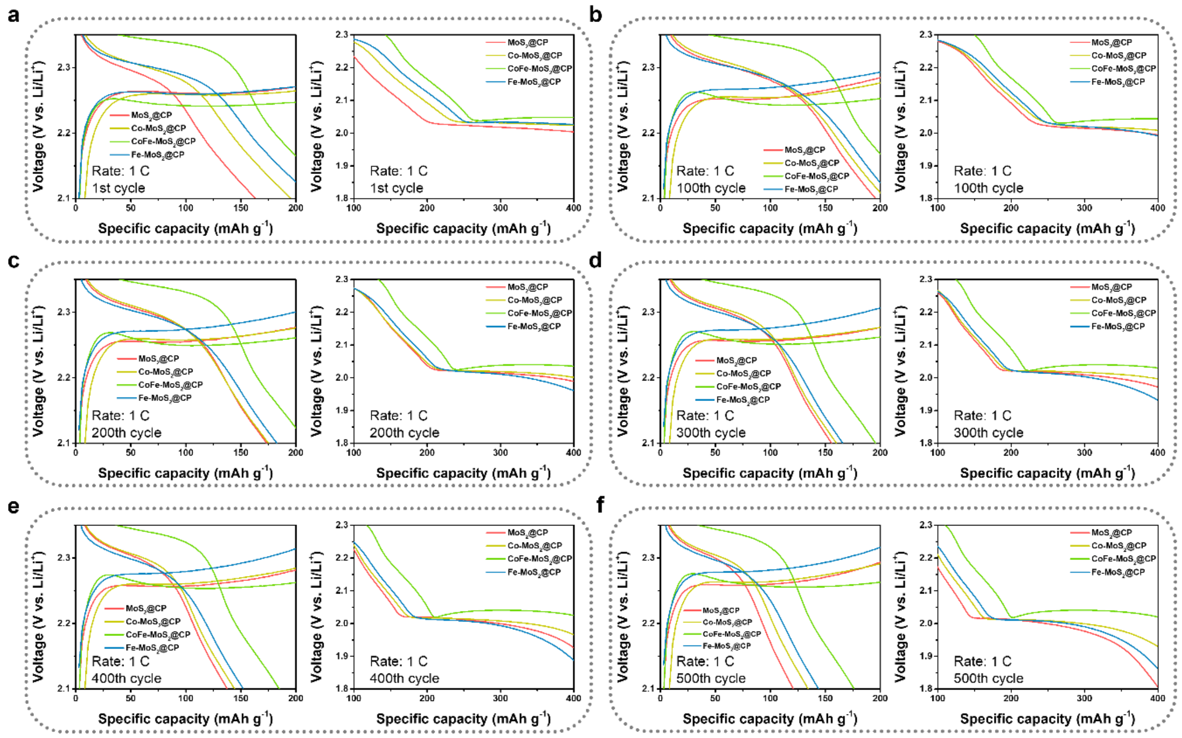

Figure S39. Galvanostatic charge-discharge profiles with enlarged areas of the first and second discharge plateaus, and beginning part of charge of the cells during cycling process under 1 C rate condition for the cells at (a) 1st, (b) 100th, (c) 200th, (d) 300th, (e) 400th, and (f) 500th cycle.

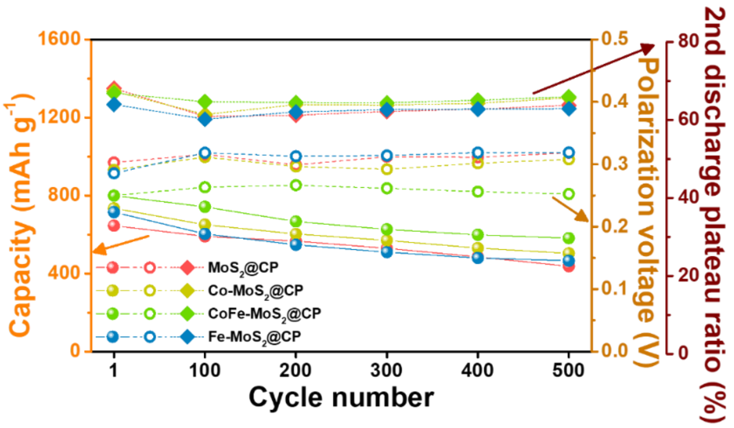

Figure S40. Overall comparison of long-term cycling performance at 1 C rate showing discharge capacity, polarization voltage at DoD 50%, and 2nd discharge plateau ratio for the MoS_2@CP, Co-MoS_2@CP, CoFe-MoS_2@CP, and Fe-MoS_2@CP cells.

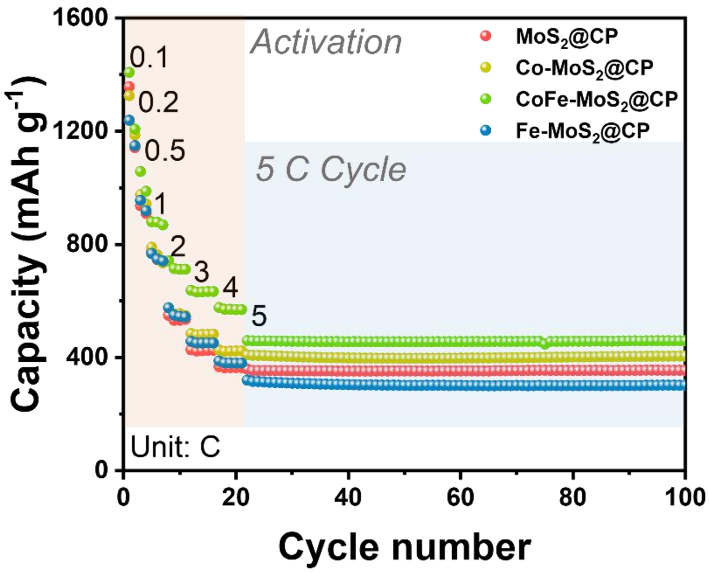

Figure S41. Activation cycle process for the MoS_2@CP, Co-MoS_2@CP, CoFe-MoS_2@CP, and Fe-MoS_2@CP cells prior to evaluating their long-term cycling performance at 5 C.

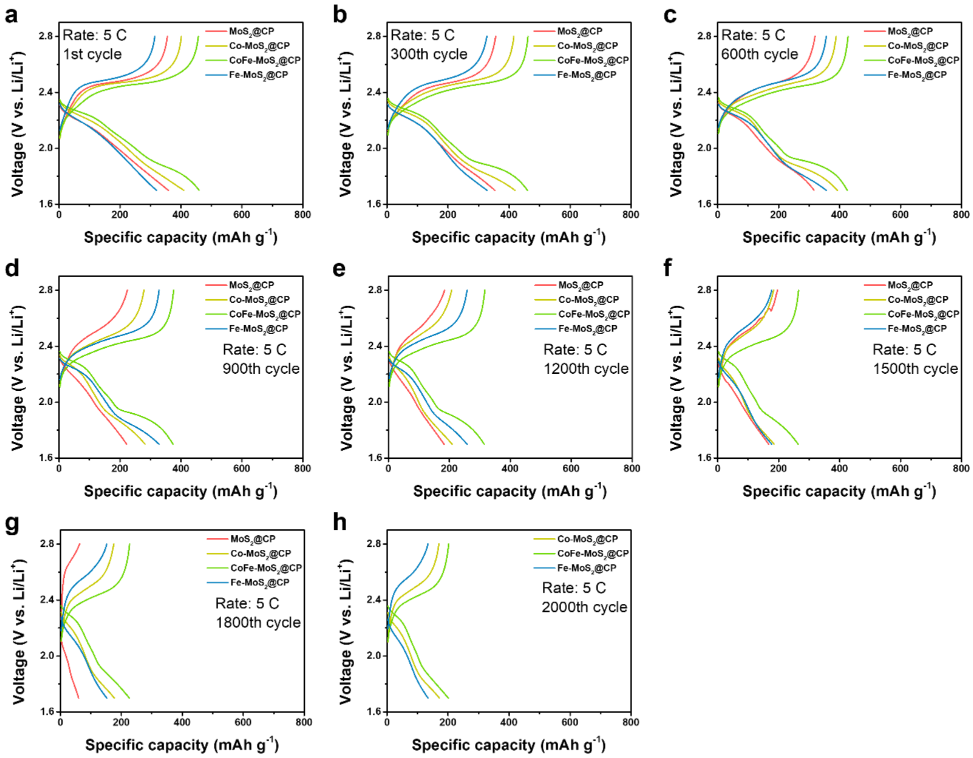

Figure S42. Galvanostatic charge-discharge profiles of the MoS_2@CP, Co-MoS_2@CP, CoFe-MoS_2@CP, and Fe-MoS_2@CP cells during cycling process under 5 C rate condition at (a) 1st, (b) 300th, (c) 600th, (d) 900th, (e) 1200th, and (f) 1500th, (g) 1800th, and (h) 2000th cycle.

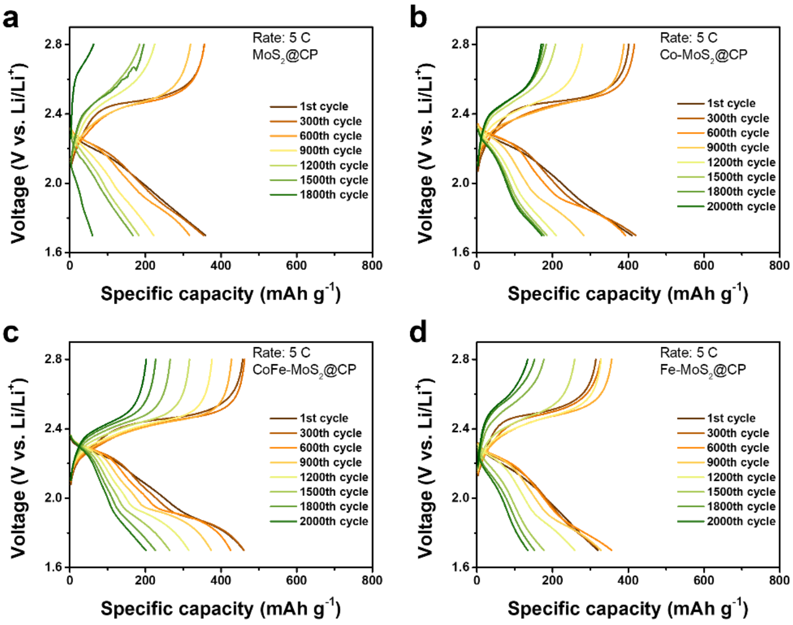

Figure S43. Galvanostatic charge-discharge profiles of the (a) MoS_2@CP, (b) Co-MoS_2@CP, (c) CoFe-MoS_2@CP, and (d) Fe-MoS_2@CP cells during cycling process under 5 C rate condition at 1st, 300th, 600th, 900th, 1200th, and 1500th, 1800th, and 2000th cycle.

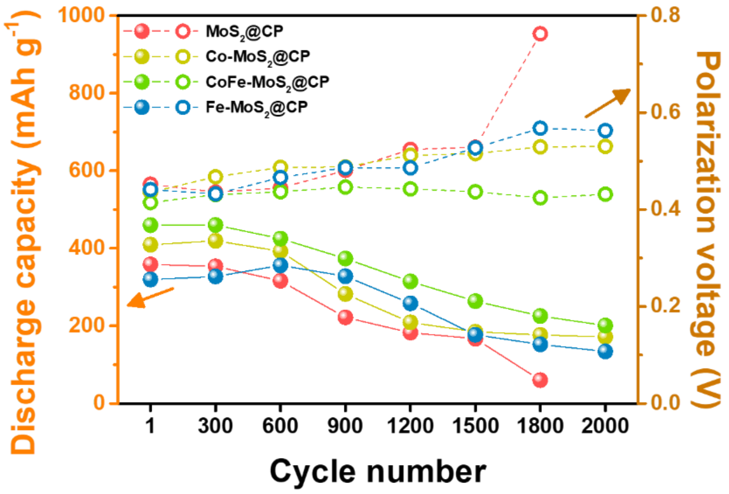

Figure S44. Overall comparison of long-term cycling performance at 5 C rate showing discharge capacity, polarization voltage at DoD 50%, and 2nd discharge plateau ratio for the MoS2@CP, Co-MoS2@CP, CoFe-MoS2@CP, and Fe-MoS2@CP cells.

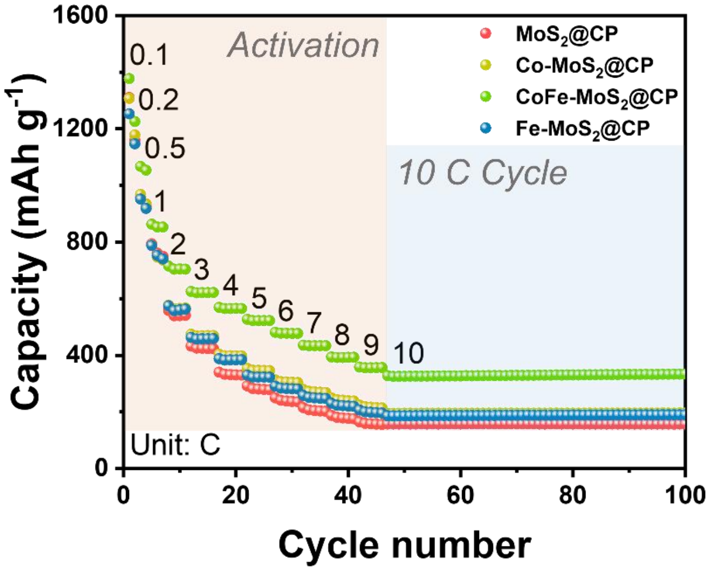

Figure S45. Activation cycle process for the MoS2@CP, Co-MoS2@CP, CoFe-MoS2@CP, and Fe-MoS2@CP cells prior to evaluating their long-term cycling performance at 10 C.

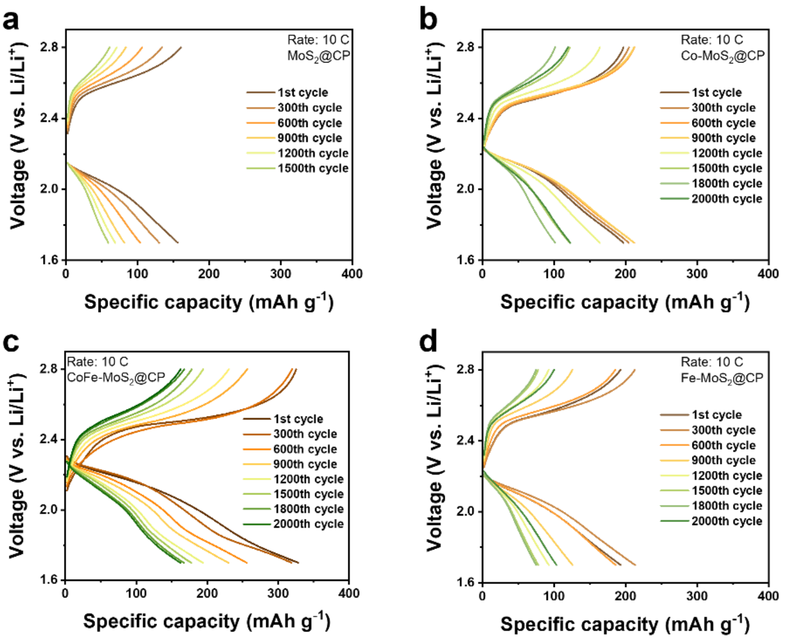

Figure S46. Galvanostatic charge-discharge profiles of the (a) MoS_2@CP, (b) Co-MoS_2@CP, (c) CoFe-MoS_2@CP, and (d) Fe-MoS_2@CP cells during cycling process under 10 C rate condition at 1st, 300th, 600th, 900th, 1200th, and 1500th, 1800th, and 2000th cycle.

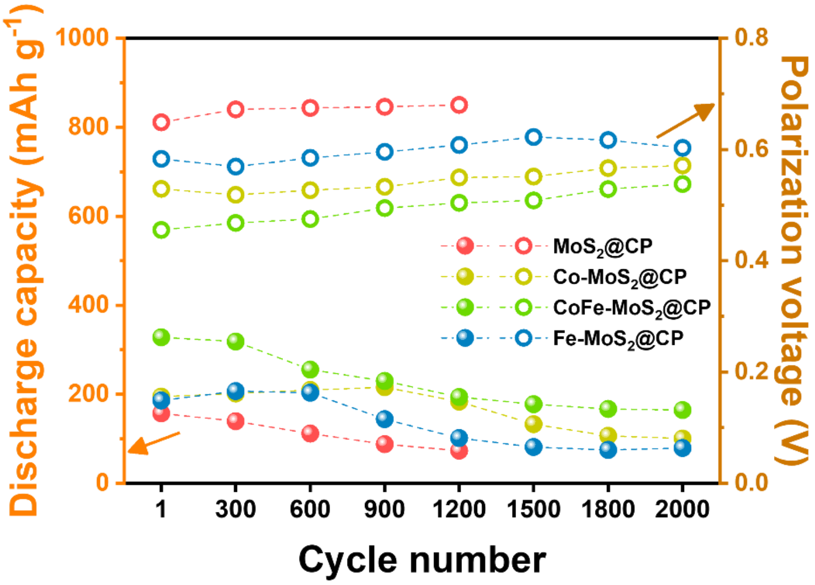

Figure S47. Overall long-term cycling performance at 10 C rate showing discharge capacity, polarization voltage at DoD 50%, and 2nd discharge plateau ratio for the cells.

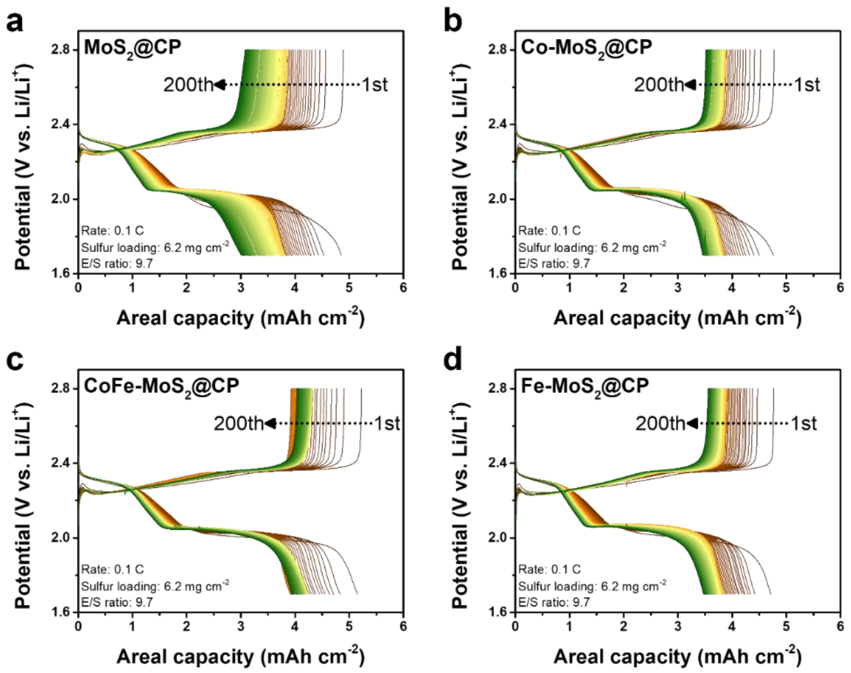

Figure S48. Galvanostatic charge-discharge profiles of the (a) MoS_2@CP, (b) Co-MoS_2@CP, (c) CoFe-MoS_2@CP, and (d) Fe-MoS_2@CP cells during cycling process for 200 cycles at 0.1 C rate under high sulfur loading and low E/S ratio (S: 6.2 mg cm^{-2}, E/S: 9.7 μL mg^{-1}) conditions.

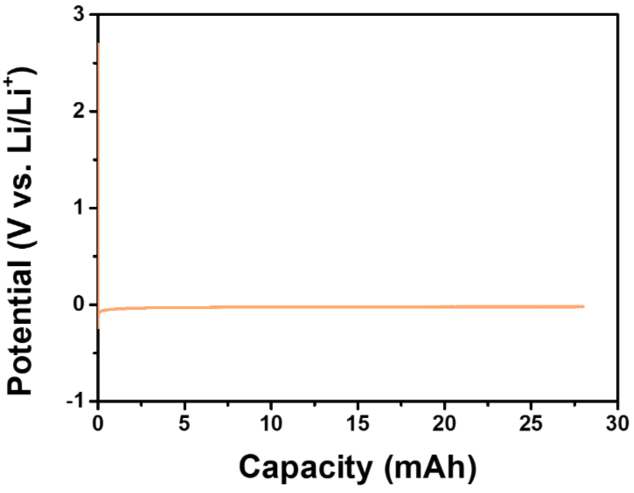

Figure S49. Electrochemical Li deposition profile of Li∥Cu cell in anode preparation of Li-S full-cell under condition of 1 mA current to acquire total about 28 mAh capacity, resulting in N/P ratio of 2.2.

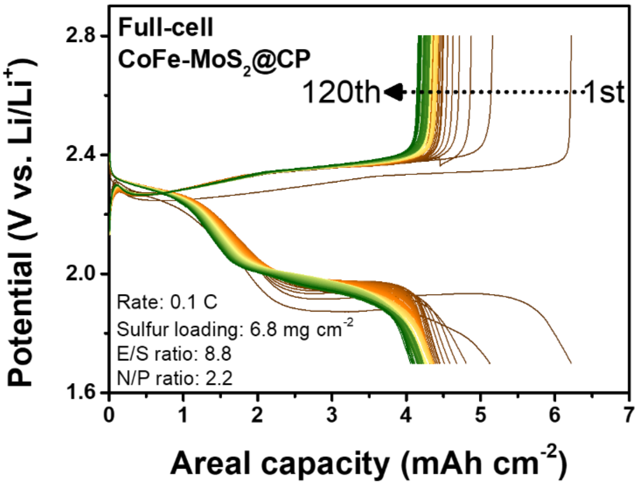

Figure S50. Galvanostatic charge-discharge profiles of the Li-S full-cell equipped with the CoFe-MoS_2@CP interlayer during cycling process for 120 cycles at 0.1 C rate under high sulfur loading and low E/S ratio (S: 6.8 mg cm^{-2}, E/S: 8.8 μL mg^{-1}) conditions.

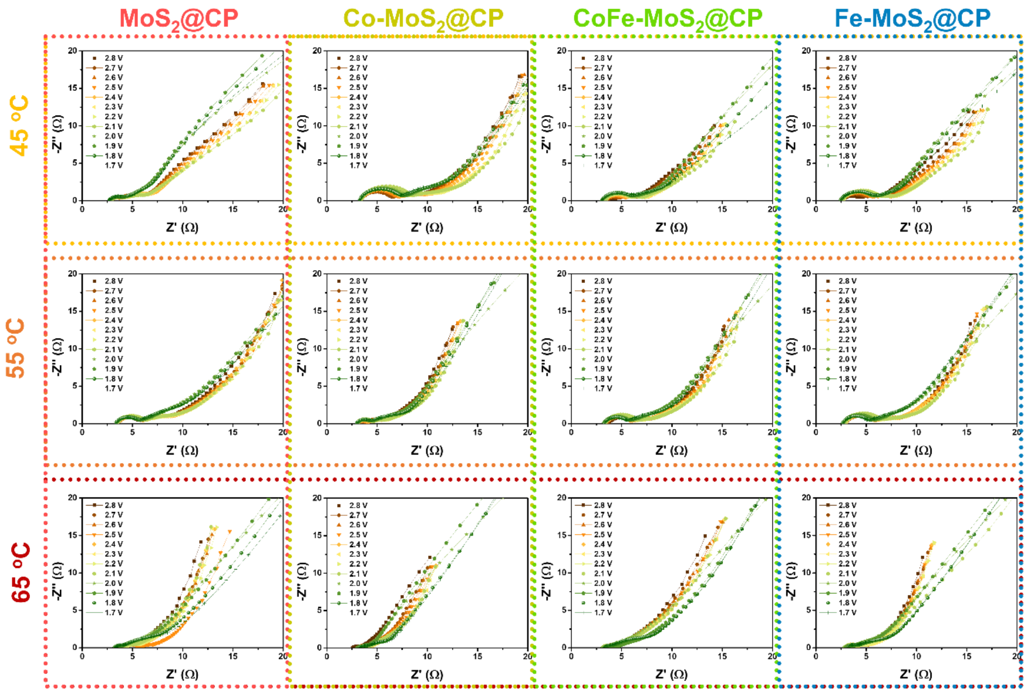

Figure S51. In-situ EIS measurements of the cells at temperatures of 45, 55, and 65 °C, recorded at 0.1 V intervals during discharge from 2.8 V (vs. Li/Li^+) to 1.7 V (vs. Li/Li^+).

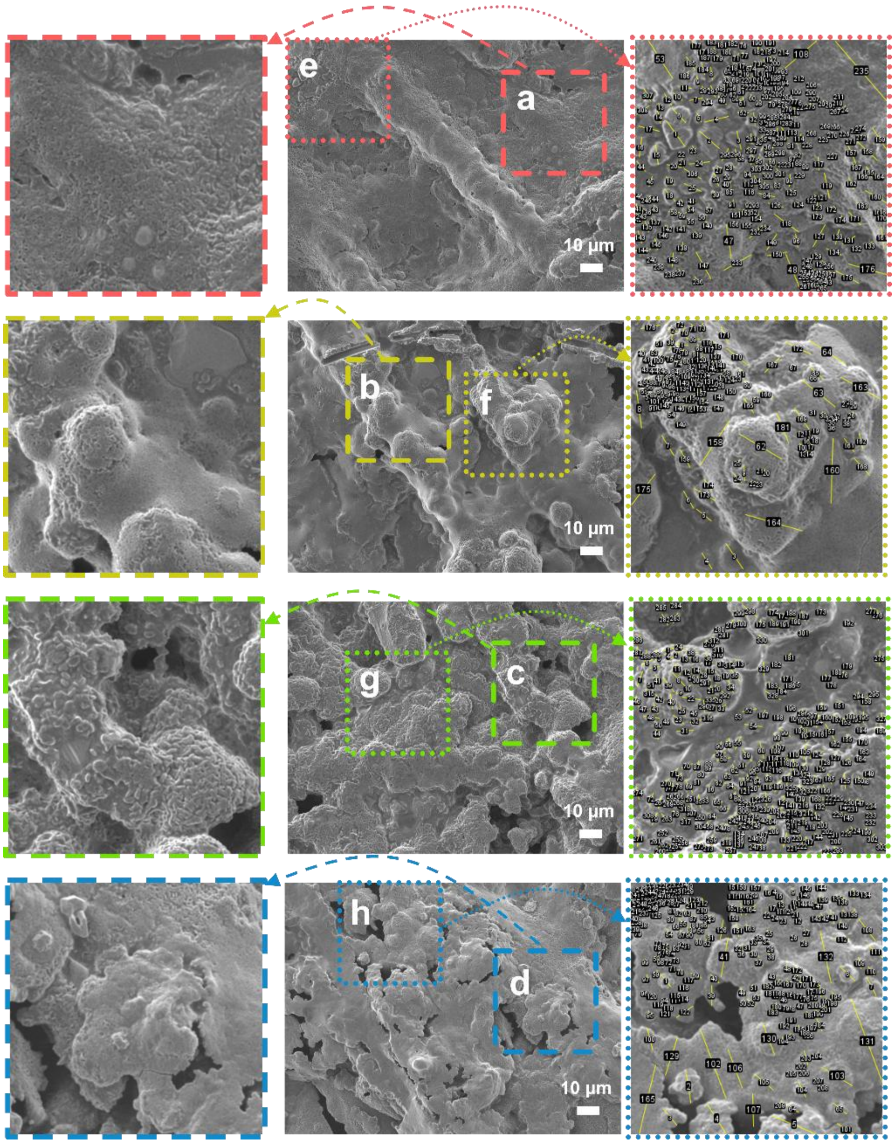

Figure S52. Ex situ SEM images visualizing the morphology of Li_2S deposited on the cathode surfaces after the potentiostatic discharge process of a Li_2S_6 electrolyte solution at 2.03 V (vs. Li/Li^+) for the (a,e) MoS_2@CP, (b,f) Co-MoS_2@CP, (c,g) CoFe-MoS_2@CP, and (d,h) Fe-MoS_2@CP cells. (e–h) Specifically, regions enabling the clear identification of distinct nuclei were selected from each image to extract statistical data on the Li_2S morphology.

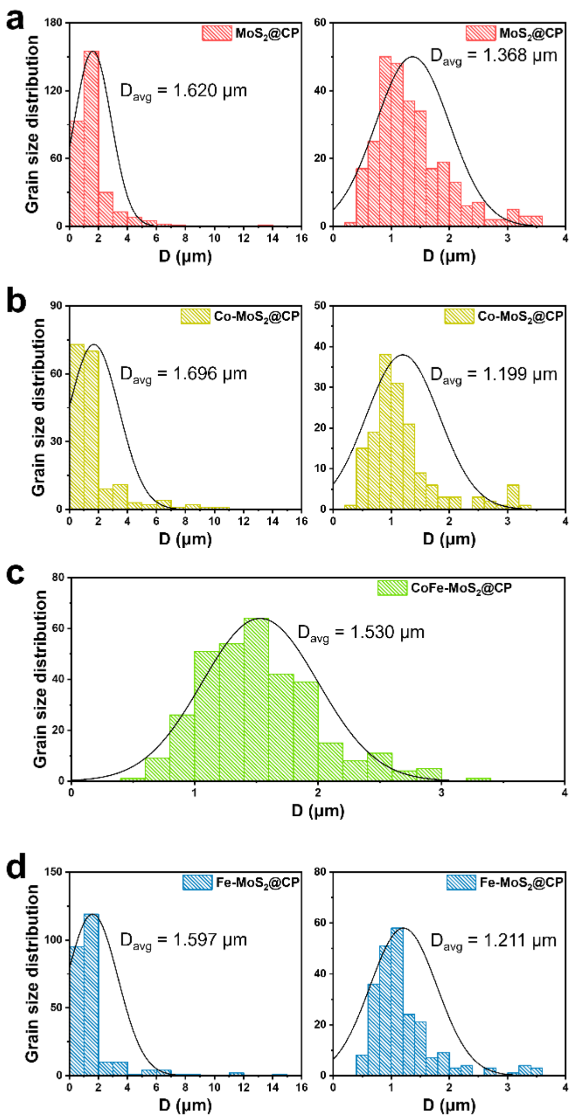

Figure S53. Statistical data on the diameters of the Li_2S nuclei for the (a) MoS_2@CP, (b) Co-MoS_2@CP, (c) CoFe-MoS_2@CP, and (d) Fe-MoS_2@CP cells after the potentiostatic discharge process of a Li_2S_6 electrolyte solution at 2.03 V (vs. Li/Li^+).

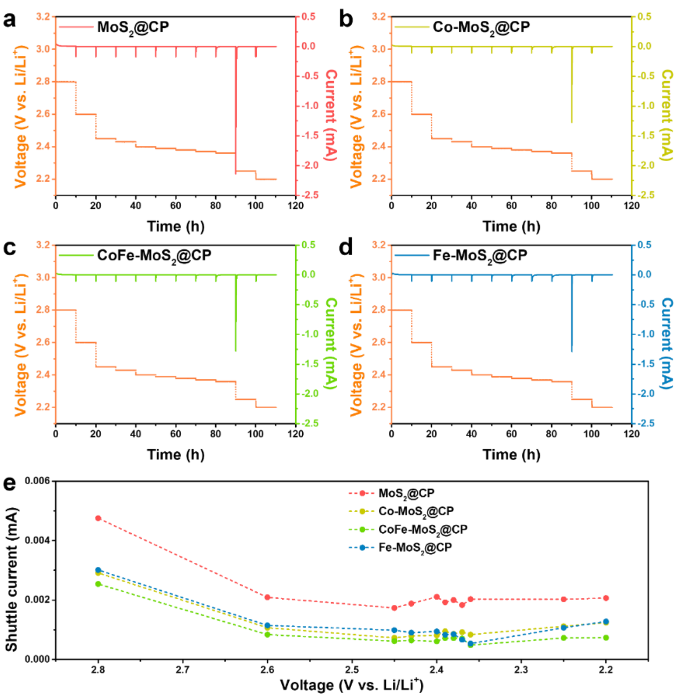

Figure S54. Polysulfide shuttle current measurement. Observation on current relaxation at stepwise changed voltage after cell-formation process of the (a) MoS_2@CP, (b) Co-MoS_2@CP, (c) CoFe-MoS_2@CP, and (d) Fe-MoS_2@CP cells. (e) Recorded shuttle current in steady-state for the cells.

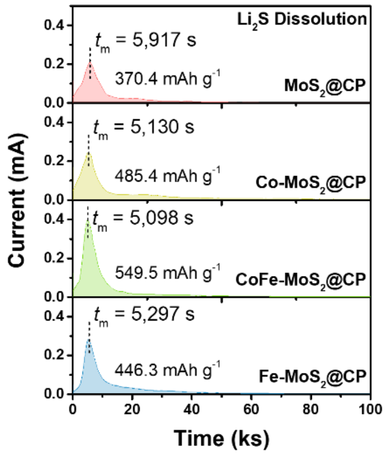

Figure S55. The constant potential curves during Li_2S dissolution test of the MoS_2@CP, Co-MoS_2@CP, CoFe-MoS_2@CP, and Fe-MoS_2@CP cells at 2.35 V (vs. Li/Li^+) for 100 ks.

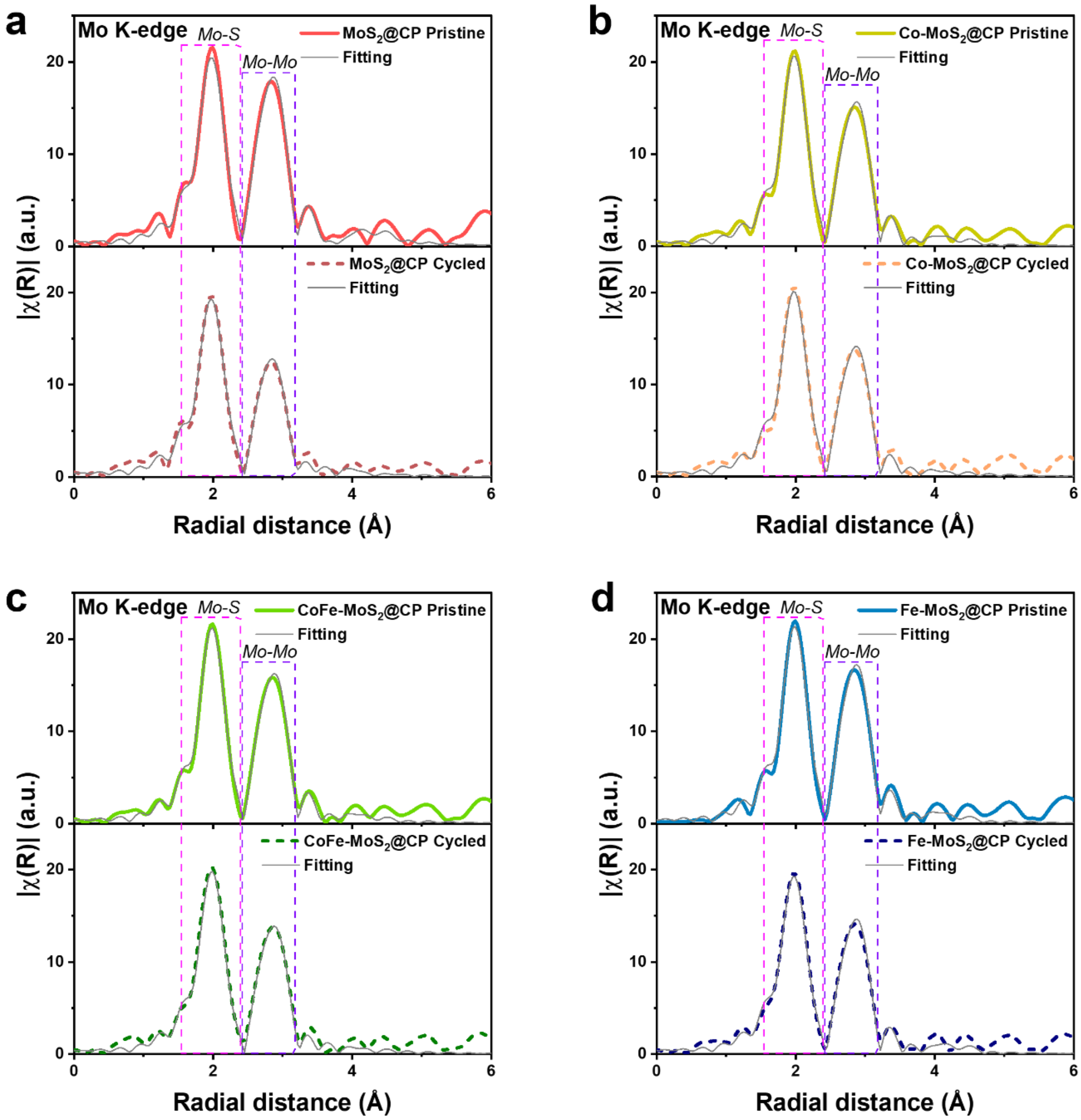

Figure S56. EXAFS mathematical fitting results for the (a) MoS_2@CP, (b) Co-MoS_2@CP, (c) CoFe-MoS_2@CP, and (d) Fe-MoS_2@CP samples at the pristine and cycled states.

Supplementary Note 5

Supplementary XAFS analysis of all Co/Fe co-doped MoS_2 cathode catalysts

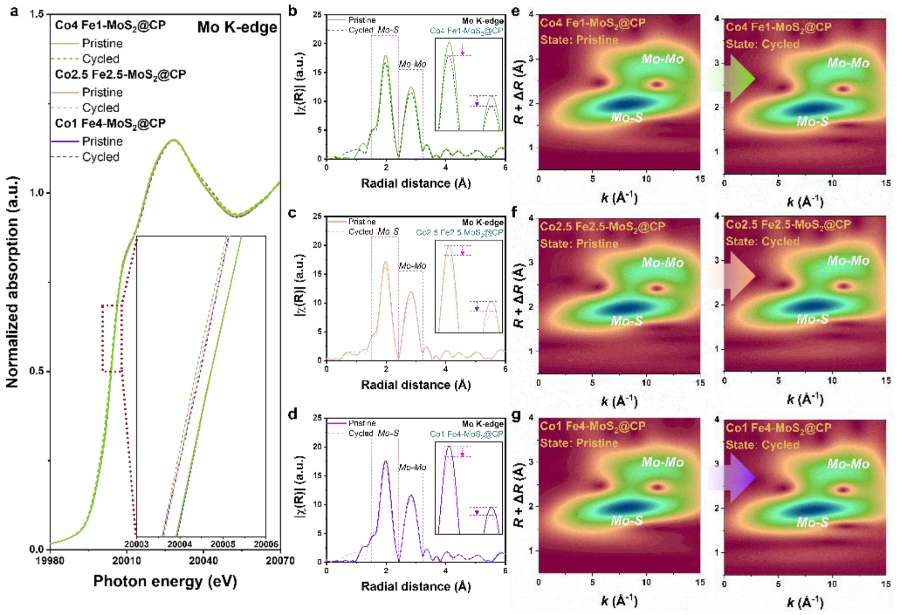

Supplementary Note Figure N22. (a) Normalized Mo K-edge XANES spectra of the Co4 Fe1-MoS_2@CP (CoFe-MoS_2@CP), Co2.5 Fe2.5-MoS_2@CP, and Co1 Fe4-MoS_2@CP interlayers at both the pristine and cycled states. Corresponding k^3-weighted FT- and WT-EXAFS spectra of the (b,e) Co4 Fe1-MoS_2@CP (CoFe-MoS_2@CP), (c,f) Co2.5 Fe2.5-MoS_2@CP, (d,g) Co1 Fe4-MoS_2@CP interlayers at the pristine and cycled states.

XAFS analysis was performed on the n-type Co/Fe co-doped Co4 Fe1-MoS_2@CP, Co2.5 Fe2.5-MoS_2@CP, and Co1 Fe4-MoS_2@CP systems. Although the data were collected utilizing a different XAFS beamline, the overall trends appear to be highly consistent. Specifically, all three samples exhibited similar changes in oxidation state after cycling, among which the Co1 Fe4-MoS_2@CP displayed a relatively slight shift owing to its weak adsorption properties, as expected (Supplementary Note Figure N22a). Furthermore, the EXAFS data revealed no significant degradation of the Mo-S/Mo-Mo bonds in any of the samples, indicating that the doping system is essential for enhancing the durability of the catalyst (Supplementary Note Figures N22b–g).

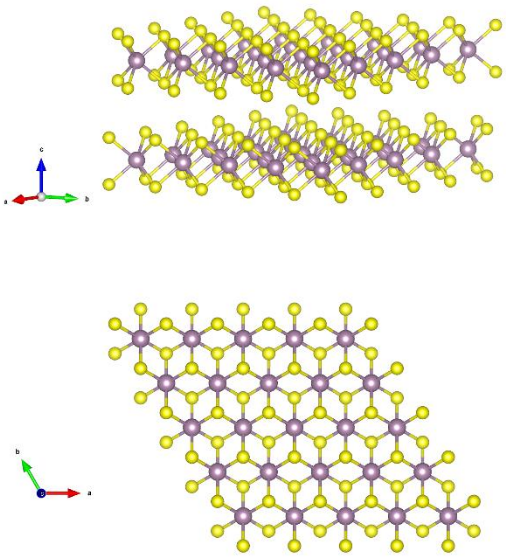

Figure S57. Computational crystal structure for DFT calculations of 1 structural model of MoS_2 (Purple: Mo, Yellow: S).

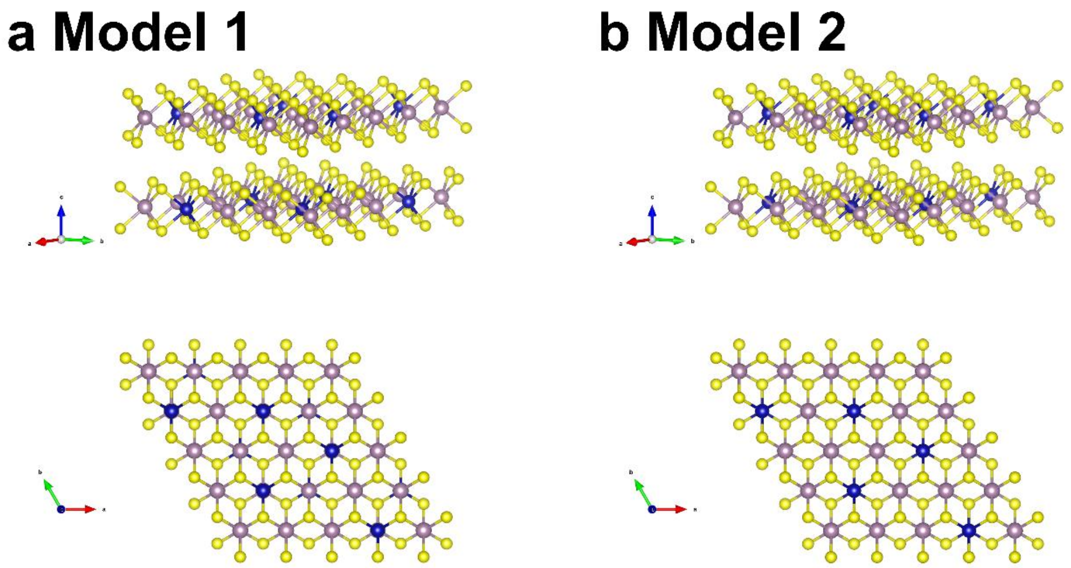

Figure S58. (a, b) Computational crystal structures for DFT calculations of 2 structural models of Co-MoS_2 (Purple: Mo, Blue: Co, Yellow: S).

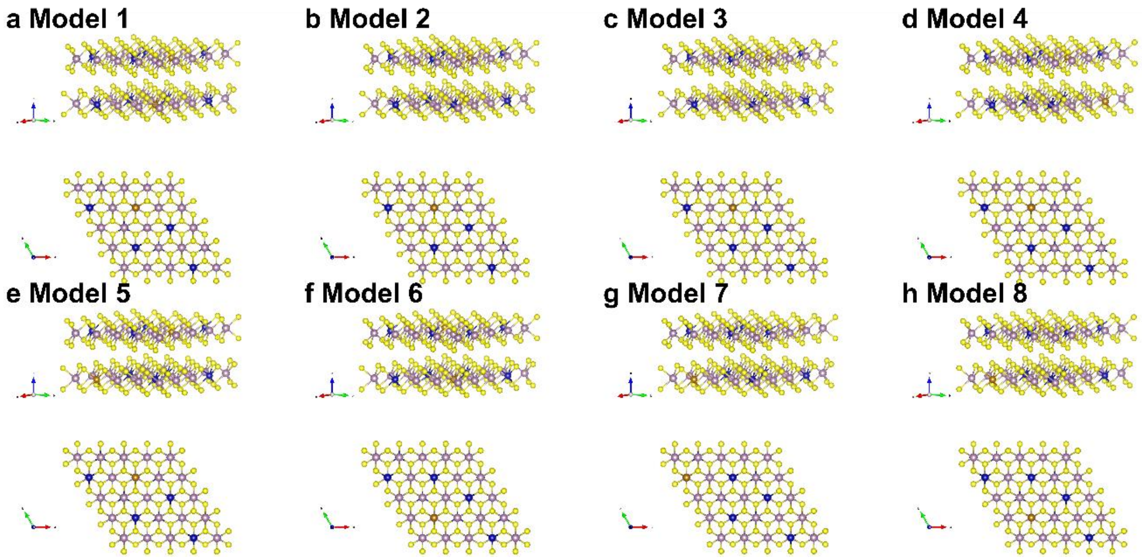

Figure S59. (a–h) Computational crystal structures for DFT calculations, showing 8 out of 17 structural models of CoFe-MoS_2 (Purple: Mo, Blue: Co, Brown: Fe, Yellow: S).

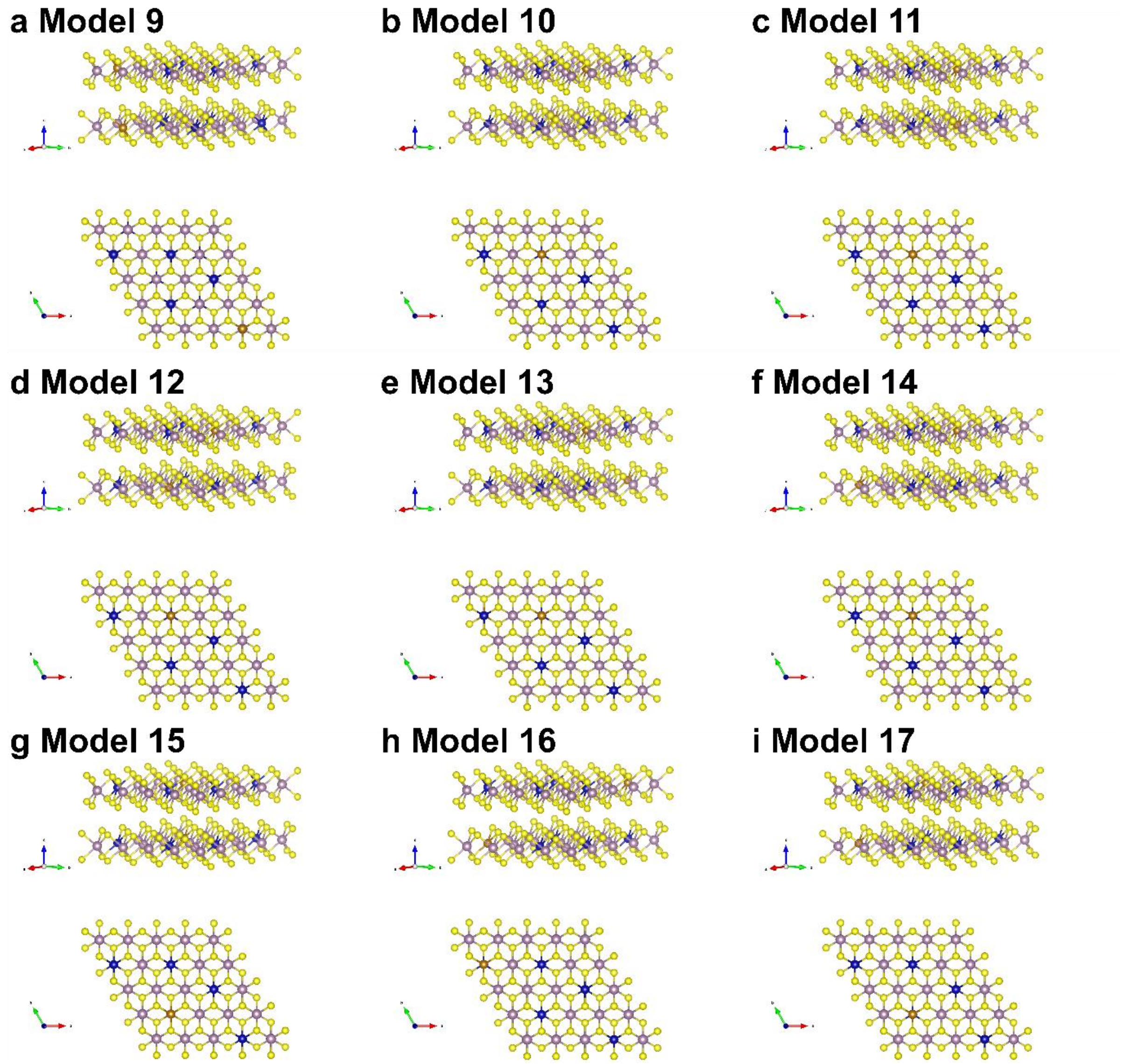

Figure S60. (a–i) Computational crystal structures for DFT calculations, showing 9 out of 17 structural models of CoFe-MoS_2 (Purple: Mo, Blue: Co, Brown: Fe, Yellow: S).

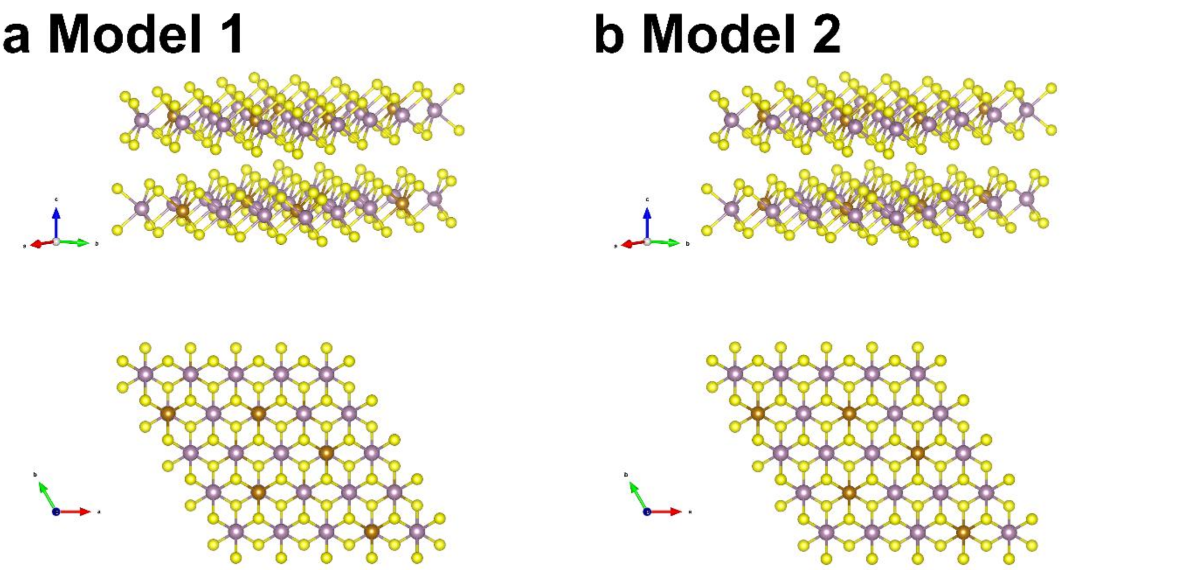

Figure S61. (a, b) Computational crystal structures for DFT calculations of 2 structural models of Fe-MoS_2 (Purple: Mo, Brown: Fe, Yellow: S).

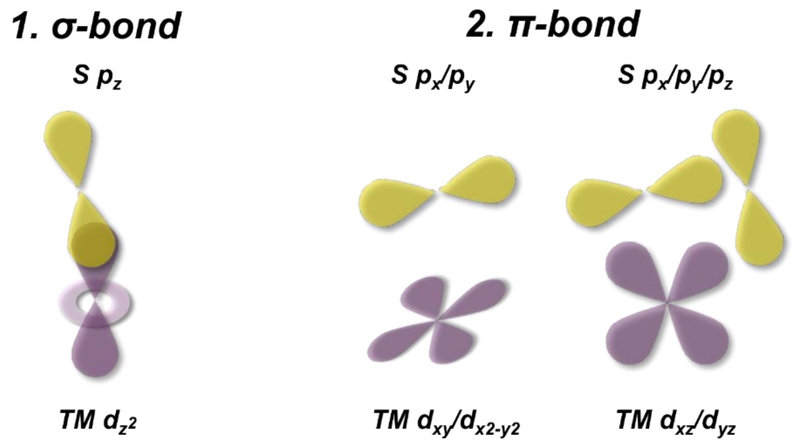

Figure S62. Potential bonding geometries formed between S 3*p* and TM 3*d* orbitals.

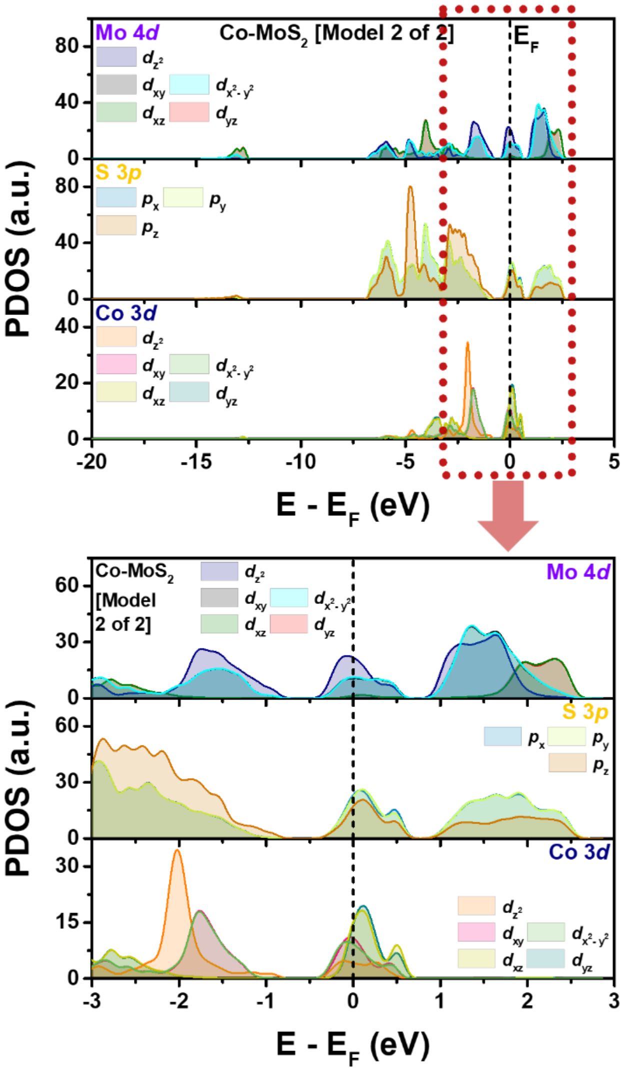

Figure S63. PDOS of Mo 4*d*, S 3*p*, and Co 3*d* orbital energy states of Co-MoS$_2$ [Model 2 of 2].

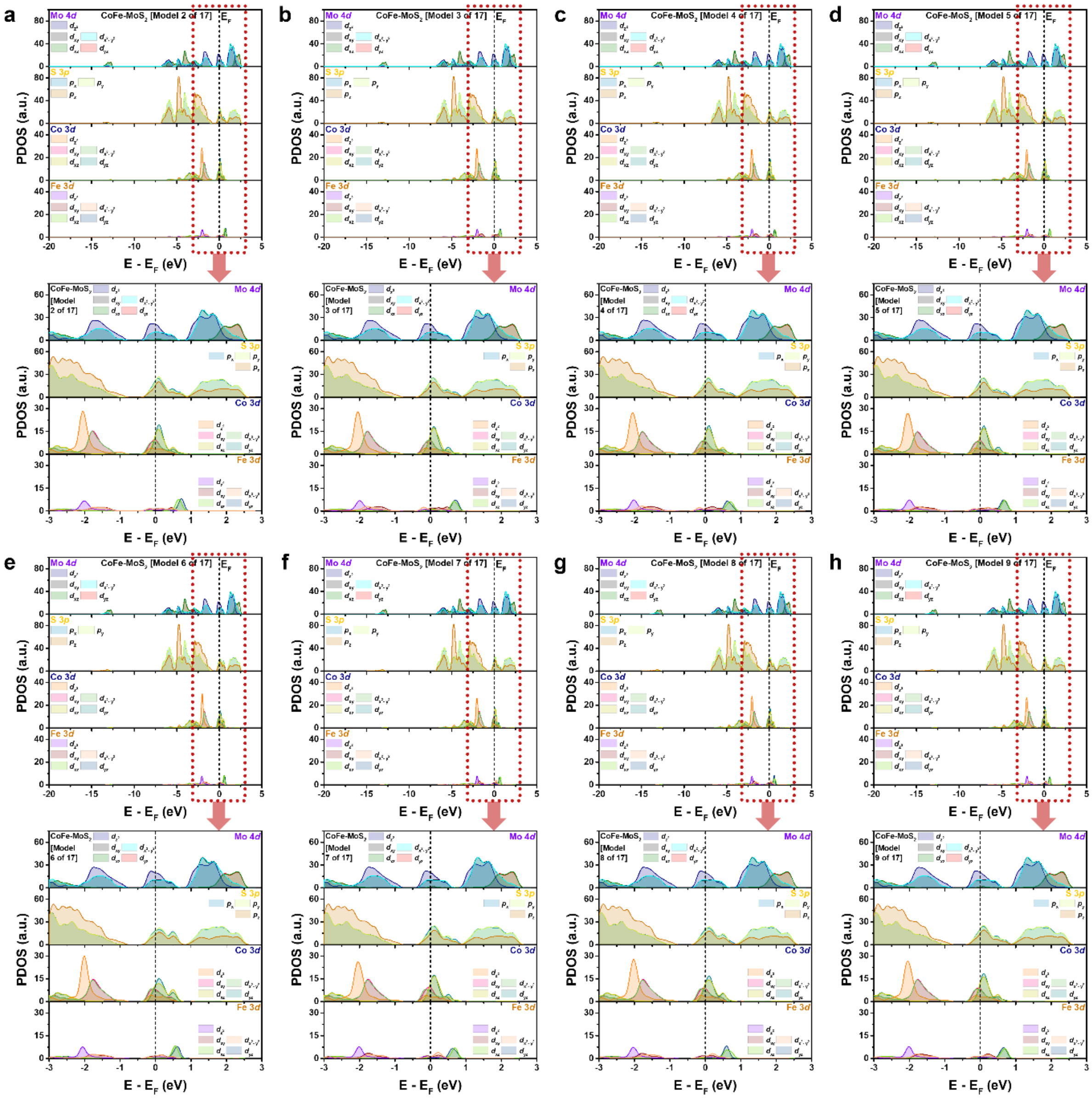

Figure S64. (a–h) PDOS of Mo 4*d*, S 3*p*, Co 3*d*, and Fe 3*d* orbital energy states of CoFe-MoS_2 [from Model 2 of 17 to Model 9 of 17].

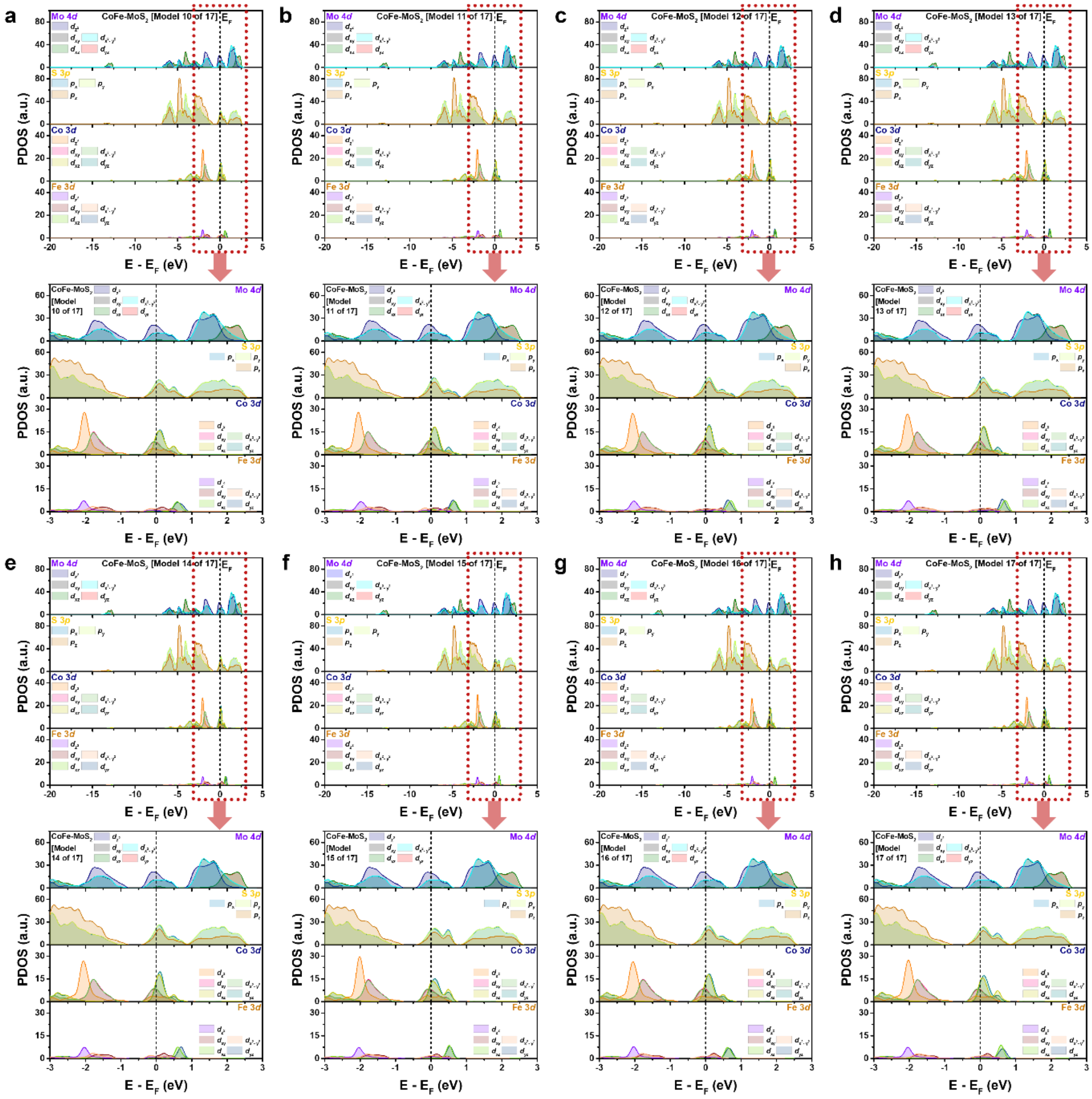

Figure S65. (a–h) PDOS of Mo 4*d*, S 3*p*, Co 3*d*, and Fe 3*d* orbital energy states of CoFe-MoS_2 [from Model 10 of 17 to Model 17 of 17].

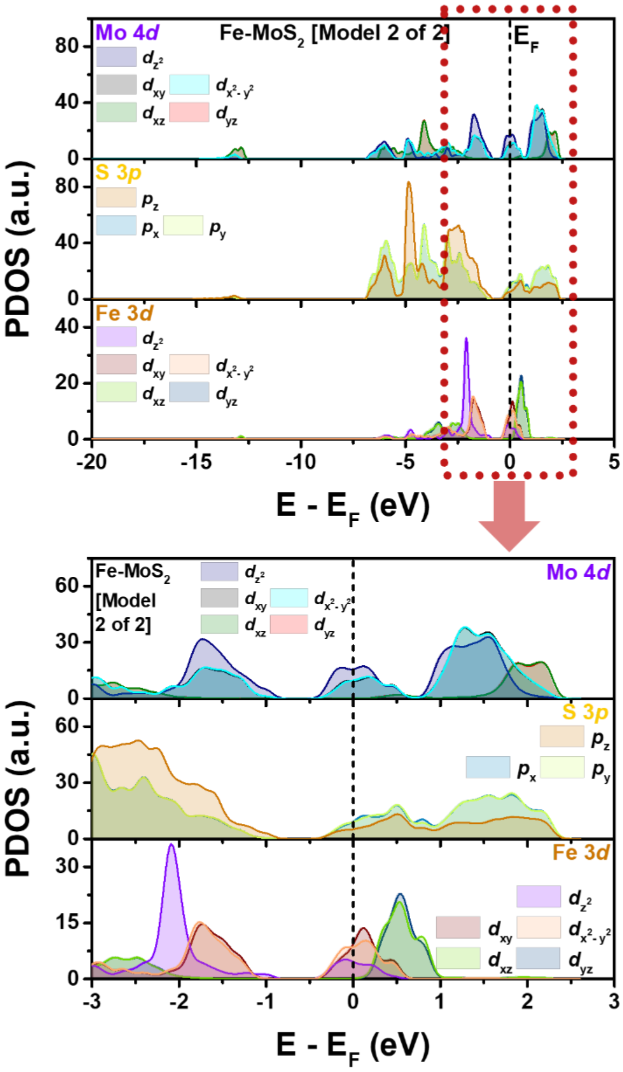

Figure S66. PDOS of Mo 4*d*, S 3*p*, and Fe 3*d* orbital energy states of Fe-MoS_2 [Model 2 of 2].

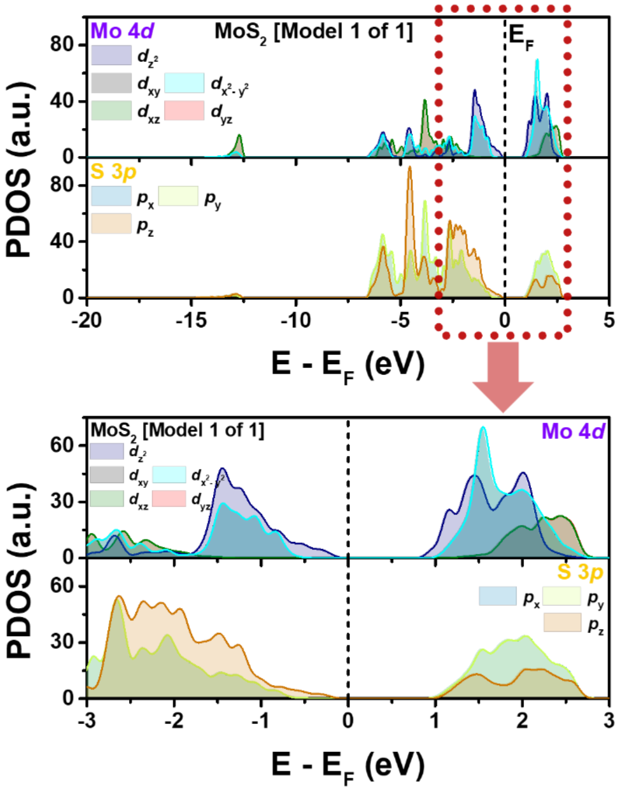

Figure S67. PDOS of Mo 4*d* and S 3*p* orbital energy states of MoS_2 [Model 1 of 1].

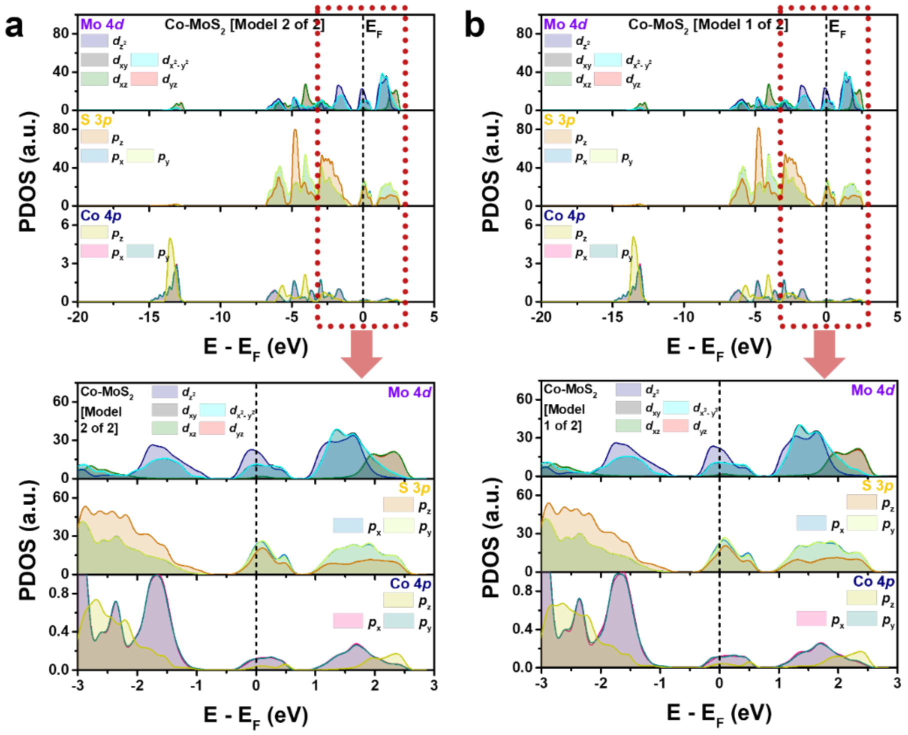

Figure S68. (a, b) PDOS of Mo 4*d*, S 3*p*, and Co 4*p* orbital energy states of Co-MoS_2 [from Model 1 of 2 to Model 2 of 2].

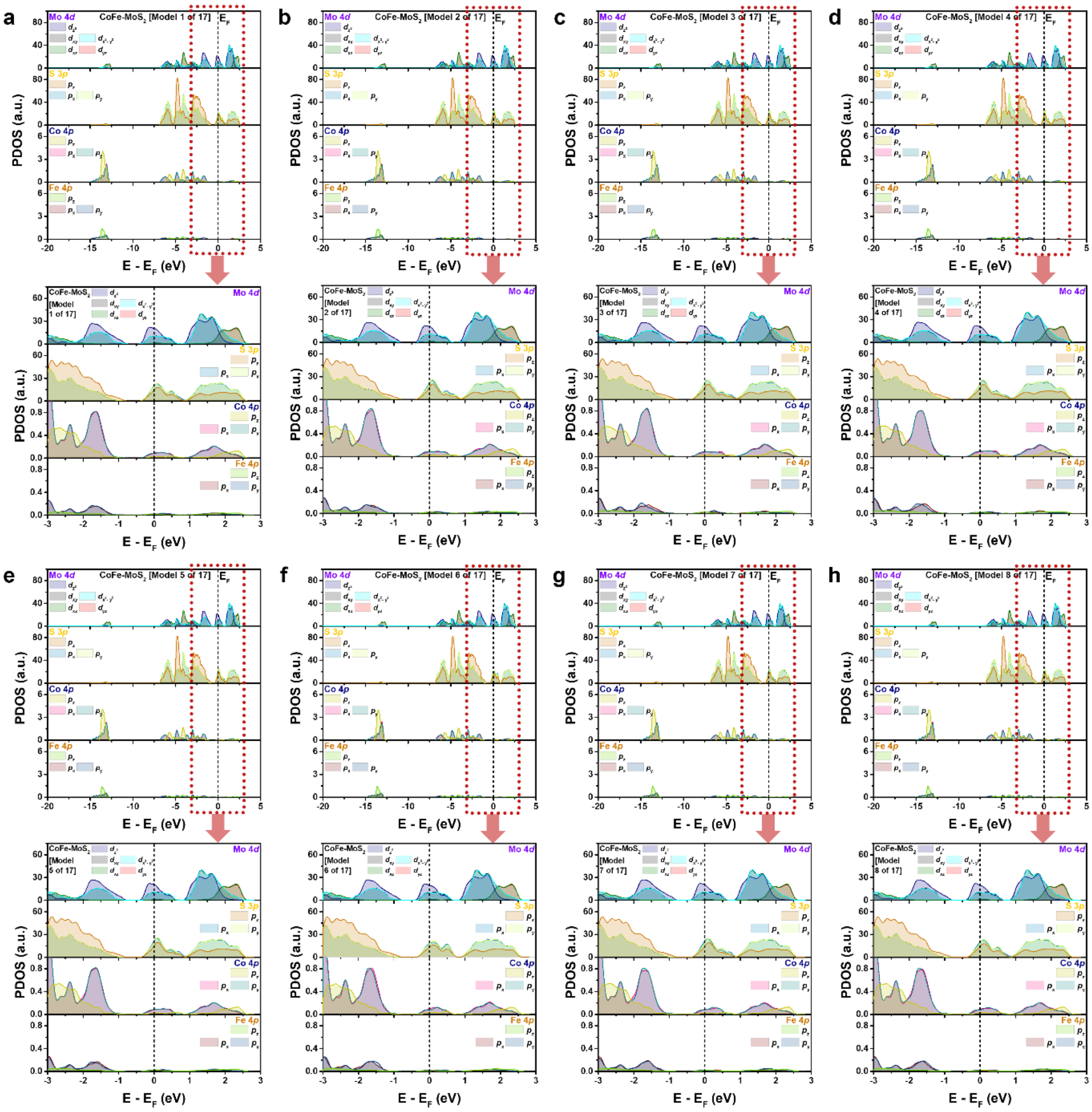

Figure S69. (a–h) PDOS of Mo 4*d*, S 3*p*, Co 4*p*, and Fe 4*p* orbital energy states of CoFe-MoS_2 [from Model 1 of 17 to Model 8 of 17].

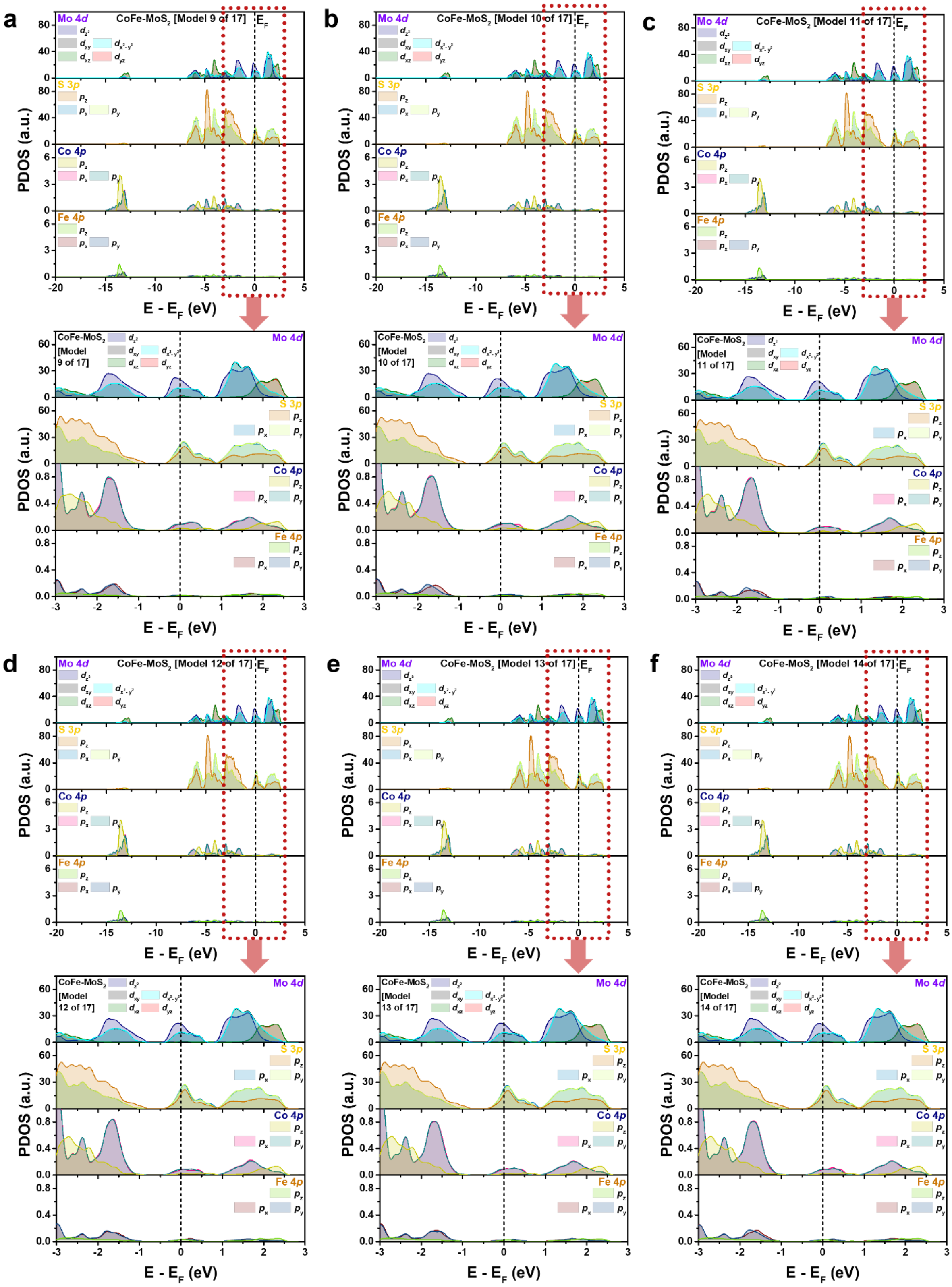

Figure S70. (a–f) PDOS of Mo 4*d*, S 3*p*, Co 4*p*, and Fe 4*p* orbital energy states of CoFe-MoS_2 [from Model 9 of 17 to Model 14 of 17].

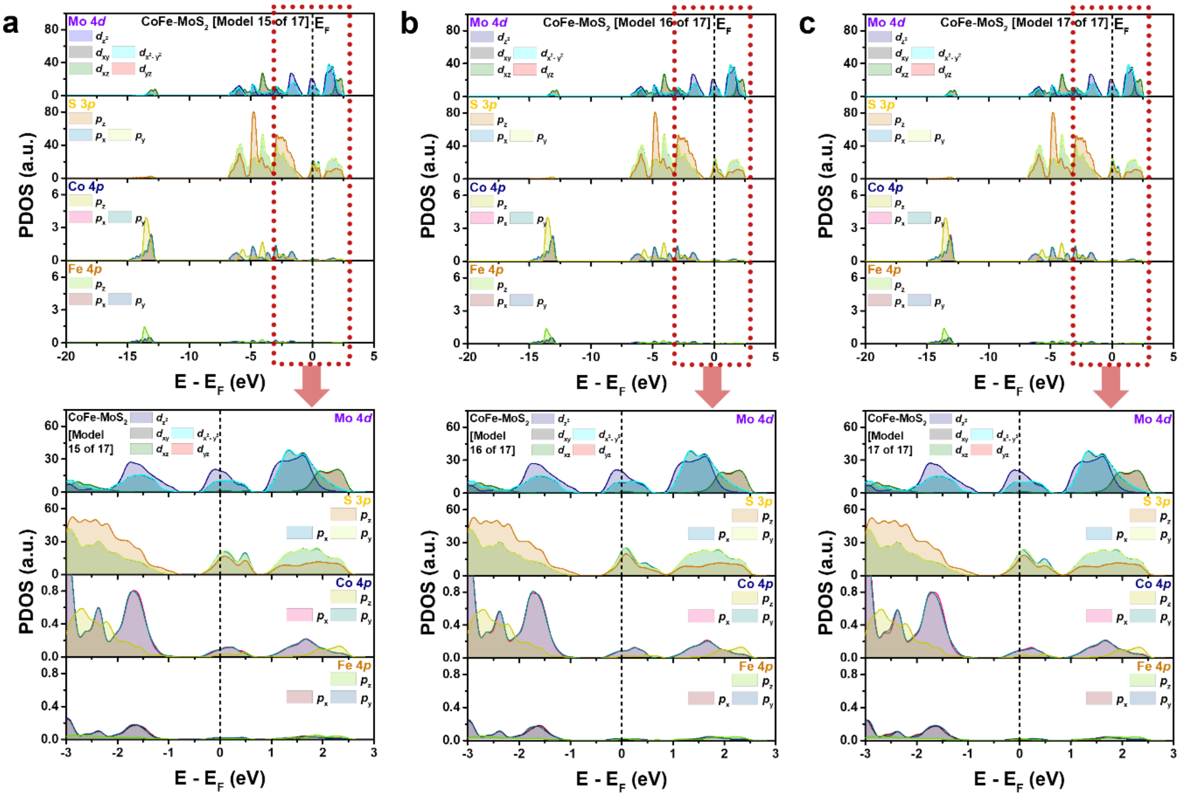

Figure S71. (a–c) PDOS of Mo 4*d*, S 3*p*, Co 4*p*, and Fe 4*p* orbital energy states of CoFe-MoS_2 [from Model 15 of 17 to Model 17 of 17].

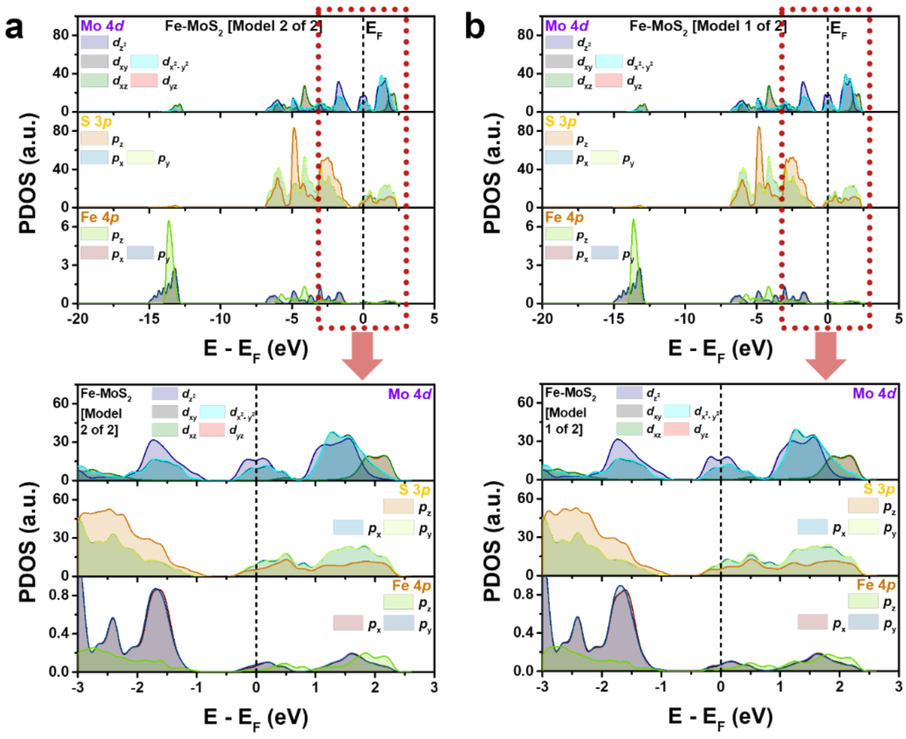

Figure S72. (a, b) PDOS of Mo 4*d*, S 3*p*, and Fe 4*p* orbital energy states of Fe-MoS_2 [from Model 1 of 2 to Model 2 of 2].

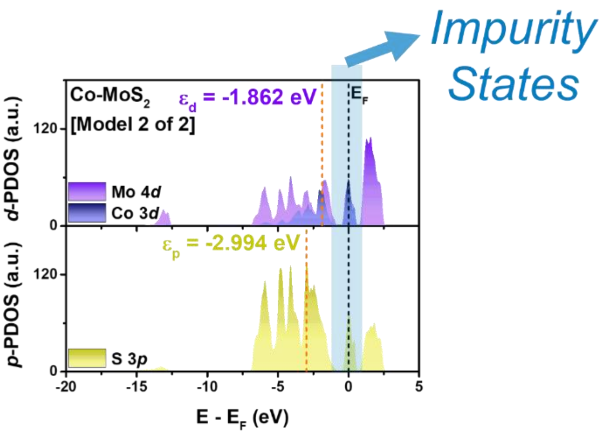

Figure S73. *d*-PDOS of Mo 4*d*, Co 3*d* and *p*-PDOS of S 3*p* orbital energy states of Co-MoS$_2$ [Model 2 of 2].

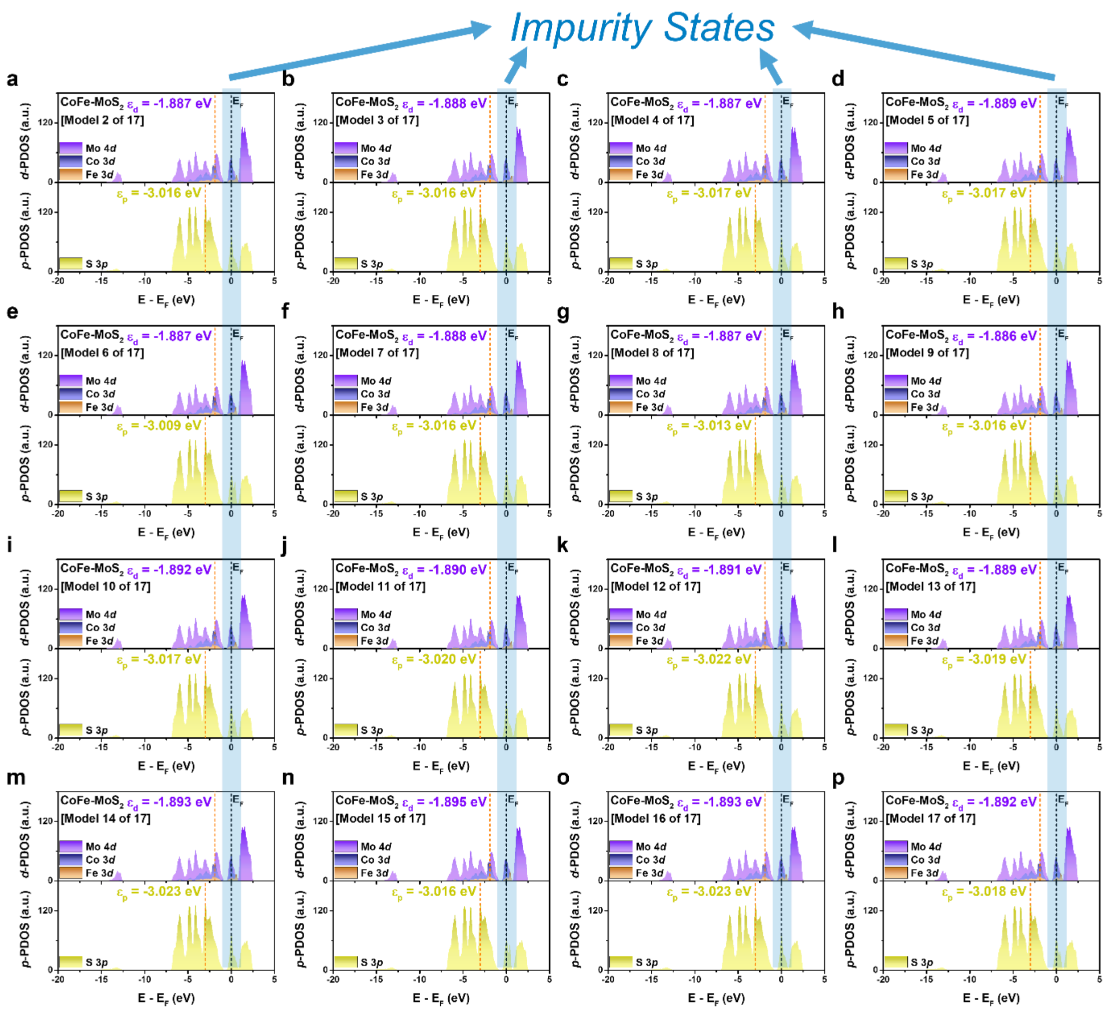

Figure S74. (a–p) *d*-PDOS of Mo 4*d*, Co 3*d*, Fe 3*d* and *p*-PDOS of S 3*p* orbital energy states of CoFe-MoS$_2$ [from Model 2 of 17 to Model 17 of 17].

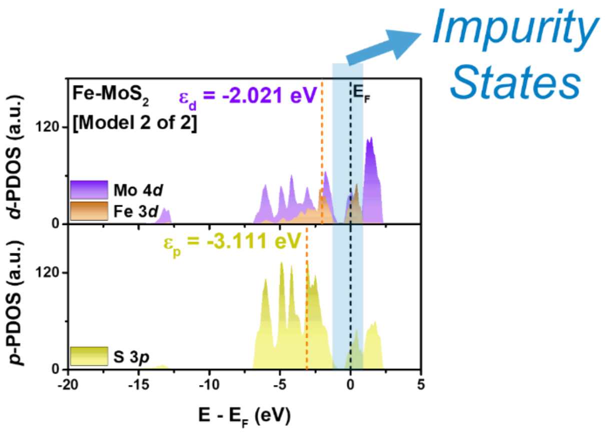

Figure S75. *d*-PDOS of Mo 4*d*, Fe 3*d* and *p*-PDOS of S 3*p* orbital energy states of Fe-MoS_2 [Model 2 of 2].

Supplementary Note 6

Design and optimization workflow for the Co2.5 Fe2.5-/Co1 Fe4-MoS$_2$ catalyst models

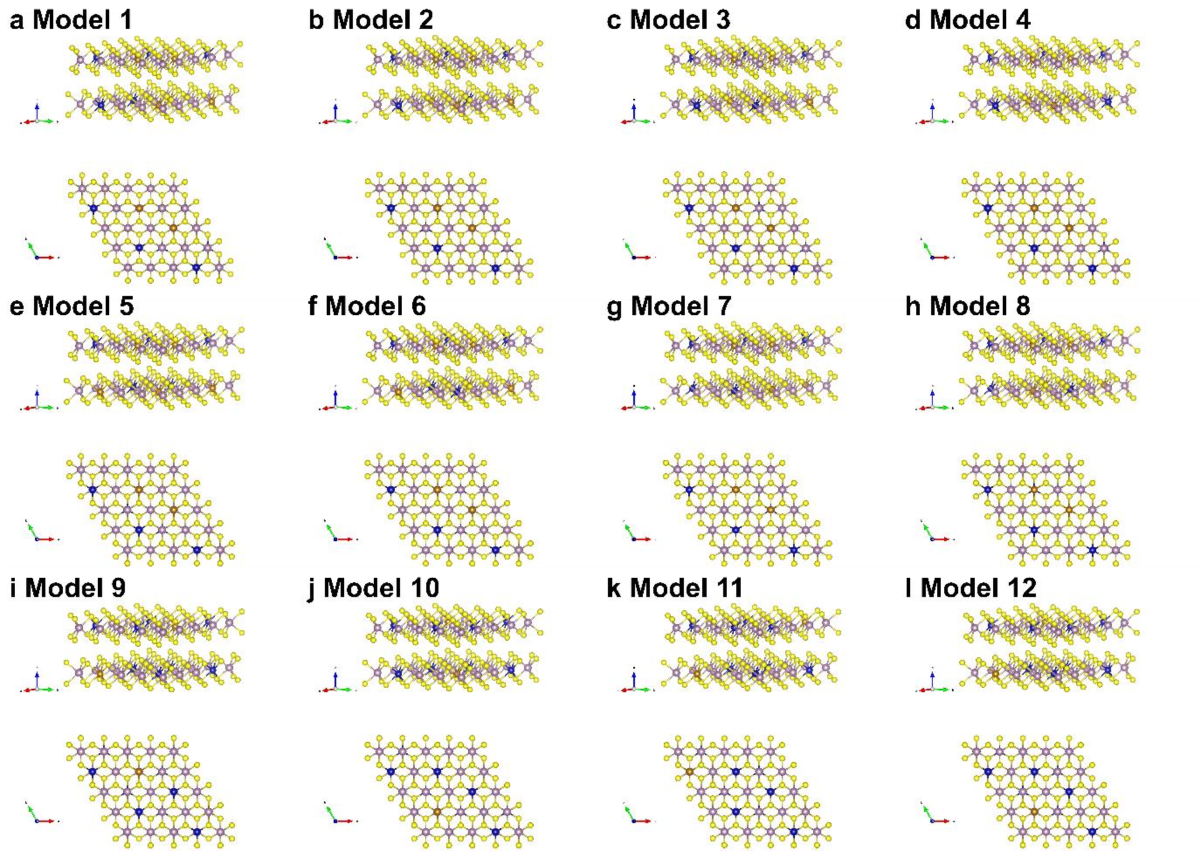

Supplementary Note Figure N23. (a–l) Computational crystal structures for DFT calculations of 12 structural models of Co2.5 Fe2.5-MoS$_2$ (Purple: Mo, Blue: Co, Brown: Fe, Yellow: S).

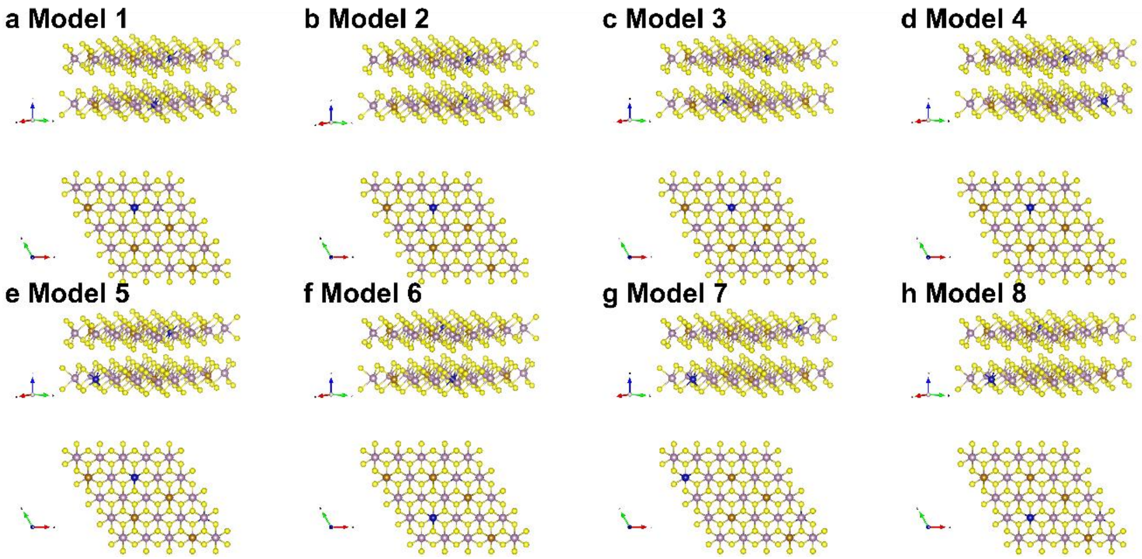

Supplementary Note Figure N24. (a–h) Computational crystal structures for DFT calculations, showing 8 out of 17 structural models of Co1 Fe4-MoS_2 (Purple: Mo, Blue: Co, Brown: Fe, Yellow: S).

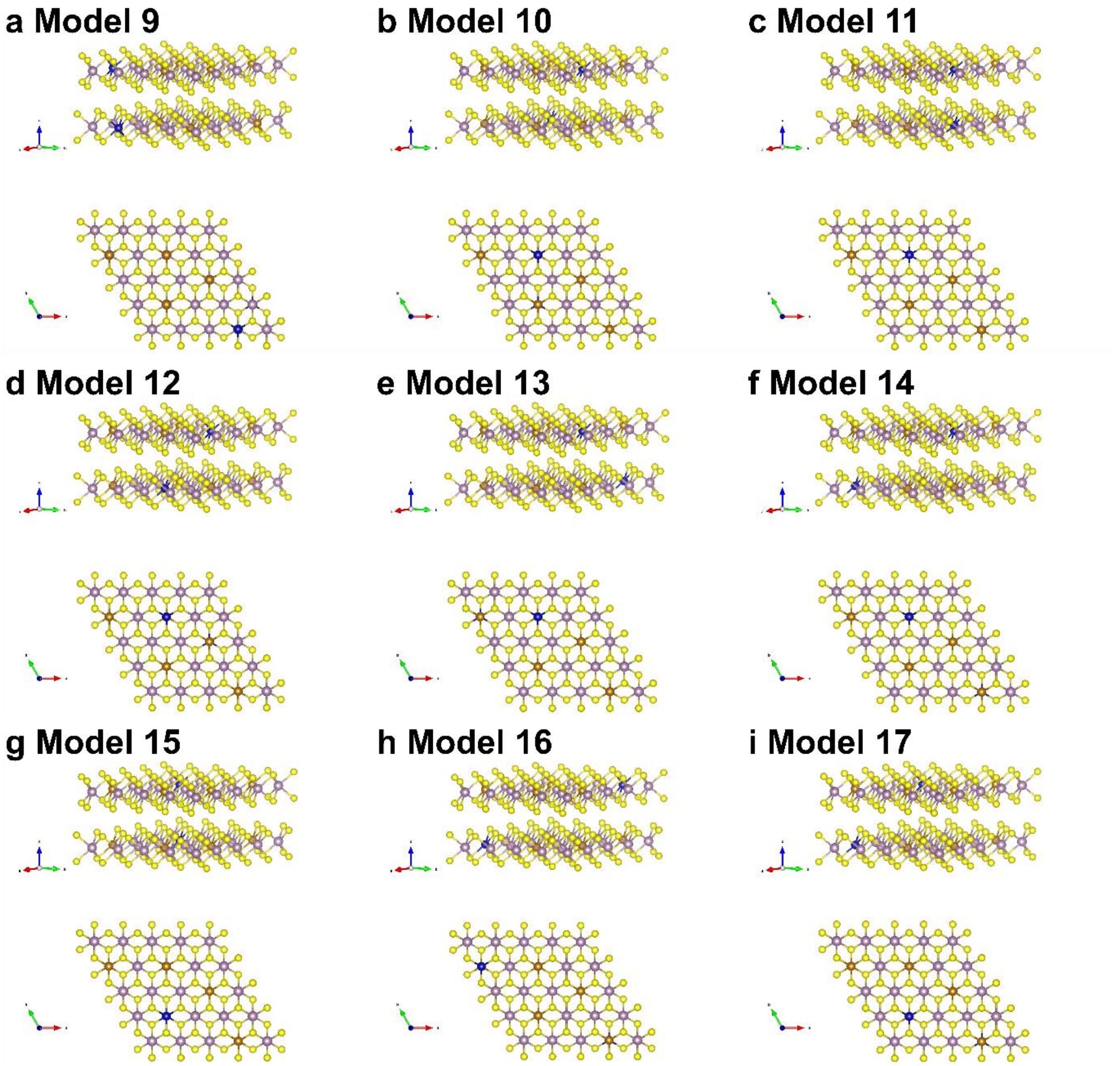

Supplementary Note Figure N25. (a–i) Computational crystal structures for DFT calculations, showing 9 out of 17 structural models of Co1 Fe4-MoS_2 (Purple: Mo, Blue: Co, Brown: Fe, Yellow: S).

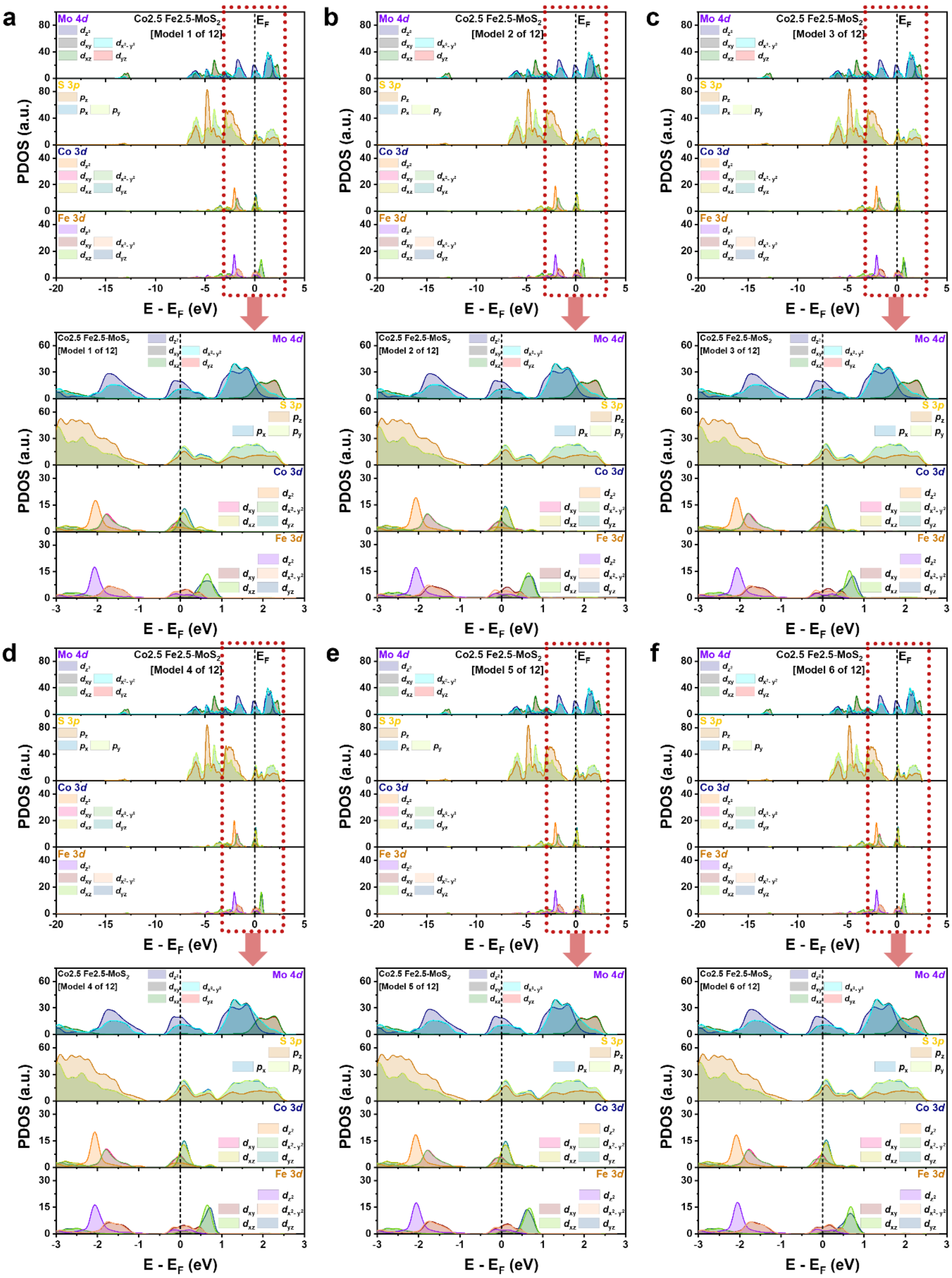

Supplementary Note Figure N26. (a–f) PDOS of Mo 4*d*, S 3*p*, Co 3*d*, and Fe 3*d* orbital energy states of Co2.5 Fe2.5-MoS_2 [from Model 1 of 12 to Model 6 of 12].

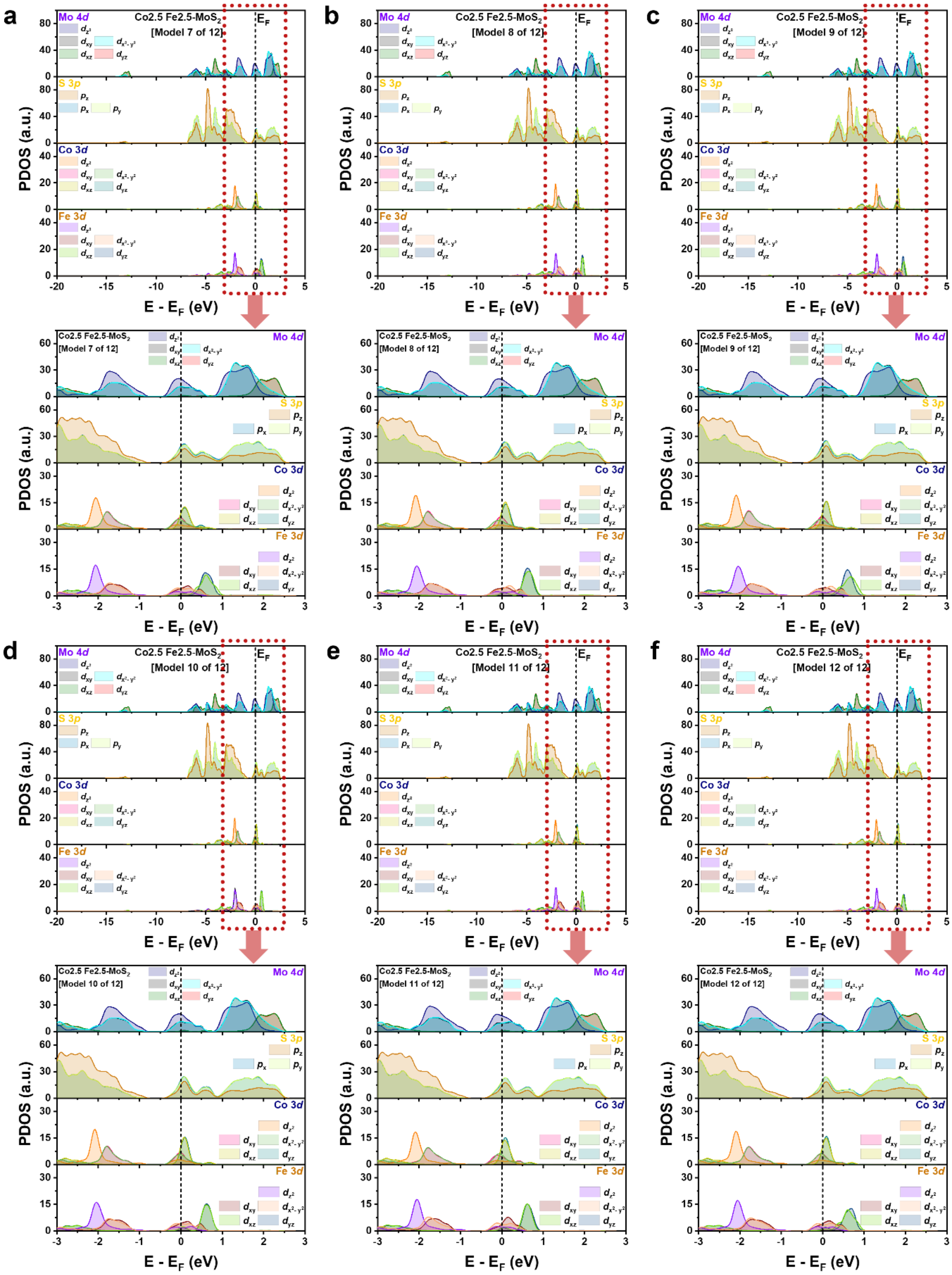

Supplementary Note Figure N27. (a–f) PDOS of Mo 4*d*, S 3*p*, Co 3*d*, and Fe 3*d* orbital energy states of Co2.5 Fe2.5-MoS_2 [from Model 7 of 12 to Model 12 of 12].

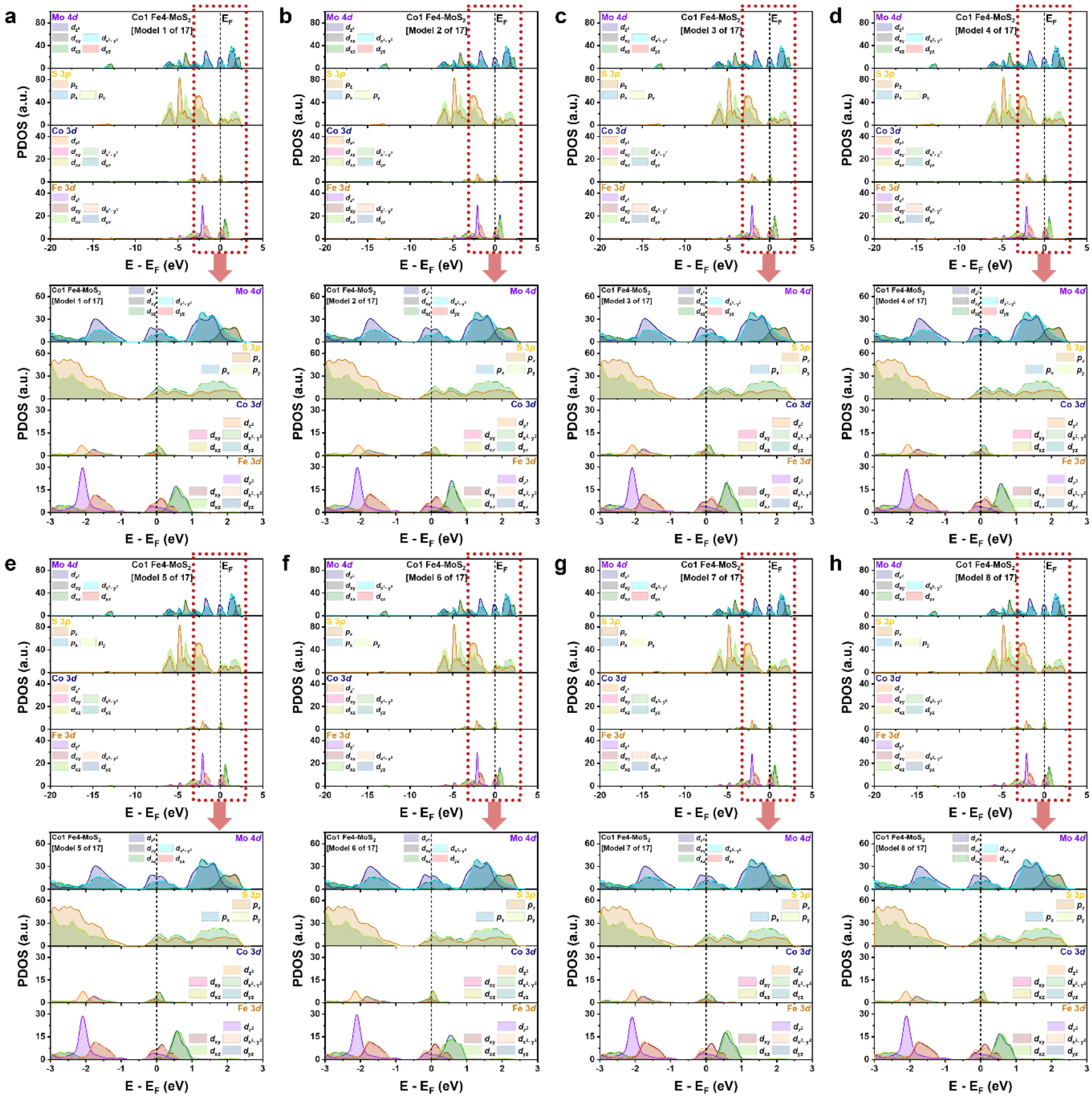

Supplementary Note Figure N28. (a–h) PDOS of Mo 4*d*, S 3*p*, Co 3*d*, and Fe 3*d* orbital energy states of Co1 Fe4-MoS_2 [from Model 1 of 17 to Model 8 of 17].

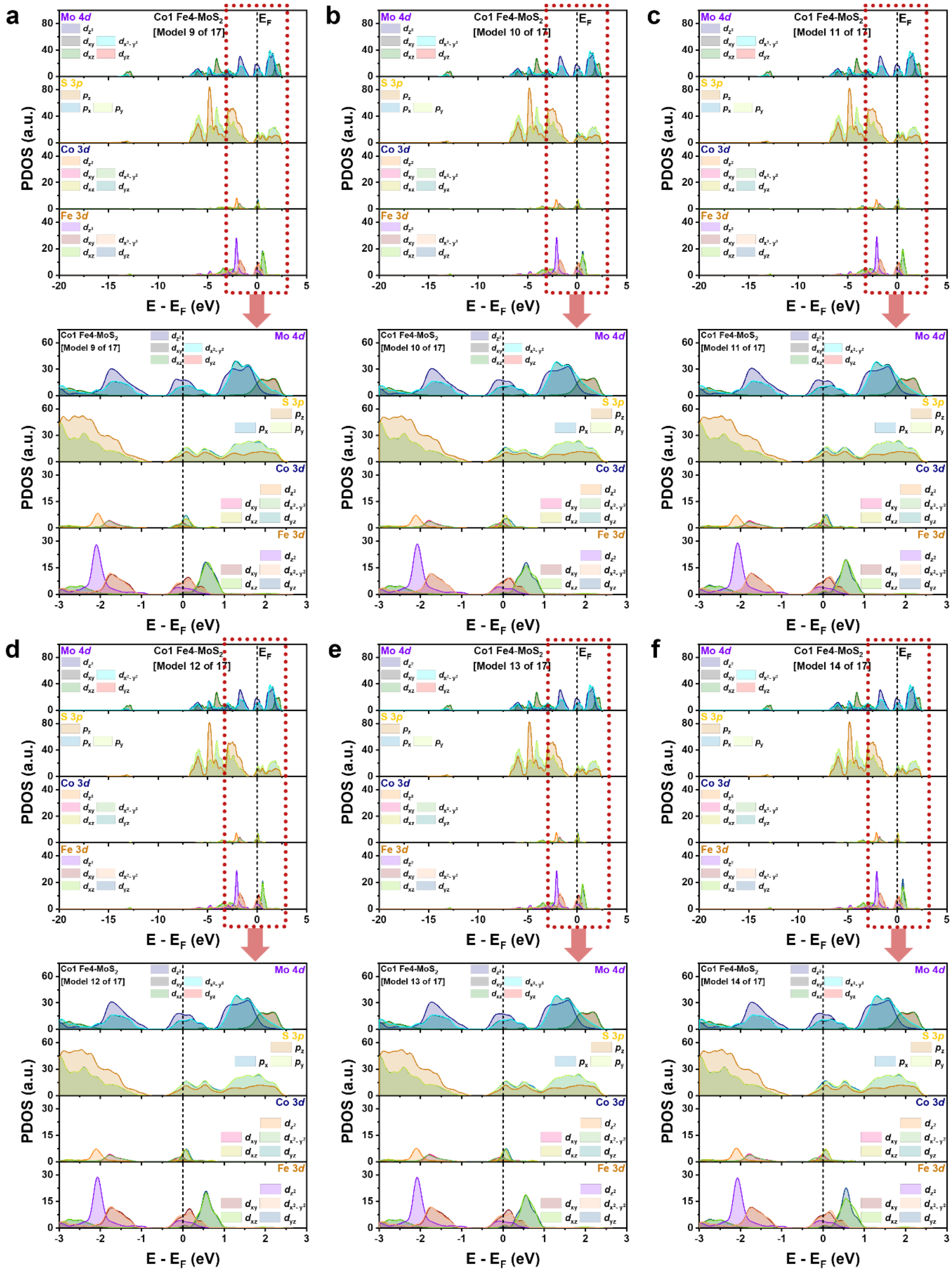

Supplementary Note Figure N29. (a–f) PDOS of Mo 4*d*, S 3*p*, Co 3*d*, and Fe 3*d* orbital energy states of Co1 Fe4-MoS_2 [from Model 9 of 17 to Model 14 of 17].

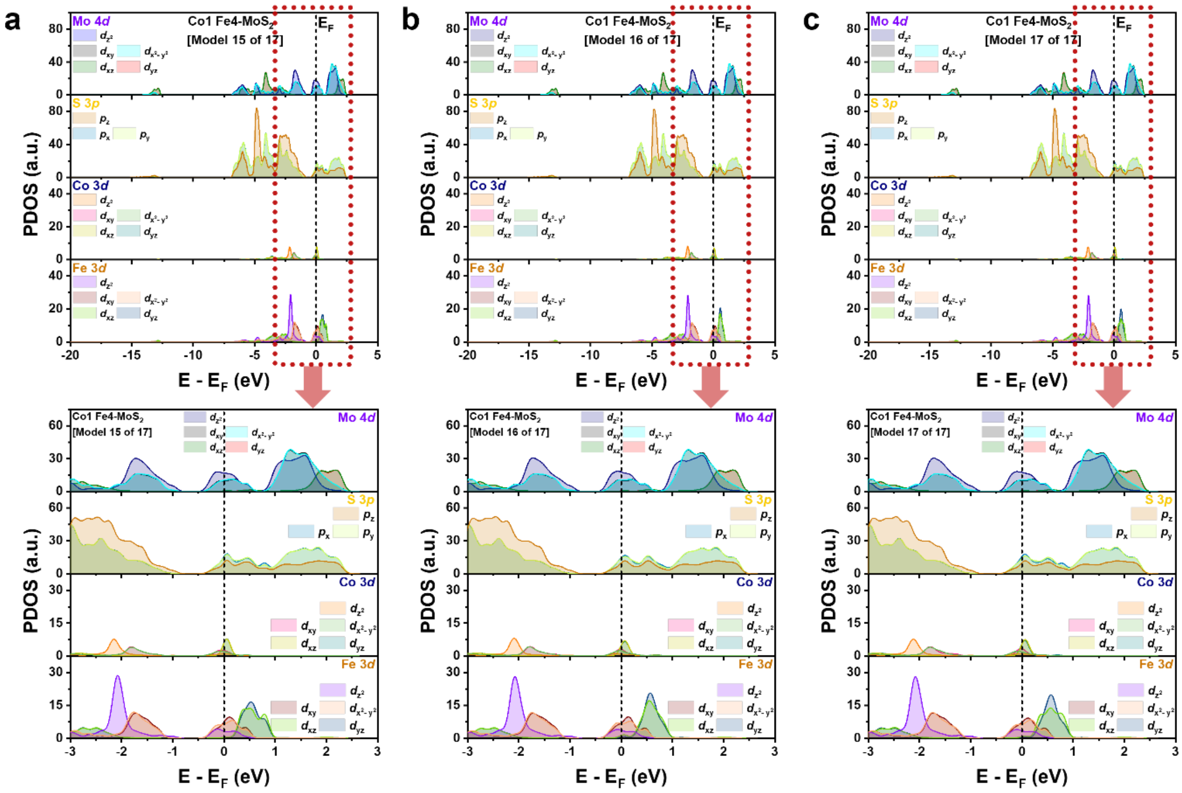

Supplementary Note Figure N30. (a–c) PDOS of Mo 4*d*, S 3*p*, Co 3*d*, and Fe 3*d* orbital energy states of Co1 Fe4-MoS_2 [from Model 15 of 17 to Model 17 of 17].

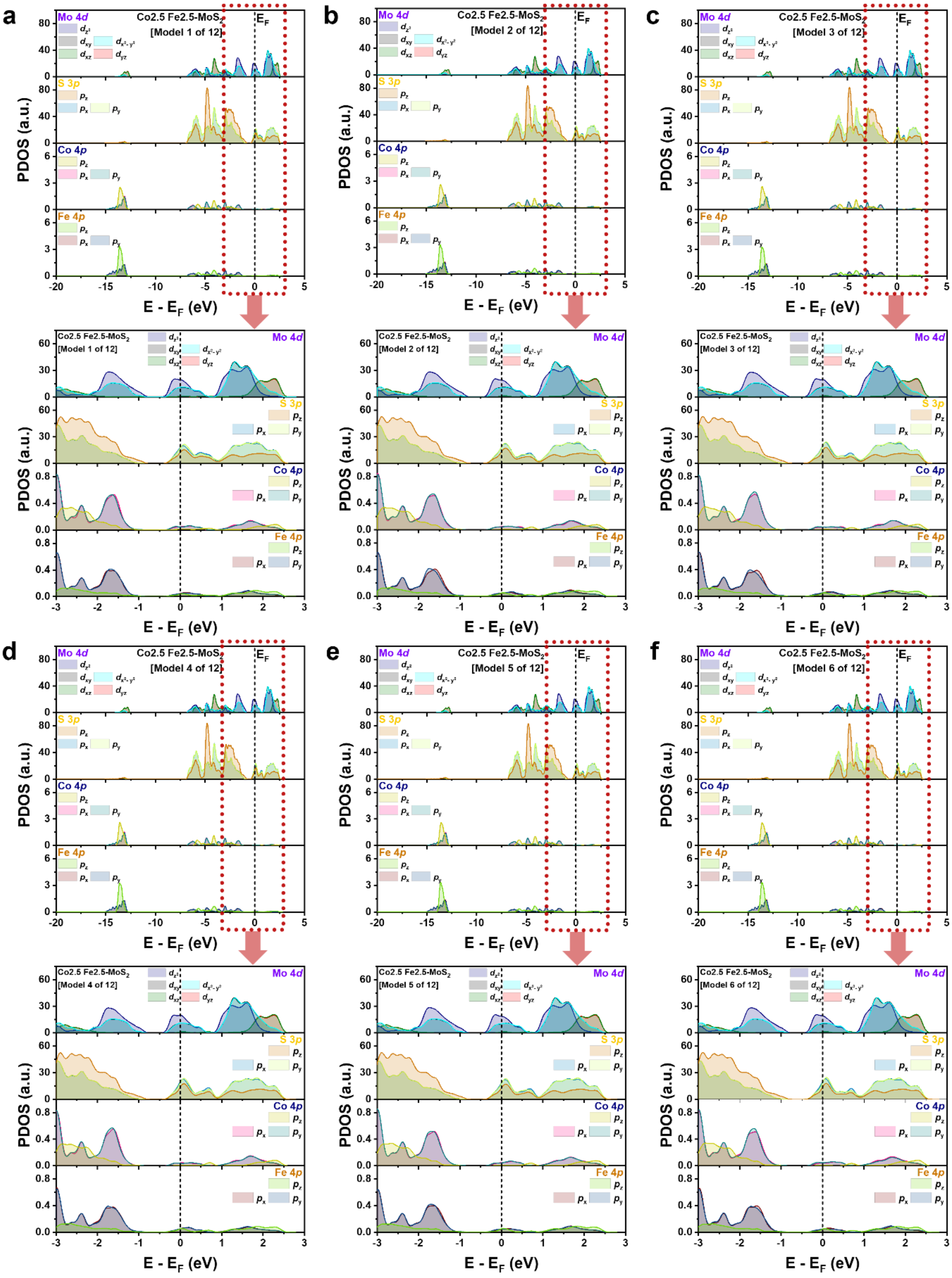

Supplementary Note Figure N31. (a–f) PDOS of Mo 4*d*, S 3*p*, Co 4*p*, and Fe 4*p* orbital energy states of Co2.5 Fe2.5-MoS_2 [from Model 1 of 12 to Model 6 of 12].

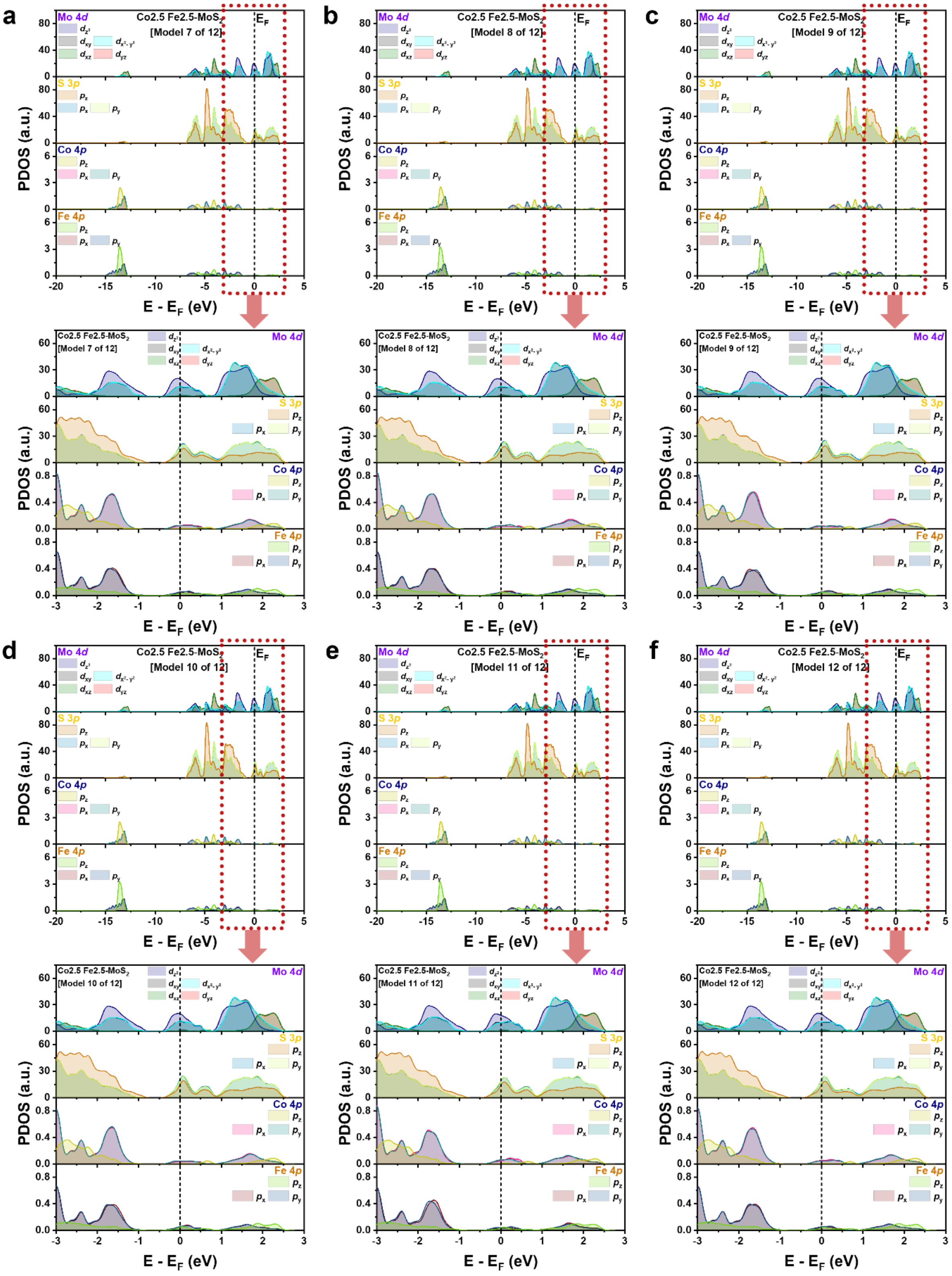

Supplementary Note Figure N32. (a–f) PDOS of Mo 4*d*, S 3*p*, Co 4*p*, and Fe 4*p* orbital energy states of Co2.5 Fe2.5-MoS_2 [from Model 7 of 12 to Model 12 of 12].

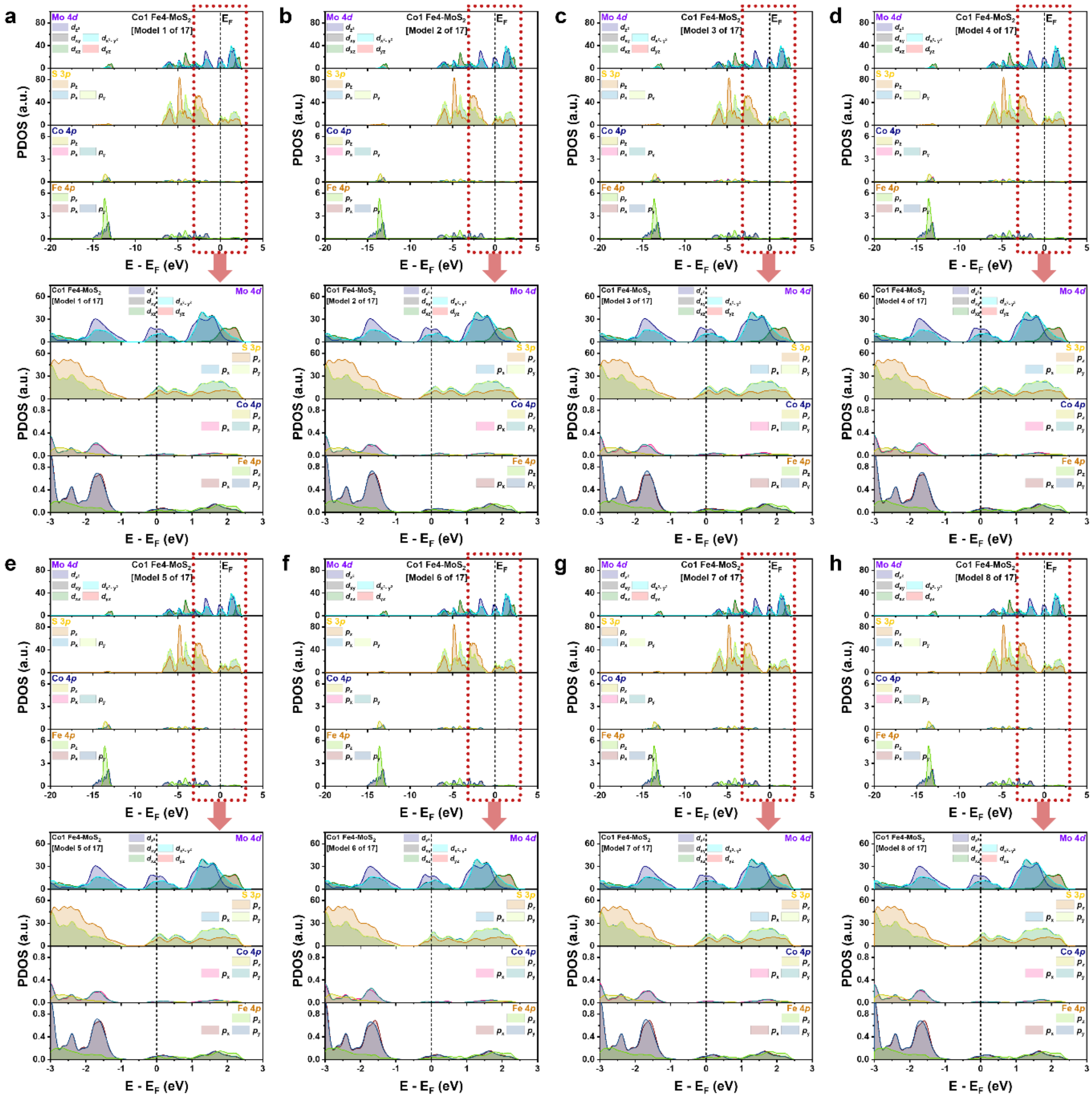

Supplementary Note Figure N33. (a–h) PDOS of Mo 4*d*, S 3*p*, Co 4*p*, and Fe 4*p* orbital energy states of Co1 Fe4-MoS_2 [from Model 1 of 17 to Model 8 of 17].

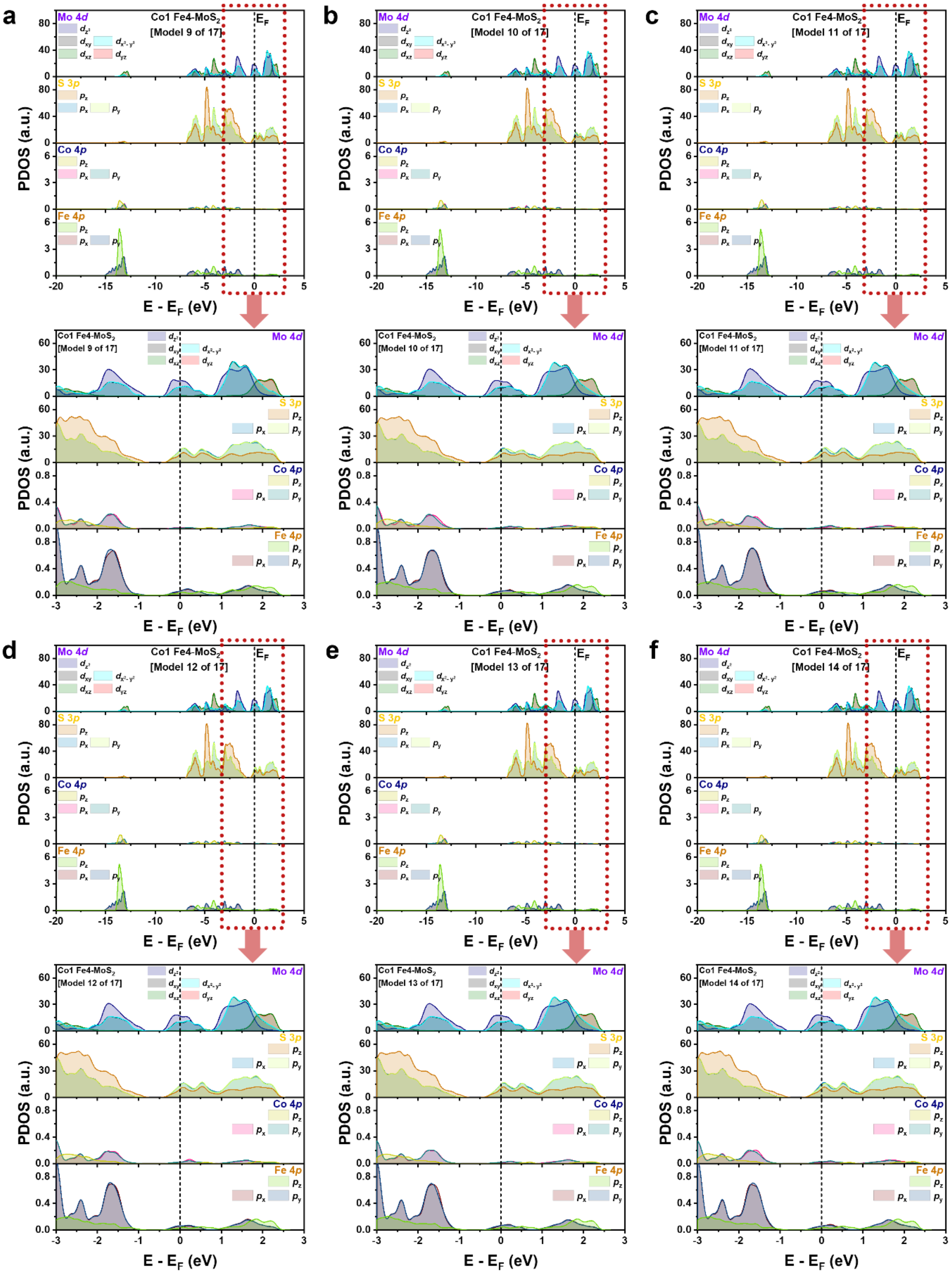

Supplementary Note Figure N34. (a–f) PDOS of Mo 4*d*, S 3*p*, Co 4*p*, and Fe 4*p* orbital energy states of Co1 Fe4-MoS_2 [from Model 9 of 17 to Model 14 of 17].

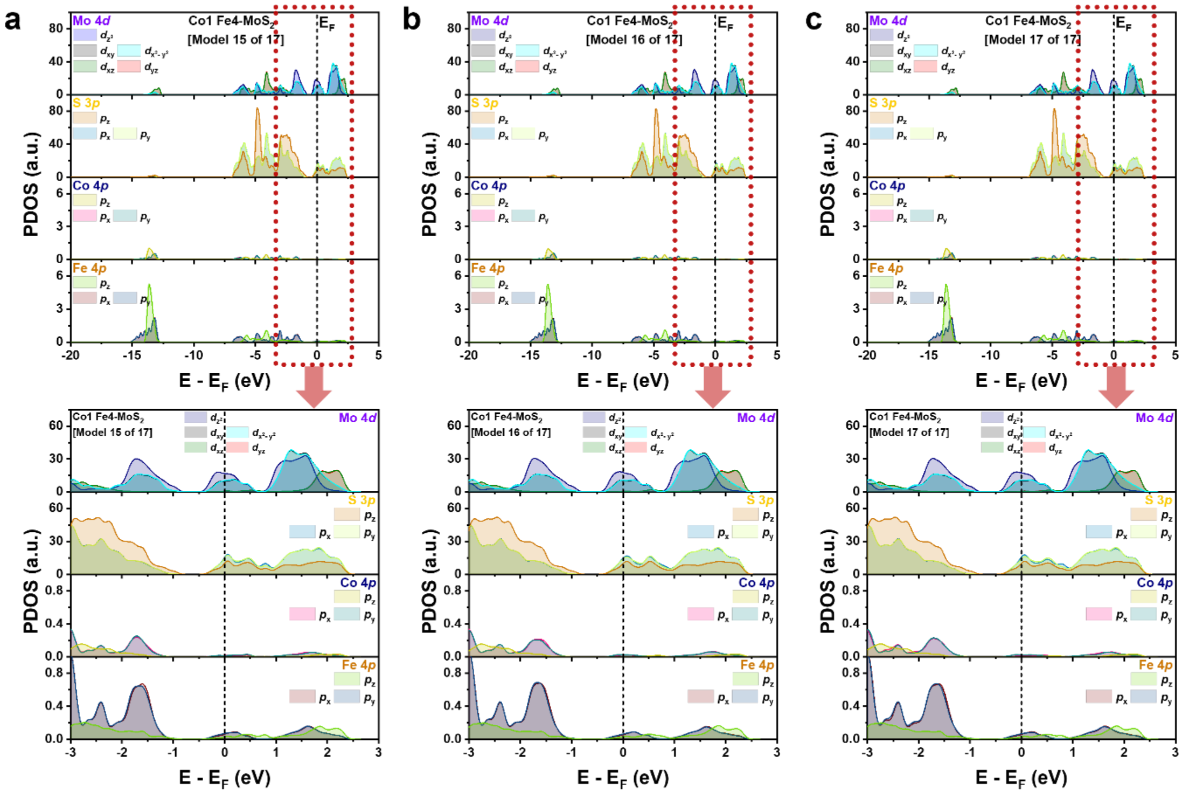

Supplementary Note Figure N35. (a–c) PDOS of Mo 4*d*, S 3*p*, Co 4*p*, and Fe 4*p* orbital energy states of Co1 Fe4-MoS_2 [from Model 15 of 17 to Model 17 of 17].

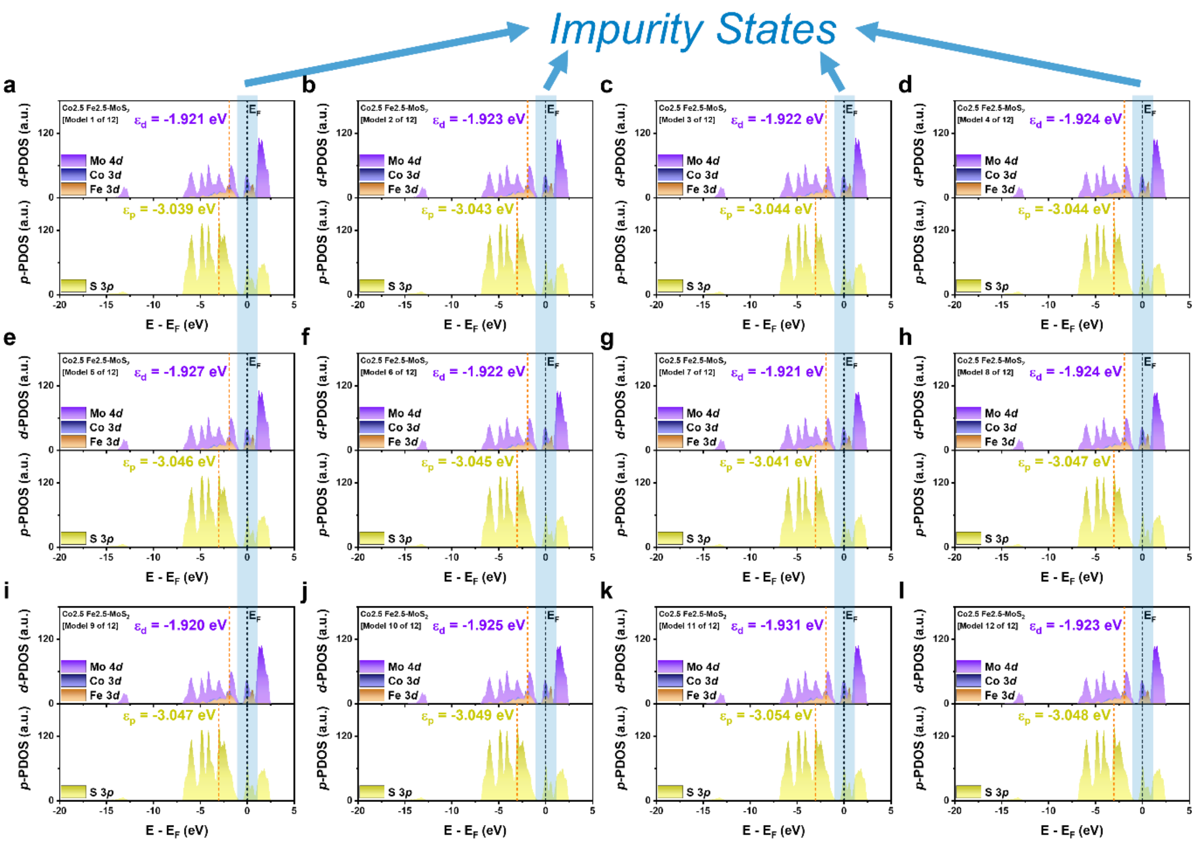

Supplementary Note Figure N36. (a–l) $\underline{d}$-PDOS of Mo 4*d*, Co 3*d*, Fe 3*d* and *p*-PDOS of S 3*p* orbital energy states of Co2.5 Fe2.5-MoS_2 [from Model 1 of 12 to Model 12 of 12].

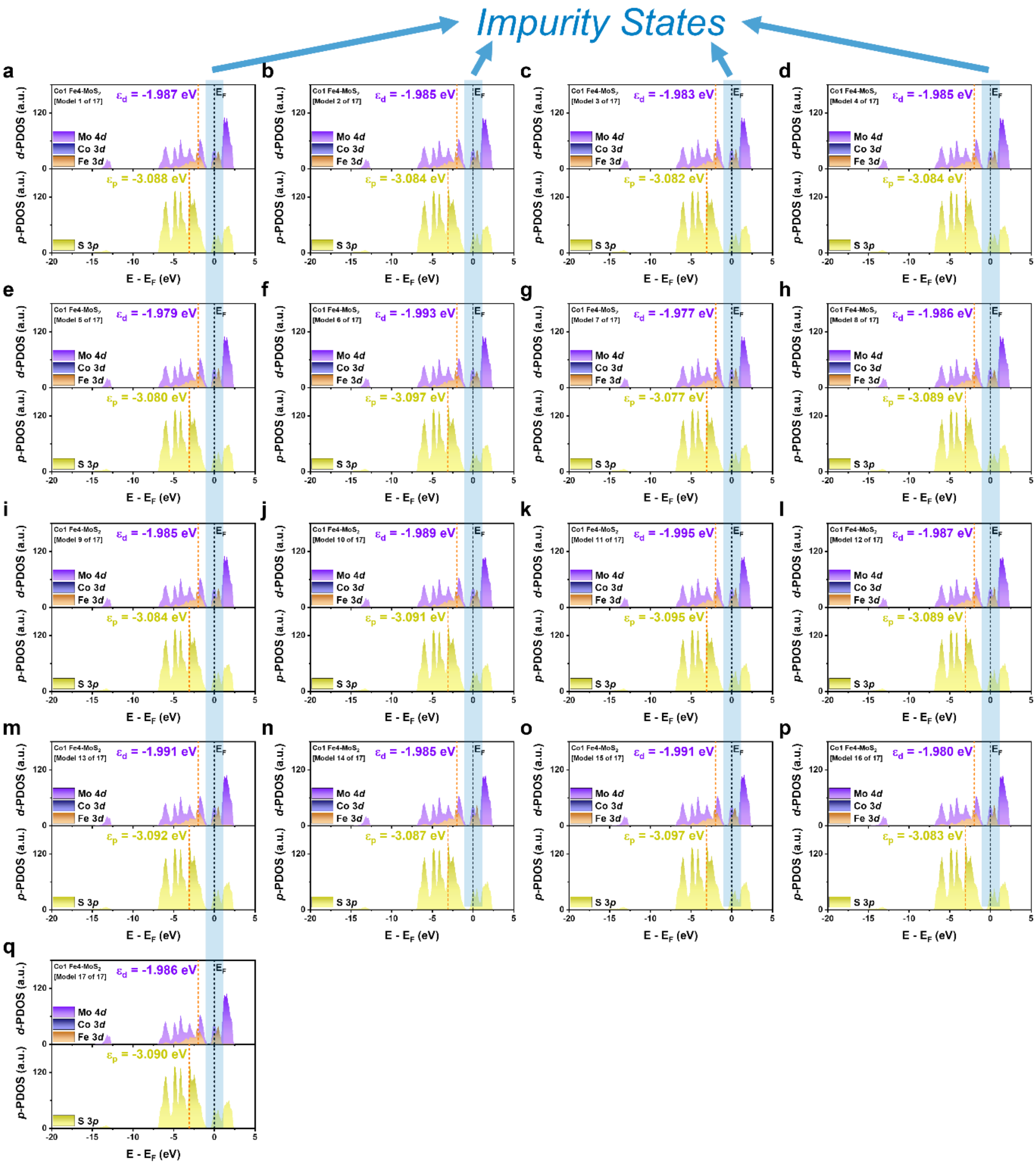

Supplementary Note Figure N37. (a–q) d-PDOS of Mo 4*d*, Co 3*d*, Fe 3*d* and *p*-PDOS of S 3*p* orbital energy states of Co1 Fe4-MoS_2 [from Model 1 of 17 to Model 17 of 17].

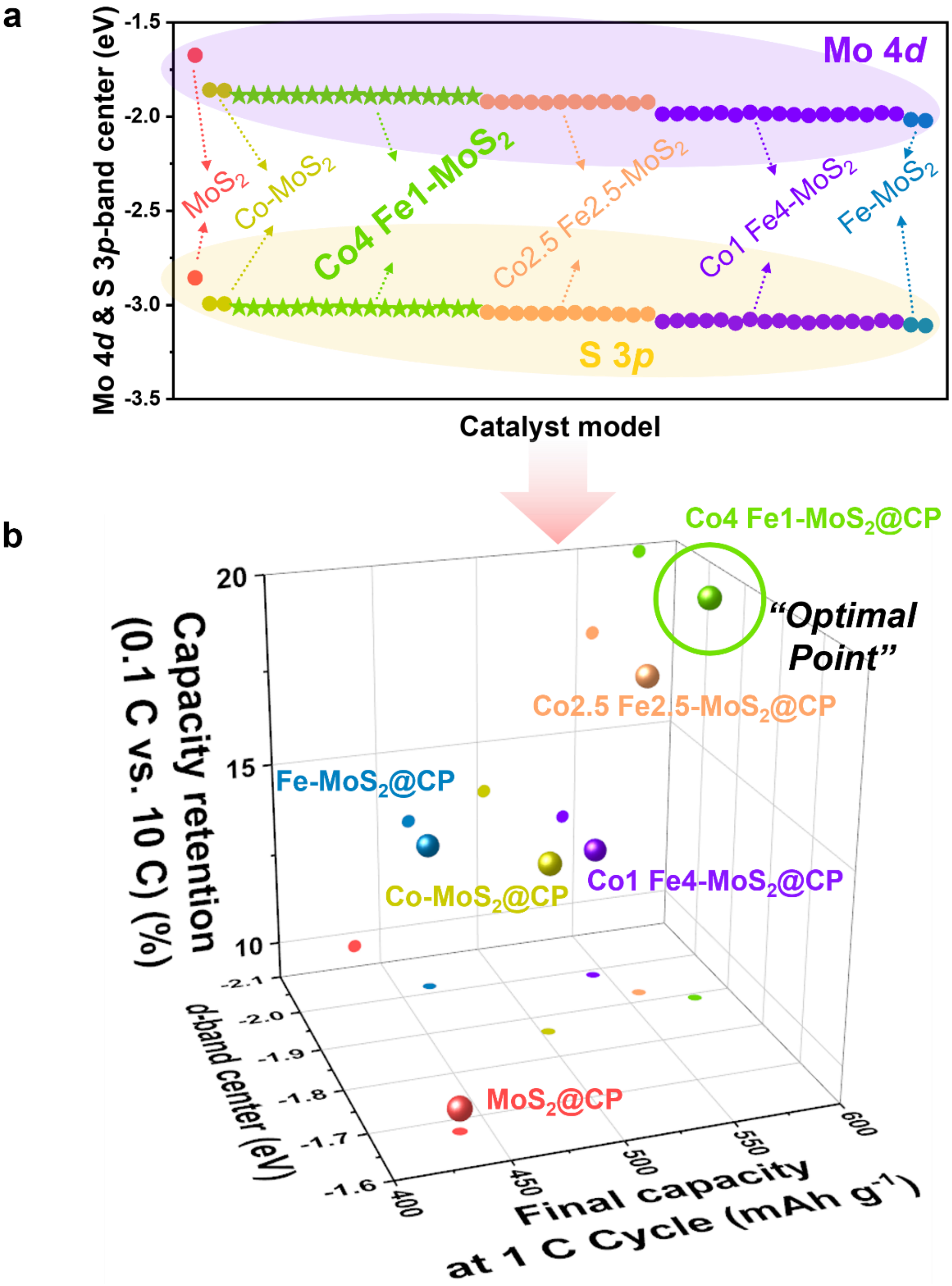

Supplementary Note Figure N38. (a) Calculated Mo 4*d* (*d*-band) and S 3*p* (*p*-band) centers for the investigated catalyst models: MoS_2, Co-MoS_2, Co4 Fe1-MoS_2 (CoFe-MoS_2), Co2.5 Fe2.5-MoS_2, Co1 Fe4-MoS_2, and Fe-MoS_2. (b) A three-way comparison of the cells based on (1) capacity retention (10 C vs. 0.1 C), (2) *d*-band center, and (3) final capacity at 1 C. This comparison highlights the optimal balance between adsorption capability and catalytic performance derived from these electronic descriptors.

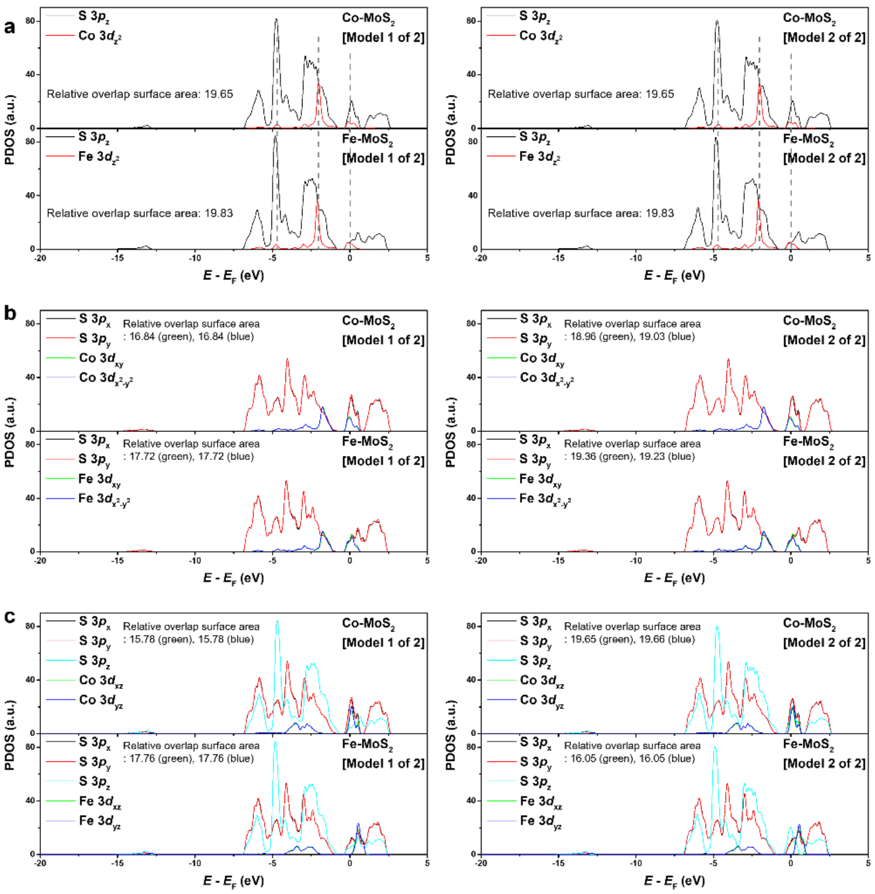

Figure S76. PDOS spectra of Co-MoS$_2$ and Fe-MoS$_2$ for orbital overlap between (a) S 3p_z and TM 3d_{z2}, (b) S 3p_x/p_y and TM 3d_{xy}/d_{x2-y2}, and (c) S 3p_{x}/p_{y}/p_z and TM 3d_{xz}/d_{yz} orbitals.

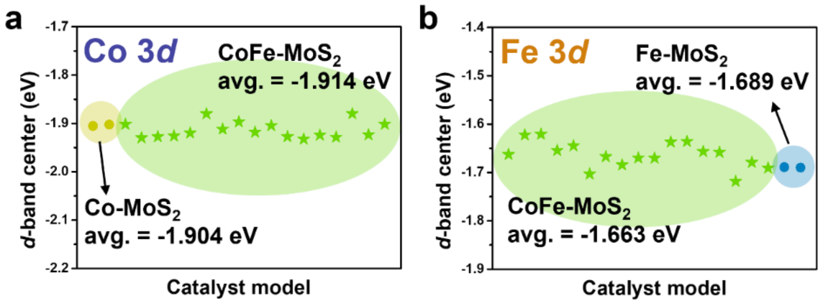

Figure S77. Average d-band center values of Co 3d and Fe 3d orbitals in the Co-MoS$_2$, CoFe-MoS$_2$, and Fe-MoS$_2$ models.

Table S1. ICP-OES results for Co-MoS_2, CoFe-MoS_2, and Fe-MoS_2 powders.

	Mass Con. (mg L^{-1})			Mole Con. (μmol L^{-1})			Mole ratio
Material	Co	Fe	Mo	Co	Fe	Mo	Mo : Co : Fe
Co-MoS_2	0.039	-	1.767	0.665	-	18.415	27.68 : 1 : 0
CoFe-MoS_2	0.039	0.009	2.108	0.660	0.157	21.969	26.87 : 0.81 : 0.19
Fe-MoS_2	-	0.058	2.754	-	1.033	28.702	27.78 : 0 : 1

Table S2. Structural properties (BET surface area, pore volume, and average pore diameter) of the composites.

Sample	Surface area ($m^2 g^{-1}$)	Pore volume ($cm^3 g^{-1}$)	Pore diameter (nm)
CP	75.1	0.115	6.13
MoS_2@CP	70.2	0.111	6.34
Co-MoS_2@CP	64.1	0.101	6.51
CoFe-MoS_2@CP	65.6	0.105	6.41
Fe-MoS_2@CP	68.7	0.102	6.11

Table S3. Fitting parameters of the Nyquist plots of the MoS_2@CP, Co-MoS_2@CP, CoFe-MoS_2@CP, and Fe-MoS_2@CP cells based on fitted equivalent circuits for different cycling conditions.

Samples	R_s (Ω)	R_{int} (Ω)	R_{ct} (Ω)
Before cycle			
MoS_2@CP	4.96	61.3	58.6
Co-MoS_2@CP	5.08	58.4	51.1
CoFe-MoS_2@CP	5.00	60.4	43.7
Fe-MoS_2@CP	5.11	66.6	54.2
After 500 cycle at 1.0 C rate			
MoS_2@CP	2.06	34.8	11.6
Co-MoS_2@CP	2.11	20.3	5.15
CoFe-MoS_2@CP	2.12	18.0	4.69
Fe-MoS_2@CP	2.08	28.6	7.59

Table S4. Comparison of the electrochemical performances measured in this work with those of previously reported Li-S batteries using similar systems.

Materials	Areal sulfur loading (mg cm^{-2})	E/S ratio (μL mg^{-1})	Rate and capacity	Capacity decay rate	Ref.
CoFe-MoS_2@CP	1.5	30	5 C / 466.4 mAh g^{-1}	0.028 % (2000 cycles)	Our work
	1.5	30	10 C / 340.9 mAh g^{-1}	0.024 % (2000 cycles)	
	6.2	9.7	0.1 C / 5.15 mAh cm^{-2}	0.118 % (200 cycles)	
OM-10/HWF/MWCNT	2.2	12.0	0.25 C / 1015 mAh g^{-1}	0.067 % (320 cycles)	[1]
	4.1	6.7	0.15 C / 3.9 mAh cm^{-2}	0.133 % (150 cycles)	
ZnS/MoS_2@Mxene	1.0	~25	1 C / 1150 mAh g^{-1}	0.071 % (500 cycles)	[2]
	3.1	11.3	0.2 C / 2.76 mAh cm^{-2}	0.165 % (100 cycles)	
	5.6	6.3	0.2 C / 4.10 mAh cm^{-2}	0.124 % (100 cycles)	
μFGF-MoS_2/C-TiN	2.5	10	1 C / 1000 mAh g^{-1} (2.5 mAh cm^{-2})	0.055 % (1000 cycles)	[3]
Co-MoS_2-G	1.0	30	1 C / 1000 mAh g^{-1}	0.029 % (1000 cycles)	[4]
	5.2	12	0.2 C / 801 mAh g^{-1} (4.17 mAh cm^{-2})	0.033 % (300 cycles)	
Ni-MoS_2/rGO	1.0	13	0.2 C / 1343 mAh g^{-1}	0.405 % (100 cycles)	[5]
			1 C / 950 mAh g^{-1}	0.139 % (400 cycles)	
MoS_2@CNT	1.5	15	0.5 C / 1291 mAh g^{-1}	0.089 % (500 cycles)	[6]
			1 C / 1200 mAh g^{-1}	0.222 % (200 cycles)	
S@MoS_2	1.0	44.2	0.5 C / 1300 mAh g^{-1}	0.128 % (300 cycles)	[7]
			1 C / 1197 mAh g^{-1}	0.076 % (300 cycles)	
CNT/MoS_2-Co	1.5	12	2 C / 900 mAh g^{-1}	0.051 % (800 cycles)	[8]

			5 C / 641 mAh g^{-1}	0.050 % (1000 cycles)	
	3.6	12	0.2 C / 882 mAh g^{-1} (3.17 mAh cm^{-2})	0.159 % (100 cycles)	
			0.5 C / 800 mAh g^{-1} (2.88 mAh cm^{-2})	0.156 % (150 cycles)	
CF@2H/1T MoS_2	~1.5	20	0.5 C / 1200 mAh g^{-1}	0.081 % (600 cycles)	[9]
			2 C / 1200 mAh g^{-1}	0.057 % (1000 cycles)	
TiO_2/C-MoS_2	1.0	80	0.2 C / 750 mAh g^{-1}	0.022 % (1500 cycles)	[10]
LE-MoS_2	4.6	5.7	0.24 C / 950 mAh g^{-1} (4.37 mAh cm^{-2})	0.140 % (150 cycles)	[11]
	6.1	4.3	0.18 C / 800 mAh g^{-1} (4.88 mAh cm^{-2})	0.250 % (100 cycles)	
CC@CS@HPP	5.6	15	0.1 C / 5.0 mAh cm^{-2}	0.667 % (30 cycles)	[12]
VN	4.3	12	0.1 C / 1200 mAh g^{-1} (5.16 mAh cm^{-2})	0.454 % (100 cycles)	[13]
CNTs-COOH@hemin	6.52	20	0.1 C / 1100 mAh g^{-1} (7.17 mAh cm^{-2})	0.259 % (200 cycles)	[14]
Ni-ZIF-8@CC	5.5	~14	0.13 C / 1098 mAh g^{-1} (6.04 mAh cm^{-2})	0.096 % (100 cycles)	[15]
Edg-MoS_2/C@PP	1.7	12	1 C / 935 mAh g^{-1}	0.047 % (1000 cycles)	[16]
MoS_{2-x}/rGO	1.5	~33.3	0.5 C / 1250 mAh g^{-1}	0.083 % (600 cycles)	[17]
NiS_2-MnS/MoS_2-3DNGr	1.0	30	0.1 C / 1011 mAh g^{-1}	0.120 % (200 cycles)	[18]
MoS_2-MoN	1,2	~66.7	1 C / 872.8 mAh g^{-1}	0.039 % (1000 cycles)	[19]
	1.2	~66.7	2 C / 778.0 mAh g^{-1}	0.041 % (1000 cycles)	
$(M\text{-}P/P)_{10}$	1.2	unknown	1 C / 1007 mAh g^{-1}	0.029 % (2000 cycles)	[20]
Mo_2C/α-MoO_3/G	4.2	9.3	0.1 C / 1055 mAh g-1 (4.43 mAh cm-2)	0.288 % (140 cycles)	[21]

Table S5. Fitting parameters of the Nyquist plots for charge transfer resistance of the MoS_2@CP, Co-MoS_2@CP, CoFe-MoS_2@CP, and Fe-MoS_2@CP cells during in-situ EIS measurement at 45 °C temperature condition.

Temperature: 45 °C	R_{ct} (Ω)			
Discharge state	MoS_2@CP	Co-MoS_2@CP	CoFe-MoS_2@CP	Fe-MoS_2@CP
2.80 V (vs. Li/Li^+)	4.93	5.64	5.60	4.31
2.70 V (vs. Li/Li^+)	5.02	5.90	5.20	4.57
2.60 V (vs. Li/Li^+)	5.14	5.94	5.55	4.70
2.50 V (vs. Li/Li^+)	5.27	5.98	5.26	4.60
2.40 V (vs. Li/Li^+)	5.48	6.14	5.26	4.77
2.30 V (vs. Li/Li^+)	5.37	6.27	5.28	4.62
2.20 V (vs. Li/Li^+)	5.81	6.35	5.27	4.90
2.10 V (vs. Li/Li^+)	5.95	6.50	5.20	5.07
2.00 V (vs. Li/Li^+)	30.5	17.4	15.1	22.5
1.90 V (vs. Li/Li^+)	30.7	17.1	15.3	22.5
1.80 V (vs. Li/Li^+)	30.4	17.1	15.0	22.1
1.70 V (vs. Li/Li^+)	32.6	17.0	15.0	21.8

Table S6. Fitting parameters of the Nyquist plots for charge transfer resistance of the MoS_2@CP, Co-MoS_2@CP, CoFe-MoS_2@CP, and Fe-MoS_2@CP cells during in-situ EIS measurement at 55 °C temperature condition.

Temperature: 55 °C	R_{ct} (Ω)			
Discharge state	MoS_2@CP	Co-MoS_2@CP	CoFe-MoS_2@CP	Fe-MoS_2@CP
2.80 V (vs. Li/Li^+)	5.62	4.61	4.00	4.44
2.70 V (vs. Li/Li^+)	5.90	4.52	4.24	4.73
2.60 V (vs. Li/Li^+)	6.00	4.74	4.50	4.69
2.50 V (vs. Li/Li^+)	6.00	4.60	4.62	4.78
2.40 V (vs. Li/Li^+)	6.05	4.75	4.75	4.83
2.30 V (vs. Li/Li^+)	6.16	4.77	4.83	4.92
2.20 V (vs. Li/Li^+)	6.28	4.60	4.77	4.90
2.10 V (vs. Li/Li^+)	6.25	4.61	4.84	5.05
2.00 V (vs. Li/Li^+)	17.1	12.9	12.2	14.6
1.90 V (vs. Li/Li^+)	17.8	13.1	12.5	15.4
1.80 V (vs. Li/Li^+)	19.2	12.5	12.1	15.7
1.70 V (vs. Li/Li^+)	19.4	12.3	12.1	15.4

Table S7. Fitting parameters of the Nyquist plots for charge transfer resistance of the MoS_2@CP, Co-MoS_2@CP, CoFe-MoS_2@CP, and Fe-MoS_2@CP cells during in-situ EIS measurement at 65 °C temperature condition.

Temperature: 65 °C	R_{ct} (Ω)			
Discharge state	MoS_2@CP	Co-MoS_2@CP	CoFe-MoS_2@CP	Fe-MoS_2@CP
2.80 V (vs. Li/Li^+)	3.01	3.67	3.51	2.68
2.70 V (vs. Li/Li^+)	3.14	3.88	3.82	2.89
2.60 V (vs. Li/Li^+)	3.31	4.02	3.93	3.02
2.50 V (vs. Li/Li^+)	3.29	4.09	3.97	3.00
2.40 V (vs. Li/Li^+)	3.39	4.23	4.12	3.04
2.30 V (vs. Li/Li^+)	3.39	4.24	3.94	3.13
2.20 V (vs. Li/Li^+)	3.50	4.25	4.06	3.18
2.10 V (vs. Li/Li^+)	3.51	4.32	3.99	3.28
2.00 V (vs. Li/Li^+)	12.2	10.5	10.0	11.8
1.90 V (vs. Li/Li^+)	14.2	11.3	10.2	12.4
1.80 V (vs. Li/Li^+)	15.3	11.3	10.4	13.3
1.70 V (vs. Li/Li^+)	15.8	11.2	10.5	13.0

Table S8. Calculated activation energy (E_a) value from the Arrhenius equation of the MoS_2@CP, Co-MoS_2@CP, CoFe-MoS_2@CP, and Fe-MoS_2@CP cells.

Temperature: 65 °C	E_a (eV)			
Discharge state	MoS_2@CP	Co-MoS_2@CP	CoFe-MoS_2@CP	Fe-MoS_2@CP
2.80 V (vs. Li/Li^+)	0.225	0.220	0.197	0.217
2.70 V (vs. Li/Li^+)	0.214	0.202	0.136	0.209
2.60 V (vs. Li/Li^+)	0.200	0.192	0.149	0.204
2.50 V (vs. Li/Li^+)	0.215	0.190	0.116	0.196
2.40 V (vs. Li/Li^+)	0.219	0.186	0.101	0.206
2.30 V (vs. Li/Li^+)	0.210	0.216	0.102	0.177
2.20 V (vs. Li/Li^+)	0.232	0.208	0.099	0.199
2.10 V (vs. Li/Li^+)	0.242	0.227	0.086	0.200
2.00 V (vs. Li/Li^+)	0.424	0.256	0.166	0.300
1.90 V (vs. Li/Li^+)	0.360	0.241	0.144	0.275
1.80 V (vs. Li/Li^+)	0.320	0.230	0.118	0.235
1.70 V (vs. Li/Li^+)	0.338	0.226	0.127	0.242

Table S9. Determination of the growth type and discharge capacity of Li_2S precipitation reaction for the MoS_2@CP, Co-MoS_2@CP, CoFe-MoS_2@CP, and Fe-MoS_2@CP cells with recorded I_m and t_m values from current-time transients.

Cell	I_m (mA)	t_m (s)	Nuclei growth type	Li_2S capacity (mAh g^{-1})
MoS_2@CP	0.440	5,136	2DI-3DP	404.0
Co-MoS_2@CP	0.275	5,613	3DP	567.6
CoFe-MoS_2@CP	0.214	5,662	3DP-3DI	589.4
Fe-MoS_2@CP	0.380	6,134	2DI-3DP	527.5

Table S10. Curve fitting results for the Mo K-edge EXAFS spectra of the electrode samples.

Samples	Bond	Coordination number	Debye-Waller factor σ^2 (x 10^{-3} Å^2)	Bond length (Å)	R-factor
MoS_2@CP State: Prsitine	Mo-S	6.0	2.840 (± 0.318)	2.404 (± 0.005)	0.0350615
	Mo-Mo	6.0	3.620 (± 0.247)	3.169 (± 0.005)	
	Mo-S	6.0	8.510 (± 3.596)	3.967 (± 0.033)	
MoS_2@CP State: Cycled	Mo-S	6.0	2.740 (± 0.395)	2.408 (± 0.004)	0.0217274
	Mo-Mo	6.0	5.070 (± 0.363)	3.169 (± 0.004)	
	Mo-S	6.0	41.950 (± 50.254)	3.877 (± 0.253)	
Co-MoS_2@CP State: Prsitine	Mo-S	6.0	2.820 (± 0.241)	2.406 (± 0.004)	0.0243267
	Mo-Mo	6.0	4.390 (± 0.231)	3.170 (± 0.004)	
	Mo-S	6.0	13.680 (± 6.101)	4.001 (± 0.047)	
Co-MoS_2@CP State: Cycled	Mo-S	6.0	3.160 (± 0.282)	2.410 (± 0.005)	0.0313208
	Mo-Mo	6.0	4.880 (± 0.283)	3.172 (± 0.005)	
	Mo-S	6.0	38.220 (± 49.218)	3.915 (± 0.239)	
CoFe-MoS_2@CP State: Prsitine	Mo-S	6.0	2.760 (± 0.244)	2.406 (± 0.004)	0.0244645
	Mo-Mo	6.0	4.180 (± 0.225)	3.170 (± 0.004)	
	Mo-S	6.0	15.310 (± 7.624)	3.989 (± 0.056)	
CoFe-MoS_2@CP State: Cycled	Mo-S	6.0	2.960 (± 0.272)	2.406 (± 0.005)	0.0318299
	Mo-Mo	6.0	4.840 (± 0.283)	3.170 (± 0.005)	
	Mo-S	6.0	22.180 (± 16.698)	3.977 (± 0.105)	
Fe-MoS_2@CP State: Prsitine	Mo-S	6.0	2.620 (± 0.272)	2.409 (± 0.004)	0.0301180
	Mo-Mo	6.0	3.930 (± 0.242)	3.172 (± 0.004)	
	Mo-S	6.0	14.140 (± 7.637)	3.999 (± 0.058)	
Fe-MoS_2@CP State: Cycled	Mo-S	6.0	3.180 (± 0.267)	2.408 (± 0.004)	0.0281947
	Mo-Mo	6.0	4.700 (± 0.254)	3.170 (± 0.004)	
	Mo-S	6.0	15.860 (± 8.092)	3.997 (± 0.059)	

Supporting Information References

[1] X. Ren, H. Wu, Y. Guo, H. Wei, H. Wu, H. Wang, Z. Lin, C. Xiong, H. Liu, L. Zhang, Z. Li, *Small* **2024**, *20*, 2312256.

[2] Y. Li, C. Xu, D. Li, Y. Zhang, B. Liu, P. Huo, *Chem. Eng. J.* **2024**, *502*, 158151.

[3] M. Waqas, Y. Han, D. Chen, S. Ali, C. Zhen, C. Feng, B. Yuan, J. Han, W. He, *Energy Storage Mater.* **2020**, *27*, 333.

[4] W. Liu, C. Luo, S. Zhang, B. Zhang, J. Ma, X. Wang, W. Liu, Z. Li, Q.-H. Yang, W. Lv, *ACS Nano* **2021**, *15*, 7491.

[5] R. Zhang, Y. Dong, M. A. Al-Tahan, Y. Zhang, R. Wei, Y. Ma, C. Yang, J. Zhang, *J. Energy Chem.* **2021**, *60*, 85.

[6] Y. Li, Q. Yan, J. Zhu, Y. Ren, Q. Liu, Y. Hou, J. Lu, X. Gao, X. Zhan, Q. Zhang, *ACS Sustain. Chem. Eng.* **2023**, *11*, 1019.

[7] Y. Yi, Z. Liu, P. Yang, T. Wang, X. Zhao, H. Huang, Y. Cheng, J. Zhang, M. Li, *J. Energy Chem.* **2020**, *45*, 18.

[8] Z. Ma, Y. Liu, J. Gautam, W. Liu, A. N. Chishti, J. Gu, G. Yang, Z. Wu, J. Xie, M. Chen, L. Ni, G. Diao, *Small* **2021**, *17*, 2102710.

[9] C. Tian, B. Li, X. Hu, J. Wu, P. Li, X. Xiang, X. Zu, S. Li, *ACS Appl. Mater. Interfaces* **2021**, *13*, 6229.

[10] Y. Yang, S. Wang, S. Lin, Y. Li, W. Zhang, Y. Chao, M. Luo, Y. Xing, K. Wang, C. Yang, P. Zhou, Y. Zhang, Z. Tang, S. Guo, *Small Methods* **2018**, *2*, 1800119.

[11] Y. Pan, L. Gong, X. Cheng, Y. Zhou, Y. Fu, J. Feng, H. Ahmed, H. Zhang, *ACS Nano* **2020**, *14*, 5917.

[12] Z. Ye, Y. Jiang, L. Li, F. Wu, R. Chen, *Adv. Mater.* **2020**, *32*, 2002168.

[13] R. Liu, W. Liu, Y. Bu, W. Yang, C. Wang, C. Priest, Z. Liu, Y. Wang, J. Chen, Y. Wang, J. Cheng, X. Lin, X. Feng, G. Wu, Y. Ma, W. Huang, *ACS Nano* **2020**, *14*, 17308.

[14] X. Ding, S. Yang, S. Zhou, Y. Zhan, Y. Lai, X. Zhou, X. Xu, H. Nie, S. Huang, Z. Yang, *Adv. Funct. Mater.* **2020**, *30*, 2003354.

[15] Y. Yang, Z. Wang, T. Jiang, C. Dong, Z. Mao, C. Lu, W. Sun, K. Sun, *J. Mater. Chem. A* **2018**, *6*, 13593.

[16] N. Zheng, G. Jiang, X. Chen, J. Mao, N. Jiang, Y. Li, *Nano-Micro Lett.* **2019**, *11*, 43.

[17] H. Lin, L. Yang, X. Jiang, G. Li, T. Zhang, Q. Yao, G. W. Zheng, J. Y. Lee, *Energy Environ. Sci.* **2017**, *10*, 1476.

[18] T. L. L. Doan, D. C. Nguyen, R. Amaral, N. Y. Dzade, C. S. Kim, C. H. Park, *Appl. Catal. B Environ.* **2022**, *319*, 121934.

[19] S. Wang, S. Feng, J. Liang, Q. Su, F. Zhao, H. Song, M. Zheng, Q. Sun, Z. Song, X. Jia, J. Yang, Y. Li, J. Liao, R. Li, X. Sun, *Adv. Energy Mater.* **2021**, *11*, 2003314.

[20] J. Wu, H. Zeng, X. Li, X. Xiang, Y. Liao, Z. Xue, Y. Ye, X. Xie, *Adv. Energy Mater.* **2018**, *8*, 1802430.

[21] S. Du, Y. Yu, X. Liu, D. Lu, X. Yue, T. Liu, Y. Yin, Z. Wu, *Chem. Eng. J.* **2024**, *500*, 157002.
